# New potent *N*‐hydroxycinnamamide‐based histone deacetylase inhibitors suppress proliferation and trigger apoptosis in THP‐1 leukaemia cells

**DOI:** 10.1002/ardp.202400889

**Published:** 2025-04-01

**Authors:** Magdalena Onuscakova, Tereza Kauerova, Eva Fialova, Hana Pizova, Vladimir Garaj, Miroslav Kemka, Vladimir Frecer, Peter Kollar, Pavel Bobal

**Affiliations:** ^1^ Department of Chemical Drugs, Faculty of Pharmacy Masaryk University Brno Czech Republic; ^2^ Department of Pharmacology and Toxicology, Faculty of Pharmacy Masaryk University Brno Czech Republic; ^3^ Department of Pharmaceutical Chemistry, Faculty of Pharmacy Comenius University Bratislava Bratislava Slovakia; ^4^ Department of Physical Chemistry of Drugs, Faculty of Pharmacy Comenius University Bratislava Bratislava Slovakia

**Keywords:** anticancer agents, HDACi, haematological malignancies, hydroxamic acid, inhibitors of histone deacetylases

## Abstract

A new group of potent histone deacetylase inhibitors (HDACis) capable of inhibiting cell growth and affecting cell‐cycle progression in Tohoku Hospital Pediatrics‐1 (THP‐1) monocytic leukaemia cells was synthesized. The inhibitors belong to a series of hydroxamic acid derivatives. We designed and synthesized a series of 22 *N*‐hydroxycinnamamide derivatives, out of which 20 are new compounds. These compounds contain various substituted anilides as the surface recognition moiety (SRM), a *p*‐hydroxycinnamate linker, and hydroxamic acids as the zinc‐binding group (ZBG). The whole series of synthesized hydroxamic acids inhibited THP‐1 cell proliferation. Compounds **7d** and **7p**, which belong to the category of derivatives with the most potent antiproliferative properties, exert a similar effect on cell‐cycle progression as vorinostat and induce apoptosis in THP‐1 cells. Furthermore, compounds **7d** and **7p** were demonstrated to inhibit HDAC class I and II in THP‐1 cells with comparable potency to vorinostat and increase acetylation of histones H2a, H2b, H3, and H4. Molecular modelling was used to predict the probable binding mode of the studied HDACis in class I and II histone deacetylases in terms of Zn^2+^ ion chelation by the hydroxamate group.

## Graphical abstract

New *N*‐hydroxycinnamamide derivatives were synthesized using a multistep reaction procedure. These compounds significantly inhibited cell growth and affected cell‐cycle progression in THP‐1 monocytic leukaemia cells. They inhibit histone deacetylases class I and II and trigger apoptosis in THP‐1 cells with comparable potency to vorinostat.

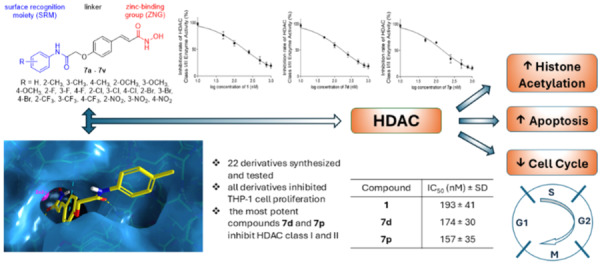

## 1 INTRODUCTION

Epigenetic mechanisms were shown to regulate gene transcription, help maintain genomic stability and integrity and contribute to normal cell growth and differentiation. Epigenetic aberrations lead to dysregulation of gene expression and genetic instability without permanent changes in the genomic sequence.^[^ ^1^, ^2^, ^3^ ^]^ These alterations could result in epigenetic silencing of tumour suppressors or can contribute to cancer development by activating oncogenes.^[^ ^4^, ^5^, ^6^ ^]^ An important aspect of epigenetic modifications is the fact that epigenetic alterations are potentially reversible. Epigenetic drugs could induce the reactivation of tumour suppressors; thus, normal cell processes and functioning could be re‐established.^[^ ^7^, ^8^, ^9^ ^]^

In general, the mechanisms of epigenetic modifications in tumourigenesis consist of three parts: DNA and RNA methylations, histone modifications and regulatory effects of noncoding RNAs (ncRNAs).^[^ ^3^ ^]^ Histone modifications, including the process of histone acetylation, are pivotal in the epigenetic regulation of gene expression. They alter the accessibility of transcription factors and other proteins to their DNA binding sites through chromatin remodeling.^[^ ^10^ ^]^ Two well‐characterized groups of enzymes regulate histone acetylation: histone acetyltransferases (HATs) and histone deacetylases (HDACs).^[^ ^11^ ^]^ While histone acetylation induces weakening of the DNA–histone interaction and thus makes the promoter region of target genes available, deacetylation causes compaction of the chromatin structure and therefore it is mainly related to gene repression.^[^ ^12^ ^]^ The imbalance of histone acetylation and deacetylation was shown to be associated with tumourigenesis, while the relationship between histone alterations and cancer has been extensively studied, mainly in haematologic malignancies.^[^ ^13^ ^]^

Treatment of haematologic malignancies, in particular, is the main area of therapeutic use of histone deacetylase inhibitors (HDACis).^[^ ^14^ ^]^ HDACis induce increased histone acetylation, so the structure of acetylated chromatin is less condensed, allowing the transcription complex to access target sites. Therefore, it enhances transcriptional activity and reinstates gene expression control over critical cellular processes such as the cell cycle, cytodifferentiation and apoptosis. However, the cellular response to this action remains intricately multifaceted.^[^ ^15^, ^16^, ^17^ ^]^

**Figure 1:**
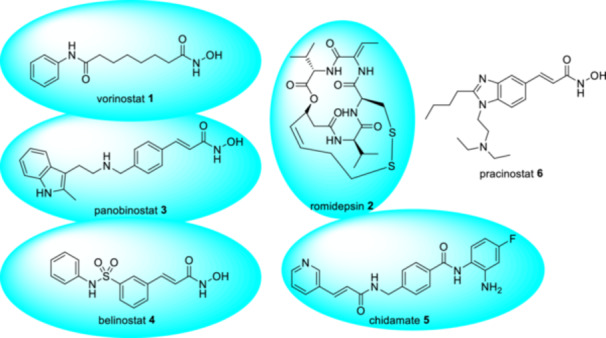
Structures of approved histone deacetylase (HDAC) inhibitors and pracinostat as drug previously developed under Orphan Designation.

A lot of HDACis are in clinical development for oncology, but only five of them have been approved for cancer chemotherapy so far (Figure 1). Currently, the most important group of HDACis is hydroxamic acid derivatives. Following hydroxamic acid‐based HDACis are registered as anticancer drugs: vorinostat to treat advanced cutaneous T‐cell lymphoma (CTCL), panobinostat to treat multiple myeloma in combination with bortezomib and dexamethasone, belinostat and tucidinostat (chidamide) to treat peripheral T‐cell lymphoma (PTCL). However, these drugs are still under investigation in terms of their possible use in other indications.^[^ ^13^, ^15^, ^18^, ^19^, ^20^ ^]^

**Figure 2:**
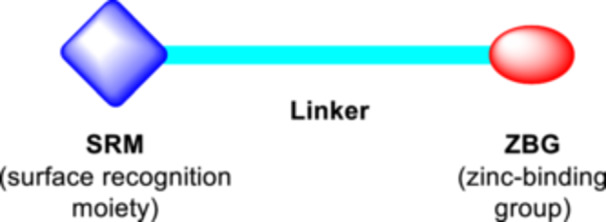
A typical three‐feature pharmacophore of histone deacetylase inhibitors (HDACis) contains a zinc‐binding group (ZBG), a linker, and a surface recognition moiety (SRM).

HDACs are versatile players in cellular regulation, impacting fundamental processes such as cell‐cycle progression, differentiation and tumourigenesis. Abnormal HDAC function can contribute to the development of many different human diseases, including cancer, lung disease, cardiac hypertrophy and neurodegenerative disorders.^[^ ^21^ ^]^ Therefore, discovering new selective HDACis may be beneficial in various areas of human medicine. The HDACs are responsible for the deacetylation of lysine residues on the *N*‐terminal part of the core histones. In humans, 18 different HDAC enzymes are divided into four classes.^[^ ^22^ ^]^ HDACis form a complex with the Zn^2+^ ion in the catalytic pocket of enzymes, leading to considerable anticancer activity.^[^ ^23^ ^]^ Based on the common three‐feature pharmacophore model of HDACi (Figure 2 ^[^ ^24^ ^]^), we have designed and synthesized a series of *N*‐hydroxycinnamamide derivatives containing various substituted anilides as the surface recognition moiety, *p*‐hydroxybenzaldehyde linker, and hydroxamic acids as the zinc‐binding group.

## 2 RESULTS AND DISCUSSION

### 2.1 Chemistry

**Scheme 1:**
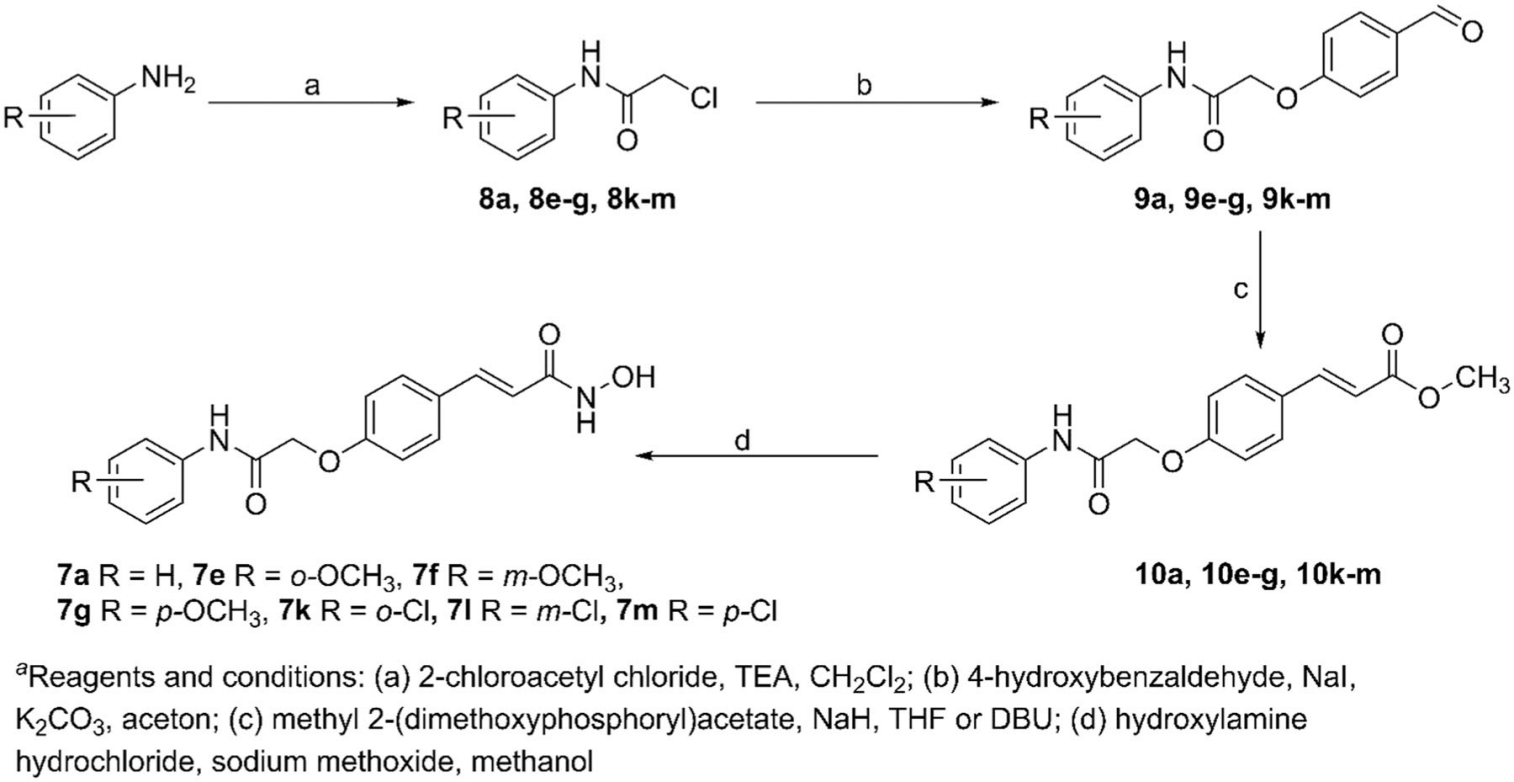
The first attempts for the synthesis of the target histone deacetylase (HDAC) inhibitors.

The first attempt to synthesize HDAC inhibitors containing hydroxamic acid are described in Scheme 1. In the pilot experiments, hydroxamic acids **7a, 7e–g**, and **7k–m** were prepared by linear synthesis. This four‐step synthesis was based on the reaction of *p*‐hydroxybenzaldehyde with seven substituted 2‐chloro‐*N*‐phenylacetamides **8a**, **8e–g**, and **8k–m** prepared from the corresponding anilines and 2‐chloroacetyl chloride in the presence of triethylamine. The phenyl‐substituted aldehydes **9a**, **9e–g**, and **9k–m** were then coupled with methyl 2‐(dimethoxyphosphoryl)acetate under Horner–Wadsworth–Emmons conditions to form ester intermediates **10a**, **10e–g**, **10k–m**.^[^ ^25^ ^]^ The preparation of HDACis continued by reacting the intermediates with hydroxylamine in the presence of a significant excess of sodium methoxide to produce hydroxamic acids **7a**, **7e–g**, and **7k–m**. Although this linear method allowed us to obtain the desired inhibitors, it was identified as ineffective for synthesizing an extensive library of compounds due to the very low overall yields.

**Scheme 2:**
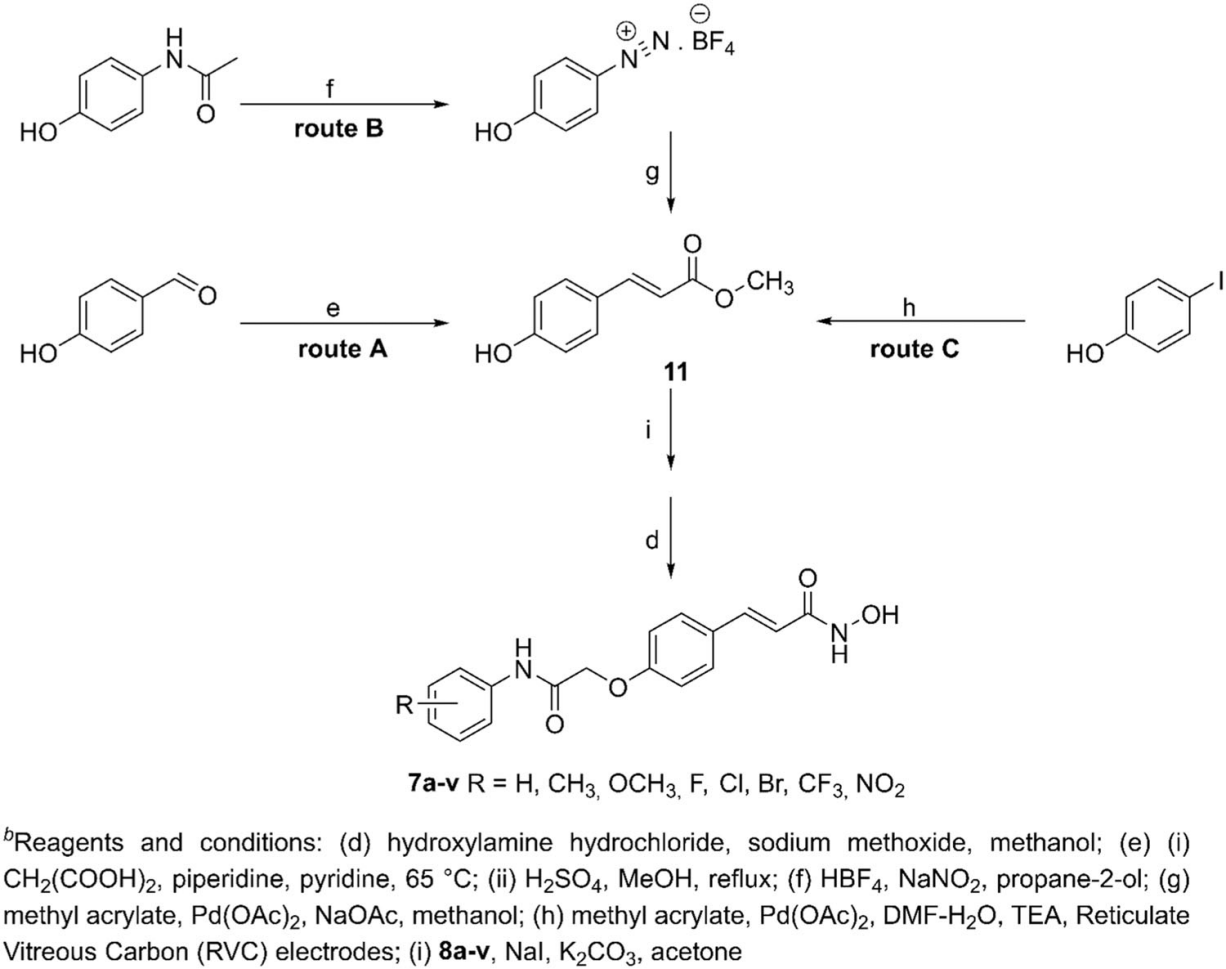
Convergent variants of the synthesis of inhibitors **7a–v** ^*b*^.

Therefore, we proposed a convergent chemical synthesis of the target compounds outlined in Scheme 2, divided into three possible routes to obtain methyl *p*‐hydroxycinnamate **11** as an essential intermediate. In route **A**, *p*‐hydroxybenzaldehyde reacted with malonic acid under basic conditions, followed by esterification of the resulting *p*‐hydroxycinnamic acid.^[^ ^26^ ^]^ Although this procedure was more tedious, the reaction yields were higher. The other route **B** starts with *p*‐hydroxyacetanilide, which was converted to 4‐hydroxybenzene‐1‐diazonium tetrafluoroborate.^[^ ^27^ ^]^ The diazonium salt underwent palladium‐catalysed Mizoroki–Heck cross‐coupling reactions with methyl acrylate to generate intermediate **11** in good yield. Still, the isolation of the intermediate required chromatographic purification.^[^ ^27^ ^]^ The last route **C**, which has been tested, uses an electrochemical reactor with reticulate vitreous carbon (RVC) electrodes. An electrochemical reaction between *p*‐iodophenol and methyl acrylate in a basic medium catalysed by Pd(OAc)_2_ led to the formation of methyl *p*‐hydroxycinnamate **11** in high yield and purity. However, this method was limited by the size of the electrochemical reaction vessel.^[^ ^28^ ^]^

For this reason, most of intermediate **11** was prepared by method **A**. The methyl ester intermediates **10a–v** were easily derived from methyl *p*‐hydroxycinnamate **11** and the number of 22 substituted 2‐chloro‐*N*‐phenylacetamides **8a–v** by standard nucleophilic substitution as described in Scheme 1. The ethyl esters **10a–v** were subjected to a hydroxylaminolysis as described above. Overall yields of these convergent syntheses of hydroxamic acid inhibitors **7a–v** were much better than in the case of the linear one.

**Scheme 3:**
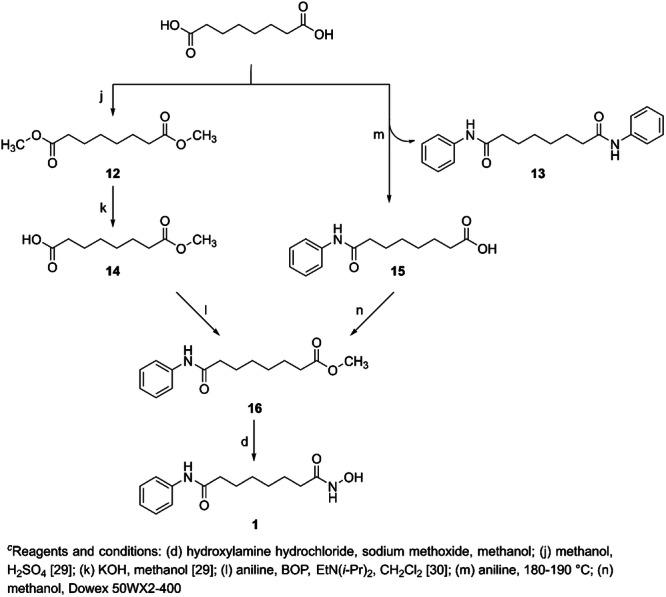
Two routes for the synthesis of vorinostat (SAHA)^*c*^.

A few years ago, due to the shortage of suberoylanilide hydroxamic acid (SAHA, vorinostat **1**) in the market, we undertook its synthesis, as illustrated in Scheme 3. Two possible paths have been tested, starting from suberic acid (octanedioic acid). In the first (pathway **A**), suberic acid was esterified by acid‐catalysed reaction with anhydrous methanol at a yield of 79%, followed by partial hydrolysis to monoester **14**, which was isolated by extraction with hexane.^[^ ^29^ ^]^ The efficiency of the partial hydrolysis step and separation was only 37%. Intermediate **14** and aniline were combined with benzotriazol‐1‐yloxytris reagent and tertiary amine. Terminal intermediate **16** was isolated with a yield of 98%. The subsequent formation of SAHA **1** was achieved by reaction with hydroxylamine in the presence of an excess of sodium methoxide in a yield of 87%. The second synthetic pathway **B** for obtaining SAHA was based on the thermal reaction of suberic acid with an excess of freshly distilled aniline. Monoanilide **15** was isolated from dianilide **13** by extraction at different pH, yielding 41%. The following esterification with methanol was catalysed by Dowex resin to yield intermediate **16.** ^[^ ^30^ ^]^ The yield of this reaction was 85%. Intermediate **16** was further converted to SAHA **1** by the method described above.

### 2.2 Molecular docking: Virtual screening of hydroxamate inhibitors

Until now, 18 human HDACs divided into four classes have been identified. Class I (HDAC1, 2, 3, 8), class IIa and IIb (HDAC4, 5, 7, 9 and 6, 10), class III (Sirtuins), and class IV (HDAC11). Class I, II, and IV HDACs are zinc‐dependent deacetylases,^[^ ^31^ ^]^ which share a common pharmacophore model of their inhibitors. Typically, HDACis contain a zinc‐binding group, such as the hydroxamate group, which chelates the Zn^2+^ ion in a bidentate manner.^[^ ^32^ ^]^ At the same time, the chelating group forms hydrogen bonds with the neighbouring histidine and catalytic tyrosine residues, further stabilizing the zinc coordination.^[^ ^33^ ^]^ In addition, inhibitors may include another group targeting a foot pocket present in class I HDACs.^[^ ^34^ ^]^

Molecular modelling strategies are regularly used to predict the binding affinities of drugs designed for targeted receptors or to rationalize the observed potencies of a series of bioactive compounds.^[^ ^35^, ^36^, ^37^ ^]^ Molecular docking studies can be used to predict the binding mode of ligands, estimate the binding affinity of protein–ligand complexes, and prioritize compounds according to their binding affinity.^[^ ^35^, ^38^ ^]^ We have used molecular docking to compare predicted binding affinities of a series of a new group of hydroxamic acid derivatives to HDACs of class I and II (HDAC1, 2, 3, 8 and 4, 6, 7) derived from in silico screening with inhibitory concentrations IC_50_ of antiproliferative effect observed in vitro on the human leukaemia monocytic Tohoku Hospital Pediatrics‐1 (THP‐1) cell line (Table 3, see below). THP‐1 cells express the family of class I HDAC genes ubiquitously, while the expression of class II HDACs is more restricted.^[^ ^39^ ^]^ The purpose of our computational study was to investigate whether the observed suppression of THP‐1 cell proliferation could be attributed to the inhibition of a specific HDAC isoform.

**Figure 3:**
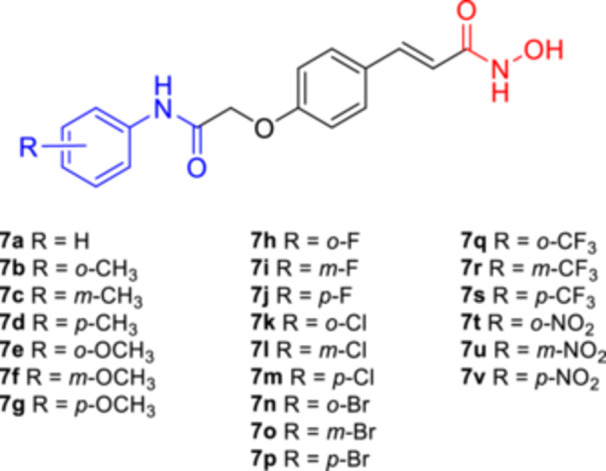
A library of compounds studied.

**Figure 4:**
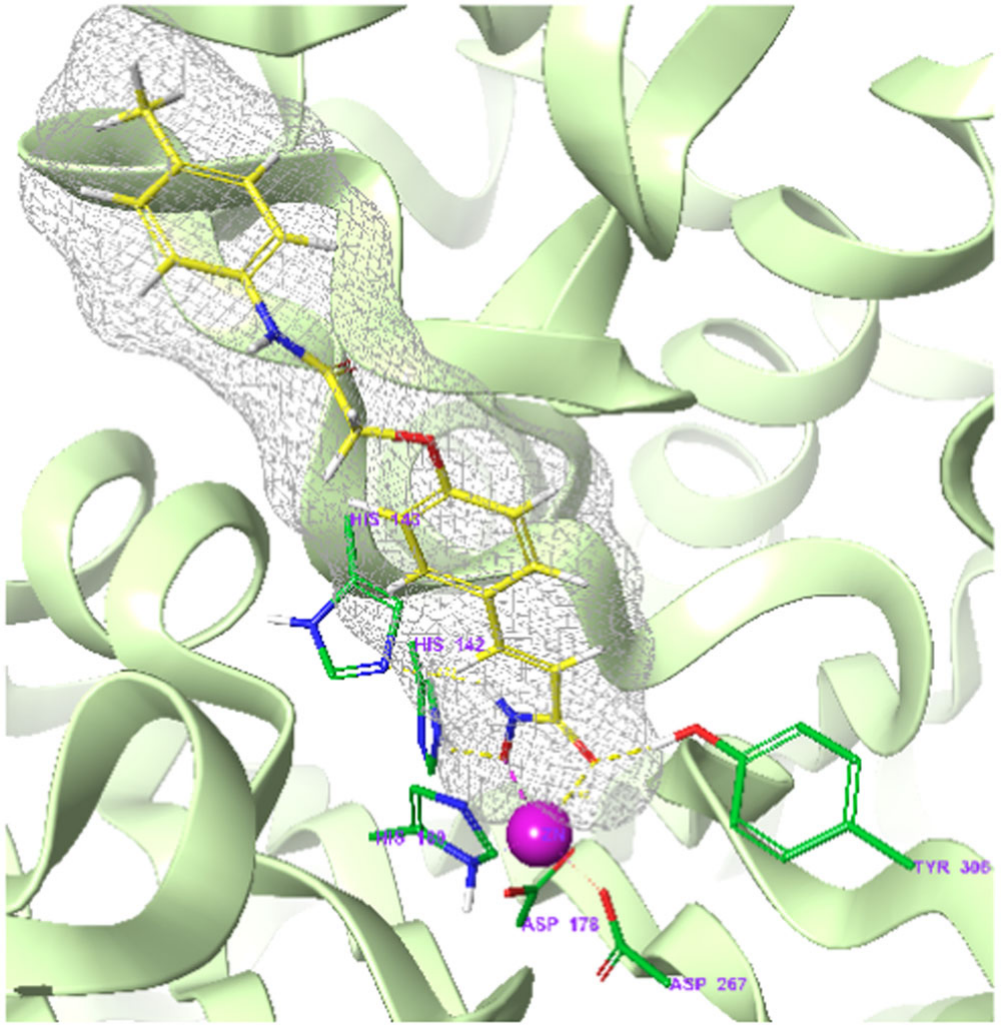
Binding mode of the most potent hydroxamic acid derivative **7d** and three‐dimensional (3D) structure of the HDAC8 binding site predicted by molecular docking using the Glide XP protocol (release 2022‐3, Schrödinger LLC, USA, 2022^[^ ^40^, ^41^ ^]^). The protein backbone is shown as a green ribbon, the mesh type molecular surface of **7d** is shown in grey colour, the atom colouring: ligand C – yellow, protein C – green, N – blue, O – red, H – light grey, Zn – purple.

All hydroxamic acid derivatives **7a**–**7v** (Figure 3) were docked to refined crystal structures of HDAC‐inhibitor complexes for 7 class I and class II HDACs using the Glide XP protocol (Release 2022‐3, Schrödinger LLC, USA, 2022^[^ ^40^, ^41^ ^]^) for flexible small‐molecule ligands considering the three types of zinc ion chelation (Figure 8). Computed XP GlideScore values (which represents an estimate of ligand binding affinity to the targeted HDAC receptor) for the derivatives tested assuming bidentate zinc ion chelation type **C** are shown in Table 1 (docking scores for type **A** and **B** chelation type are shown in Supporting Information S2: Tables S1 and S2). The calculated GlideScore of the new hydroxamates is similar to that of the reference inhibitor vorinostat one for all HDAC receptors considered for all three zinc chelation types. Due to only minor structural variations of the series of hydroxamates located in the capping group (Figure 3), the computed differences in the docking score between the 22 derivatives are relatively small (standard deviation of 0.2–1.5 for the seven HDACs considered). Therefore, it is more convenient to compare mean values of the XP GlideScore averaged over the set of tested hydroxamates rather than the scores of individual compounds when making conclusions about the importance of individual HDAC targets for the antiproliferative effect caused or about modes of binding in the active sites of individual HDACs. A comparison of averaged values of XP GlideScore for the hydroxamic acid derivatives **7a**–**7v** between three types of zinc ion chelation **A**, **B**, and **C** (Figure 8) for the selected HDACs is given in Table 2. It follows from the averaged docking scores in Table 2 that the preferred type of zinc complexation depends significantly on the 3D structure of the binding site of individual HDAC isoforms with a minor preference for bidenate type **C** zinc coordination by oxygens of anionic hydroxamate group with a proton transfer to N_ε_ of His142 (Figure 4). As expected, most of the inhibitors tested **7a**–**7v** displayed predicted binding affinities to HDAC1–HDAC8 comparable or superior to the vorinostat **1** (Table 1).

**Table 1:** XP GlideScore for new hydroxamic acid derivatives **7a–7v** (Figure 3) and reference inhibitor vorinostat **1** in [kcal·mol^−1^] computed for bidenate type **C** chelation of the zinc ion (Figure 8) for selected class I and class II HDACs: HDAC1, 2, 3, 8 and 4, 6, 7.

C ‐ Bidenate binding mode: two oxygens, anionic hydroxamate –C═O–NH–O^(–)^							
HDACs	HDAC1	HDAC2	HDAC3	HDAC4	HDAC6	HDAC7	HDAC8
Ligand	*5ICN*	*7KBG*	*4A69*	*2VQM*	*5EDU*	*3ZNR*	*5VI6*
**Vorinostat 1**	–7.5	–2.9	–6.8	–7.5	–7.3	–10.4	–8.6
**7a**	–8.5	–8.7	–10.3	–6.9	–7.1	–9.0	–9.3
**7b**	–8.3	–5.4	–10.0	–7.0	–6.4	–8.9	–9.1
**7c**	–8.5	–7.6	–10.4	–6.8	–4.0	–7.9	–9.3
**7d**	–8.2	–4.6	–10.6	–7.2	–5.5	–8.9	–9.0
**7e**	–8.3	–8.5	–10.2	–7.1	–6.6	–9.1	–9.3
**7f**	–8.4	–5.4	–10.3	–7.4	–7.3	–9.0	–9.0
**7g**	–8.5	–7.4	–10.4	–6.6	–6.3	–9.0	–8.0
**7h**	–8.3	–5.6	–10.1	–6.8	–6.6	–9.0	–9.4
**7i**	–8.3	–7.1	–10.2	–6.4	–6.3	–8.9	–9.1
**7j**	–8.0	–7.5	–6.1	–6.6	–6.8	–8.9	–9.0
**7k**	–8.4	–5.6	–9.8	–7.0	–7.5	–8.9	–9.3
**7l**	–8.5	–7.6	–10.2	–7.0	–6.4	–8.9	–9.1
**7m**	–8.3	–8.6	–10.2	–6.6	–6.5	–8.5	–8.9
**7n**	–8.3	–5.8	–9.7	–6.9	–7.7	–8.6	–9.3
**7o**	–8.3	–8.4	–9.8	–6.9	–3.8	–8.9	–8.9
**7p**	–7.9	–7.6	–10.3	–6.4	–3.8	–8.6	–8.9
**7q**	–8.2	–3.0	–9.6	–6.6	–3.6	–8.7	–8.5
**7r**	–8.3	–8.4	–10.2	–6.5	–7.7	–8.8	–9.3
**7s**	–8.0	–8.5	–10.2	–6.3	–7.0	–8.8	–8.9
**7t**	–9.0	–5.7	–10.2	–7.2	–6.9	–8.9	–8.7
**7u**	–8.4	–7.5	–10.1	–7.2	–6.6	–8.7	–8.8
**7v**	–8.3	–7.3	–10.3	–6.4	–6.3	–8.8	–8.7
**Average C** a	–8.3 ± 0.2	–6.9 ± 1.5	–10.0 ± 0.9	–6.8 ± 0.3	–6.2 ± 1.2	–8.8 ± 0.3	–9.0 ± 0.3

*Note*: Green shaded rows indicate data for standard and yellow shaded rows indicate data for the most potent compounds studied in detail.

Abbreviation: HDAC, histone deacetylase.

a Average XP GlideScore in [kcal·mol^−1^] ± standard deviation over 22 hydroxamate inhibitors **7a**–**7v.**

**Table 2:** Comparison of averaged XP GlideScore over 22 hydroxamic acid derivatives **7a–7v** (Figure 3) in [kcal·mol^−1^] for three types of zinc ion chelation **A**, **B** and **C** (Figure 8) with vorinostat 1 for selected class I and class II HDACs: HDAC1, 2, 3, 8 and 4, 6, 7.

	**Class I**			**Class II**			
**Binding mode**	**HDAC1**	**HDAC2**	**HDAC3**	**HDAC4**	**HDAC6**	**HDAC7**	**HDAC8**
Average A^a^	–8.7 ± 0.6	–5.4 ± 0.3	–8.3 ± 0.6	–7.7 ± 0.3	–5.5 ± 1.6	–3.5 ± 0.8	–8.4 ± 0.4
Average B	–6.4 ± 0.6	–8.9 ± 0.2	–9.0 ± 0.6	–5.1 ± 0.2	–8.9 ± 0.5	–1.7 ± 1.2	–8.2 ± 0.7
Average C	–8.3 ± 0.2	–6.9 ± 1.5	–10.0 ± 0.9	–6.8 ± 0.3	–6.2 ± 1.2	–8.8 ± 0.3	–9.0 ± 0.3
Vorinostat **1**	–7.5	–2.9	–6.8	–7.5	–7.3	–10.4	–8.6

*Note*: A = Bidentate binding mode: Zn^2+^ coordination by two oxygens, neutral hydroxamate –C**═**O–NH–OH. B = Monodentate binding mode: Zn^2+^ coordination by carbonyl oxygen, neutral hydroxamate –C**═**O–NH–OH. C = Bidenate binding mode: Zn^2+^ coordination by two oxygens, anionic hydroxamate –C**═**O–NH–O(–).

Abbreviation: HDAC, histone deacetylase.

a Average XP GlideScore in [kcal·mol^‐1^] ± standard deviation over 22 hydroxamate inhibitors **7a**–**7v**.

### 2.3 Pharmacology/Biology

#### 2.3.1 Proliferation inhibitory effects of new hydroxamic acids in THP‐1 cell line

To determine whether the tested hydroxamic acid derivatives suppress the proliferation of the human leukaemia monocytic cell line THP‐1 and to compare this activity with the effect of vorinostat **1**, the proliferation of the THP‐1 cell line was evaluated after 24 h of incubation with serial dilutions of hydroxamic acid compounds. The antiproliferative effect was monitored at three incubation intervals to describe the dependence of the antiproliferative activity on time. The results shown in Table 3 revealed that all the compounds demonstrated the ability to effectively inhibit THP‐1 cells. The intensity of proliferation inhibition increases between the time interval of 24 and 48 h. On the contrary, a less significant difference in the intensity of the antiproliferative effect was observed between the incubation times of 48 and 72 h. Even in the case of the reference substance vorinostat **1**, its IC50 value was not determined after 24 h of incubation, because the value was probably higher than the highest concentration tested. However, in the following two incubation times of 48 and 72 h, vorinostat **1** already showed the most potent antiproliferative effect. Only compound **7d** induced proliferation inhibition after 72 h comparable to vorinostat, based on IC50 values. However, all hydroxamic acids exerted antiproliferative activity in THP‐1 cells with single‐digit micromolar IC50 values after 48 and 72 h of incubation. The cytotoxic potential of the compounds tested on the THP‐1 cell line was also evaluated at the same time. However, an LC50 value was not determined for any of the hydroxamic acid derivatives, including vorinostat **1**, so in all cases, it was probably higher than the highest tested concentration of 30 µM (data not shown).

**Table 3:** Effect of hydroxamic acid derivatives on proliferation of THP‐1 cells.

Compound	IC_50_ (μM) ± SD		
	24 h	48 h	72 h
Vorinostat **1**	>30	0.8 ± 0.1	0.7 ± 0.4
**7a**	>30	5.0 ± 1.3	3.9 ± 0.4
**7b**	>30	4.1 ± 0.7	1.8 ± 0.1
**7c**	15.3 ± 0.3	1.9 ± 0.2	1.3 ± 0.1
**7d**	15.1 ± 0.1	1.6 ± 0.2	1.2 ± 0.1
**7e**	21.7 ± 2.0	4.2 ± 1.0	3.6 ± 0.2
**7f**	28.4 ± 2.4	3.3 ± 0.5	4.8 ± 0.4
**7g**	>30	5.6 ± 0.7	3.5 ± 1.0
**7h**	12.9 ± 1.1	3.0 ± 0.4	1.8 ± 0.2
**7i**	12.5 ± 0.5	3.0 ± 0.4	1.6 ± 0.2
**7j**	>30	2.9 ± 0.3	1.7 ± 0.2
**7k**	8.1 ± 0.3	2.9 ± 0.5	3.6 ± 0.6
**7l**	7.0 ± 0.2	3.0 ± 0.9	2.6 ± 0.3
**7m**	>30	3.5 ± 1.5	3.4 ± 0.8
**7n**	11.1 ± 1.4	2.2 ± 0.3	2.8 ± 0.1
**7o**	11.6 ± 1.5	2.7 ± 0.5	2.3 ± 0.3
**7p**	9.4 ± 1.4	2.2 ± 0.4	1.6 ± 0.2
**7q**	9.8 ± 0.2	2.9 ± 0.4	2.5 ± 0.1
**7r**	22.2 ± 1.8	7.5 ± 0.5	6.4 ± 0.5
**7s**	10.1 ± 1.1	2.8 ± 0.2	2.7 ± 0.2
**7t**	>30	5.6 ± 0.4	2.9 ± 0.4
**7u**	>30	2.5 ± 0.3	2.0 ± 0.1
**7v**	>30	9.9 ± 0.5	3.5 ± 0.5

*Note*: Cell proliferation was evaluated using WST‐1 analysis after 24, 48, or 72 h of incubation with serial dilutions of the compounds tested. Values shown are the mean ± SD of three independent experiments, each of which was performed in triplicate.

#### 2.3.2 Class I and II enzyme activity in THP‐1 cells

**Figure 5:**
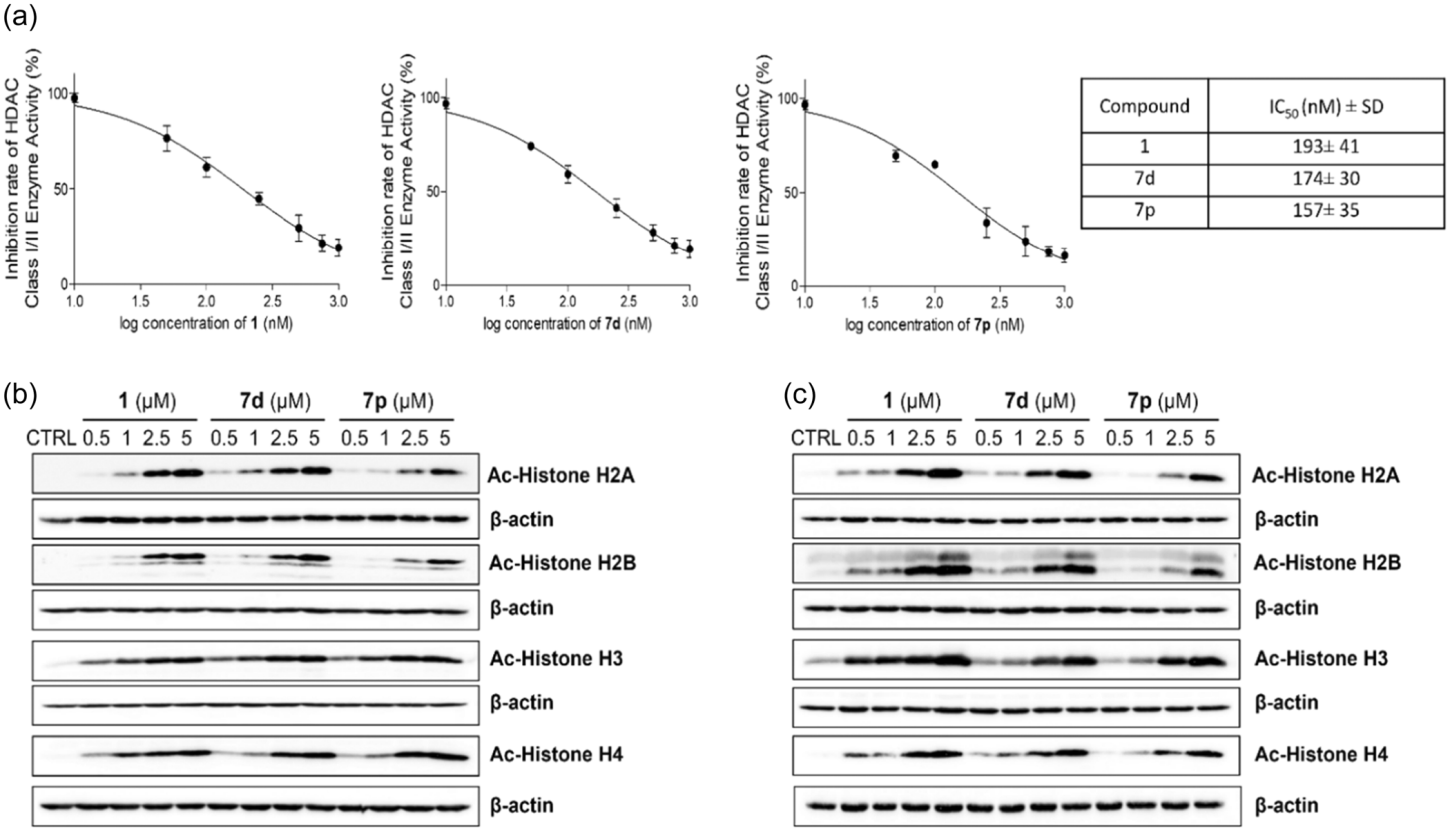
Hydroxamic acid derivatives **7d** and **7p** induce inhibition of histone deacetylase (HDAC) class I and II enzyme activity in THP‐1 cells. (a) The inhibitory effects of vorinostat **1**, **7d**, and **7p** on the enzyme activity of HDAC class I and II were determined using the HDAC‐Glo™ I/II Assay. The results are expressed as the mean ± SD of three independent experiments. The levels of histone H2A, H2B, H3, or H4 acetylation after (b) 24 h or (c) 48 h of treatment with vorinostat **1**, **7d**, and **7p** were determined by immunoblot analysis using appropriate antibodies. Representative immunoblots of one of three experiments are shown. CTRL is a drug‐free control.

The purpose of the following part of the study was to verify the ability of new hydroxamic acid derivatives to inhibit the enzymatic activity of HDAC class I and II (HDAC1 – HDAC10). Based on the previous data demonstrating the antiproliferative activity of new hydroxamic acids in THP‐1 cells, for the assessment of HDAC inhibition, we selected compound **7d**, whose antiproliferative effect after 72 h of incubation was quantitatively comparable to the effect of vorinostat **1**. Additionally, compound **7p** was selected for evaluation as one of the most potent substances among halogen‐substituted hydroxamic acids. As shown in Figure 5a, our results demonstrated that both substances **7p** and **7d**, as well as vorinostat **1**, inhibited HDAC class I and II enzymatic activity. Based on the calculated IC50 values (at nanomolar concentrations), it can be stated that the inhibitory effect of all three tested compounds, **7d**, **7p**, and vorinostat **1**, is quantitatively comparable. Furthermore, we decided to verify the impact of the tested compounds on the acetylation of different types of histones and to evaluate the changes in histone acetylation at two different time points. As demonstrated in Figure 5b,c, tested compounds **7d**, **7p**, and vorinostat **1** increased the histone acetylation of all four histone types, H2A, H2B, H3, and H4, in a concentration‐dependent manner. Furthermore, our results show that this increase in histone acetylation is still present even after 48 h of incubation.

#### 2.3.3 Effects of new hydroxamic acids on cell‐cycle progression

**Figure 6:**
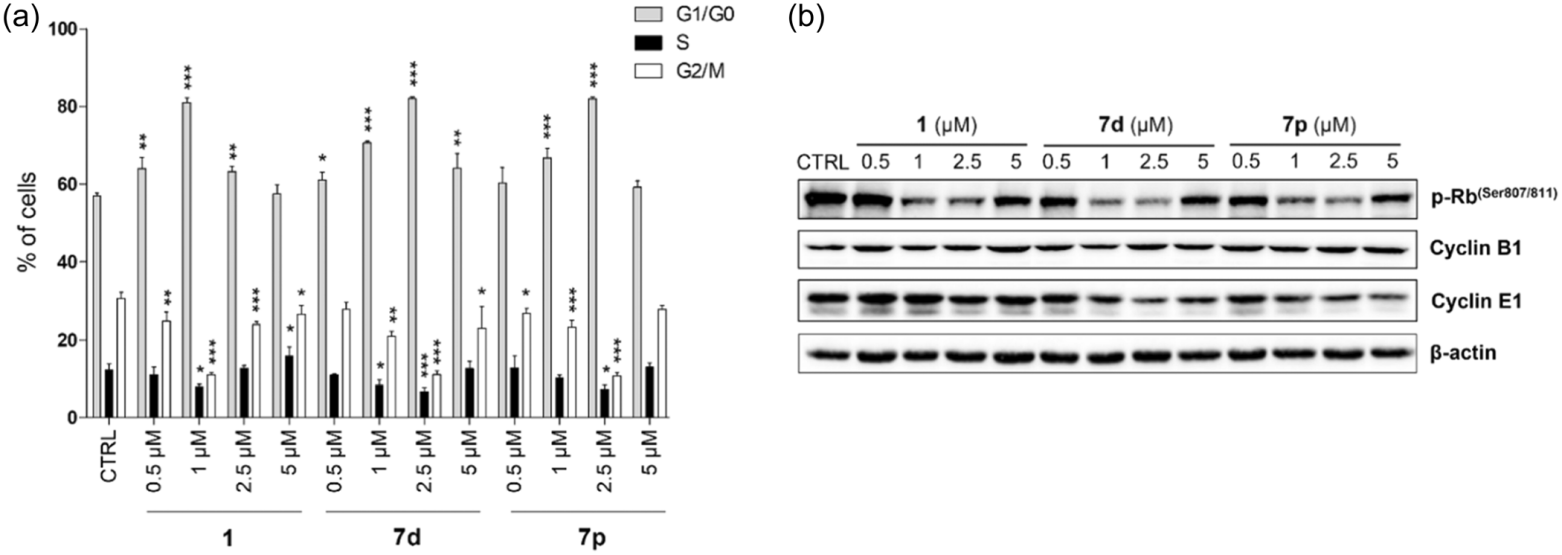
Compounds **7d** and **7p** induce changes in the cell‐cycle progression of THP‐1 cells. THP‐1 cells were treated with indicated concentrations of compounds vorinostat **1**, **7d**, or **7p** for 48 h. (a) The results of the distribution of THP‐1 cells in the cell‐cycle phases are expressed as mean ± SD from three independent experiments. *p < 0.05, **p < 0.01, ***p < 0.001, significantly different from drug‐free control (CTRL). (b) Cell‐cycle regulator levels were determined by immunoblot analysis using appropriate antibodies. Representative immunoblots of one of three experiments are shown. CTRL is a drug‐free control.

Following the previously determined antiproliferative activity of new hydroxamic acids, we decided to further investigate their effect on the cell‐cycle progression of THP‐1 cells. All three tested compounds vorinostat **1**, **7d,** and **7p** induced statistically significant changes in the number of cells in different phases of the cell cycle (Figure 6a). Specifically, the accumulation of cells in the G1 phase of the cell cycle was observed. All the compounds tested increase the number of cells in the G1 phase in a concentration‐dependent manner, in the case of vorinostat **1** up to a concentration of 1 µM, in the case of compounds **7d,** and **7p** up to the concentration of 2.5 µM. On the contrary, higher concentrations of the compounds tested caused less intense changes in the number of cells in cell‐cycle phases. These findings were further supported by immunoblot analysis of the levels of selected cell‐cycle regulators (Figure 6b). In correspondence with the previous results of cell‐cycle progression, all three compounds reduced levels of the phosphorylated form of retinoblastoma protein [p‐Rb (Ser 807/811)] at concentrations that significantly increased the number of cells in the G1 phase. Changes in p‐Rb levels in cells treated with higher concentrations of tested compounds were less intense. Additionally, cyclin E1 levels decreased slightly in a concentration‐dependent manner; this effect was more prominent in the case of **7d** and **7p**. In contrast, no change in cyclin B1 levels was observed.

#### 2.3.4 Compounds **7d** and **7p** induce apoptosis in THP‐1 cells

**Figure 7:**
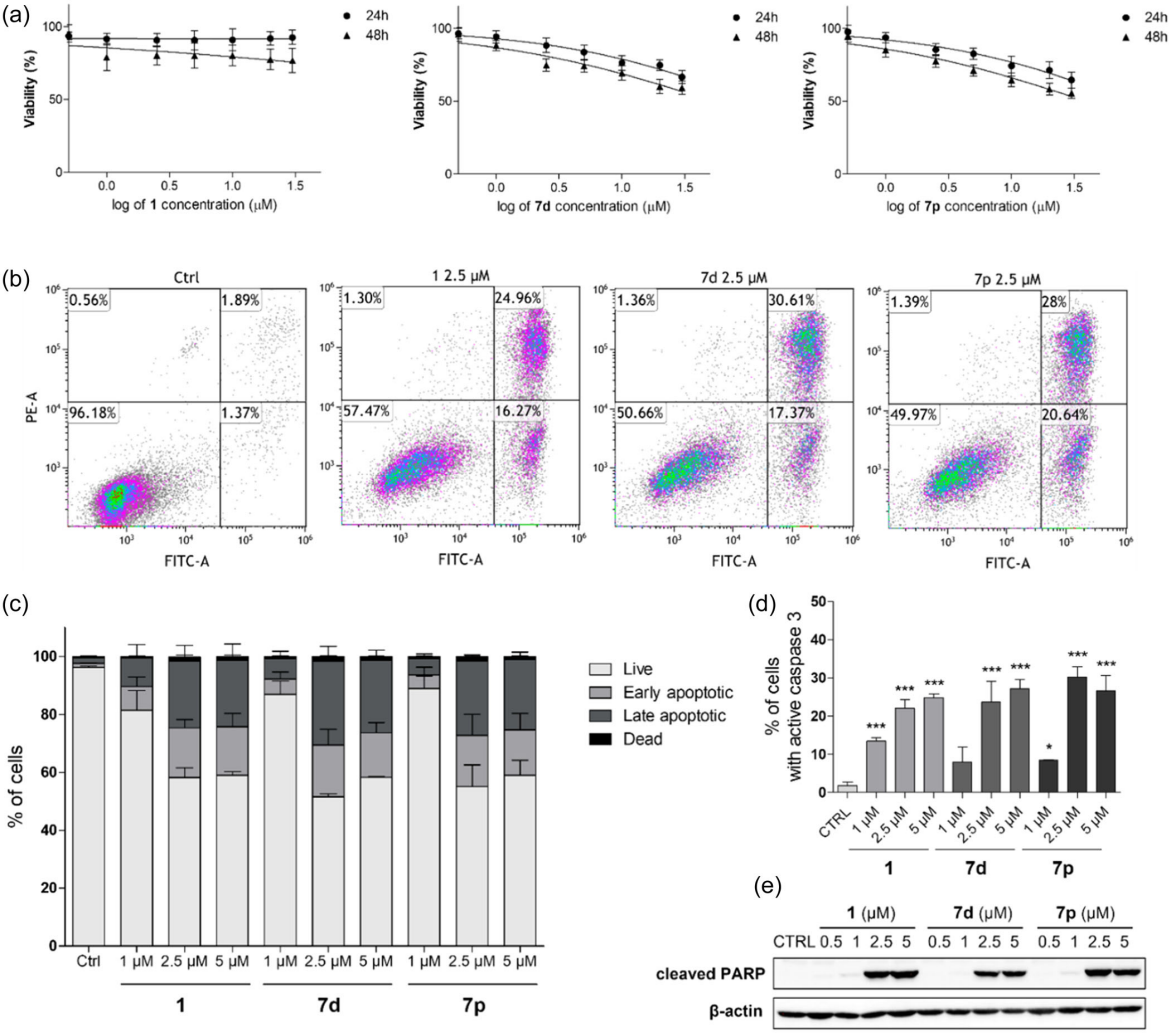
Compounds **7d** and **7p** induce apoptosis in THP‐1 cells. (a) THP‐1 cells were incubated with indicated concentrations and vorinostat **1**, **7d,** or **7p**. Cell viability was determined by LDH assay after 24 and 48 h of incubation. (b, c) THP‐1 cells were treated with compound and vorinostat **1**, **7d,** or **7p**, incubated for 48 h, and analysed by flow cytometry after staining with annexin V‐FITC conjugate and propidium iodide (PI). Here are shown the representative flow cytometry plots of the results of the control sample and the results of samples treated with 2.5 µM of the compounds tested. (d) THP‐1 cells were treated with compounds vorinostat **1**, **7d,** or**7p**, incubated for 48 h, and caspase 3 activation was evaluated by flow cytometry. (e) After 48 h of incubation of THP‐1 cells with and vorinostat **1**, **7d,** or **7p** cleaved poly(ADP‐ribose)polymerase (PARP) levels were detected by immunoblot analysis. The results are shown as the mean ± SD of three independent experiments. *p < 0.05, ***p < 0.001, significantly different from drug‐free control (CTRL). LDF, lactate dehydrogenase.

Although LC_50_ values were not specifically assessed for hydroxamic acids during the cytotoxicity evaluation, these compounds significantly reduce the viability of THP‐1 cells in a manner that depends on both concentration and exposure time (see Figure 7a). Furthermore, since the cell‐cycle analysis additionally revealed the presence of a subdiploid cell population as a marker of cells with fractional DNA content in samples treated by vorinostat **1**, **7d,** or **7p** (Supporting Information S2: Figure S1), we decided to further investigate the proapoptotic effects of the compounds tested. As depicted in Figure 7b,c, flow cytometric analysis demonstrated a noticeable rise in the percentage of both early and late apoptotic cells following treatment with all three tested compounds. Additionally, compounds vorinostat **1**, **7d**, and **7p** were observed to significantly enhance caspase 3 activity (Figure 7d). Finally, we demonstrate the presence of a cleaved form of poly(ADP‐ribose)polymerase (PARP) in samples treated by higher concentrations of the three compounds vorinostat **1**, **7d**, and **7p** (Figure 7e) since PARP is a crucial substrate of caspase 3 in the process of apoptosis.^[^ ^42^ ^]^

## 3 DISCUSSION

The anti‐tumour potential of hydroxamate‐based HDACis remains in the field of the therapy of haematologic malignancies.^[^ ^43^ ^]^ Vorinostat is the first Food and Drug Administration (FDA)‐approved anticancer drug from a group of HDACis that was approved for the therapy of advanced primary cutaneous T‐cell lymphoma.^[^ ^44^ ^]^ Nevertheless, further clinical trials involving vorinostat, either as monotherapy or in combination with other anticancer agents, are warranted for various haematological malignancies. These include conditions like acute myeloid leukaemia (AML). The goal is to fully harness its anti‐tumour capabilities and broaden its range of applications.^[^ ^45^, ^46^, ^47^, ^48^ ^]^

Based on the observations from various studies, haematopoietic malignancies, including aggressive haematopoietic malignancies, such as AML, are more susceptible to epigenetic alterations compared with solid malignancies.^[^ ^49^ ^]^ In preclinical studies, vorinostat demonstrated effects on cell growth and cell‐cycle progression in AML cells. Moreover, it induced apoptosis and triggered the production of reactive oxygen species in these cells.^[^ ^50^, ^51^ ^]^ In the study by Silva et al. THP‐1 cells, in addition to other AML cell lines, were found to be the most sensitive to such activity.^[^ ^52^ ^]^ In our study, our aim was to explore the antiproliferative and proapoptotic effects of a new group of hydroxamic acids in THP‐1 cells and compare their effects with vorinostat **1** activity. The entire group of new hydroxamic acids **7a**–**7v** inhibited THP‐1 cell proliferation with single‐digit micromolar IC_50_ values in longer incubation times of 48 and 72 h, while compound **7d** suppressed proliferation with an effectivity comparable to vorinostat **1** after 72 h of incubation.

Vorinostat, as a pan‐HDAC inhibitor, has an inhibitory effect against a broad spectrum of HDAC isoforms. Vorinostat has been shown to target HDACs 1, 2, 3, and 8 that belong to class I, HDAC6 from class IIb, and even HDAC4 from class IIa.^[^ ^53^ ^]^ Our data showed the ability of selected hydroxamic acids **7d** and **7p**, which were among the derivatives with the highest antiproliferative potential, to inhibit HDAC class I and II in THP‐1 cells at nanomolar concentrations. This effect was quantitatively comparable to that of vorinostat **1**. The inhibition of HDACs by vorinostat **1** was shown to lead to the accumulation of acetylated histones H2a, H2b, H3, and H4.^[^ ^54^ ^]^ We have demonstrated a concentration‐dependent increase in the levels of all acetylated histones H2a, H2b, H3, and H4 after treatment with **7d** or **7p,** similar to the effect of vorinostat **1**. The accumulation of acetylated histones remained stable even after 48 h of exposition to the tested compounds.

It is assumed that histone acetylation, induced by HDACis, which is subsequently followed by restored expression of tumour suppressor genes, is responsible for the antitumour effect of HDACis.^[^ ^55^, ^56^ ^]^ In addition, a hypothesis of the ‘epigenetic vulnerability’ of certain cancer cells was proposed. While normal cells are relatively resistant to the effects of HDACis, because they possess alternative regulatory mechanisms, cancer cells appear to maintain the expression of several key target genes to survive and grow.^[^ ^49^ ^]^ Thus, HDACis can cause growth inhibition, cell‐cycle arrest or cell death, including apoptosis in cancer cells. Vorinostat at 1 µM concentration was shown to induce G1 arrest in THP‐1 cells and decreases the number of cells in phases S and G2/M phases.^[^ ^52^ ^]^ Consistent with those findings, vorinostat **1** showed the same activity in our study. Previously, it was also stated that low concentrations of HDACis can induce cell‐cycle arrest mainly in the G1 cell‐cycle phase, while higher concentrations can affect both G1 and G2/M phases.^[^ ^57^ ^]^ Additionally, Shiozawa et al. described such vorinostat behaviour in HL‐60 human myeloid leukaemia cells, where low vorinostat concentrations of 2.5 µM induce significant accumulation of cells in the G1 phase, while higher concentrations of 10 and 25 µM decrease the number of cells in the G1 phase and accumulate cells in the G2/M phase.^[^ ^58^ ^]^ In our study, it is reasonable to assume that vorinostat **1** impacted cell‐cycle progression in a similar way. Furthermore, higher concentrations of vorinostat **1** would likely influence the distribution of cells across individual phases similarly. The tested compounds **7d** and **7p** also demonstrated a qualitatively similar effect on the cell‐cycle progression of THP‐1 cells.

Cell‐cycle arrest induced by HDACis is most likely mediated through the restoration of expression of various cell‐cycle regulators, including cyclin‐dependent kinase (CDK) inhibitors and cyclins. This leads to an increase of CDK inhibitors and the decrease of cyclin activity and the associated CDK activity causing dephosphorylation of the retinoblastoma protein (Rb), which inhibits E2F role in the expression of genes necessary for cell progression through the G1 phase and subsequent transition to the S phase.^[^ ^59^ ^]^ Consistent with that statement, compounds **7d** and **7p,** including vorinostat **1**, decreased levels of phosphorylated Rb protein at concentrations that induce cell accumulation in phase G1, and mainly **7d** and **7p** slightly decreased levels of cyclin E1 connected with G1/S transition.^[^ ^60^ ^]^

HDAC is have been shown to induce apoptosis through regulation of pro‐ and anti‐apoptotic gene expression and could activate both intrinsic and extrinsic apoptotic pathways.^[^ ^61^ ^]^ Silva et al. described that THP‐1 cells are prone to the proapoptotic activity of vorinostat.^[^ ^52^ ^]^ Our results proved these findings and showed the same activity of our tested compounds. Both **7d** and **7p** were able to increase the number of cells in the early and late stages of apoptosis, caspase 3 activation, and the presence of a cleaved form of PARP, in all cases in a concentration‐dependent manner.

### 3.1 Virtual screening of hydroxamate inhibitors

**Figure 8:**
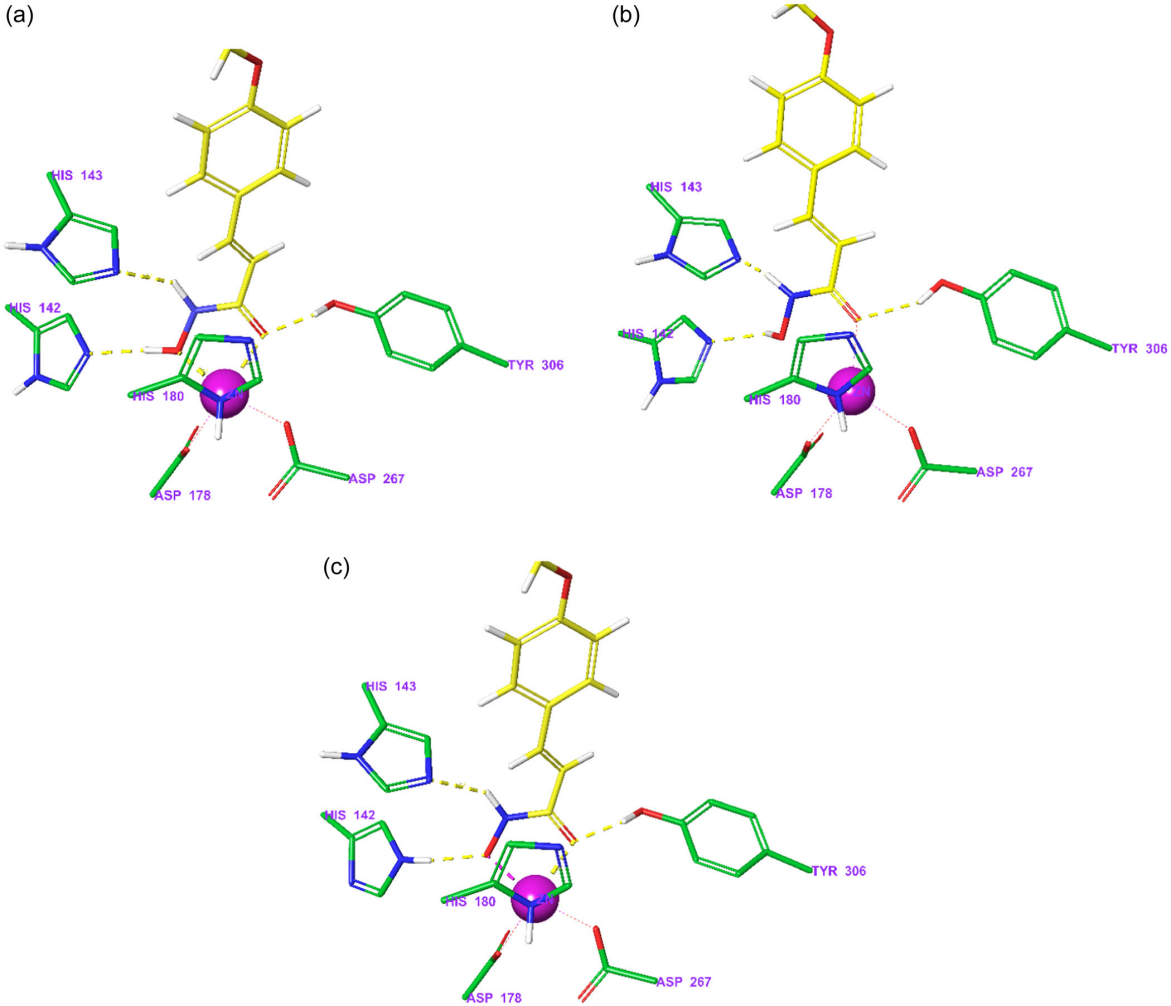
Three types of chelation of the Zn^2+^ ion by hydroxamate group of HDACi **7d**. Type A – bidenate neutral, type B – monodenate neutral and type C – bidenate anionic hydroxamate group. Imidazole N_δ_ of His180 coordinates the zinc ion from above.

Molecular docking and estimated binding affinities to HDAC1—HDAC8 suggest that the new hydroxamic acid derivatives show potential for inhibition of deacetylases comparable to that of the reference inhibitor vorinostat. Except for HDAC7, the averaged predicted binding affinity for at least one chelation type (Figure 8) exceeds the XP GlideScore of vorinostat **1** (Table 2). The computed docking score (Table 2, S1, S2) does not allow one to draw conclusions about the detailed mechanism of the zinc ion coordination by the hydroxamate group. More significant differences in the averaged docking score for the type **A**, **B**, and **C** zinc chelation were found only for the HDAC2, HDAC6, and HDAC7 (Table 2). However, it is likely that in class I HDACs chelation of type **C** could be preferred (Table 2). The highest XP GlideScore for the probable type **C** coordination (Table 1) shows that the hydroxamic acid derivatives display the predicted highest binding affinity to class I deacetylase HDAC3. However, no correlation could be established between the calculated XP GlideScore for HDAC3 and the observed IC_50_ after 72 h in THP‐1 cells. This fact suggests that the overall antiproliferative effect of the tested hydroxamates can hardly be attributed to inhibition of a single specific HDAC isoform but rather to a combined effect on the set of present HDAC variants.

## 4 CONCLUSION

In our study, we showed that a newly synthesized group of 22 hydroxamic acid derivatives can effectively inhibit cell growth in THP‐1 monocytic leukaemia cells and can affect cell‐cycle progression and induce apoptosis in THP‐1 cells similarly to vorinostat, HDACi anticancer agent.

## 5 EXPERIMENTAL

### 5.1 Chemistry

#### 5.1.1 General

The reagents and solvents utilized for synthesis were procured from Sigma‐Aldrich and employed as received unless otherwise specified. Anhydrous reagents and solvents were absolutized as usual and distilled before use. ^1^H‐NMR spectra were obtained on a JEOL ECZR‐400 MHz instrument (Jeol Corp.). All NMR measurements were done at 25°C. Chemical shifts δ are reported in parts per million (ppm) and *J* values in Hz. NMR spectra were acquired in dimethyl sulfoxide (DMSO)‐*d*_6._ The residual solvent signal of DMSO‐*d*_6_ was used for reference. Norell StandartSeries^TM^ 5 mm NMR tubes were utilized. MS analysis was performed on an Impact II (Q‐TOF, Bruker Daltonics) or LTQ Orbitrap XL high‐resolution mass spectrometer (Thermo Fisher Scientific). Melting points were measured using a Böetius apparatus (Franz Küstner Nachf. KG) and were not corrected. Mass spectra were obtained using electrospray ionization (ESI) in the positive or negative ion mode. Merck Kieselgel 60 silica gel (70–230 mesh particle size) was used for column chromatography. All fractions were monitored by silica gel thin‐layer chromatography (TLC) plates (225 μm thickness, 60 A silica gel medium) with the detection of compounds with UV light. A Dionex Ultimate 3000 (Thermo Scientific) HPLC system controlled with the Chromeleon® Chromatography Data System (version 7.2, Thermo Scientific) was used to analyse the final compounds’ purity. The separation was performed on a YMC‐Triart C18 (3 μm, 150 mm × 2 mm) column (Agilent Technologies). The mobile phase consisted of a mixture of acetonitrile and water with 0.1% formic acid in a ratio from 40:60 to 90:10. The total flow rate was set at 0.2 mL/min; the injection volume was 1 μL; and the column temperature was maintained at 30°C. The detection wavelength of 210 nm was chosen. The purity of each compound was determined by calculating the average of relative peak areas in the sample solution chromatograms.

The InChI codes of the investigated compounds, together with some biological activity data, are provided as Supporting Information.

#### 5.1.2 General procedure for the synthesis of compounds **7a–7v** (hydroxamic acids)

Prepared according to the modified literature procedure.^[^ ^62^, ^63^ ^]^

To a three‐necked flask with dissolved hydroxylamine hydrochloride (0.22 g, 3.17 mmol) in 5 mL of anhydrous MeOH was added 1 mg of phenolphthalein. A freshly prepared solution of sodium metal (1.21 g, 5.28 mmol) in 13 mL anhydrous MeOH was added via syringe to the reaction mixture to reach a pink endpoint. A precipitate of NaCl appeared in the flask. After 30 min of stirring a mixture of the corresponding methyl ester **9a–9v** (1.32 mmol) in MeOH and dry cosolvent (THF/DMSO) was added via syringe dropwise to the flask. The remainder of the prepared solution of sodium methanolate was added to the vigorously stirred mixture. This was left to stir under argon at the ambient temperature for 24 h. The reaction mixture was poured into water (20 mL) and acidified with glacial acetic acid with stirring to reach pH=4. The resulting solid was filtered off and washed successively with cold water to remove acetic acid. The crude product was purified by column chromatography (SiO_2_, 1−15% MeOH/dichloromethane (DCM) + 0.1% acetic acid). The final product was dried in vacuo at 40°C giving the final product of hydroxamic acid.

(2*E*)‐*N*‐Hydroxy‐3‐{4‐[(phenylcarbamoyl)methoxy]phenyl}prop‐2‐enamide (**7a**)^[^ ^64^ ^]^: Yellow solid, mp 135−138°C (156 mg, 31%); ^1^H NMR (400 MHz, DMSO‐*d*_*6*_) *δ* 10.68 (br. s., 1H), 10.11 (s, 1H), 8.98 (br. s., 1H), 7.63 (d, *J* = 7.8 Hz, 2H), 7.53 (d, *J* = 8.5 Hz, 2H), 7.41 (d, *J* = 15.8 Hz, 1H), 7.32 (t, *J* = 7.9 Hz, 2H), 7.08 (t, *J* = 7.4 Hz, 1H), 7.03 (d, *J* = 8.7 Hz, 2H), 6.33 (d, *J* = 15.8 Hz, 1H), 4.74 (s, 2H);^13^C NMR (101 MHz, DMSO‐*d*_*6*_ *) δ* 166.3, 163.1, 158.9, 138.4, 137.9, 129.0, 128.8, 128.0, 123.7, 119.7, 116.9, 115.1, 67.1; HRMS *m/z* [M‐H]^‐^ calcd for C_17_H_15_N_2_O_4_: 311.1110; found: 311.1034.

(2*E*)‐*N*‐Hydroxy‐3‐(4‐{[(2‐methylphenyl)carbamoyl]methoxy}phenyl)prop‐2‐enamide (**7b**): Pale brown solid, mp 138−140°C (280 mg, 55%); ^1^H NMR (400 MHz, DMSO‐*d*_*6*_) *δ* 9.52 (br. s., 1H), 7.31−7.68 (m, 4H), 6.95−7.30 (m, 5H), 6.35 (d, *J* = 11.0 Hz, 1H), 4.78 (br. s., 2H), 2.17 (br. s., 3H); ^13^C NMR (101 MHz, DMSO‐*d*_*6*_) *δ* 166.4, 163.0, 158.7, 137.8, 135.5, 132.2, 130.3, 129.0, 128.1, 126.0, 125.7, 125.3, 117.0, 115.2, 67.0, 17.7; HRMS *m/z* [M‐H]^‐^ calcd for C_18_H_17_N_2_O_4_: 325.1183; found: 325.1174.

(2*E*)‐*N*‐Hydroxy‐3‐(4‐{[(3‐methylphenyl)carbamoyl]methoxy}phenyl)prop‐2‐enamide (**7c**): Pale brown solid, mp 136−138°C (271 mg, 54%); ^1^H NMR (400 MHz, DMSO‐*d*_*6*_) *δ* 10.71 (br. s., 1H), 10.04 (br. s., 1H), 9.01 (br. s., 1H), 7.35−7.61 (m, 5H), 7.20 (t, *J* = 7.1 Hz, 1H), 7.03 (d, *J* = 6.9 Hz, 2H), 6.90 (d, *J* = 7.0 Hz, 1H), 6.34 (d, *J* = 15.2 Hz, 1H), 4.74 (br. s., 2H), 2.27 (s, 3H); ^13^C NMR (101 MHz, DMSO‐*d*_*6*_) *δ* 166.2, 163.2, 158.9, 138.3, 138.0, 129.1, 128.6, 128.1, 124.5, 120.2, 116.9, 115.1, 67.1, 21.2; HRMS *m/z* [M‐H]^‐^ calcd for C_18_H_17_N_2_O_4_: 325.1183; found: 325.1179.

(2*E*)‐*N*‐Hydroxy‐3‐(4‐{[(4‐methylphenyl)carbamoyl]methoxy}phenyl)prop‐2‐enamide (**7d**): Pale brown solid, mp 146−148°C (286 mg, 57%); ^1^H NMR (400 MHz, DMSO‐*d*_*6*_) *δ* 10.31−11.00 (m, 1H), 10.03 (br. s., 1H), 8.67−9.33 (m, 1H), 7.52 (br. s., 4H), 7.41 (d, *J* = 15.1 Hz, 1H), 6.97−7.18 (m, 4H), 6.34 (d, *J* = 14.4 Hz, 1H), 4.73 (br. s., 2H), 2.25 (s, 3H); ^13^C NMR (101 MHz, DMSO‐*d*_*6*_) *δ* 166.0, 163.1, 158.9, 137.9, 135.8, 132.7, 129.1, 129.0, 128.0, 119.7, 116.9, 115.1, 67.1, 20.5; HRMS *m/z* [M‐H]^‐^ calcd for C_18_H_17_N_2_O_4_: 325.1183; found: 325.1179.

(2*E*)‐*N*‐Hydroxy‐3‐(4‐{[(2‐methoxyphenyl)cabamoyl]‐methoxy}phenyl)prop‐2‐enamide (**7e**): Yellow solid, mp 134−137°C (271 mg, 54%); ^1^H NMR (400 MHz, DMSO‐*d*_*6*_) *δ* 10.71 (br. s., 1H), 9.24 (s, 1H), 9.00 (br. s., 1H), 8.05 (d, *J* = 7.8 Hz, 1H), 7.55 (d, *J* = 8.5 Hz, 2H), 7.42 (d, *J* = 15.8 Hz, 1H), 7.02−7.15 (m, 4H), 6.89−6.96 (m, 1H), 6.35 (d, *J* = 15.8 Hz, 1H), 4.81 (s, 2H), 3.85 (s, 3H); ^13^C NMR (101 MHz, DMSO‐*d*_*6*_) *δ* 166.1, 163.1, 158.5, 149.1, 137.9, 129.1, 128.3, 126.5, 124.7, 120.9, 120.5, 117.1, 115.2, 111.2, 67.1, 55.9; HRMS *m/z* [M‐H]^‐^ calcd for C_18_H_17_N_2_O_5_: 341.1216; found: 341.1140.

(2*E*)‐*N*‐Hydroxy‐3‐(4‐{[(3‐methoxyphenyl)carbamoyl]‐methoxy}phenyl)prop‐2‐enamide (**7f**): Pale brown solid, mp 130−132°C (241 mg, 48%);^1^H NMR (400 MHz, DMSO‐*d*_*6*_) *δ* 10.72 (br. s., 1H), 10.12 (br. s., 1H), 9.02 (br. s., 1H), 7.53 (d, *J* = 5.7 Hz, 2H), 7.28−7.47 (m, 2H), 7.21 (br. s., 2H), 7.03 (d, *J* = 6.0 Hz, 2H), 6.66 (d, *J* = 5.5 Hz, 1H), 6.34 (d, *J* = 14.9 Hz, 1H), 4.75 (br. s., 2H), 3.72 (s, 3H); ^13^C NMR (101 MHz, DMSO‐*d*_*6*_) *δ* 166.1,4 163.2, 159.5, 158.9, 139.6, 137.9, 129.6, 129.1, 128.1, 116.9, 115.1, 111.9, 109.2, 105.4, 67.1, 55.0; HRMS *m/z* [M‐H]^‐^ calcd for C_18_H_17_N_2_O_5_: 341.1216; found: 341.1138.

(2*E*)‐*N*‐Hydroxy‐3‐(4‐{[(4‐methoxyphenyl)cabamoyl]‐methoxy}phenyl)prop‐2‐enamide (**7g**): Yellowish solid, mp 169−171°C (261 mg, 52%); ^1^H NMR (400 MHz, DMSO‐*d*_*6*_) *δ* 9.97−10.61 (m, 1H), 7.57 (d, *J* = 8.8 Hz, 2H), 7.35 (d, *J* = 8.4 Hz, 2H), 6.91 (dd, *J* = 9.1, 15.3 Hz, 5H), 6.26 (d, *J* = 15.8 Hz, 1H), 4.66 (s, 2H), 3.72 (s, 3H); ^13^C NMR (101 MHz, DMSO‐*d*_*6*_) *δ* 166.1, 164.6, 157.0, 155.5, 131.6, 130.9, 127.9, 127.4, 124.9, 121.3, 114.9, 113.8, 67.2, 55.2; HRMS *m/z* [M‐H]^‐^ calcd for C_18_H_17_N_2_O_5_: 341.1216; found: 341.1140.

(2*E*)‐3‐(4‐{[(2‐Fluorophenyl)carbamoyl]methoxy}phenyl)‐*N*‐hydroxyprop‐2‐enamide (**7h**): Brown solid, mp 129−131°C (160 mg, 32%); ^1^H NMR (400 MHz, DMSO‐*d*_*6*_) *δ* 10.68 (br. s., 1H), 9.93 (br. s., 1H), 9.01 (br. s., 1H), 7.76−7.87 (m, 1H), 7.53 (d, *J* = 7.9 Hz, 2H), 7.41 (d, *J* = 15.7 Hz, 1H), 7.14−7.33 (m, 3H), 7.02 (d, *J* = 8.0 Hz, 2H), 6.33 (d, *J* = 15.7 Hz, 1H), 4.82 (br. s., 2H); ^19^F NMR (376 MHz, DMSO‐*d*_*6*_) *δ* ‐123.99; ^13^C NMR (101 MHz, DMSO‐*d*_*6*_) *δ* 166.7, 163.1, 158.8, 154.2 (d, *J* = 254.6 Hz, Cq), 137.9, 129.1, 128.1, 126.1 (d, *J* = 7.7 Hz, CH), 125.4 (d, *J* = 10.6 Hz, Cq), 124.8, 124.5, 116.9, 115.6 (d, *J* = 19.3 Hz, CH), 115.1, 66.8; HRMS *m/z* [M+Na]^+^ calcd for C_17_H_15_FN_2_NaO_4_: 353.0908; found: 353.0909.

(2*E*)‐3‐(4‐{[(3‐Fluorophenyl)carbamoyl]methoxy}phenyl)‐*N*‐hydroxyprop‐2‐enamide (**7i**): Brown solid, mp 140−142°C (176 mg, 35%); ^1^H NMR (400 MHz, DMSO‐*d*_*6*_) *δ* 10.69 (s, 1H), 10.33 (s, 1H), 8.99 (s, 1H), 7.62 (d, *J* = 11.7 Hz, 1H), 7.53 (d, *J* = 8.6 Hz, 2H), 7.29−7.47 (m, 3H), 7.03 (d, *J* = 8.7 Hz, 2H), 6.87−6.96 (m, 1H), 6.32 (d, *J* = 15.8 Hz, 1H), 4.76 (s, 2H); ^19^F NMR (376 MHz, DMSO‐*d*_*6*_) *δ* ‐111.91; ^13^C NMR (101 MHz, DMSO‐*d*_*6*_) *δ* 166.7, 163.1, 162.1 (d, *J* = 240.8 Hz, Cq), 158.8, 140.1 (d, *J* = 11.6 Hz, Cq), 137.9, 130.5 (d, *J* = 8.6 Hz, CH), 129.1, 128.1, 116.9, 115.4 (d, *J* = 2.9 Hz, CH), 115.1, 110.2 (d, *J* = 21.2 Hz, CH), 106.4 (d, *J* = 26.0 Hz, CH), 67.0; HRMS *m/z* [M‐H]^‐^ calcd for C_17_H_14_FN_2_O_4_: 329.0932; found: 329.0930.

(2*E*)‐3‐(4‐{[(4‐Fluorophenyl)carbamoyl]methoxy}phenyl)‐*N*‐hydroxyprop‐2‐enamide (**7j**)^[^ ^64^ ^]^: Pale brown solid, mp 138−140°C (105 mg, 21%); ^1^H NMR (400 MHz, DMSO‐*d*_*6*_) *δ* 12.26 (s, 1H), 10.18 (s, 1H), 7.59−7.75 (m, 4H), 7.54 (d, *J* = 16.0 Hz, 1H), 7.17 (t, *J* = 8.8 Hz, 2H), 7.03 (d, *J* = 8.5 Hz, 2H), 6.40 (d, *J* = 15.9 Hz, 1H), 4.75 (s, 2H); ^19^F NMR (376 MHz, DMSO‐*d*_*6*_) *δ* –118.68; ^13^C NMR (101 MHz, DMSO‐*d*_*6*_) *δ* 166.3, 163.0, 158.8, 158.3 (d, *J* = 240.8 Hz, Cq), 137.8, 134.8, 129.0, 128.1, 121.6 (d, *J* = 7.7 Hz, CH), 116.9, 115.3 (d, *J* = 22.2 Hz, CH), 115.1, 67.1; HRMS *m/z* [M‐H]^‐^ calcd for C_17_H_14_FN_2_O_4_: 329.0932; found: 329.0936.

(2*E*)‐3‐(4‐{[(2‐Chlorophenyl)carbamoyl]methoxy}phenyl)‐*N*‐hydroxyprop‐2‐enamide (**7k**): Brown solid, mp 144−147°C (215 mg, 43%); ^1^H NMR (400 MHz, DMSO‐*d*_*6*_) *δ* 10.69 (br. s., 1H), 9.68 (br. s., 1H), 9.00 (br. s., 1H), 7.81 (d, *J* = 7.3 Hz, 1H), 7.54 (br. s., 3H), 7.31−7.47 (m, 2H), 7.23 (br. s., 1H), 7.06 (d, *J* = 6.63 Hz, 2H), 6.34 (d, *J* = 14.4 Hz, 1H), 4.83 (br. s., 2H); ^13^C NMR (101 MHz, DMSO‐*d*_*6*_) *δ* 166.7, 163.0, 158.5, 137.1, 134.2, 129.6, 129.1, 128.4, 127.7, 126.7, 126.5, 117.2, 115.2, 67.0; HRMS *m/z* [M‐H]^‐^ calcd for C_17_H_14_^35^ClN_2_O_4_: 345.0720/C_17_H_14_^37^ClN_2_O_4_: 347.0691; found: 345.0646.

(2*E*)‐3‐(4‐{[(3‐Chlorophenyl)carbamoyl]methoxy}phenyl)‐*N*‐hydroxyprop‐2‐enamide (**7l**): Yellowish solid, mp 157−159°C (210 mg, 42%); ^1^H NMR (400 MHz, DMSO‐*d*_*6*_) *δ* 10.71 (br. s., 1H), 10.31 (br. s., 1H), 8.90−9.14 (m, 1H), 7.83 (br. s., 1H), 7.53 (d, *J* = 7.9 Hz, 4H), 7.41 (d, *J* = 15.9 Hz, 1H), 7.35 (t, *J* = 8.1 Hz, 1H), 7.14 (d, *J* = 7.6 Hz, 1H), 7.03 (d, *J* = 8.1 Hz, 2H), 6.33 (d, *J* = 15.7 Hz, 1H), 4.76 (s, 2H); ^13^C NMR (101 MHz, DMSO‐*d*_*6*_) *δ* 166.8, 163.2, 158.8, 139.8, 138.0, 133.1, 130.6, 129.1, 128.2, 123.5, 119.2, 118.1, 115.2, 67.1; HRMS *m/z* [M‐H]^‐^ calcd for C_17_H_14_^35^ClN_2_O_4_: 345.0720/C_17_H_14_^37^ClN_2_O_4_: 347.0691; found: 345.0649.

(2*E*)‐3‐(4‐{[(4‐Chlorophenyl)carbamoyl]methoxy}phenyl)‐*N*‐hydroxyprop‐2‐enamide (7m): Beige solid, mp 145−146°C (230 mg, 46%); ^1^H NMR (400 MHz, DMSO‐*d*_*6*_) *δ* 10.31 (s, 1H), 7.68 (d, *J* = 8.8 Hz, 2H), 7.53 (d, *J* = 8.5 Hz, 2H), 7.34−7.45 (m, 3H), 7.03 (d, *J* = 8.6 Hz, 2H), 6.34 (d, *J* = 15.8 Hz, 1H), 4.76 (s, 2H); ^13^C NMR (101 MHz, DMSO‐*d*_*6*_) *δ* 166.5, 163.0, 158.8, 137.8, 137.4, 129.0, 128.7, 128.1, 127.3, 121.3, 117.0, 115.1, 67.1; HRMS *m/z* [M‐H]^‐^ calcd for C_17_H_14_^35^ClN_2_O_4_: 345.0720/C_17_H_14_^37^ClN_2_O_4_: 347.0691; found: 345.0647.

(2*E*)‐3‐(4‐{[(2‐Bromophenyl)carbamoyl]methoxy}phenyl)‐*N*‐hydroxyprop‐2‐enamide (**7n**): Yellow solid, mp 144−146°C (120 mg, 24%); ^1^H NMR (400 MHz, DMSO‐*d*_*6*_) *δ* 12.10−12.43 (br. s., 1H), 9.63 (s, 1H), 7.79 (d, *J* = 7.9 Hz, 1H), 7.68 (d, *J* = 8.1 Hz, 3H), 7.55 (d, *J* = 15.9 Hz, 1H), 7.41 (t, *J* = 7.7 Hz, 1H), 7.16 (t, *J* = 7.7 Hz, 1H), 7.08 (d, *J* = 8.4 Hz, 2H), 6.41 (d, *J* = 16.0 Hz, 1H), 4.83 (s, 2H); ^13^C NMR (101 MHz, DMSO‐*d*_*6*_) d 166.6, 163.0, 158.4, 137.8, 135.4, 132.7, 129.1, 128.34, 128.30, 127.1, 125.7, 117.2, 117.0, 115.2, 67.0; HRMS *m/z* [M‐H]^‐^ calcd for C_17_H_14_^79^BrN_2_O_4_: 389.0215/C_17_H_14_^81^BrN_2_O_4_: 391.0195; found: 389.0129.

(2*E*)‐3‐(4‐{[(3‐Bromophenyl)carbamoyl]methoxy}phenyl)‐*N*‐hydroxyprop‐2‐enamide (**7o**): Beige solid, mp 136−139°C (111 mg, 22%); ^1^H NMR (400 MHz, DMSO‐*d*_*6*_) *δ* 10.31 (br. s., 1H), 7.98 (s, 1H), 7.47−7.67 (m, 3H), 7.40 (d, *J* = 15.8 Hz, 1H), 7.24−7.34 (m, 2H), 7.03 (d, *J* = 8.5 Hz, 2H), 6.33 (d, *J* = 15.8 Hz, 1H), 4.76 (s, 2H); ^13^C NMR (101 MHz, DMSO‐*d*_*6*_) *δ* 166.7, 163.0, 158.7, 139.9, 137.8, 130.7, 129.0, 128.1, 126.3, 122.0, 121.5, 118.4, 116.9, 115.1, 67.0; HRMS *m/z* [M‐H]^‐^ calcd C_17_H_14_^79^BrN_2_O_4_: 389.0215/C_17_H_14_^81^BrN_2_O_4_: 391.0195; found: 389.0128.

(2*E*)‐3‐(4‐{[(4‐Bromophenyl)carbamoyl]methoxy}phenyl)‐*N*‐hydroxyprop‐2‐enamide (**7p**): Beige solid, mp 148−150°C (141 mg, 28%); ^1^H NMR (400 MHz, DMSO‐*d*_*6*_) *δ* 10.70 (br. s., 1H), 10.27 (br. s., 1H), 9.00 (br. s., 1H), 7.45−7.79 (m, 6H), 7.41 (d, *J* = 14.9 Hz, 1H), 7.03 (br. s., 2H), 6.33 (d, *J* = 13.8 Hz, 1H), 4.75 (br. s., 2H); ^13^C NMR (101 MHz, DMSO‐*d*_*6*_) *δ* 166.5, 163.1, 158.8, 137.9, 137.7, 131.6, 129.0, 128.1, 121.6, 116.9, 115.4, 115.1, 67.1; HRMS *m/z* [M‐H]^‐^ calcd for C_17_H_14_^79^BrN_2_O_4_: 389.0215/C_17_H_14_^81^BrN_2_O_4_: 391.0195; found: 389.0123.

(2*E*)‐*N*‐Hydroxy‐3‐[4‐({[2‐(trifluoromethyl)phenyl]‐cabamoyl}methoxy)phenyl]prop‐2‐enamide (7q): Light red solid, mp 140−142°C (230 mg, 46%); ^1^H NMR (400 MHz, DMSO‐*d*_*6*_) *δ* 10.48 (br. s., 1H), 7.82 (d, *J* = 8.5 Hz, 2H), 7.66 (d, *J* = 8.6 Hz, 2H), 7.49 (d, *J* = 8.5 Hz, 2H), 7.30−7.40 (m, 1H), 6.99 (d, *J* = 8.6 Hz, 2H), 6.29 (d, *J* = 15.8 Hz, 1H), 4.76 (s, 2H); ^19^F NMR (376 MHz, DMSO‐*d*_*6*_) *δ* –60.29; ^13^C NMR (101 MHz, DMSO‐*d*_*6*_) *δ* 168.2, 167.0, 159.2, 142.7, 142.0, 129.8, 127.8, 126.1 (q, *J* = 3.9 Hz, CH), 124.4 (q, *J* = 271.7 Hz, CF_3_), 123.7 (q, *J* = 31.8 Hz, C_q_), 119.6, 118.2, 115.1, 67.0; HRMS *m/z* [M‐H]^‐^ calcd for C_18_H_14_F_3_N_2_O_4_: 379.0900; found: 379.0899.

(2*E*)‐*N*‐Hydroxy‐3‐[4‐({[3‐(trifluoromethyl)phenyl]carbamoyl}methoxy)phenyl]prop‐2‐enamide (**7r**): Pale brown solid, mp 133−135°C (186 mg, 37%); ^1^H NMR (400 MHz, DMSO‐*d*_*6*_) *δ* 12.03−12.53 (br. s., 1H), 9.76 (s, 1H), 7.77 (d, *J* = 7.8 Hz, 1H), 7.62−7.74 (m, 4H), 7.55 (d, *J* = 15.9 Hz, 1H), 7.44−7.51 (m, 1H), 7.03 (d, *J* = 8.6 Hz, 2H), 6.41 (d, *J* = 16.0 Hz, 1H), 4.81 (s, 2H); ^19^F NMR (376 MHz, DMSO‐*d*_*6*_) *δ* ‐59.24; ^13^C NMR (101 MHz, DMSO‐*d*_*6*_) *δ* 167.4, 163.0, 158.5, 137.8, 134.7, 133.3, 129.1, 128.3, 126.9, 126.4 (q, *J* = 4.8 Hz, CH), 124.2 (q, *J* = 29.9 Hz, C_q_), 123.6 (q, *J* = 273.6 Hz, CF_3_),117.0, 115.1, 66.8; HRMS *m/z* [M+Na]^+^ calcd for C_18_H_15_F_3_N_2_NaO_4_: 403.0876; found: 403.0879.

(2*E*)‐*N*‐Hydroxy‐3‐[4‐({[4‐(trifluoromethyl)phenyl]carbamoyl}methoxy)phenyl]prop‐2‐enamide (**7s**): Beige solid, mp 155−157°C (249 mg, 50%); ^1^H NMR (400 MHz, DMSO‐*d*_*6*_) *δ* 12.06−12.44 (br. s., 1H), 10.47 (s, 1H), 7.82 (d, *J* = 8.5 Hz, 2H), 7.64 (dd, *J* = 8.7, 15.9 Hz, 4H), 7.50 (d, *J* = 16.0 Hz, 1H), 6.99 (d, *J* = 8.7 Hz, 2H), 6.36 (d, *J* = 16.0 Hz, 1H), 4.78 (s, 2H); ^19^F NMR (376 MHz, DMSO‐*d*_*6*_) *δ* ‐60.26; ^13^C NMR (101 MHz, DMSO‐*d*_*6*_) *δ* 167.9, 167.0, 159.4, 143.5, 142.0, 129.9, 127.6, 126.2 (q, *J* = 3.9 Hz, CH), 124.4 (q, *J* = 271.7 Hz, CF_3_), 123.7 (q, *J* = 31.8 Hz, C_q_), 119.5, 117.1, 115.1, 67.0; HRMS *m/z* [M‐H]^‐^ calcd for C_18_H_14_F_3_N_2_O_4_: 379.0900; found: 379.0900.

(2*E*)‐*N*‐Hydroxy‐3‐(4‐{[(2‐nitrophenyl)carbamoyl]methoxy}phenyl)prop‐2‐enamide (**7t**): Pale brown solid, mp 153−155°C (89 mg, 18%); ^1^H NMR (400 MHz, DMSO‐*d*_*6*_) *δ* 12.30 (br. s., 1H), 10.12 (s, 1H), 7.65 (t, *J* = 9.3 Hz, 3H), 7.55 (d, *J* = 15.9 Hz, 1H), 7.32 (t, *J* = 7.7 Hz, 2H), 6.98−7.12 (m, 3H), 6.40 (d, *J* = 15.9 Hz, 1H), 4.75 (s, 2H); ^13^C NMR (101 MHz, DMSO‐*d*_*6*_) *δ* 167.8, 166.3, 159.5, 143.6, 138.4, 129.9, 128.8, 127.5, 123.8, 119.7, 116.9, 115.1, 67.1; HRMS *m/z* [M‐H]^‐^ calcd for C_17_H_14_N_3_O_6_: 356.0877; found: 356.0895.

(2*E*)‐*N*‐Hydroxy‐3‐(4‐{[(3‐nitrophenyl)carbamoyl]methoxy}phenyl)prop‐2‐enamide (**7u**): Beige solid, mp 148−150°C (117 mg, 23%); ^1^H NMR (400 MHz, DMSO‐*d*_*6*_) *δ* 10.70 (br. s., 1H), 10.64 (s, 1H), 8.67 (s, 1H), 8.02 (d, *J* = 7.7 Hz, 1H), 7.95 (d, *J* = 7.0 Hz, 1H), 7.63 (t, *J* = 8.1 Hz, 1H), 7.54 (d, *J* = 8.2 Hz, 2H), 7.41 (d, *J* = 15.7 Hz, 1H), 7.05 (d, *J* = 8.4 Hz, 2H), 6.33 (d, *J* = 15.7 Hz, 1H), 4.81 (s, 2H); ^13^C NMR (101 MHz, DMSO‐*d*_*6*_) *δ* 167.2, 163.1, 158.7, 147.9, 139.5, 137.9, 130.3, 129.1, 128.2, 125.7, 118.3, 117.0, 115.1, 113.8, 67.0; HRMS *m/z* [M‐H]^‐^ calcd for C_17_H_14_N_3_O_6_: 356.0877; found: 356.0872.

(2*E*)‐*N*‐Hydroxy‐3‐(4‐{[(4‐nitrophenyl)carbamoyl]methoxy}phenyl)prop‐2‐enamide (7 v): Red−yellow solid, mp 155−157°C (124 mg, 25%); ^1^H NMR (400 MHz, DMSO‐*d*_*6*_) *δ* 11.65−13.00 (bs, 1H), 10.15−10.36 (bs, 1H), 7.64 (dd, *J* = 8.8, 14.0 Hz, 4H), 7.48−7.57 (m, 3H), 7.02 (d, *J* = 8.7 Hz, 2H), 6.39 (d, *J* = 16.0 Hz, 1H), 4.76 (s, 2H); ^13^C NMR (101 MHz, DMSO‐*d*_*6*_) *δ* 167.9, 166.5, 159.4, 143.4, 137.7, 131.6, 129.9, 127.6, 121.6, 117.2, 115.4, 115.1, 67.1; HRMS *m/z* [M‐H]^‐^ calcd for C_17_H_14_N_3_O_6_: 356.0877; found 356.0894.

#### 5.1.3 General procedure for the synthesis of compounds **10a–10v** (methyl esters)

Prepared according to the modified literature procedure.^[^ ^65^ ^]^

To a suspension mixture of (*E*)‐methyl 3‐(4‐hydroxyphenyl)acrylate (1.97 g, 11 mmol) in anhydrous acetone (25 mL) was added anhydrous K_2_CO_3_ (2.76 g, 20 mmol) and NaI (150 mg, 1 mmol). The mixture was stirred at ambient temperature for 30 min and then 2‐chloro‐*N*‐substituted‐phenylacetamide **8a–v** (10 mmol) dissolved in anhydrous acetone was through an additional funnel added dropwise. The mixture was stirred and refluxed for another 24−36 h under argon. Then the mixture was cooled down to room temperature. Solid K_2_CO_3_ was filtered off and the acetone was removed in vacuo to obtain the crude product. The solid residue was purified by recrystallization (EtOH) or column chromatography (SiO_2_, 3%–10% MeOH/DCM) to give the corresponding ester product.

Methyl (2*E*)‐3‐{4‐[(phenylcarbamoyl)methoxy]phenyl}prop‐2‐enoate (**10a**)^[^ ^62^ ^]^: White solid, mp 113−115°C, (1.81 g, 58%); ^1^H NMR (400 MHz, DMSO‐*d*_*6*_) *δ* 10.13 (s, 1H), 7.70 (d, *J* = 8.7 Hz, 2H), 7.58−7.67 (m, 3H), 7.32 (t, *J* = 7.9 Hz, 2H), 7.00−7.12 (m, 3H), 6.51 (d, *J* = 16.0 Hz, 1H), 4.77 (s, 2H), 3.70 (s, 3H); ^13^C NMR (101 MHz, DMSO‐*d*_*6*_) *δ* 166.9, 166.2, 159.7, 144.2, 138.4, 130.1, 128.8, 127.2, 123.7, 119.7, 115.5, 115.1, 67.1, 51.4; HRMS *m/z* [M + Na]^+^ calcd for C_18_H_17_NNaO_4_: 334.1050; found: 334.1050.

Methyl (2*E*)‐3‐(4‐{[(2‐methylphenyl)carbamoyl]methoxy}phenyl)prop‐2‐enoate (**10b**): White solid, mp 137−140°C, (2.51 g, 77%); ^1^H NMR (400 MHz, DMSO‐*d*_*6*_) *δ* 9.54 (br. s., 1H), 7.72 (d, *J* = 8.5 Hz, 2H), 7.63 (d, *J* = 16.0 Hz, 1H), 7.40 (d, *J* = 7.6 Hz, 1H), 7.00−7.27 (m, 5H), 6.52 (d, *J* = 16.0 Hz, 1H), 4.80 (s, 2H), 3.71 (s, 3H), 2.17 (s, 3H); ^13^C NMR (101 MHz, DMSO‐*d*_*6*_) *δ* 166.9, 166.3, 159.6, 144.2, 135.5, 132.2, 130.4, 130.1, 127.3, 126.1, 125.3, 125.7, 115.5, 115.1, 67.0, 51.4, 17.7; HRMS *m/z* [M + H]^+^ calcd for C_19_H_20_NO_4_: 326.1314; found: 326.1378.

Methyl (2*E*)‐3‐(4‐{[(3‐methylphenyl)carbamoyl]methoxy}phenyl)prop‐2‐enoate (**10c**)^[^ ^66^ ^]^: Yellowish solid, mp 144−147°C (2.47 g, 76%), lit. mp 145−148°C; ^1^H NMR (400 MHz, DMSO‐*d*_*6*_) *δ* 10.03 (br. s., 1H), 7.69 (d, *J* = 8.7 Hz, 2H), 7.62 (d, *J* = 16.0 Hz, 1H), 7.38−7.50 (m, 2H), 7.20 (t, *J* = 7.8 Hz, 1H), 7.04 (d, *J* = 8.7 Hz, 2H), 6.90 (d, *J* = 7.4 Hz, 1H), 6.51 (d, *J* = 16.0 Hz, 1H), 4.75 (s, 2H), 3.71 (s, 3H), 2.28 (s, 3H); ^13^C NMR (101 MHz, DMSO‐*d*_*6*_) *δ* 166.9, 166.1, 159.7, 144.2, 138.3, 137.9, 130.1, 128.6, 127.2, 124.4, 120.2, 116.8, 115.5, 115.1, 67.1, 51.3, 21.1; HRMS *m/z* [M + H]^+^ calcd for C_19_H_20_NO_4_: 326.1314; found: 326.1378.

Methyl (2*E*)‐3‐(4‐{[(4‐methylphenyl)carbamoyl]methoxy}phenyl)prop‐2‐enoate (**10d**)^[^ ^66^ ^]^: Yellowish solid, mp 123−126°C, (2.64 g, 81%); ^1^H NMR (400 MHz, DMSO‐*d*_*6*_) *δ* 10.05 (br. s., 1H), 7.44−7.79 (m, 5H), 6.96−7.21 (m, 4H), 6.50 (d, *J* = 16.0 Hz, 1H), 4.75 (s, 2H), 3.70 (s, 3H), 2.25 (s, 3H); ^13^C NMR (101 MHz, DMSO‐*d*_*6*_) *δ* 166.9, 165.9, 159.7, 144.2, 135.8, 132.7, 130.1, 129.1, 127.2, 119.7, 115.5, 115.1, 67.1, 51.3, 20.4; HRMS *m/z* [M + H]^+^ calcd for C_19_H_20_NO_4_: 326.1314; found: 326.1378.

Methyl (2*E*)‐3‐(4‐{[(2‐methoxyphenyl)carbamoyl]methoxy}phenyl)prop‐2‐enoate (**10e**): White solid, mp 101−103°C, (1.60 g, 47%); ^1^H NMR (400 MHz, DMSO‐*d*_*6*_) *δ* 9.26 (s, 1H), 8.05 (d, *J* = 7.7 Hz, 1H), 7.71 (d, *J* = 8.7 Hz, 2H), 7.63 (d, *J* = 16.0 Hz, 1H), 7.01−7.14 (m, 4H), 6.87−6.97 (m, 1H), 6.52 (d, *J* = 16.0 Hz, 1H), 4.83 (s, 2H), 3.85 (s, 3H), 3.71 (s, 3H); ^13^C NMR (101 MHz, DMSO‐*d*_*6*_) *δ* 166.9, 166.0, 159.3, 149.2, 144.1, 130.2, 127.5, 126.5, 124.7, 120.9, 120.4, 115.7, 115.2, 111.2, 67.1, 55.8, 51.4; HRMS *m/z* [M + Na]^+^ calcd for C_19_H_19_NNaO_5_: 364.1155; found: 364.1158.

Methyl (2*E*)‐3‐(4‐{[(3‐methoxyphenyl)carbamoyl]methoxy}phenyl)prop‐2‐enoate (**10f**)^[^ ^66^ ^]^: White solid, mp 142−144°C, (3.11 g, 91%), lit. mp 144−146°C; ^1^H NMR (400 MHz, DMSO‐*d*_*6*_) *δ* 10.11 (s, 1H), 7.70 (d, *J* = 8.5 Hz, 2H), 7.62 (d, *J* = 16.0 Hz, 1H), 7.33 (br. s., 1H), 7.15−7.27 (m, 2H), 7.03 (d, *J* = 8.7 Hz, 2H), 6.66 (d, *J* = 7.4 Hz, 1H), 6.51 (d, *J* = 16.0 Hz, 1H), 4.76 (s, 2H), 3.72 (s, 3H), 3.70 (s, 3H); ^13^C NMR (101 MHz, DMSO‐*d*_*6*_) *δ* 166.9, 166.3, 159.7, 159.5, 144.2, 139.6, 130.1, 129.6, 127.3, 115.5, 115.1, 111.9, 109.2, 105.4, 67.1, 55.0, 51.4; HRMS *m/z* [M + Na]^+^ calcd for C_19_H_19_NNaO_5_: 364.1155; found: 364.1159.

Methyl (2*E*)‐3‐(4‐{[(4‐methoxyphenyl)carbamoyl]methoxy}phenyl)prop‐2‐enoate (**10g**)^[^ ^66^ ^]^: White solid, mp 175−178°C, (2.49 g, 73%), lit. mp 167−172°C; ^1^H NMR (400 MHz, DMSO‐*d*_*6*_) *δ* 9.99 (s, 1H), 7.70 (d, *J* = 8.7 Hz, 2H), 7.62 (d, *J* = 16.0 Hz, 1H), 7.54 (d, *J* = 9.0 Hz, 2H), 7.04 (d, *J* = 8.8 Hz, 2H), 6.90 (d, *J* = 8.9 Hz, 2H), 6.51 (d, *J* = 16.0 Hz, 1H), 4.73 (s, 2H), 3.72 (s, 3H), 3.70 (s, 3H); ^13^C NMR (101 MHz, DMSO‐*d*_*6*_) *δ* 166.9, 165.7, 159.7, 155.6, 144.2, 131.4, 130.1, 127.2, 121.3, 115.5, 115.1, 113.9, 67.1, 55.2, 51.4; HRMS *m/z* [M + Na]^+^ calcd for C_19_H_19_NNaO_5_: 364.1155; found: 364.1156.

Methyl (2*E*)‐3‐(4‐{[(2‐fluorophenyl)carbamoyl]methoxy}phenyl)prop‐2‐enoate (**10h**)^[^ ^66^ ^]^: White solid, mp 144−147°C, (2.24 g, 68%), lit. mp 144−146°C; ^1^H NMR (400 MHz, DMSO‐*d*_*6*_) *δ* 9.76−10.11 (m, 1H), 7.76−7.86 (m, 1H), 7.70 (d, *J* = 8.7 Hz, 2H), 7.62 (d, *J* = 16.0 Hz, 1H), 7.14−7.33 (m, 3H), 7.03 (d, *J* = 8.7 Hz, 2H), 6.52 (d, *J* = 16.0 Hz, 1H), 4.84 (s, 2H), 3.71 (s, 3H); ^19^F NMR (376 MHz, DMSO‐*d*_*6*_) *δ* ‐124.01; ^13^C NMR (101 MHz, DMSO‐*d*_*6*_) *δ* 166.9, 166.7, 159.6, 154.2 (d, *J* = 244.68 Hz, C_q_), 144.2, 130.2, 127.3, 126.0 (d, *J* = 7.71 Hz, CH), 125.4 (d, *J* = 11.56 Hz, C_q_), 124.8, 124.4 (d, *J* = 3.85 Hz, CH), 115.6 (d, *J* = 19.27 Hz, CH), 115.5, 115.1, 68.8, 51.4; HRMS *m/z* [M‐H]^−^ calcd for C_18_H_15_FNO_4_: 328.1063; found: 328.0982.

Methyl (2*E*)‐3‐(4‐{[(3‐fluorophenyl)carbamoyl]methoxy}phenyl)prop‐2‐enoate (**10i**)^[^ ^66^ ^]^: White solid, mp 173−175°C, (3.0 g, 91%), lit. mp 169−172°C; ^1^H NMR (400 MHz, DMSO‐*d*_*6*_) *δ* 10.28 (br. s., 1H), 7.56−7.74 (m, 4H), 7.30−7.43 (m, 2H), 7.05 (d, *J* = 8.6 Hz, 2H), 6.91 (dt, *J* = 1.3, 7.60 Hz, 1H), 6.50 (d, *J* = 16.0 Hz, 1H), 4.78 (s, 2H), 3.71 (s, 3H); ^19^F NMR (376 MHz, DMSO‐*d*_*6*_) *δ* ‐111.89; ^13^C NMR (101 MHz, DMSO‐*d*_*6*_) *δ* 166.9, 166.7, 162.1 (d, *J* = 241.8 Hz, C_q_), 159.6, 144.2, 140.1 (d, *J* = 11.6 Hz, C_q_), 130.5 (d, *J* = 9.6 Hz, CH), 130.2, 127.3, 115.5 (d, *J* = 13.5 Hz, CH), 115.1, 110.2 (d, *J* = 21.2 Hz, CH), 106.4 (d, *J* = 26.0 Hz, CH), 67.0, 51.4; HRMS *m/z* [M‐H]^−^ calcd for C_18_H_15_FNO_4_: 328.1063; found: 328.0983.

Methyl (2*E*)‐3‐(4‐{[(4‐fluorophenyl)carbamoyl]methoxy}phenyl)prop‐2‐enoate (**10j**)^[^ ^66^ ^]^: White solid, mp 170−171°C, (2.70 g, 82%), lit. mp 172−174°C; ^1^H NMR (400 MHz, DMSO‐*d*_*6*_) *δ* 10.19 (br. s., 1H), 7.58−7.75 (m, 5H), 7.17 (t, *J* = 8.9 Hz, 2H), 7.04 (d, *J* = 8.7 Hz, 2H), 6.51 (d, *J* = 16.0 Hz, 1H), 4.76 (s, 2H), 3.70 (s, 3H); ^19^F NMR (376 MHz, DMSO‐*d*_*6*_) *δ* ‐118.66; ^13^C NMR (101 MHz, DMSO‐*d*_*6*_) *δ* 166.9, 166.2, 159.7, 158.3 (d, *J* = 240.8 Hz, C_q_), 144.2, 134.7 (d, *J* = 1.9 Hz, C_q_), 130.1, 127.3, 121.6 (d, *J* = 7.7 Hz, CH), 115.5, 115.3 (d, *J* = 22.2 Hz, CH), 115.1, 67.0, 51.4; HRMS *m/z* [M‐H]^‐^ calcd for C_18_H_15_FNO_4_: 328.1063; found: 328.0983.

Methyl (2*E*)‐3‐(4‐{[(2‐chlorophenyl)carbamoyl]methoxy}phenyl)prop‐2‐enoate (**10k**): Pale brown solid, mp 127−130°C, (2.14 g, 62%); ^1^H NMR (400 MHz, DMSO‐*d*_*6*_) *δ* 9.43−10.00 (bs, 1H), 7.76−7.83 (m, 1H), 7.72 (d, *J* = 8.6 Hz, 2H), 7.63 (d, *J* = 16.0 Hz, 1H), 7.48−7.56 (m, 1H), 7.31−7.40 (m, 1H), 7.18−7.27 (m, 1H), 7.07 (d, *J* = 8.6 Hz, 2H), 6.53 (d, *J* = 16.0 Hz, 1H), 4.85 (s, 2H), 3.71 (s, 3H); ^13^C NMR (101 MHz, DMSO‐*d*_*6*_) *δ* 166.9, 166.6, 159.4, 144.2, 134.2, 130.2, 129.5, 127.6, 127.4, 126.55, 126.63, 125.7, 115.6, 115.2, 66.9, 51.4; HRMS *m/z* [M + Na]^+^ calcd for C_18_H_16_^35^ClNNaO_4_: 368.0660/C_18_H_16_^37^ClNNaO_4_: 370.0636; found: 368.0658.

Methyl (2*E*)‐3‐(4‐{[(3‐chlorophenyl)carbamoyl]methoxy}phenyl)prop‐2‐enoate (**10l**)^[^ ^66^ ^]^: Beige solid, mp 158−161°C, (1.94 g, 56%), lit. mp 157−160°C; ^1^H NMR (400 MHz, DMSO‐*d*_*6*_) *δ* 10.33 (br. s., 1H), 7.84 (s, 1H), 7.70 (d, *J* = 8.7 Hz, 2H), 7.62 (d, *J* = 16.0 Hz, 1H), 7.54 (d, *J* = 8.2 Hz, 1H), 7.35 (t, *J* = 8.1 Hz, 1H), 7.14 (dd, *J* = 1.1, 7.9 Hz, 1H), 7.04 (d, *J* = 8.6 Hz, 2H), 6.51 (d, *J* = 16.0 Hz, 1H), 4.79 (s, 2H), 3.70 (s, 3H); ^13^C NMR (101 MHz, DMSO‐*d*_*6*_) *δ* 166.9, 166.7, 159.6, 144.2, 139.8, 133.1, 130.5, 130.1, 127.3, 123.5, 119.1, 118.1, 115.5, 115.1, 67.0, 51.4; HRMS *m/z* [M‐H]^‐^ calcd for C_18_H_15_^35^ClNO_4_: 344.0768/C_18_H_15_^37^ClNNaO_4_: 346.0738; found: 344.0677.

Methyl (2*E*)‐3‐(4‐{[(4‐chlorophenyl)carbamoyl]methoxy}phenyl)prop‐2‐enoate (**10m**): Pale brown solid, mp 127−130°C, (2.04 g, 59%); ^1^H NMR (400 MHz, DMSO‐*d*_*6*_) *δ* 10.08−10.51 (m, 1H), 7.68 (t, *J* = 8.4 Hz, 4H), 7.62 (d, *J* = 16.0 Hz, 1H), 7.38 (d, *J* = 8.9 Hz, 2H), 7.03 (d, *J* = 8.8 Hz, 2H), 6.51 (d, *J* = 16.0 Hz, 1H), 4.78 (s, 2H), 3.70 (s, 3H); ^13^C NMR (101 MHz, DMSO‐*d*_*6*_) *δ* 166.9, 166.4, 159.6, 144.2, 137.3, 130.1, 128.7, 127.3, 121.2, 115.5, 115.1, 67.0, 51.4; HRMS *m/z* [M‐H]^‐^ calcd for C_18_H_15_^35^ClNO_4_: 344.0768/C_18_H_15_^37^ClNNaO_4_: 346.0738; found: 344.0680.

Methyl (2*E*)‐3‐(4‐{[(2‐bromophenyl)carbamoyl]methoxy}phenyl)prop‐2‐enoate (**10n**): White solid, mp 134−137°C, (2.89 g, 74%); ^1^H NMR (400 MHz, DMSO‐*d*_*6*_) *δ* 9.62 (s, 1H), 7.79 (d, *J* = 7.8 Hz, 1H), 7.66−7.76 (m, 3H), 7.63 (d, *J* = 16.0 Hz, 1H), 7.40 (t, *J* = 7.7 Hz, 1H), 7.16 (t, *J* = 7.3 Hz, 1H), 7.09 (d, *J* = 8.5 Hz, 2H), 6.53 (d, *J* = 16.0 Hz, 1H), 4.83 (s, 2H), 3.71 (s, 3H); ^13^C NMR (101 MHz, DMSO‐*d*_*6*_) *δ* 166.9, 166.5, 159.2, 144.1, 135.4, 132.7, 130.2, 128.3, 127.5, 127.1, 125.8, 117.3, 115.7, 115.2, 66.9, 51.4; HRMS *m/z* [M‐H]^‐^ calcd for C_18_H_15_^79^BrNO_4_: 388.0263/C_18_H_15_^81^BrNO_4_: 390.0242; found: 388.0199.

Methyl (2*E*)‐3‐(4‐{[(3‐bromophenyl)carbamoyl]methoxy}phenyl)prop‐2‐enoate (**10o**): White solid, mp 102−105°C, (3.32 g, 85%); ^1^H NMR (400 MHz, DMSO‐*d*_*6*_) *δ* 10.23 (s, 1H), 7.97 (s, 1H), 7.55−7.73 (m, 4H), 7.24−7.33 (m, 2H), 7.04 (d, *J* = 8.8 Hz, 2H), 6.49 (d, *J* = 16.0 Hz, 1H), 4.78 (s, 2H), 3.71 (s, 3H); ^13^C NMR (101 MHz, DMSO‐*d*_*6*_) *δ* 166.7, 166.5, 159.5, 144.0, 139.8, 130.6, 130.0, 127.3, 126.3, 122.0, 121.4, 118.4, 115.5, 115.0, 67.0, 51.2. HRMS *m/z* [M‐H]^‐^ calcd for C_18_H_15_^79^BrNO_4_: 388.0263/C_18_H_15_^81^BrNO_4_: 390.0242; found: 388.0195.

Methyl (2*E*)‐3‐(4‐{[(4‐bromophenyl)carbamoyl]methoxy}phenyl)prop‐2‐enoate (**10p**)^[^ ^66^ ^]^: White solid, mp 154−155°C, (3.16 g, 81%), lit. mp 158−160°C; ^1^H NMR (400 MHz, DMSO‐*d*_*6*_) *δ* 10.20 (s, 1H), 7.69 (d, *J* = 8.8 Hz, 2H), 7.58−7.65 (m, 3H), 7.47−7.53 (m, 2H), 7.04 (d, *J* = 8.7 Hz, 2H), 6.49 (d, *J* = 16.0 Hz, 1H), 4.76 (s, 2H), 3.71 (s, 3H); ^13^C NMR (101 MHz, DMSO‐*d*_*6*_) *δ* 166.7, 166.3, 159.5, 144.0, 137.6, 131.5, 130.0, 127.2, 121.6, 115.5, 115.3, 115.0, 67.1, 51.2; HRMS *m/z* [M‐H]^‐^ calcd for C_18_H_15_^79^BrNO_4_: 388.0263/C_18_H_15_^81^BrNO_4_: 390.0242; found: 388.0191.

Methyl (2*E*)‐3‐[4‐({[2‐(trifluoromethyl)phenyl]carbamoyl}methoxy)phenyl]prop‐2‐enoate (**10q**): White solid, mp 166−169°C, (3.45 g, 91%); ^1^H NMR (400 MHz, DMSO‐*d*_*6*_) *δ* 10.44 (s, 1H), 7.86 (d, *J* = 8.5 Hz, 2H), 7.69 (d, *J* = 8.7 Hz, 4H), 7.62 (d, *J* = 16.0 Hz, 1H), 7.05 (d, *J* = 8.8 Hz, 2H), 6.50 (d, *J* = 16.0 Hz, 1H), 4.82 (s, 2H), 3.71 (s, 3H); ^19^F NMR (376 MHz, DMSO‐*d*_*6*_) *δ* ‐60.34; ^13^C NMR (101 MHz, DMSO‐*d*_*6*_) *δ* 167.3, 166.9, 159.4, 144.2, 134.7, 133.3, 130.2, 129.2, 127.5, 126.9, 126.5 (q, *J* = 5.8 Hz, CH), 124.3 (q, *J* = 29.9 Hz, C_q_), 123.6 (q, *J* = 273.6 Hz, CF_3_), 115.6, 115.1, 66.8, 51.4; HRMS *m/z* [M‐H]^‐^ calcd for C_19_H_15_F_3_NO_4_: 378.1031; found: 378.0949.

Methyl (2*E*)‐3‐[4‐({[3‐(trifluoromethyl)phenyl]carbamoyl}methoxy)phenyl]prop‐2‐enoate (**10r**)^[^ ^66^ ^]^: White solid, mp 154−155°C, (3.22 g, 85%), lit. mp 156−158°C; ^1^H NMR (400 MHz, DMSO‐*d*_*6*_) *δ* 9.36−10.14 (m, 1H), 7.59−7.80 (m, 6H), 7.42−7.51 (m, 1H), 7.04 (d, *J* = 8.6 Hz, 2H), 6.53 (d, *J* = 16.0 Hz, 1H), 4.81 (s, 2H), 3.71 (s, 3H); ^19^F NMR (376 MHz, DMSO‐*d*_*6*_) *δ* ‐59.22; ^13^C NMR (101 MHz, DMSO‐*d*_*6*_) *δ* 166.8, 166.7, 159.5, 144.0, 141.9, 130.0, 127.3, 125.9 (q, *J* = 2.9 Hz, CH), 124.2 (q, *J* = 271.7 Hz, CF_3_), 123.7 (q, *J* = 31.8 Hz, C_q_), 119.5, 115.5, 115.0, 67.0, 51.2; HRMS *m/z* [M‐H]^‐^ calcd for C_19_H_15_F_3_NO_4_: 378.1031; found: 378.0950.

Methyl (2*E*)‐3‐[4‐({[4‐(trifluoromethyl)phenyl]carbamoyl}methoxy)phenyl]prop‐2‐enoate (**10s**)^[^ ^66^ ^]^: White solid, mp 167−170°C, (3.34 g, 88%), lit. mp 162−163°C; ^1^H NMR (400 MHz, DMSO‐*d*_*6*_) *δ* 9.76 (br. s., 1H), 7.59−7.81 (m, 6H), 7.42−7.53 (m, 1H), 7.04 (d, *J* = 8.4 Hz, 2H), 6.53 (d, *J* = 16.0 Hz, 1H), 4.81 (s, 2H), 3.71 (s, 3H); ^19^F NMR (376 MHz, DMSO‐*d*_*6*_) *δ* ‐60.28; ^13^C NMR (101 MHz, DMSO‐*d*_*6*_) *δ* 167.0, 166.9, 159.6, 144.2, 142.0, 130.2, 127.3, 126.1 (q, *J* = 3.9 Hz, CH), 124.4 (q, *J* = 271.7 Hz, CF_3_), 123.7 (q, *J* = 31.8 Hz, C_q_), 119.5, 115.5, 115.1, 67.0, 51.4; HRMS *m/z* [M‐H]^‐^ calcd for C_19_H_15_F_3_NO_4_: 378.1031; found: 378.0952.

Methyl (2*E*)‐3‐(4‐{[(2‐nitrophenyl)carbamoyl]methoxy}phenyl)prop‐2‐enoate (**10t**): Pale brown solid, mp 124−127°C, (2.03 g, 57%); ^1^H NMR (400 MHz, DMSO‐*d*_*6*_) *δ* 10.87 (s, 1H), 8.12 (dd, *J* = 8.3, 15.5 Hz, 2H), 7.70−7.82 (m, 3H), 7.63 (d, *J* = 16.0 Hz, 1H), 7.39 (t, *J* = 7.8 Hz, 1H), 7.09 (d, *J* = 8.6 Hz, 2H), 6.54 (d, *J* = 16.0 Hz, 1H), 4.84 (s, 2H), 3.71 (s, 3H); ^13^C NMR (101 MHz, DMSO‐*d*_*6*_) *δ* 166.97, 166.91, 159.0, 144.1, 140.1, 135.0, 131.7, 130.2, 127.7, 125.5, 125.1, 123.9, 115.8, 115.2, 67.0, 51.4. HRMS *m/z* [M + H]^+^ calcd for C_18_H_17_N_2_O_6_: 357.1008; found: 357.1070.

Methyl (2*E*)‐3‐(4‐{[(3‐nitrophenyl)carbamoyl]methoxy}phenyl)prop‐2‐enoate (**10u**)^[^ ^66^ ^]^: Pale yellow solid, mp 184−185°C, (2.10 g, 59%), lit. mp 181−184°C; ^1^H NMR (400 MHz, DMSO‐*d*_*6*_) *δ* 10.31−10.97 (m, 1H), 8.66 (t, *J* = 2.00 Hz, 1H), 7.90−8.06 (m, 2H), 7.56−7.75 (m, 4H), 7.06 (d, *J* = 8.7 Hz, 2H), 6.51 (d, *J* = 16.0 Hz, 1H), 4.83 (s, 2H), 3.70 (s, 3H); ^13^C NMR (101 MHz, DMSO‐*d*_*6*_) *δ* 167.1, 166.9, 159.5, 147.9, 144.1, 139.5, 130.2, 130.1, 127.4, 125.7, 118.3, 115.6, 115.1, 113.8, 67.0, 51.3; HRMS *m/z* [M + H]^+^ calcd for C_18_H_17_N_2_O_6_: 357.1008; found: 357.1067.

Methyl (2*E*)‐3‐(4‐{[(4‐nitrophenyl)carbamoyl]methoxy}phenyl)prop‐2‐enoate (**10v**): Yellowish solid, mp 107−110°C, (1.75 g, 49%); ^1^H NMR (400 MHz, DMSO‐*d*_*6*_) *δ* 10.75 (s, 1H), 8.24 (d, *J* = 9.2 Hz, 2H), 7.90 (d, *J* = 9.3 Hz, 2H), 7.70 (d, *J* = 8.8 Hz, 2H), 7.62 (d, *J* = 16.1 Hz, 1H), 7.04 (d, *J* = 8.7 Hz, 2H), 6.51 (d, *J* = 16.0 Hz, 1H), 4.86 (s, 2H), 3.70 (s, 3H); ^13^C NMR (101 MHz, DMSO‐*d*_*6*_) *δ* 167.3, 166.9, 159.6, 144.6, 144.2, 142.5, 130.1, 127.4, 125.0, 119.3, 115.6, 115.1, 67.0, 51.4; HRMS *m/z* [M + Na]^+^ calcd for C_18_H_16_N_2_NaO_6_: 379.0901; found: 379.0900.

#### 5.1.4 General procedure for the synthesis of compounds **9a**, **9e–9g**, **9k–9m** (phenyl substituted aldehydes)

Prepared according to the modified literature procedure.^[^ ^65^ ^]^

To a suspension mixture of *p‐*hydroxybenzaldehyde (1.35 g, 11 mmol) in anhydrous acetone (25 mL) was added anhydrous K_2_CO_3_ (2.76 g, 20 mmol) and NaI (150 mg, 1 mmol). The mixture was stirred at ambient temperature for 30 min, and then 2‐chloro‐*N*‐substituted‐phenylacetamide **9a**, **9e–9g**, **9k–9m** (10 mmol) dissolved in anhydrous acetone, through an additional funnel added dropwise. The mixture was stirred and refluxed for another 24–36 h under Ar. Then, the mixture was cooled down to room temperature. Solid K_2_CO_3_ was filtered off, and the acetone was removed in vacuo to obtain the crude product. The solid residue was purified by column chromatography (SiO_2_, *n*‐hexane‐EtOAc = 2:1) to give the corresponding product.

(9a) white solid, mp 132°C, yield: 25% (mp 132−134°C^[^ ^65^, ^67^ ^]^); (**9e**) white solid, mp 111−114°C, yield: 28% (mp 110−111°C^[^ ^68^ ^]^); (**9f**) white solid, mp 108°C, yield: 23% (mp 109°C^[^ ^69^ ^]^); (**9g**) white solid, mp 145−146°C, yield: 18% (mp 149°C^[^ ^67^, ^68^, ^70^ ^]^); (9k) white solid, mp 128−130°C, yield: 36%^[^ ^68^ ^]^; (**9l**) pale brown solid, mp 106−108°C, yield: 27%^[^ ^65^, ^67^ ^]^; (**9m**) white solid, mp 129−130°C, yield: 15% (mp 134−135°C^[^ ^65^, ^67^, ^70^ ^]^).

#### 5.1.5 General procedure for the synthesis of 2‐chloro‐N‐substituted‐acetamides **8a–8v**

Prepared according to the modified literature procedure.^[^ ^65^ ^]^

The corresponding aniline R‐NH_2_ (40 mmol) was dissolved in dry DCM (35 mL) and dry Et_3_N (6.6 mL, 24 mmol) and cooled to 0°C in an ice salt bath. Then 2‐chloroacetyl chloride (3.8 mL, 48 mmol) was added dropwise to the mixture keeping the temperature below 0°C. The reaction mass was left to stir in an ice‐salt bath for an additional 1 h. Then the temperature in the flask was gradually warmed up to room temperature and is left to stir overnight. After starting material was consumed, the mixture was concentrated in vacuo*.* The solid residue was washed with 3 × 40 mL of ice−cold water. The crude product was dissolved in a minimum amount of DCM and purified by short column chromatography (SiO_2_, 100% DCM), or recrystallized from EtOH/H_2_O.

(**8a**) white solid, mp 129−130°C, yield: 77% (mp 135−138°C^[^ ^71^ ^]^); (**8b**) white solid, mp 158−161°C, yield: 73% (mp 164−166°C^[^ ^71^ ^]^); (**8c**) white solid, mp 167−170°C Yield: 87% (mp 168−170°C^[^ ^71^ ^]^); (**8d**) white solid, mp 158−159°C Yield: 86% (mp 161−163°C^[^ ^71^ ^]^), (**8e**) white solid, mp 40−42°C Yield: 87% (mp 40−42°C^[^ ^72^ ^]^); (**8f**) yellowish solid, mp 91−92°C, yield: 69% (mp 93−94°C^[^ ^73^ ^]^); (**8g**) white solid, mp 121−123°C, yield: 75% (mp 119−120°C^[^ ^71^ ^]^) (**8h**) white solid, mp 134−136°C, yield: 89% (mp 130−132°C^[^ ^74^ ^]^); (**8i**) white solid, mp 120−122°C yield:76% (mp 121−123°C^[^ ^75^ ^]^); (**8j**) grey solid, mp 131−133°C, yield: 75% (mp 130−131°C^[^ ^76^ ^]^); (8k) white solid, mp 61−63°C, yield: 80% (mp 65−67°C^[^ ^72^ ^]^; (**8l**) white solid, mp 99−100°C, yield: 64% (mp 98−99°C^[^ ^72^ ^]^); (**8m**) light yellow solid, mp 166−167°C, yield: 81% (mp 168−170°C^[^ ^71^ ^]^); (**8n**) light yellow solid, mp 69−70°C, yield: 55% (mp 75−77°C^[^ ^77^ ^]^); (**8o**) pale brown solid, mp 107−109°C, yield: 56% (mp 110−113°C^[^ ^78^ ^]^); (**8p**) yellow solid, mp 179−180°C, yield: 64% (mp 180−182°C^[^ ^75^ ^]^); (**8q**) white solid, mp 100−102°C, yield: 73% (mp 99−101°C^[^ ^79^ ^]^); (**8r**) white solid, mp 82−84°C, yield: 80% (mp 84−86°C^[^ ^74^ ^]^); (**8s**) white solid, mp 154−156°C, yield: 91% (mp 155−156°C^[^ ^74^ ^]^); (**8t**) yellow solid, mp 169−170°C, yield: 71% (mp 176−180°C^[^ ^71^ ^]^); (**8u**) yellow solid, mp 177−179°C, yield: 65% (mp 181−183°C^[^ ^71^ ^]^), (**8v**) yellow solid, mp 188−190°C, yield: 68% (mp 185−186°C^[^ ^71^ ^]^).

#### 5.1.6 General procedure for the synthesis of (E)‐methyl 3‐(4‐hydroxyphenyl)acrylate (11)

##### Route A

(*E*)‐Methyl 3‐(4‐hydroxyphenyl)acrylate (**11**): Prepared according to the modified literature procedure.^[^ ^27^ ^]^ To a suspension of 4‐hydroxybenzenediazonium tetrafluoroborate (10 g, 48.1 mmol), methyl acrylate (8.7 mL, 96.2 mmol), and anhydrous NaOAc (11.83 g, 114.2 mmol) in 40 mL dry MeOH was added precatalyst Pd(OAc)_2_ (0.25 g, 2.5 mol%) at an ambient temperature. The reaction mixture was stirred under Ar for 24 h or until the starting material was consumed. Then an active charcoal (5 g) was added, and the mass was stirred for 10 min. The mixture was filtered through a pad of Celite® and the solvent was evaporated in vacuoThe solid residue was washed with cold water (150 mL) and purified by recrystallization (DCM/PET). The solid was dried at room temperature in vacuoto afford 10 as a white solid (6.44 g, 75%), mp 133−135°C (reported in the literature 134−136°C).

4‐Hydroxybenzenediazonium tetrafluoroborate: Prepared according to the modified literature procedure.^[^ ^27^ ^]^ A suspension of *N‐*(4‐hydroxyphenyl)acetamide (50.0 g, 330 mmol) in HBF_4_ (70.6 mL, 48% solution in water) and propan‐2‐ol (50 mL) was heated at 90°C for 3 h to reach completely dissolved mixture indicating complete consumption of the starting material. The mixture was cooled to 0°C in an ice bath and solid NaNO_2_ (31.0 g, 440 mmol) and was gradually added in small portions to keep the temperature in the mixture below 5°C. The resulting suspension was stirred for another 30 min at 0°C. The resulting solid was filtered off and washed with cold diethyl ether (200 mL) and dried in vacuo at room temperature, giving 11 as pale‐yellow solid (49.0 g, 71%), mp 134−135°C (reported in the literature 135−140°C^[^ ^80^ ^]^).

##### Route **B**

(*E*)‐Methyl 3‐(4‐hydroxyphenyl)acrylate (**11**): Prepared according to the modified literature procedure.^[^ ^26^ ^]^ (2*E*)‐3‐(4‐Hydroxyphenyl)prop‐2‐enoic acid (5.0 g 30.5 mmol) was dissolved in 35 mL anhydrous MeOH containing a catalytic amount (0.8 mL) of conc. H_2_SO_4,_ and then the mixture was heated at reflux for 4 h (controlled by TLC 3% MeOH/DCM). After cooling at room temperature, the solvent was evaporated in vacuo. The solid residue was dissolved in EtOAc and washed with a sat. aq NaHCO_3_ to reach a neutral pH in the organic layer. The organic layers were then washed with water (20 mL) and dried over anhydrous Na_2_SO_4_. The solvent was removed *in vacuo* to afford 10 as a white solid powder (5.1 g, 94%) mp 133−135°C (reported in the literature 134−136°C).^[^ ^27^ ^]^

(2*E*)‐3‐(4‐Hydroxyphenyl)prop‐2‐enoic acid: Prepared according to the modified literature procedure.^[^ ^26^ ^]^ To a suspension of malonic acid (18.6 g, 17.9 mmol) and *p‐*hydroxybenzaldehyde (10 g, 81.5 mmol) in pyridine (44.7 mL) was added piperidine (0.8 mL, 8.3 mmol). The mixture was stirred for 2 h at 130°C. After completion of the reaction (controlled by TLC EtOAc: DCM = 1:4), the reaction was quenched with 100 mL of cold water and then slowly acidified to pH=3 using 6 M HCl. The yellow‐coloured solid precipitate was filtered off and dried in vacuo to afford 14 as pale yellow solid (10.48 g, 78%), mp^[^ ^81^ ^]^ 210−212°C (reported in the literature 210.5−211°C).

##### Route **C**

(*E*)‐Methyl 3‐(4‐hydroxyphenyl)acrylate (**11**): Prepared according to the modified literature procedure.^[^ ^28^ ^]^ To 10 mL vial with a mixture of 4‐Iodophenol (110 mg, 0.5 mmol), methyl acrylate (51.6 mg, 0.6 mmol), Pd(OAc)_2_ added solvent mixture of 4.5 mL DMF, 0.5 mL water and 0.5 ml Et_3_N. The resulting mixture was stirred and electrolysed with constant current (15 mA) on RVC electrodes at room temperature under Ar for 3 h. Then, the 1 M HCl was added to the reaction mixture to reach a pH below 5. The resulting suspension was repetitively extracted with DCM. The combined organic layers were washed with water and brine, dried over anhydrous Na_2_SO_4_, and evaporated. The crude product was purified by column chromatography (SiO_2_, 2.5% MeOH/DCM) and dried, giving **11** as a white solid product (70.5 mg, 79%), mp 132−135°C. (reported in the literature 134−136°C).^[^ ^27^ ^]^

#### 5.1.7 General procedure for the synthesis of SAHA

Suberanilic acid (15): Prepared according to the modified literature procedure.^[^ ^62^ ^]^ Suberic acid (6.96 g, 40.0 mmol) and freshly distilled aniline (4.09 g, 44.0 mmol) were mixed in a round bottom flask equipped with a stirring bar and heated at 185−190°C for 15 min. The mixture was cooled down and then dispersed in a solution of 4.0 g of KOH in 50 mL of water in an ultrasonic bath. The resulting white suspension was filtered off, and the solid was rinsed with water (50 mL). The clear filtrate was acidified with 1 M HCl. The resulting fluffy white precipitate was filtered off. The solid was mixed with 100 mL of water and heated to 50°C and left to stir for 10 min. This was filtered hot, and the solid was washed again two times with another 80 mL of hot water. The final product was dried in vacuo at 50°C to give 15 as white solid (4.18, 42%). mp 125−126°C (reported in the literature mp 126−128°C^[^ ^62^ ^]^). ^1^H NMR (400 MHz, DMSO‐*d*_*6*_) *δ* 9.85 (s, 1H), 7.57 (d, *J* = 7.9 Hz, 2H), 7.27 (t, *J* = 7.9 Hz, 2H), 7.01 (t, *J* = 7.4 Hz, 1H), 2.28 (t, *J* = 7.3 Hz, 2H), 2.14−2.23 (m, 2H), 1.41−1.63 (m, 5H), 1.19−1.34 (m, 5H); ^13^C NMR (101 MHz, DMSO‐*d*_*6*_) *δ* 171.6, 171.3, 139.4, 128.7, 123.0, 119.1, 36.4, 33.7, 28.44, 28.38, 25.1, 24.4.

Methyl Suberanilate (16): Prepared according to the modified literature procedure.^[^ ^62^ ^]^ Suberanilic acid (4.14 g, 16.6 mmol) was dissolved in 25 mL of MeOH, and 0.5 g of Dowex 50W‐X2 acid resin was added. This mixture was slowly heated at reflux and left to stir for another 22 h in reflux. Then mass was cooled, filtered, and methanol was evaporated in vacuo to afford a yellowish solid. The resulting solid was dissolved in 10 mL of CHCl_3_ and purified by short column chromatography (SiO_2_, 100% CHCl_3_). The solvent was removed in vacuo to afford 16 as pale yellow solid (3.72 g, 85%), mp. 63−66°C (reported in the literature.^[^ ^62^ ^]^ mp. 63−66°C (reported in the literature^[^ ^62^ ^]^ mp 64−65.5°C). ^1^H NMR (400 MHz, CDCl_3_) *δ* 7.52 (d, *J* = 8.0 Hz, 2H), 7.30 (t, *J* = 7.8 Hz, 2H), 7.04−7.15 (m, 1H), 3.67 (s, 3H), 2.33 (td, *J* = 7.5, 12.5 Hz, 4H), 1.55−1.84 (m, 4H), 1.26−1.44 (m, 4H); ^13^C NMR (101 MHz, CDCl_3_) *δ* 171.2, 171.4, 138.0, 128.9, 124.1, 119.8, 51.5, 37.5, 33.9, 28.70, 28.67, 25.3, 24.6.

Suberoylanilide hydroxamic acid (1): Prepared according to the modified literature procedure^[^ ^62^ ^]^: Hydroxyazanium chloride (2.17 g, 31.2 mmol) and 1 mg of phenolphthalein were dissolved in 15 mL of dry MeOH in a flask with an addition funnel. A solution of freshly prepared sodium metal (1.08 g, 46.8 mmol) in 13 mL of dry MeOH was placed in the addition funnel, and enough was added to reach a pink endpoint. Solid methyl suberanilate (4.10 g, 15.6 mmol) was added, which was dissolved readily in dry MeOH. The remainder of the sodium methoxide solution was added, and the mixture was stirred overnight under Ar at room temperature. Then the mixture was rinsed into 100 mL of cold distilled water, and glacial acetic acid (4.0 g) was added with stirring. The resulting heavy white precipitate was suction‐filtered and rinsed repetitively with cold water (150 mL). The solid was dried at room temperature to afford 1 as white solid (1.90 g, 87%), mp 157−158°C (reported in the literature 159−160.5°C^[^ ^62^ ^]^).

### 5.2 Pharmacological/biological assays

#### 5.2.1 Cell culture, materials, and experimental conditions

For in vitro assays, the human monocytic leukaemia cell line THP‐1 was used. Cell line THP‐1 was purchased from the European Collection of Cell Cultures (Salisbury). The cells were cultured in Roswell Park Memorial Institute (RPMI 1640 medium supplemented with 10% of foetal bovine serum, antibiotic solution (100 U/mL of penicillin, 100 µg/mL of streptomycin), and 2 mmol/L of l‐glutamine, all were obtained from HyClone Laboratories Inc. (GE Healthcare). The cells were maintained in an incubator with 5% CO_2_ at 37°C. The test substances were dissolved in DMSO from Sigma Aldrich. The final concentration of DMSO in experiments never exceeded 0.1%, while solutions of the substances were prepared fresh before each experiment.

#### 5.2.2 Analysis of antiproliferative and cytotoxic effects

THP‐1 cells were seeded into 96‐well plates and treated with an indicated broad concentration range of vorinostat **1**, **7d,** or **7p**. Cell proliferation was determined after 24 h, 48 h, and 72 h incubation using Cell Proliferation Reagent WST‐1 (2‐(4‐iodophenyl)‐3‐(4‐nitrophenyl)‐5‐(2,4‐disulfophenyl)‐2H‐tetrazolium) (Roche Diagnostics) as it was previously described.^[^ ^82^, ^83^ ^]^ Cytotoxicity of vorinostat **1**, **7d,** or **7p** was assessed after 24 and 48 h using Cytotoxicity Detection Kit (lactate dehydrogenase) (Roche Diagnostics) as described previously.^[^ ^84^, ^85^ ^]^ Measurements were performed using an Epoch Microplate Spectrophotometer (BioTek). The results show the average of three independent measurements performed in triplicate. The IC_50_ was determined using the nonlinear regression four‐parameter logistic model using GraphPad Prism 5.00 software (GraphPad Software).

#### 5.2.3 Western blot analysis

Briefly, THP‐1 cells were treated with vorinostat **1**, **7d,** and **7p** compounds and incubated for indicated time before cell lyses with radioimmunoprecipitation assay (RIPA) buffer supplemented with phenylmethanesulfonyl fluoride (PMSF, 1 mM), both from Cell Signalling Technology, and protease and phosphatase inhibitor cocktails (Roche Diagnostics). Protein concentration was determined by Roti®‐Quant universal (Carl Roth), and subsequently, sodium dodecyl sulfate polyacrylamide (SDS‐PAGE) and transfer onto nitrocellulose membranes were carried out according to the previously described protocol.^[^ ^82^, ^86^ ^]^ Membranes were stained overnight at 4°C with appropriate primary antibodies: anti‐β‐actin antibody (Santa Cruz Biotechnology), antibodies against cyclin E1 (Clone: HE12, Cat. #: sc‐247), p‐Rb [Ser 807/811] (sc‐16670‐R), cleaved PARP (D64E10, 5625), acetyl‐histone H2A [Lys5] (2576), acetyl‐histone H2B [Lys5] (D5H1S, 12799), acetyl‐histone H3 [Lys9] (C5B11, 9649) and acetyl‐histone H4 [Lys8] (2594) were purchased Cell Signalling Technology. Antibody against cyclin B1 (ab2949) was purchased from Abcam. Subsequently, membranes were incubated with appropriate secondary antibodies, anti‐rabbit immunoglobulin G (IgG) horseradish peroxidase (HRP)‐linked antibody (7074) or anti‐mouse IgG HRP‐linked antibody (7076), both purchased from Cell Signalling Technology. Visualization of proteins was performed by ECL Plus reagent. Semi‐quantitative analysis of band intensity was carried out with the ImageJ software (National Institute of Mental Health).

#### 5.2.4 Analysis of HDAC class I and II enzyme activity

The inhibitory effects of vorinostat **1**, **7d,** and **7p** against enzyme activity of HDAC class I and II were determined using HDAC‐Glo™ I/II Assay (Cat. #: G6420; Promega Corporation) according to the manufacturer's instructions. THP‐1 cells were seeded into a white‐walled 96‐well plate in density of 7.5 × 10^3^ cells/well in serum‐free and phenol red‐free RPMI medium. Cells were treated with vorinostat **1**, **7d,** and **7p** to their final concentration ranging 0.01–1 μM and to a final volume of 100 μL per well. The plate was shortly mixed using an orbital shaker at room temperature and incubated for 4 h at 37°C. Meanwhile, the HDAC‐Glo™ I/II Reagent was prepared by adding 10 mL of HDAC‐Glo™ I/II Buffer to the HDAC‐Glo™ I/II Substrate and mixing with 10 μL of Developer Reagent. After the end of incubation, 100 μL of HDAC‐Glo™ I/II Reagent were added into each well, shortly mixed using an orbital shaker, and incubated at room temperature for approx. 30 min. The chemiluminescence of the well content was measured using Cytation 3 Cell Imaging Multi‐Mode Reader (BioTek).

#### 5.2.5 Flow cytometric analysis of apoptosis

The externalization of phosphatidylserine was assessed using Annexin V‐FITC (fluorescein isothiocyanate) Early Apoptosis Detection Kit (Cell Signalling Technology) according to the manufacturer's instructions. At the end of incubation of THP‐1 cells with vorinostat **1**, **7d** or **7p**, cells were harvested, washed with ice‐cold 1× PBS, and resuspended in with 1× annexin V binding buffer (1 × 10^6^ cell/mL). 1 µL of annexin V‐FITC conjugate and 12.5 µL of propidium iodide (PI) were added to 96 µL of cell suspension and samples were kept on ice for 10 min, protected from light. Then the cell suspension was further diluted to a final volume of 250 µL with 1 × annexin V binding buffer. The following analysis was performed by flow cytometry. Caspase 3 activation was assessed using CellEvent™ Caspase 3/7 Green Detection Reagent (Molecular Probes Inc.). THP‐1 cells previously incubated with vorinostat **1**, **7d,** or **7p** were harvested and resuspended in 1 × PBS (1 × 10^6^ cell/mL). 1 μL of CellEvent™ Caspase‐3/7 Green Detection Reagent was added to 1 mL of each sample, followed by incubation of the samples for 30 min at 37°C protected from light. The analyses were performed using flow cytometer BriCyte‐E6 (Mindray) in FITC and PE channels (laser: 488 nm), and data were analysed with Kaluza Flow Cytometry Analysis Software 1.2 (Beckman Coulter).

#### 5.2.6 Cell‐cycle analysis

THP‐1 cells were treated and subsequently incubated with indicated concentrations vorinostat of vorinostat **1**, **7d,** and **7p** for 48 h. Cell‐cycle analysis was performed as it was previously.^[^ ^82^ ^]^ Briefly, cells were harvested and washed twice with 1× PBS. After fixation in 70% ethanol samples were stored at −20°C overnight. Cells were then collected by centrifugation and the cell pellet was washed twice with 1× PBS followed by incubation with RNaseA (0.02 mg/mL) and 0.05% (v/v) Triton X‐100 in 1× PBS for 30 min at 37°C. Before flow cytometric analysis, the nuclei were stained with propidium iodide (PI) (0.04 mg/mL). Cell‐cycle distribution was analysed using a flow cytometer BriCyte‐E6 (Mindray). The quantification of cell‐cycle distribution was carried out using the software Kaluza Flow Cytometry Analysis Software 1.2 (Beckman Coulter). A total number of 2 × 10^4^ cells were analysed per sample.

### 5.3 Molecular docking

#### 5.3.1 HDACis docking and scoring

Crystal structures of the HDAC inhibitor complexes were obtained from the Protein Data Bank^[^ ^87^ ^]^ for HDAC of class I (HDAC1, PDB ID *5ICN* ^[^ ^88^ ^]^; HDAC2, PDB ID *7KBG* ^[^ ^89^ ^]^; HDAC3, PDB ID *4A69* ^[^ ^90^ ^]^; and HDAC8, PDB ID *5VI6* ^[^ ^91^ ^]^) and class II (HDAC4, PDB ID *2VQM* ^[^ ^92^ ^]^; HDAC6, PDB ID *5EDU* ^[^ ^93^ ^]^; and HDAC7, PDB ID *3ZNR* ^[^ ^94^ ^]^). The three‐dimensional (3D) structures of the complexes were processed by the Protein Preparation Wizard (Maestro, release 2022‐3, Schrödinger LLC, USA, 2022^[^ ^95^ ^]^). During processing of the complexes, redundant protein chains and water molecules were removed, hydrogen atoms added, terminal residues capped, bond orders assigned, protonation states and tautomeric structures for ligands and charge states of metals generated at neutral pH using Epik,^[^ ^96^ ^]^ protonation of amino acid side chains adjusted to pH 7, and hydrogen bonds optimized using PROPKA.^[^ ^97^ ^]^ The complexes were refined by molecular mechanics (MM) energy minimization to a minimum in the potential energy landscape by employing the Polak‐Ribière conjugate gradient method with the convergence criterion set to an energy gradient of 0.01 kJ·mol^‐1^·Å^‐1^. During geometry optimization, an extended cut‐off distance of 20 Å was used for electrostatic interactions. The OPLS4 force field, which is suitable for small‐molecule and protein simulations,^[^ ^98^ ^]^ together with the implicit solvent model (water),^[^ ^99^ ^]^ was used throughout the minimization of protein complexes (MacroModel, release 2022‐3, Schrödinger LLC, USA, 2022). The 3D structures of the optimized HDAC‐inhibitor complexes were aligned using HDAC1^[^ ^88^ ^]^ as a template. Hydroxamate ligands were built using 2D Sketcher and LigPrep modules (Maestro, release 2022‐3, Schrödinger LLC, USA, 2022). LigPrep generates tautomeric and ionization states, ring conformations, and stereoisomers of ligands. The Epik module^[^ ^96^ ^]^ was used to determine the most probable ligand structures at neutral pH. Ligand models were minimized using OPLS4 force field.^[^ ^98^ ^]^ Afterwards, receptor grids for the docking of hydroxamate ligands to HDAC binding sites bounded by regions occupied by the co‐crystallized ligand were set up and calculated. Inhibitor docking to individual HDAC targets was performed in Glide (release 2022‐3, Schrödinger LLC, USA, 2022^[^ ^40^, ^41^ ^]^). Glide is a ligand docking programme that is used to predict binding poses and ligand ranking according to the calculated docking score. The XP protocol and the XP GlideScore scoring function were used for all HDACis using generated receptor grids.^[^ ^40^, ^41^ ^]^ Core pattern comparison of the hydroxamate group position from the crystal structure of human HDAC8 complexed with trichostatin A (PDB ID *3F0R*)^[^ ^32^ ^]^ was used for docking of hydroxamic acid derivatives to the class I and II HDACs considering the bidenate chelation types **A** and **C**. Instead, for type **B** monodenate zinc chelation, the core pattern comparison of hydroxamate group in the crystal structure of HDAC4 (PDB ID *2VQM* ^[^ ^92^ ^]^) was used. The docking was restricted to the reference position of the hydroxamate group within a tolerance of 0.1 Å. To conserve the bidentate metal coordination of the zinc ion by the hydroxamate group, a constraint was applied. The protein structures were modified to direct imine nitrogen of the closest histidine to the oxygen atom of the hydroxamate group. Penalization was used for low‐probability ionization states. Five thousand poses for flexible ligands with three different binding modes of the hydroxamate function group, which coordinates the zinc ion located at the bottom of the ligand binding cleft of HDACs were considered:

(**A**) bidenate metal chelation by neutral hydroxamate group with two oxygen atoms coordinating Zn^2+^, amide nitrogen forms a hydrogen bond to N_ε_ atom of the proximate His143, (Figure 8);

(**B**) monodenate metal chelation of Zn^2+^ by carbonyl oxygen of neutral hydroxamate group, hydroxyl group forms a hydrogen bond to N_ε_ atom of the proximate His142;

(**C**) bidenate metal chelation by anionic hydroxamate group with two oxygen atoms coordinating Zn^2+^, the proton was transferred from the hydroxyl group to N_ε_ atom of the proximate His142.

## CONFLICTS OF INTEREST STATEMENT

The authors declare no conflicts of interest.

## Supporting information

Supporting information.

Supporting information.

## Data Availability

The data that support the findings of this study are available in the supplementary material of this article.
