# Supplementary material for: New potent N‐hydroxycinnamamide‐based histone deacetylase inhibitors suppress proliferation and trigger apoptosis in THP‐1 leukaemia cells
Source: Arch Pharm (Weinheim). 2025 Apr 1;358(4):e2400889. doi: 10.1002/ardp.202400889 (PMC11959351; doi:10.1002/ardp.202400889)
Supplement: Supplementary file 1 — Supporting information. [file ARDP-358-e2400889-s001.doc]

**Supplemental Material: Novel Compounds and Biological Screening Results**

**New Potent *N*-Hydroxycinnamamide-Based HDAC Inhibitors Inhibits Cell Growth, Affect Cell Cycle, and Induce Apoptosis in THP-1 Monocytic Leukemia Cells**

Magdalena Onuscakova^1‡^, Tereza Kauerova^2‡^, Eva Fialova^1^, Hana Pizova^1^, Vladimir Garaj^3^, Miroslav Kemka^3^, Vladimir Frecer^4^, Peter Kollar^2^*, Pavel Bobal^1^*

1 Department of Chemical Drugs, Faculty of Pharmacy, Masaryk University, Palackeho 1946/1, 612 00 Brno, Czech Republic

2 Department of Pharmacology and Toxicology, Faculty of Pharmacy, Masaryk University, Palackeho 1946/1, 612 00 Brno, Czech Republic

3 Department of Pharmaceutical Chemistry, Faculty of Pharmacy, Comenius University Bratislava, Odbojarov 10, 832 32 Bratislava, Slovakia

4 Department of Physical Chemistry of Drugs, Faculty of Pharmacy, Comenius University Bratislava, Odbojarov 10, 832 32 Bratislava, Slovakia

^‡^ These authors contributed equally (Joint first authorship)

*Correspondence:

* Pavel Bobal – Department of Chemical Drugs, Faculty of Pharmacy, Masaryk University, Palackeho 1946/1, 612 00 Brno, Czech Republic; orcid.org/0000-0002-8429-3810.

Email: [bobalp@pharm.muni.cz](mailto:bobalp@pharm.muni.cz)

* Peter Kollar – Department of Pharmacology and Toxicology, Faculty of Pharmacy, Masaryk University, Palackeho 1946/1, 612 00 Brno, Czech Republic; orcid.org/0000-0003-2265-1528.

Email: kollarp@pharm.muni.cz

| **Compound No.** | **InChI** | **IC_50_ (μM) ± SD (24h)^a^** | **IC_50_ (μM) ± SD (48h)^a^** | **IC_50_ (μM) ± SD (72h)^a^** | **IC_50_ (nM) ± SD^b^** |
| --- | --- | --- | --- | --- | --- |
| **1** | 1/C14H20N2O3/c17-13(15-12-8-4-3-5-9-12)10-6-1-2-7-11-14(18)16-19/h3-5,8-9,19H,1-2,6-7,10-11H2,(H,15,17)(H,16,18) | >30 | 0.8 ± 0.1 | 0.7 ± 0.4 | 193 ± 41 |
| **7a** | 1/C17H16N2O4/c20-16(19-22)11-8-13-6-9-15(10-7-13)23-12-17(21)18-14-4-2-1-3-5-14/h1-11,22H,12H2,(H,18,21)(H,19,20)/b11-8+ | >30 | 5.0 ± 1.3 | 3.9 ± 0.4 | -- |
| **7b** | 1/C18H18N2O4/c1-13-4-2-3-5-16(13)19-18(22)12-24-15-9-6-14(7-10-15)8-11-17(21)20-23/h2-11,23H,12H2,1H3,(H,19,22)(H,20,21)/b11-8+ | >30 | 4.1 ± 0.7 | 1.8 ± 0.1 | -- |
| **7c** | 1/C18H18N2O4/c1-13-3-2-4-15(11-13)19-18(22)12-24-16-8-5-14(6-9-16)7-10-17(21)20-23/h2-11,23H,12H2,1H3,(H,19,22)(H,20,21)/b10-7+ | 15.3 ± 0.3 | 1.9 ± 0.2 | 1.3 ± 0.1 | -- |
| **7d** | 1/C18H18N2O4/c1-13-2-7-15(8-3-13)19-18(22)12-24-16-9-4-14(5-10-16)6-11-17(21)20-23/h2-11,23H,12H2,1H3,(H,19,22)(H,20,21)/b11-6+ | 15.1 ± 0.1 | 1.6 ± 0.2 | 1.2 ± 0.1 | 174 ± 30 |
| **7e** | 1/C18H18N2O5/c1-24-16-5-3-2-4-15(16)19-18(22)12-25-14-9-6-13(7-10-14)8-11-17(21)20-23/h2-11,23H,12H2,1H3,(H,19,22)(H,20,21)/b11-8+ | 21.7 ± 2.0 | 4.2 ± 1.0 | 3.6 ± 0.2 | -- |
| **7f** | 1/C18H18N2O5/c1-24-16-4-2-3-14(11-16)19-18(22)12-25-15-8-5-13(6-9-15)7-10-17(21)20-23/h2-11,23H,12H2,1H3,(H,19,22)(H,20,21)/b10-7+ | 28.4 ± 2.4 | 3.3 ± 0.5 | 4.8 ± 0.4 | -- |
| **7g** | 1/C18H18N2O5/c1-24-15-9-5-14(6-10-15)19-18(22)12-25-16-7-2-13(3-8-16)4-11-17(21)20-23/h2-11,23H,12H2,1H3,(H,19,22)(H,20,21)/b11-4+ | >30 | 5.6 ± 0.7 | 3.5 ± 1.0 | -- |
| **7h** | 1/C17H15FN2O4/c18-14-3-1-2-4-15(14)19-17(22)11-24-13-8-5-12(6-9-13)7-10-16(21)20-23/h1-10,23H,11H2,(H,19,22)(H,20,21)/b10-7+ | 12.9 ± 1.1 | 3.0 ± 0.4 | 1.8 ± 0.2 | -- |
| **7i** | 1/C17H15FN2O4/c18-13-2-1-3-14(10-13)19-17(22)11-24-15-7-4-12(5-8-15)6-9-16(21)20-23/h1-10,23H,11H2,(H,19,22)(H,20,21)/b9-6+ | 12.5 ± 0.5 | 3.0 ± 0.4 | 1.6 ± 0.2 | -- |
| **7j** | 1/C17H15FN2O4/c18-13-4-6-14(7-5-13)19-17(22)11-24-15-8-1-12(2-9-15)3-10-16(21)20-23/h1-10,23H,11H2,(H,19,22)(H,20,21)/b10-3+ | >30 | 2.9 ± 0.3 | 1.7 ± 0.2 | -- |
| **7k** | 1/C17H15ClN2O4/c18-14-3-1-2-4-15(14)19-17(22)11-24-13-8-5-12(6-9-13)7-10-16(21)20-23/h1-10,23H,11H2,(H,19,22)(H,20,21)/b10-7+ | 8.1 ± 0.3 | 2.9 ± 0.5 | 3.6 ± 0.6 | -- |
| **7l** | 1/C17H15ClN2O4/c18-13-2-1-3-14(10-13)19-17(22)11-24-15-7-4-12(5-8-15)6-9-16(21)20-23/h1-10,23H,11H2,(H,19,22)(H,20,21)/b9-6+ | 7.0 ± 0.2 | 3.0 ± 0.9 | 2.6 ± 0.3 | -- |
| **7m** | 1/C17H15ClN2O4/c18-13-4-6-14(7-5-13)19-17(22)11-24-15-8-1-12(2-9-15)3-10-16(21)20-23/h1-10,23H,11H2,(H,19,22)(H,20,21)/b10-3+ | >30 | 3.5 ± 1.5 | 3.4 ± 0.8 | -- |
| **7n** | 1/C17H15BrN2O4/c18-14-3-1-2-4-15(14)19-17(22)11-24-13-8-5-12(6-9-13)7-10-16(21)20-23/h1-10,23H,11H2,(H,19,22)(H,20,21)/b10-7+ | 11.1 ± 1.4 | 2.2 ± 0.3 | 2.8 ± 0.1 | -- |
| **7o** | 1/C17H15BrN2O4/c18-13-2-1-3-14(10-13)19-17(22)11-24-15-7-4-12(5-8-15)6-9-16(21)20-23/h1-10,23H,11H2,(H,19,22)(H,20,21)/b9-6+ | 11.6 ± 1.5 | 2.7 ± 0.5 | 2.3 ± 0.3 | -- |
| **7p** | 1/C17H15BrN2O4/c18-13-4-6-14(7-5-13)19-17(22)11-24-15-8-1-12(2-9-15)3-10-16(21)20-23/h1-10,23H,11H2,(H,19,22)(H,20,21)/b10-3+ | 9.4 ± 1.4 | 2.2 ± 0.4 | 1.6 ± 0.2 | 157 ± 35 |
| **7q** | 1/C18H15F3N2O4/c19-18(20,21)14-3-1-2-4-15(14)22-17(25)11-27-13-8-5-12(6-9-13)7-10-16(24)23-26/h1-10,26H,11H2,(H,22,25)(H,23,24)/b10-7+ | 9.8 ± 0.2 | 2.9 ± 0.4 | 2.5 ± 0.1 | -- |
| **7r** | 1/C18H15F3N2O4/c19-18(20,21)13-2-1-3-14(10-13)22-17(25)11-27-15-7-4-12(5-8-15)6-9-16(24)23-26/h1-10,26H,11H2,(H,22,25)(H,23,24)/b9-6+ | 22.2 ± 1.8 | 7.5 ± 0.5 | 6.4 ± 0.5 | -- |
| **7s** | 1/C18H15F3N2O4/c19-18(20,21)13-4-6-14(7-5-13)22-17(25)11-27-15-8-1-12(2-9-15)3-10-16(24)23-26/h1-10,26H,11H2,(H,22,25)(H,23,24)/b10-3+ | 10.1 ± 1.1 | 2.8 ± 0.2 | 2.7 ± 0.2 | -- |
| **7t** | 1/C17H15N3O6/c21-16(19-23)10-7-12-5-8-13(9-6-12)26-11-17(22)18-14-3-1-2-4-15(14)20(24)25/h1-10,23H,11H2,(H,18,22)(H,19,21)/b10-7+ | >30 | 5.6 ± 0.4 | 2.9 ± 0.4 | -- |
| **7u** | 1/C17H15N3O6/c21-16(19-23)9-6-12-4-7-15(8-5-12)26-11-17(22)18-13-2-1-3-14(10-13)20(24)25/h1-10,23H,11H2,(H,18,22)(H,19,21)/b9-6+ | >30 | 2.5 ± 0.3 | 2.0 ± 0.1 | -- |
| **7v** | 1/C17H15N3O6/c21-16(19-23)10-3-12-1-8-15(9-2-12)26-11-17(22)18-13-4-6-14(7-5-13)20(24)25/h1-10,23H,11H2,(H,18,22)(H,19,21)/b10-3+ | >30 | 9.9 ± 0.5 | 3.5 ± 0.5 | -- |

^a^ Effect of hydroxamic acid derivatives on proliferation of THP-1 cell line expressed as IC_50_ values. Cell proliferation was evaluated using WST-1 analysis after 24, 48, or 72 h of incubation with serial dilutions of the compounds tested. Values shown are the mean ± SD of three independent experiments, each of which was performed in triplicate. Data are presented in this manuscript.

^b^ The inhibitory effects of vorinostat 1, 7d, and 7p on the enzyme activity of HDAC class I and II in THP-1 cell line were determined using the HDAC-Glo™ I/II Assay. The results are expressed as the mean ± SD of three independent experiments. Data are presented in this manuscript.
