# Supplementary material for: New potent N‐hydroxycinnamamide‐based histone deacetylase inhibitors suppress proliferation and trigger apoptosis in THP‐1 leukaemia cells
Source: Arch Pharm (Weinheim). 2025 Apr 1;358(4):e2400889. doi: 10.1002/ardp.202400889 (PMC11959351; doi:10.1002/ardp.202400889)
Supplement: Supplementary file 2 — Supporting information. [file ARDP-358-e2400889-s002.pdf]

Supporting Information

New group of hydroxamic acid derivatives inhibits cell growth, affects cell cycle progression, and induces apoptosis in THP-1 monocytic leukemia cells

Magdaléna Onuščáková[1‡], Tereza Kauerová[2‡], Eva Fialová[1], Hana Pížová[1], Vladimír Garaj[3], Miroslav Kemka[3], Vladimir Frecer[4], Peter Kollar[2]*, Pavel Bobal[1]*

**corresponding authors:*

bobalp@pharm.muni.cz, kollarp@pharm.muni.cz

[1]Department of Chemical Drugs, Faculty of Pharmacy, Masaryk University, Palackeho tr. 1946/1, 612 00 Brno, Czech Republic

[2]Department of Pharmacology and Toxicology, Faculty of Pharmacy, Masaryk University, Palackeho tr. 1946/1, 612 00 Brno, Czech Republic

[3]Department of Pharmaceutical Chemistry, Faculty of Pharmacy, Comenius University Bratislava, Odbojarov 10, 832 32 Bratislava, Slovakia

[4]Department of Physical Chemistry of Drugs, Faculty of Pharmacy, Comenius University Bratislava, Odbojarov 10, 832 32 Bratislava, Slovakia

Table of Contents

General Information ……………………………………………………………………………………….S2

NMR, HRMS Spectra, and HPLC Purities of Products ....…………………………………………..…….S3

In Silico Modeling and Structural Studies ..………………………………..………………………….……...S92

Analysis of Subdiploid Cell Population ……………………...……………………………………….…S94

ADMET-related properties…………………………………………………………………………….....S95

General information

The reagents and solvents utilized for synthesis were procured from Sigma-Aldrich and employed as received unless otherwise specified. Anhydrous reagents and solvents were absolutized as usual and distilled prior to use.

^{1}H-NMR spectra were obtained on a JEOL ECZR-400 MHz instrument (Jeol Corp.). All NMR measurements were done at 25 °C. Chemical shifts δ are reported in parts per million (ppm) and *J* values in Hz. NMR spectra were acquired in DMSO-d_6. The residual solvent signal of DMSO-d_6 was used for reference. Norell StandartSeries^{TM} 5 mm NMR tubes were utilized.

MS analysis was performed on an Impact II (Q-TOF, Bruker Daltonics) or LTQ Orbitrap XL (Thermo Fisher Scientific) high-resolution mass spectrometers. Melting points were measured using a Böetius apparatus (Franz Küstner Nachf. KG) and were not corrected. Mass spectra were obtained using electrospray ionization (ESI) in the positive or negative ion mode.

Merck Kieselgel 60 silica gel (70-230 mesh particle size) was used for column chromatography. All fractions were monitored by silica gel thin-layer chromatography (TLC) plates (225 μm thickness, 60 A silica gel medium) with the detection of compounds with UV light. A Dionex Ultimate 3000 (Thermo Scientific) HPLC system controlled with the Chromeleon® Chromatography Data System (version 7.2, Thermo Scientific, Waltham, MA USA) was used to analyze the final compounds' purity. The separation was performed on a YMC-Triart C18 (3 μm, 150 mm × 2 mm) column (Agilent Technologies, Waldbronn, Germany). The mobile phase consisted of a mixture of acetonitrile and water containing 0.1 % formic acid in a ratio from 40:60 to 90:10. The total flow rate was set at 0.2 mL/min; the injection volume was 1 μL; and the column temperature was maintained at 30 °C. The detection wavelength of 210 nm was chosen. The purity of each compound was determined by calculating the average of relative peak areas in the sample solution chromatograms. All compounds are >95% pure by HPLC analysis.

The analysis of the percentage of subdiploid cell population was performed as a part of the evaluation of cell cycle distribution. THP-1 cells were treated and subsequently incubated with indicated concentrations of **1**, **7d**, and **7p** for 48 h. Cells were collected and washed twice with 1 × PBS. After fixation 70% ethanol samples were stored at −20 °C overnight. Cells were then harvested by centrifugation, and the cell pellet was washed twice with 1 × PBS followed by incubation with RNaseA (0.02 mg/mL) and 0.05% (v/v) Triton X-100 in 1 × PBS for 30 min at 37 °C. Before flow cytometric analysis, the nuclei were stained with propidium iodide (PI) (0.04 mg/mL). The percentage of subdiploid cell population was analyzed using a flow cytometer BriCyte-E6 (Mindray, Shenzhen, China), and its quantification was carried out using the software Kaluza Flow Cytometry Analysis Software 1.2 (Beckman Coulter). A total number of 2×10^4 cells were analyzed per sample.

NMR, HRMS Spectra, and HPLC Purities of Products

(2*E*)-*N*-Hydroxy-3-{4-[(phenylcarbamoyl)methoxy]phenyl}prop-2-enamide (**7a**)

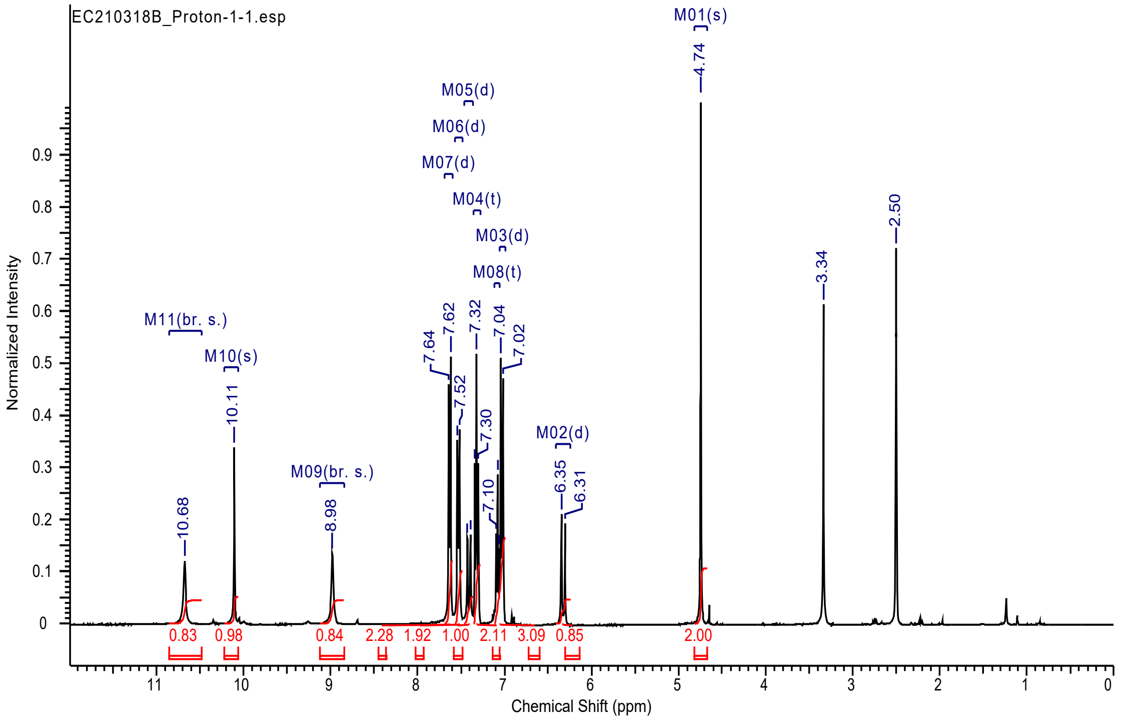

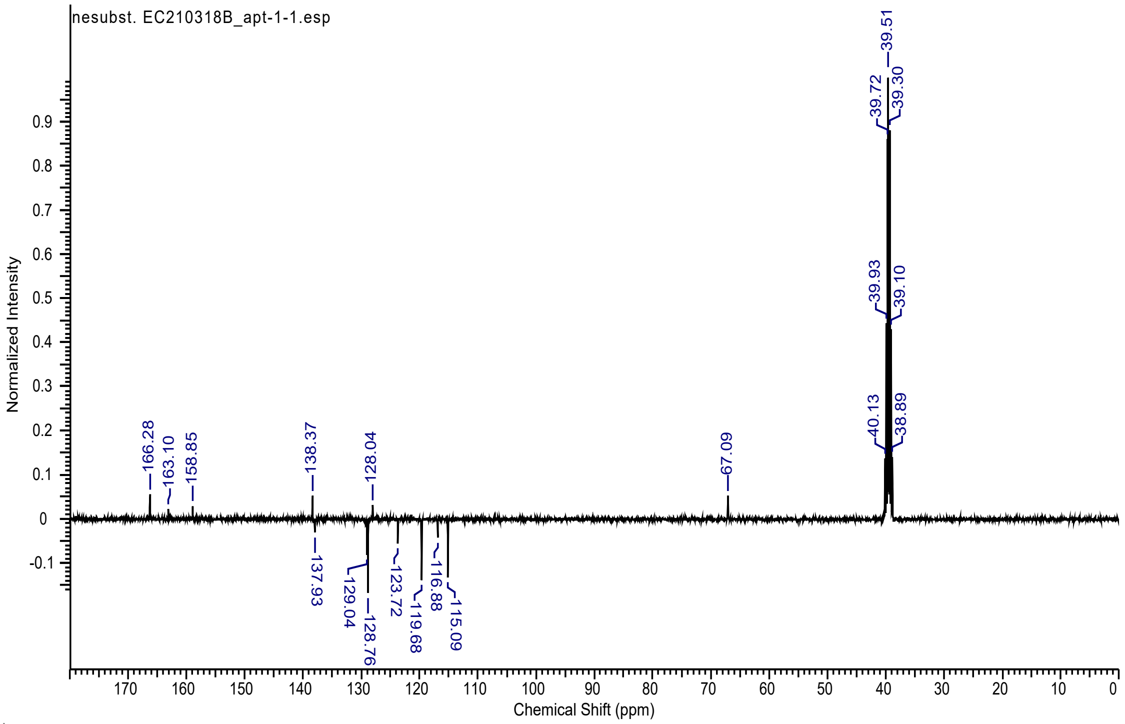

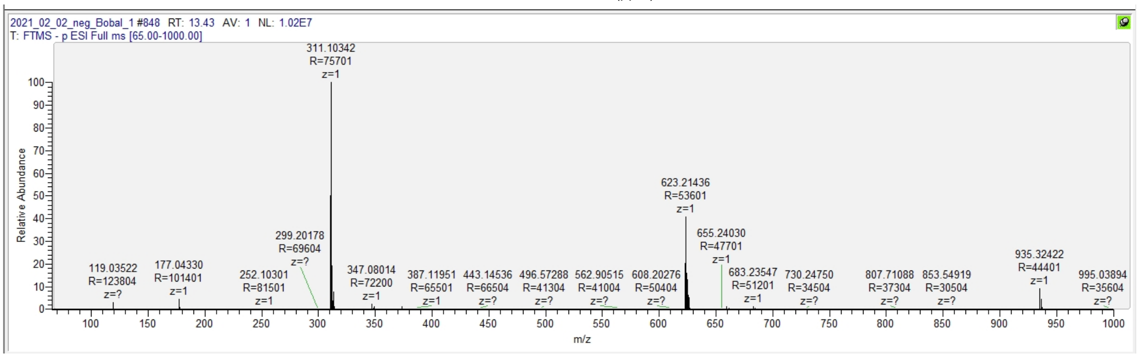

Chromatogram and Results

Injection Details

Injection Name:	**MO nesub. HA**	*Run Time (min):*	**30,00**
Vial Number:	**BA2**	*Injection Volume:*	**10,00**
Injection Type:	**Unknown**	*Channel:*	**UV_VIS_1**
Calibration Level:		*Wavelength:*	**210,0**
Instrument Method:	**Grad40-60to90-10 MeCN-H2O**	*Bandwidth:*	**2**
Processing Method:	**New Processing Method**	*Dilution Factor:*	**1,0000**
Injection Date/Time:	**04.1.22 12:18**	*Sample Weight:*	**1,0000**

Chromatogram

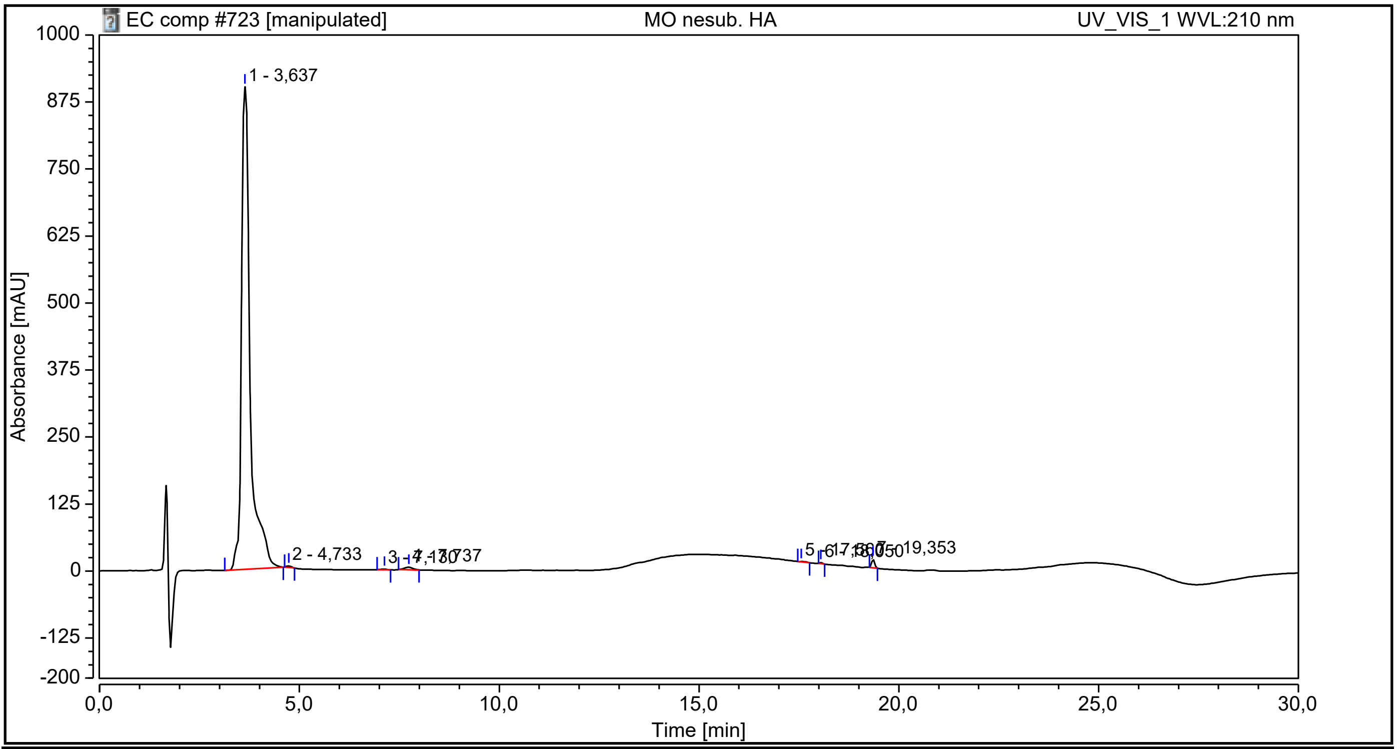

Integration Results

No.	Peak Name	Retention Time min	Area mAU*min	Height mAU	Relative Area %	Relative Height %	Amount n.a.
1		3,637	229,095	900,011	98,486	96,92	n.a.
2		4,733	0,398	2,888	0,171	0,31	n.a.
3		7,130	0,226	1,405	0,097	0,15	n.a.
4		7,737	1,171	5,023	0,504	0,54	n.a.
5		17,560	0,174	0,844	0,075	0,09	n.a.
6		18,050	0,164	2,009	0,071	0,22	n.a.
7		19,353	1,388	16,395	0,597	1,77	n.a.
**Total:**			**232,617**	**928,574**	**100,00**	**100,00**	

(2*E*)-*N*-Hydroxy-3-(4-{[(2-methylphenyl)carbamoyl]methoxy}phenyl)prop-2-enamide (**7b**)

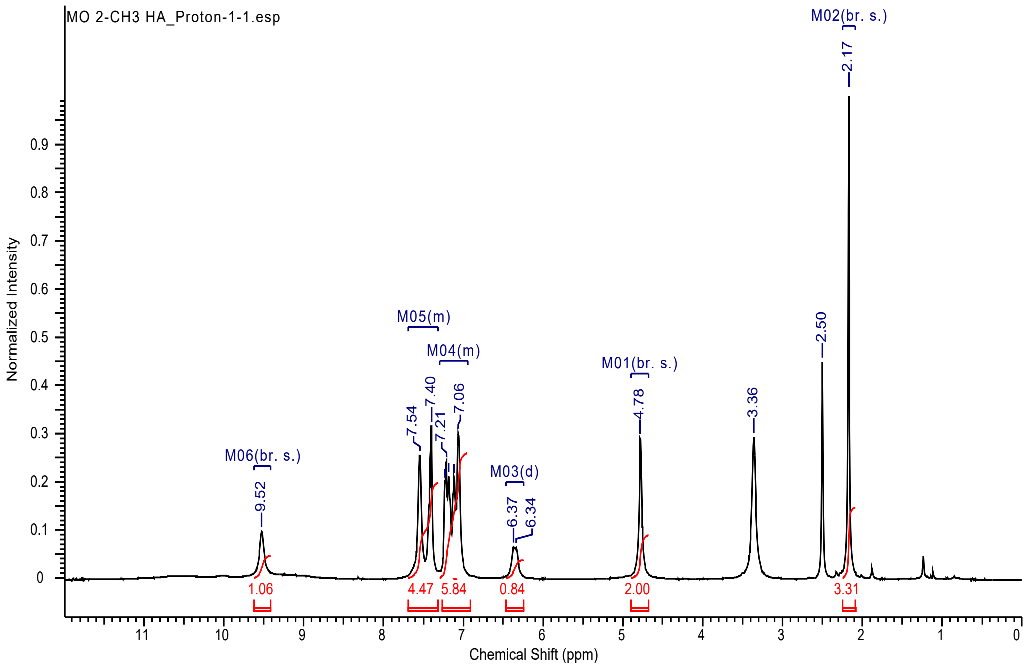

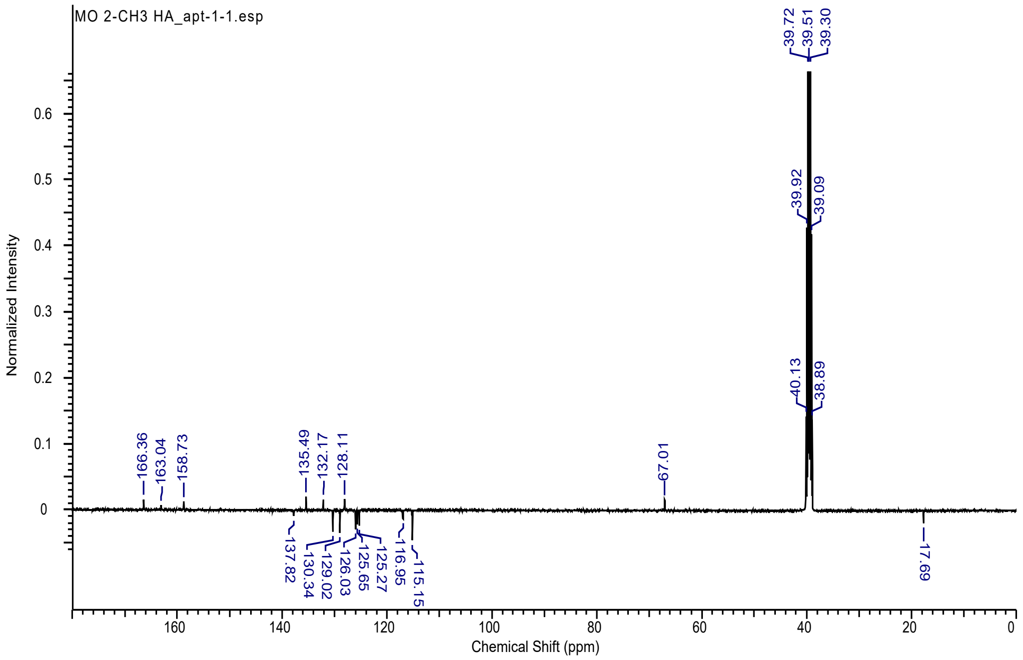

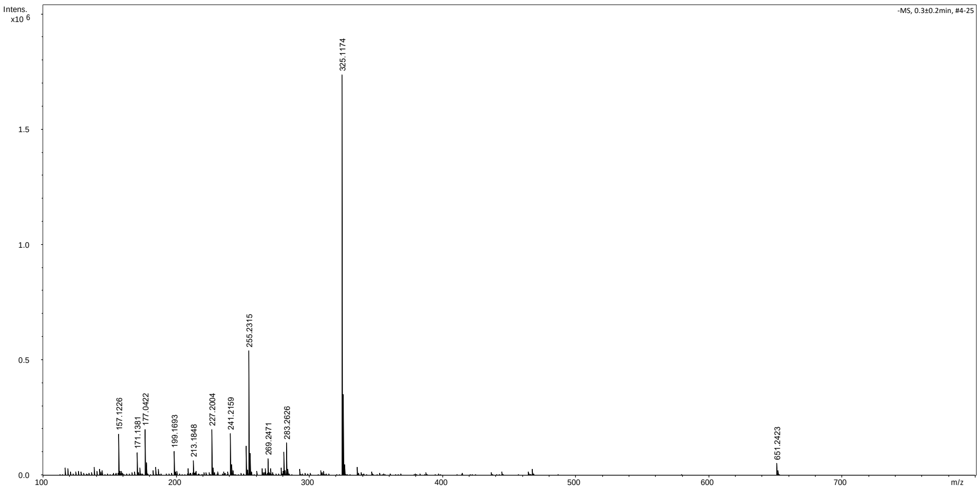

Chromatogram and Results

Injection Details

Injection Name:	**MO 2-CH3 HA**	*Run Time (min):*	**30,00**
Vial Number:	**BA6**	*Injection Volume:*	**10,00**
Injection Type:	**Unknown**	*Channel:*	**UV_VIS_1**
Calibration Level:		*Wavelength:*	**210,0**
Instrument Method:	**Grad40-60to90-10 MeCN-H2O**	*Bandwidth:*	**2**
Processing Method:	**New Processing Method**	*Dilution Factor:*	**1,0000**
Injection Date/Time:	**05.1.22 09:31**	*Sample Weight:*	**1,0000**

Chromatogram

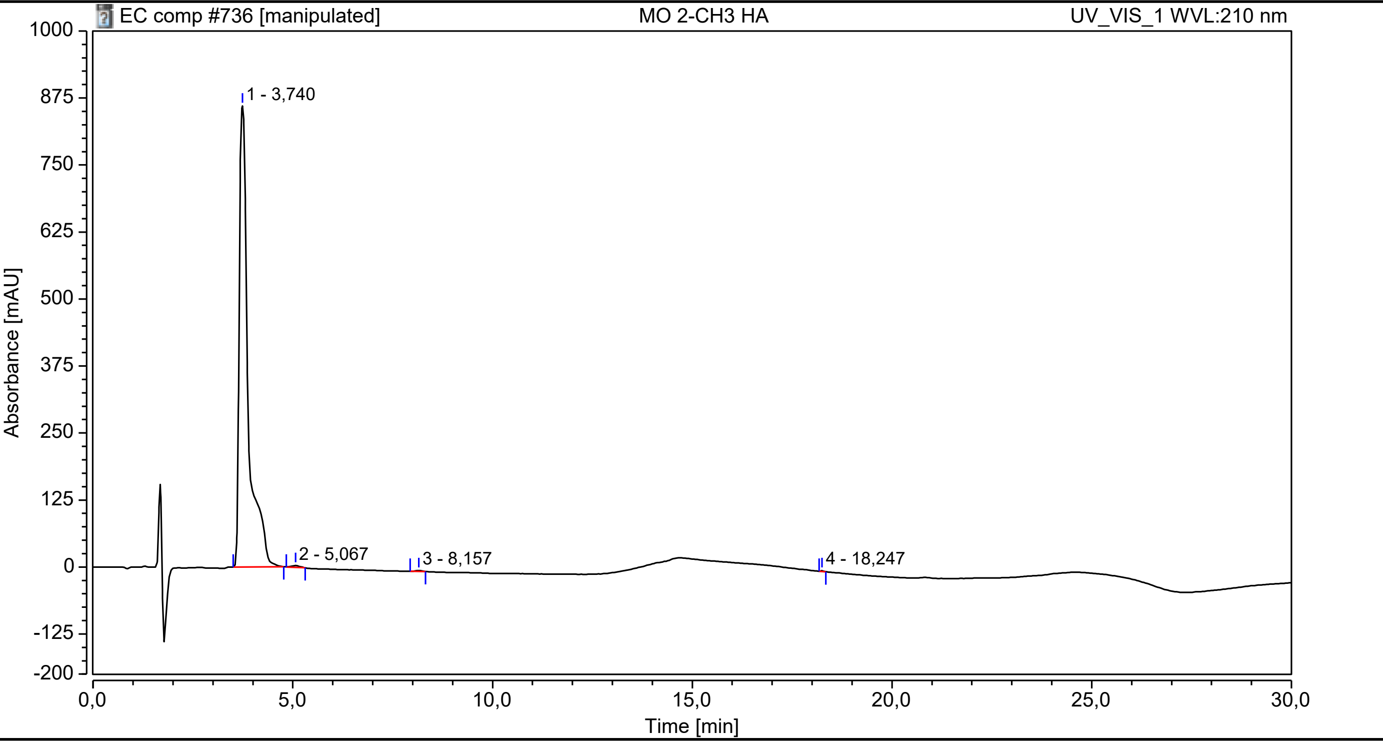

Integration Results

No.	Peak Name	Retention Time min	Area mAU*min	Height mAU	Relative Area %	Relative Height %	Amount n.a.
1	n.a.	3,740	225,691	860,497	99,465	99,28	n.a.
2	n.a.	5,067	0,781	3,195	0,344	0,37	n.a.
3	n.a.	8,157	0,320	1,570	0,141	0,18	n.a.
4	n.a.	18,247	0,112	1,500	0,049	0,17	n.a.
**Total:**			**226,904**	**866,763**	**100,00**	**100,00**	

(2*E*)-*N*-Hydroxy-3-(4-{[(3-methylphenyl)carbamoyl]methoxy}phenyl)prop-2-enamide (**7c**)

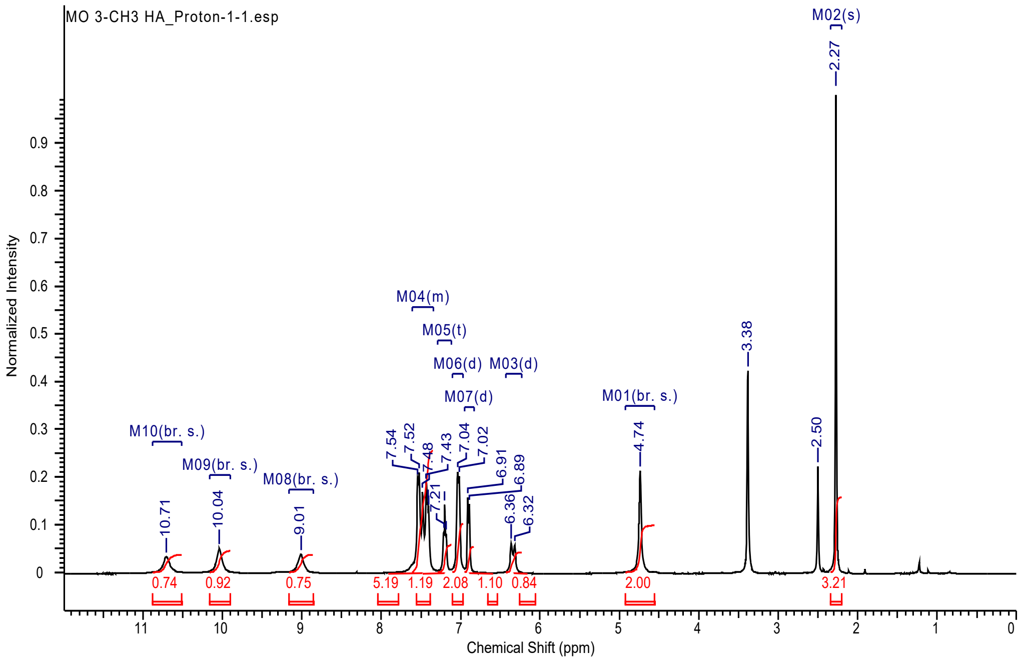

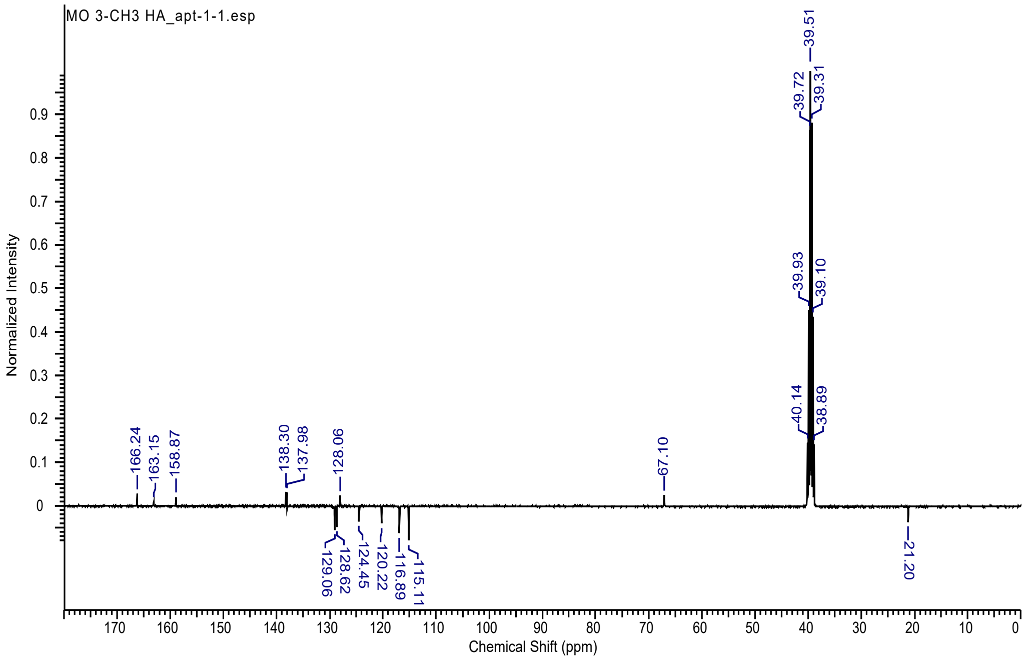

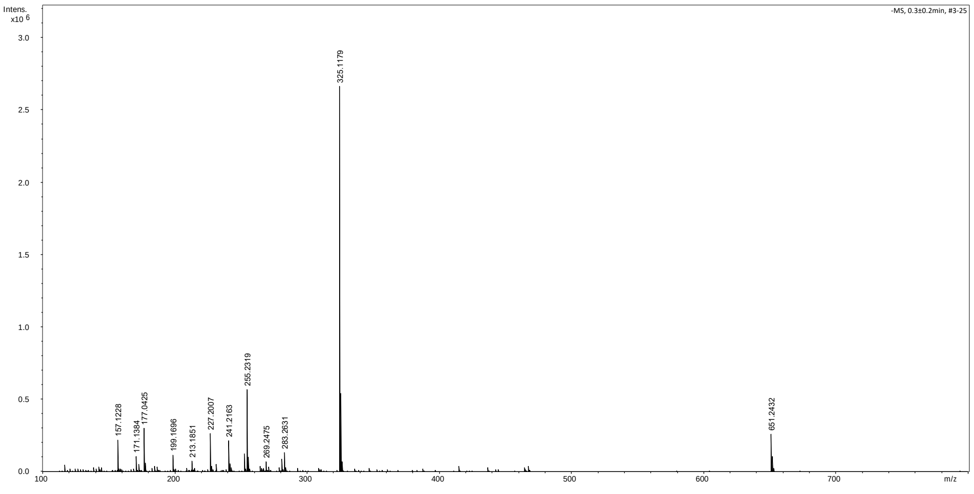

Chromatogram and Results

Injection Details

Injection Name:	**MO 3-CH3 HA**	*Run Time (min):*	**30,00**
Vial Number:	**BA7**	*Injection Volume:*	**10,00**
Injection Type:	**Unknown**	*Channel:*	**UV_VIS_1**
Calibration Level:		*Wavelength:*	**210,0**
Instrument Method:	**Grad40-60to90-10 MeCN-H2O**	*Bandwidth:*	**2**
Processing Method:	**New Processing Method**	*Dilution Factor:*	**1,0000**
Injection Date/Time:	**05.1.22 11:37**	*Sample Weight:*	**1,0000**

Chromatogram

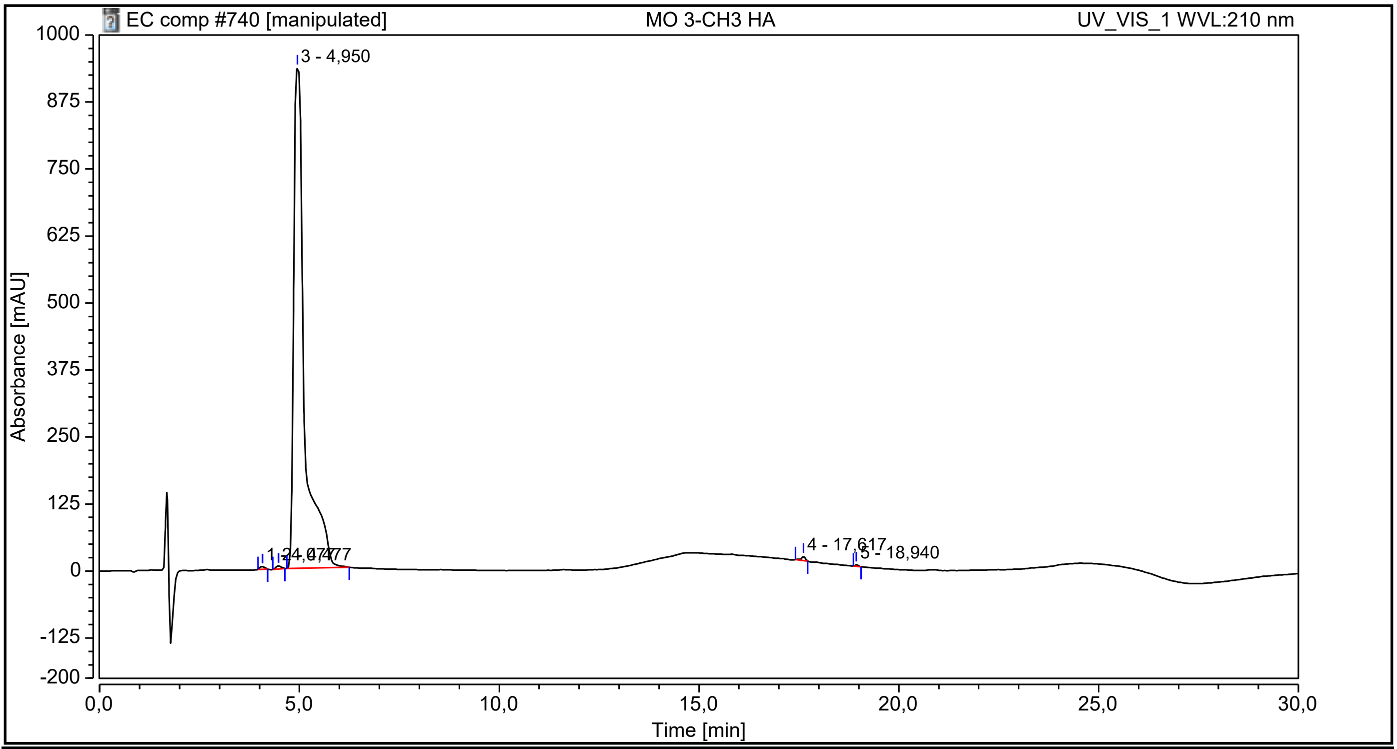

Integration Results

No.	Peak Name	Retention Time min	Area mAU*min	Height mAU	Relative Area %	Relative Height %	Amount n.a.
1	n.a.	4,077	0,734	5,364	0,249	0,56	n.a.
2	n.a.	4,477	0,859	5,405	0,291	0,57	n.a.
3	n.a.	4,950	292,022	933,730	99,048	97,68	n.a.
4	n.a.	17,617	0,968	8,427	0,328	0,88	n.a.
5	n.a.	18,940	0,245	2,979	0,083	0,31	n.a.
**Total:**			**294,828**	**955,906**	**100,00**	**100,00**	

(2*E*)-*N*-Hydroxy-3-(4-{[(4-methylphenyl)carbamoyl]methoxy}phenyl)prop-2-enamide (**7d**)

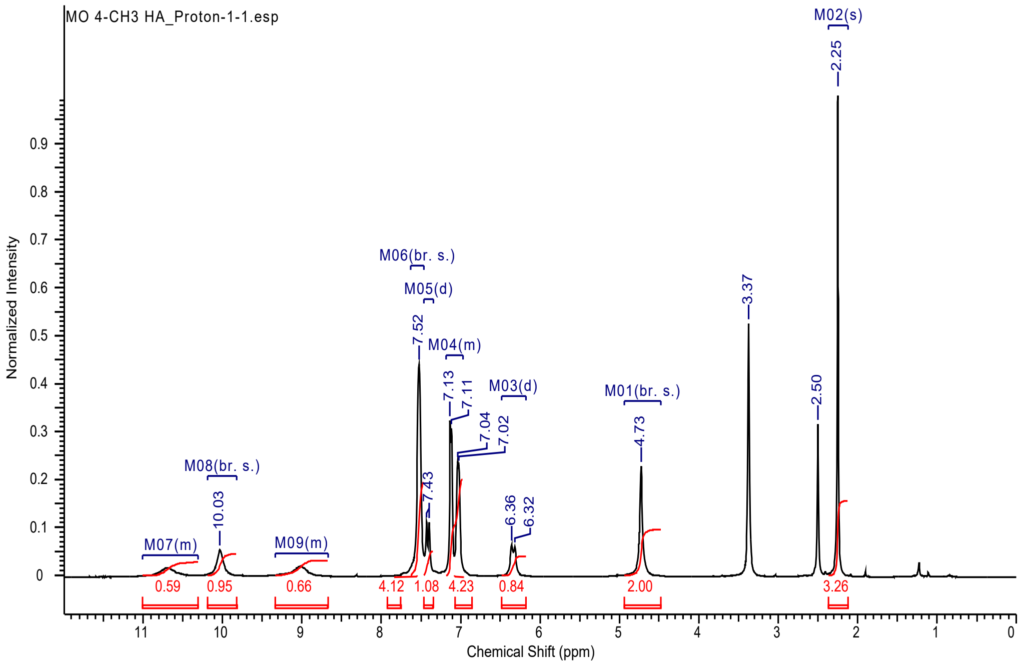

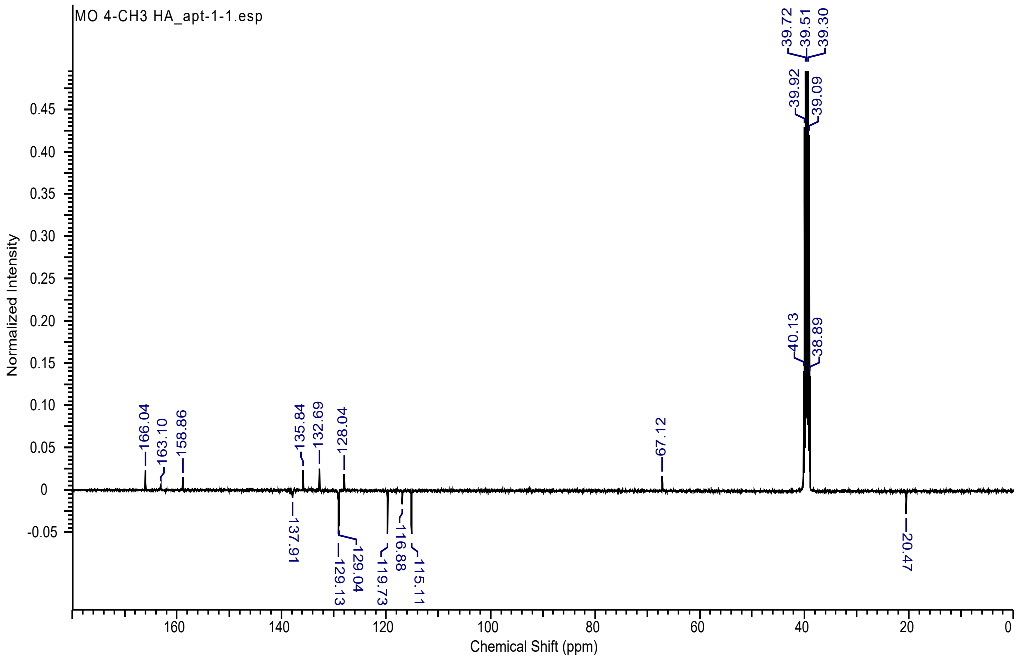

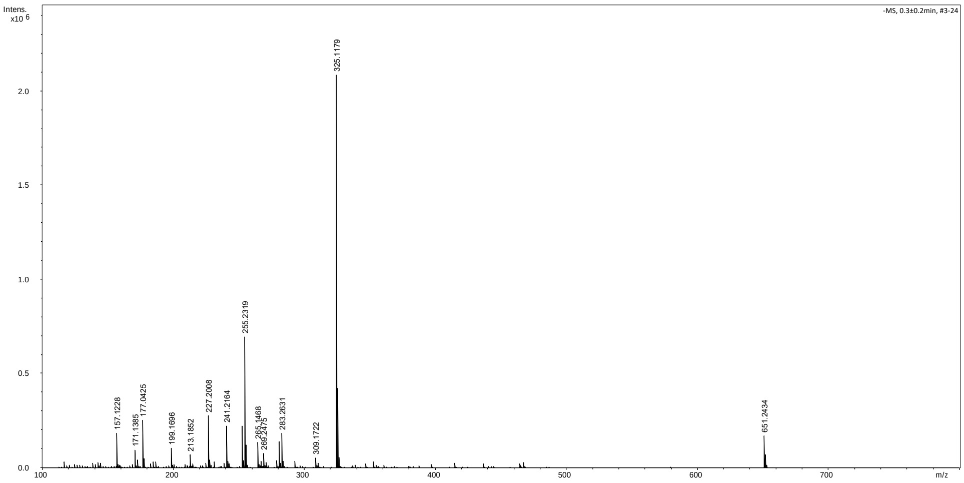

Chromatogram and Results

Injection Details

Injection Name:	**MO 4-CH3 HA**	*Run Time (min):*	**30,00**
Vial Number:	**BA8**	*Injection Volume:*	**10,00**
Injection Type:	**Unknown**	*Channel:*	**UV_VIS_1**
Calibration Level:		*Wavelength:*	**210,0**
Instrument Method:	**Grad40-60to90-10 MeCN-H2O**	*Bandwidth:*	**2**
Processing Method:	**New Processing Method**	*Dilution Factor:*	**1,0000**
Injection Date/Time:	**05.1.22 12:08**	*Sample Weight:*	**1,0000**

Chromatogram

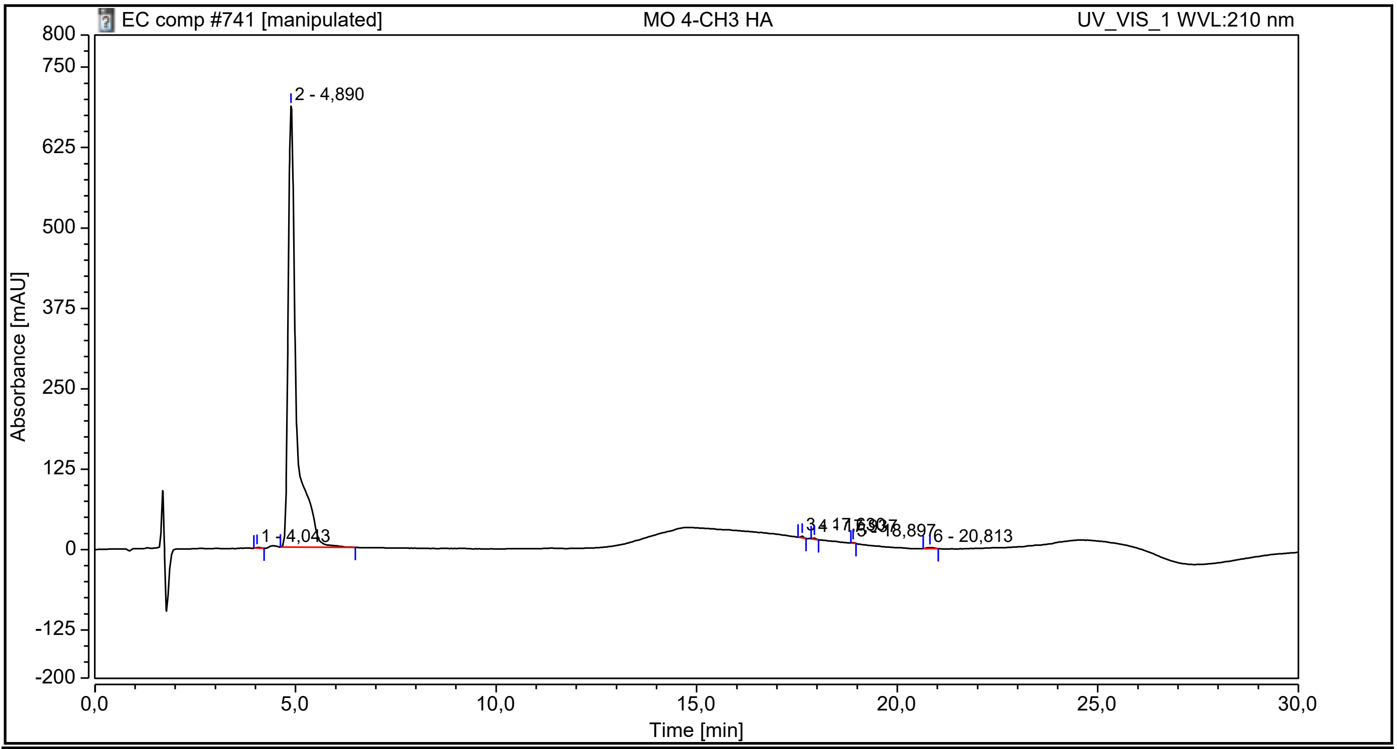

Integration Results

No.	Peak Name	Retention Time min	Area mAU*min	Height mAU	Relative Area %	Relative Height %	Amount n.a.
1	n.a.	4,043	0,129	1,036	0,079	0,15	n.a.
2	n.a.	4,890	162,736	685,529	99,422	98,84	n.a.
3	n.a.	17,630	0,193	2,385	0,118	0,34	n.a.
4	n.a.	17,937	0,169	1,943	0,103	0,28	n.a.
5	n.a.	18,897	0,072	1,060	0,044	0,15	n.a.
6	n.a.	20,813	0,383	1,644	0,234	0,24	n.a.
**Total:**			**163,683**	**693,598**	**100,00**	**100,00**	

(2*E*)-*N*-Hydroxy-3-(4-{[(2-methoxyphenyl)carbamoyl]methoxy}phenyl)prop-2-enamide (**7e**)

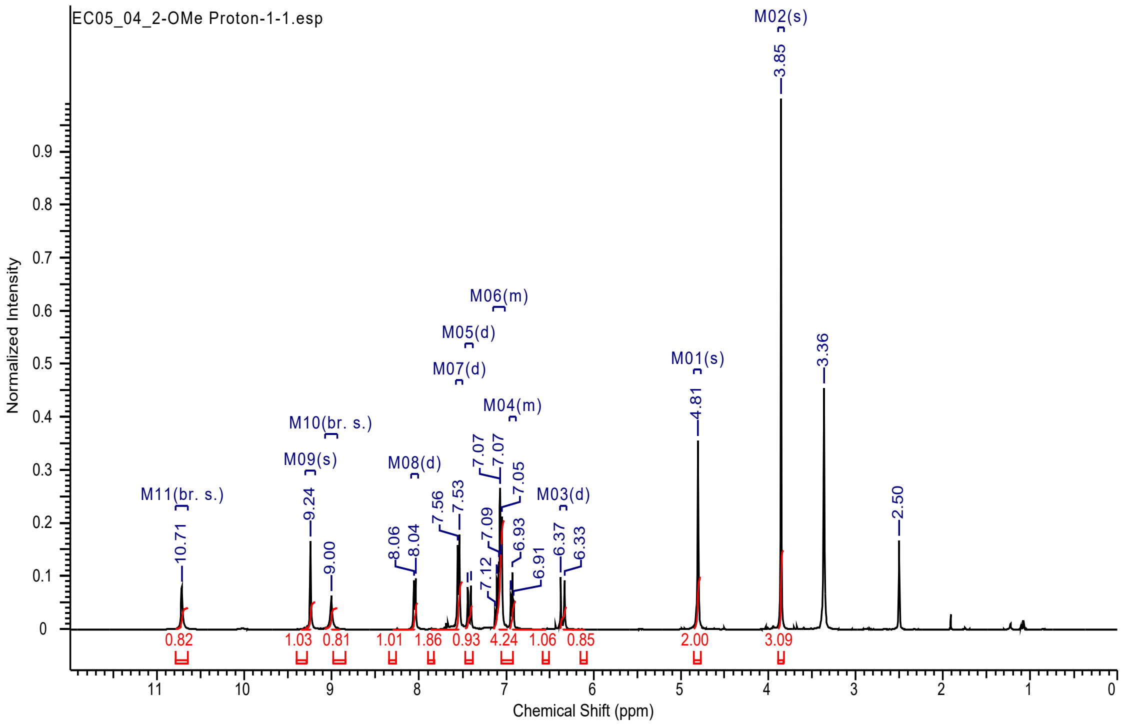

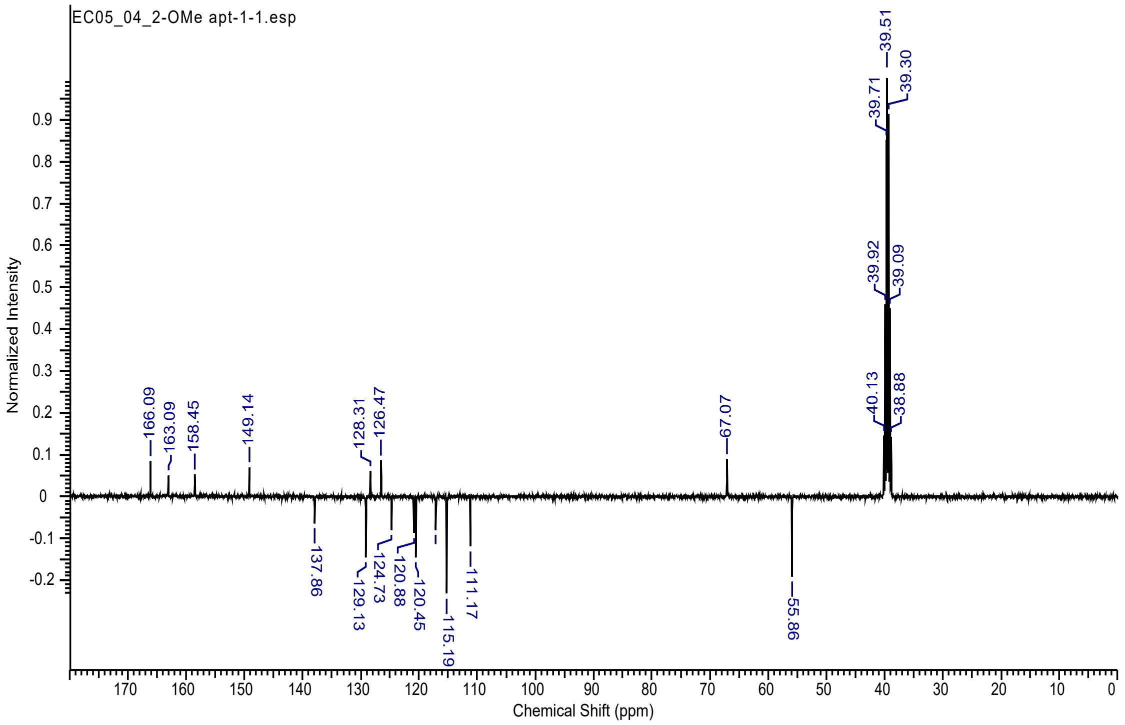

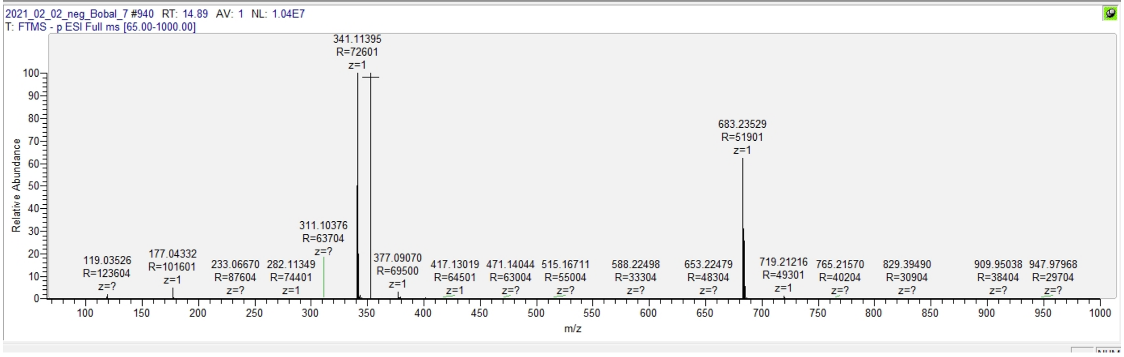

Chromatogram and Results

Injection Details

Injection Name:	**MO 2-OCH3 HA**	*Run Time (min):*	**30,00**
Vial Number:	**BC2**	*Injection Volume:*	**10,00**
Injection Type:	**Unknown**	*Channel:*	**UV_VIS_1**
Calibration Level:		*Wavelength:*	**210,0**
Instrument Method:	**Grad40-60to90-10 MeCN-H2O**	*Bandwidth:*	**2**
Processing Method:	**New Processing Method**	*Dilution Factor:*	**1,0000**
Injection Date/Time:	**06.1.22 16:21**	*Sample Weight:*	**1,0000**

Chromatogram

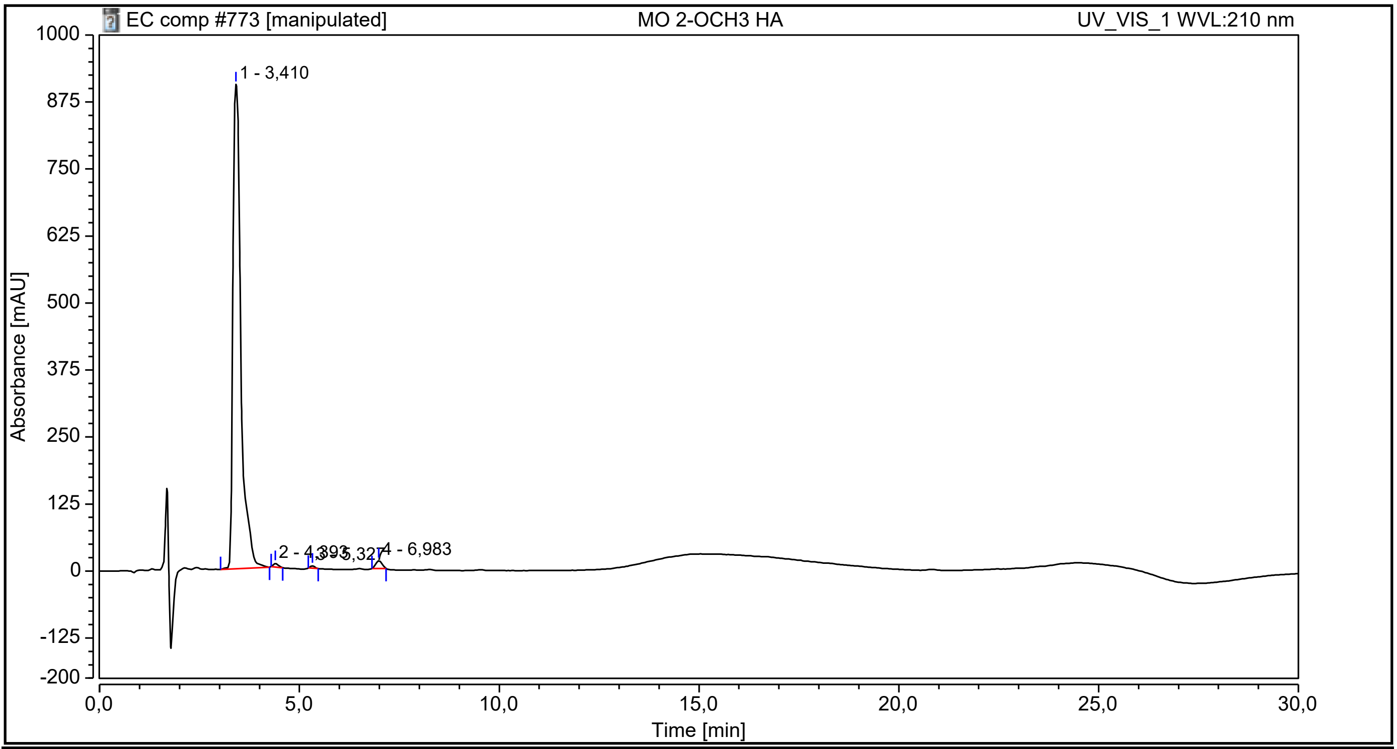

Integration Results

No.	Peak Name	Retention Time min	Area mAU*min	Height mAU	Relative Area %	Relative Height %	Amount n.a.
1	n.a.	3,410	209,130	903,380	98,028	97,26	n.a.
2	n.a.	4,393	1,004	6,656	0,471	0,72	n.a.
3	n.a.	5,327	0,585	4,347	0,274	0,47	n.a.
4	n.a.	6,983	2,618	14,415	1,227	1,55	n.a.
**Total:**			**213,336**	**928,798**	**100,00**	**100,00**	

(2*E*)-*N*-Hydroxy-3-(4-{[(3-methoxyphenyl)carbamoyl]methoxy}phenyl)prop-2-enamide (**7f**)

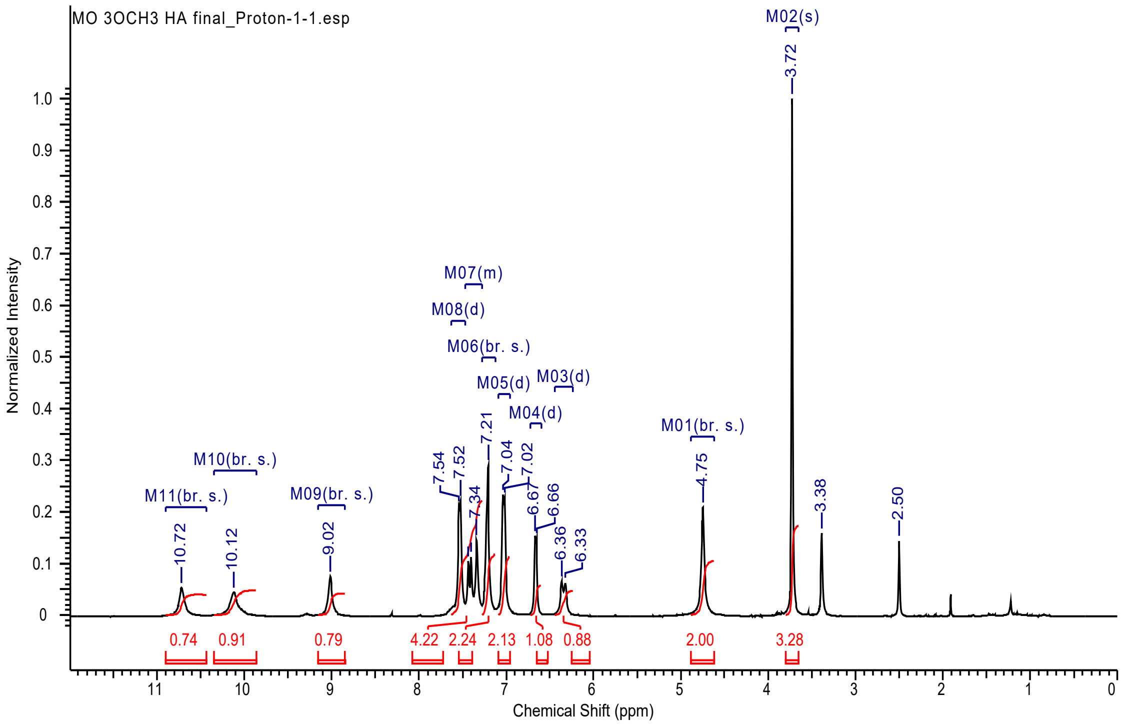

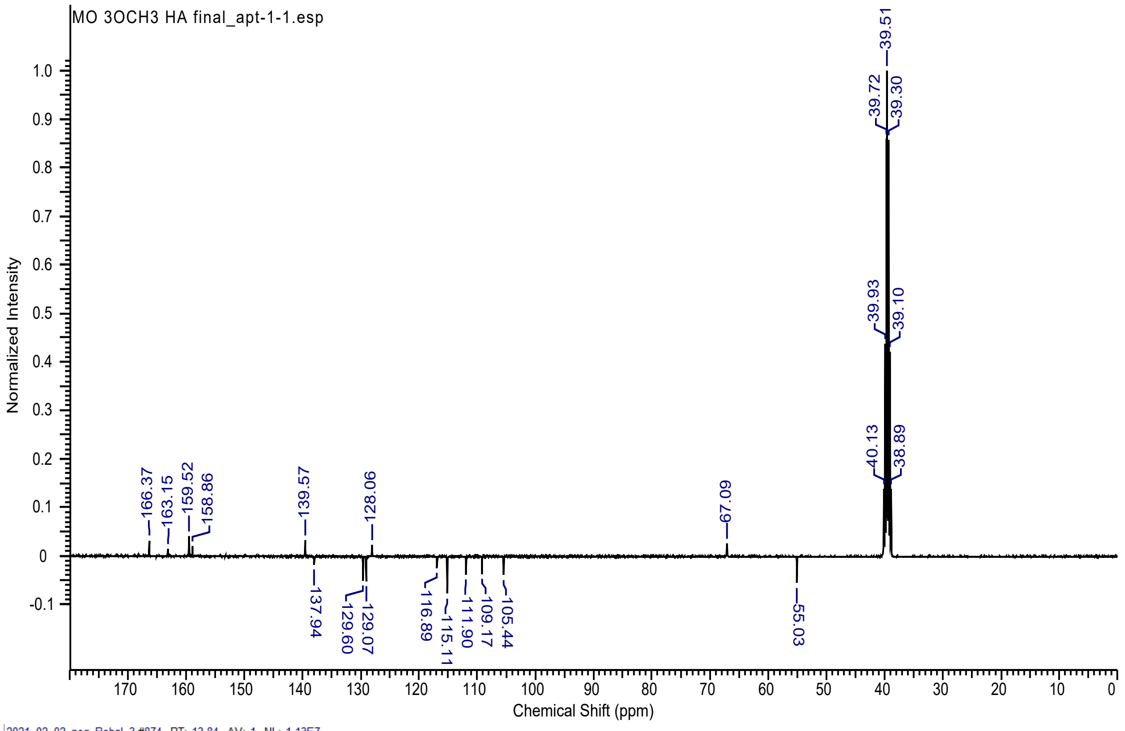

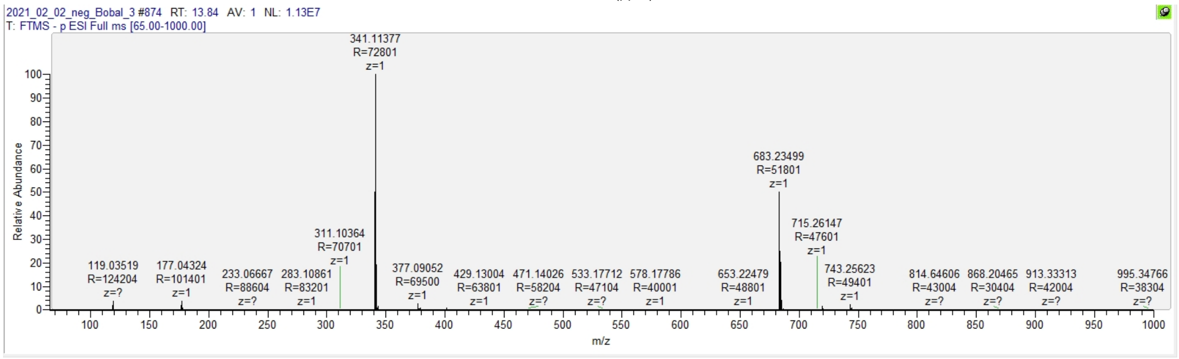

Chromatogram and Results

Injection Details

Injection Name:	**MO 3-OCH3 HA**	*Run Time (min):*	**30,00**
Vial Number:	**BC3**	*Injection Volume:*	**10,00**
Injection Type:	**Unknown**	*Channel:*	**UV_VIS_1**
Calibration Level:		*Wavelength:*	**210,0**
Instrument Method:	**Grad40-60to90-10 MeCN-H2O**	*Bandwidth:*	**2**
Processing Method:	**New Processing Method**	*Dilution Factor:*	**1,0000**
Injection Date/Time:	**06.1.22 16:52**	*Sample Weight:*	**1,0000**

Chromatogram

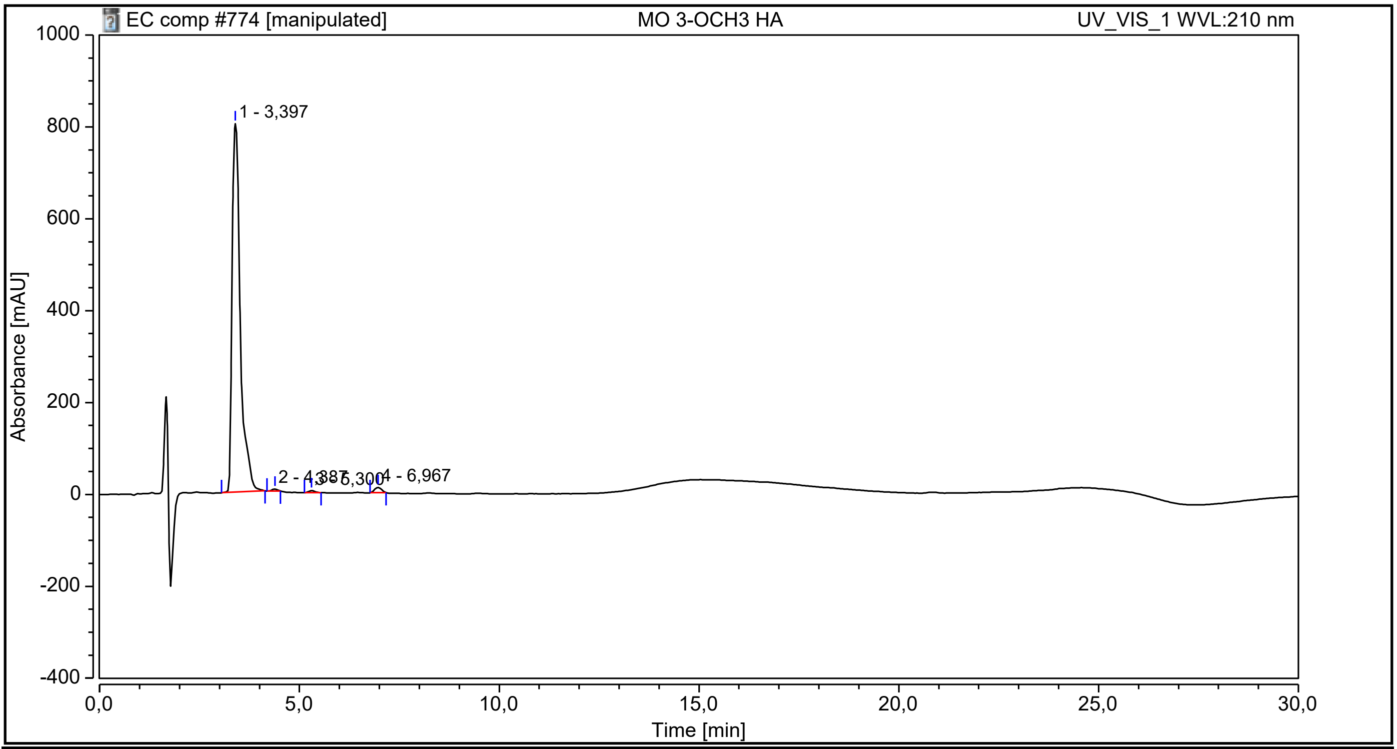

Integration Results

No.	Peak Name	Retention Time min	Area mAU*min	Height mAU	Relative Area %	Relative Height %	Amount n.a.
1	n.a.	3,397	190,212	801,734	98,040	97,55	n.a.
2	n.a.	4,387	0,655	4,367	0,338	0,53	n.a.
3	n.a.	5,300	0,814	4,332	0,420	0,53	n.a.
4	n.a.	6,967	2,334	11,405	1,203	1,39	n.a.
**Total:**			**194,015**	**821,838**	**100,00**	**100,00**	

(2*E*)-*N*-Hydroxy-3-(4-{[(4-methoxyphenyl)carbamoyl]methoxy}phenyl)prop-2-enamide (**7g**)

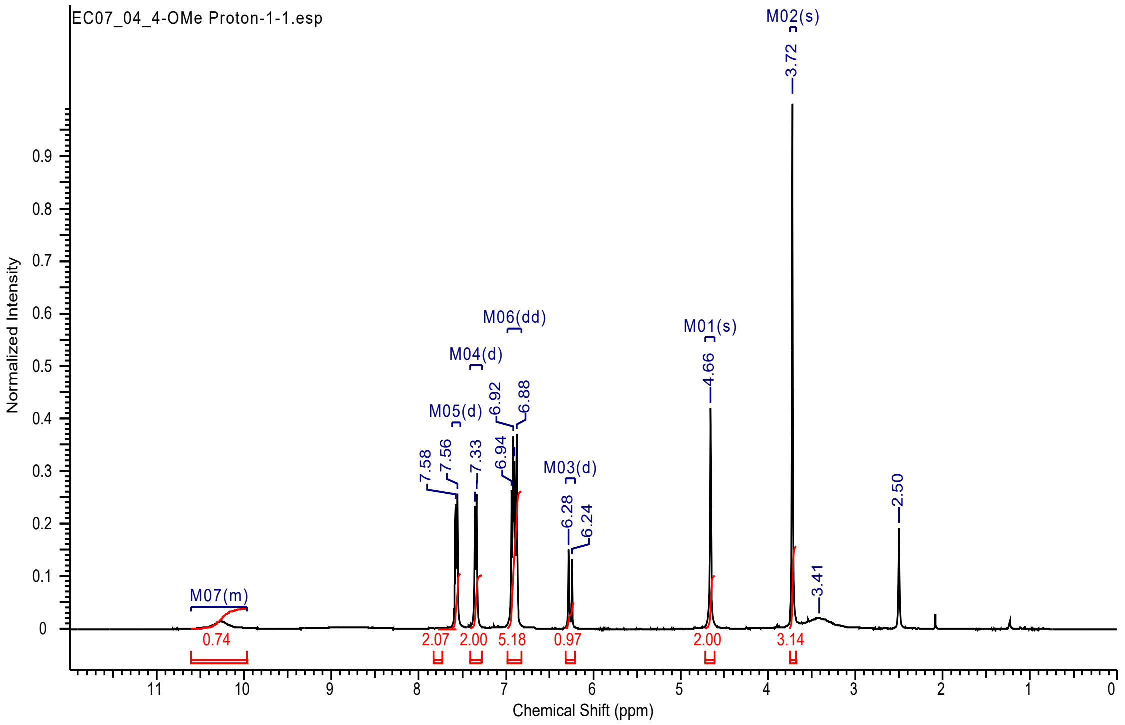

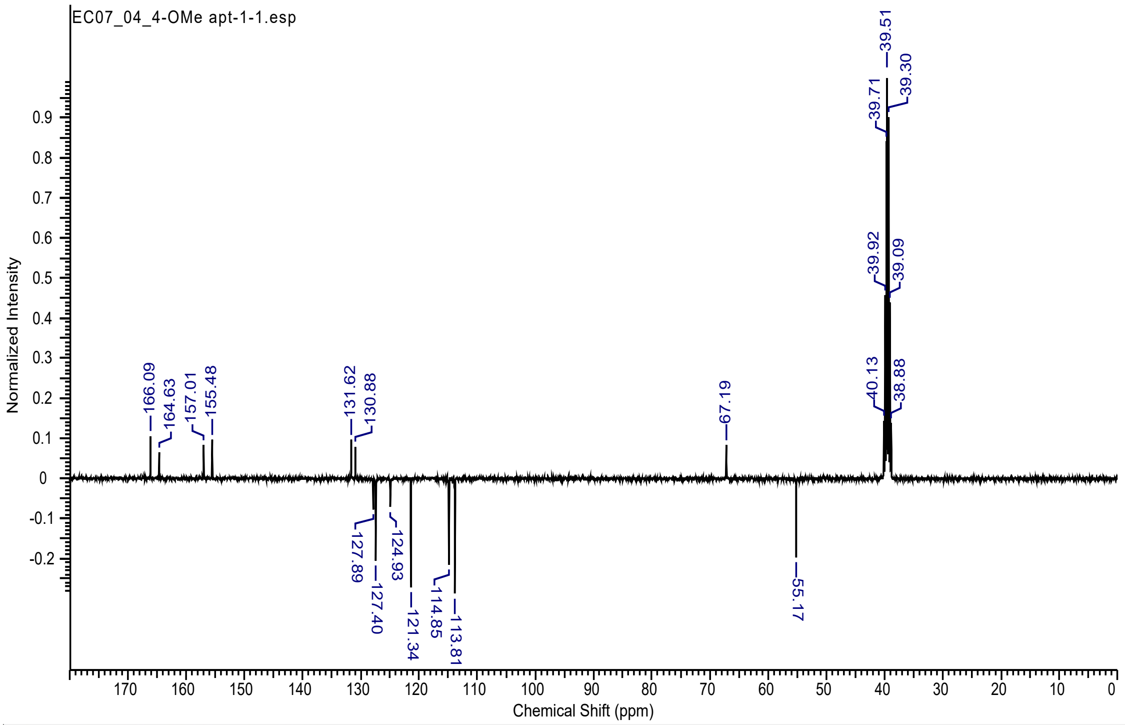

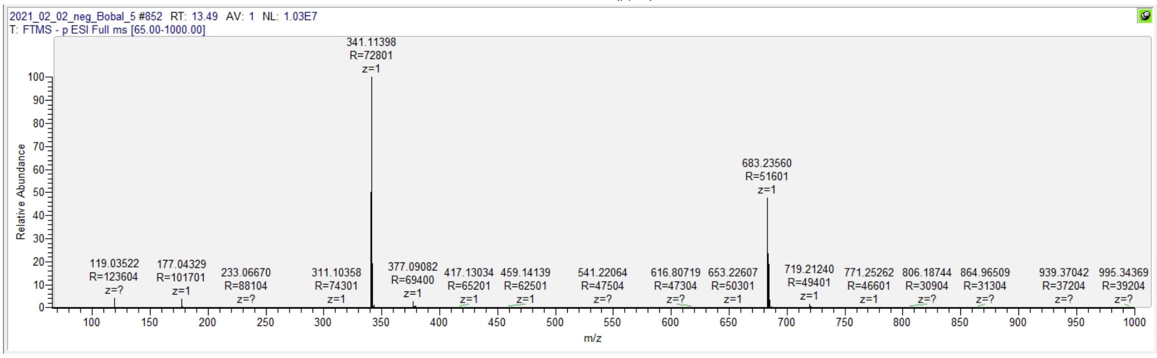

Chromatogram and Results

Injection Details

Injection Name:	**MO 4-OCH3 HA**	*Run Time (min):*	**30,00**
Vial Number:	**BD7**	*Injection Volume:*	**10,00**
Injection Type:	**Unknown**	*Channel:*	**UV_VIS_1**
Calibration Level:		*Wavelength:*	**210,0**
Instrument Method:	**Grad40-60to90-10 MeCN-H2O**	*Bandwidth:*	**2**
Processing Method:	**New Processing Method**	*Dilution Factor:*	**1,0000**
Injection Date/Time:	**11.1.22 14:25**	*Sample Weight:*	**1,0000**

Chromatogram

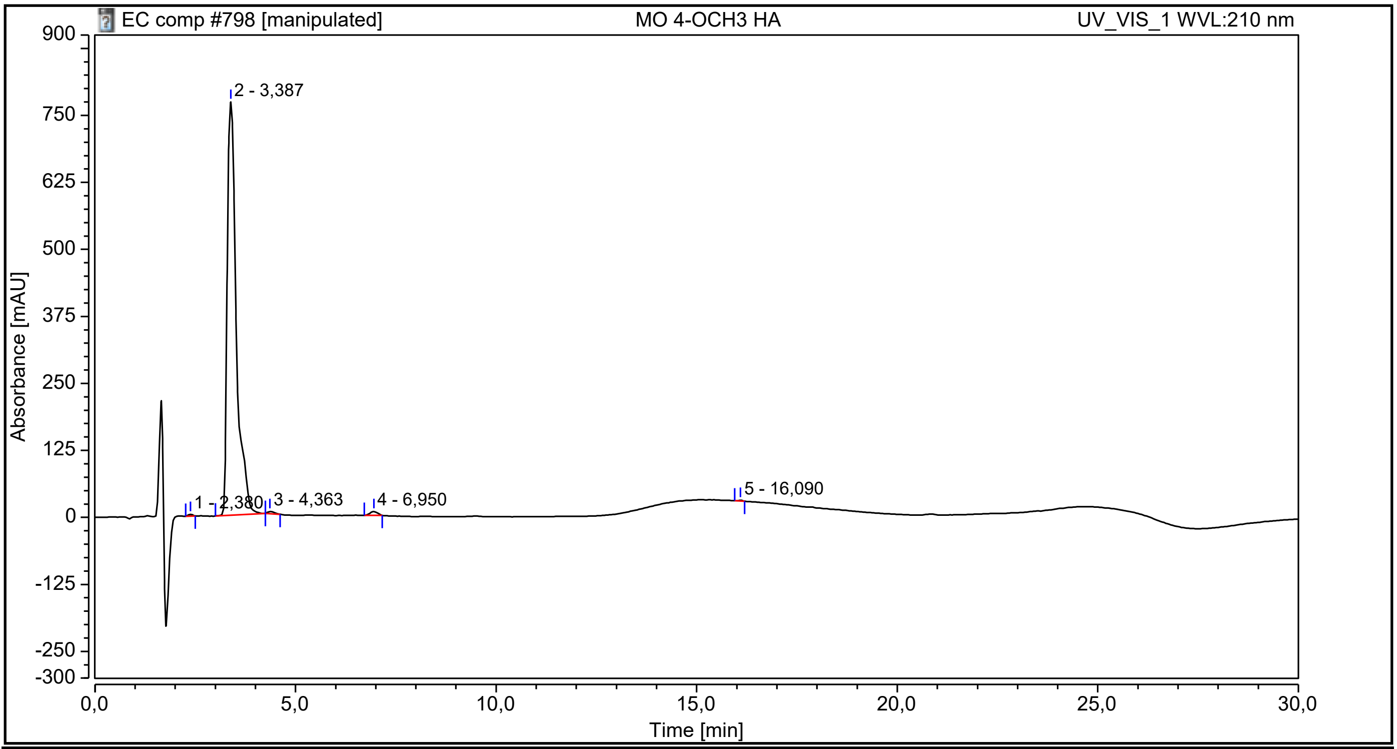

Integration Results

No.	Peak Name	Retention Time<br>min	Area<br>mAU*min	Height<br>mAU	Relative Area<br>%	Relative Height<br>%	Amount<br>n.a.
1	n.a.	2,380	0,457	3,421	0,224	0,43	n.a.
2	n.a.	3,387	201,098	770,606	98,500	97,96	n.a.
3	n.a.	4,363	0,795	3,886	0,389	0,49	n.a.
4	n.a.	6,950	1,616	7,250	0,791	0,92	n.a.
5	n.a.	16,090	0,195	1,470	0,096	0,19	n.a.
**Total:**			**204,161**	**786,633**	**100,00**	**100,00**	

(2*E*)-3-(4-{[(2-Fluorophenyl)carbamoyl]methoxy}phenyl)-*N*-hydroxyprop-2-enamide (**7h**)

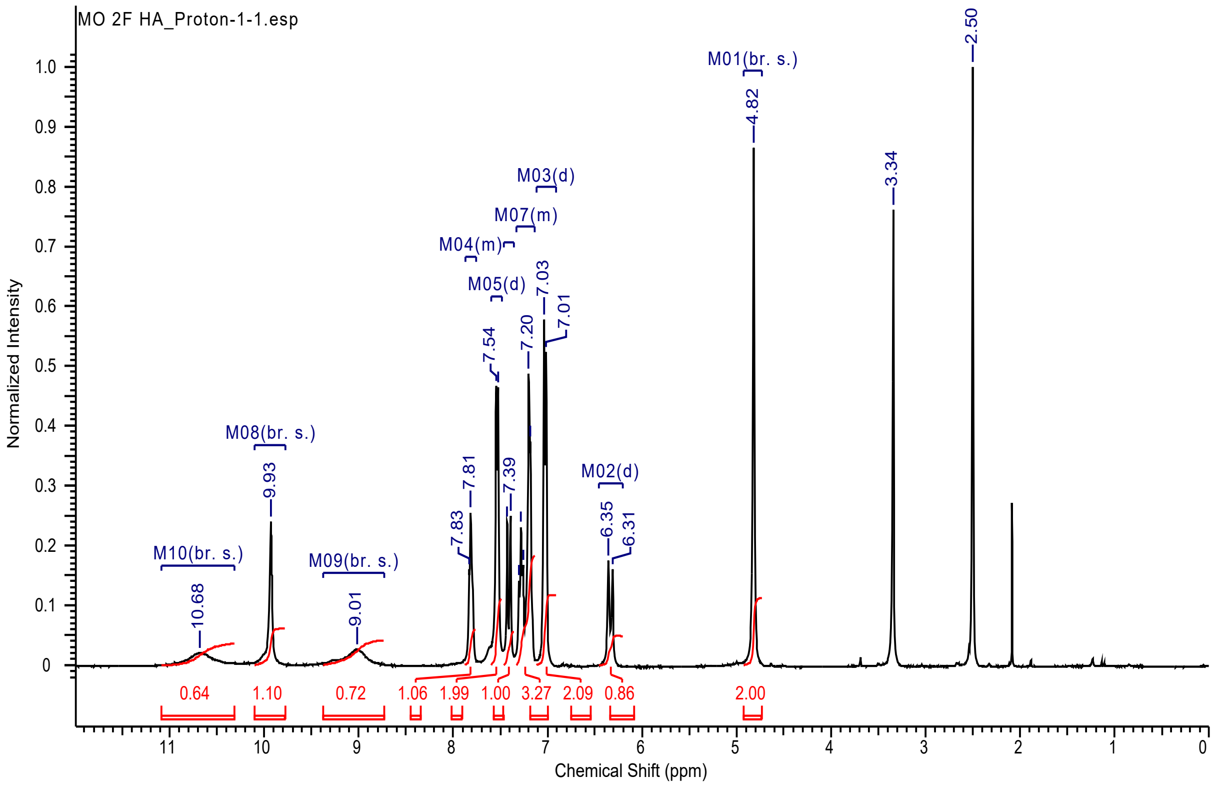

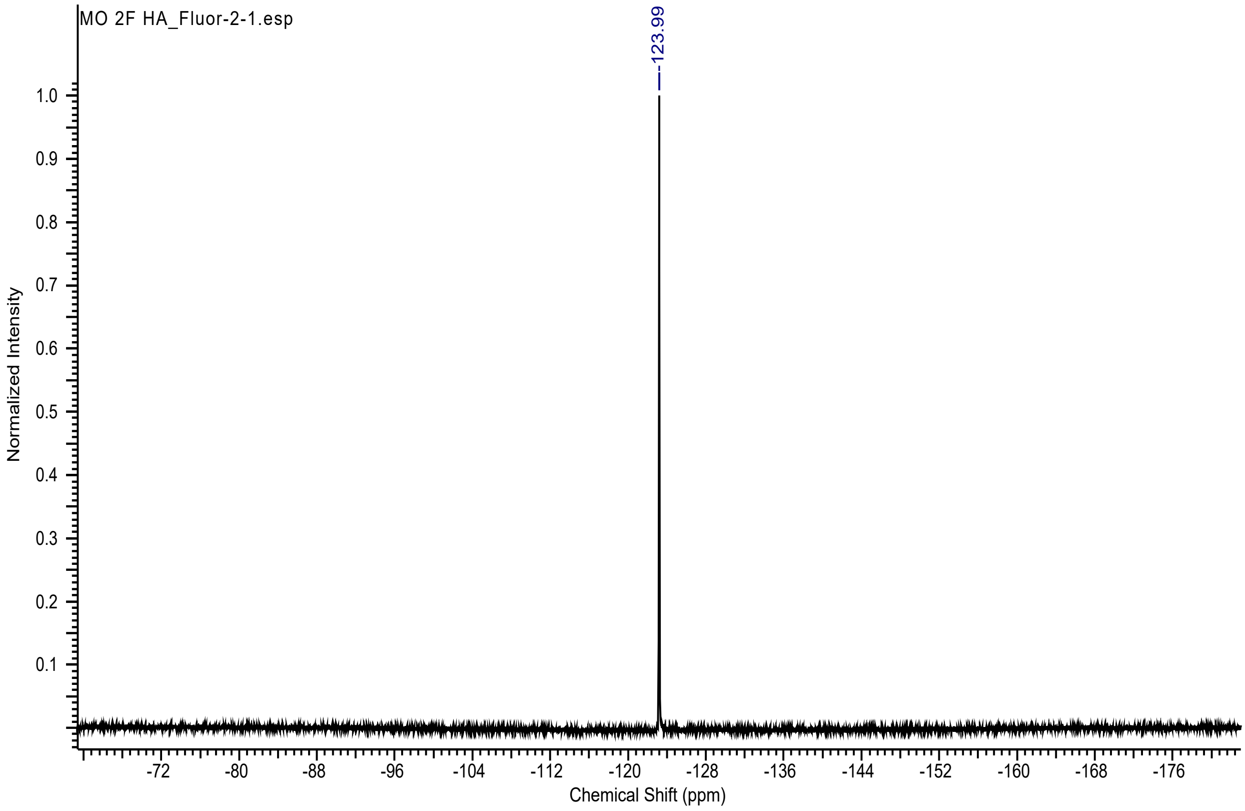

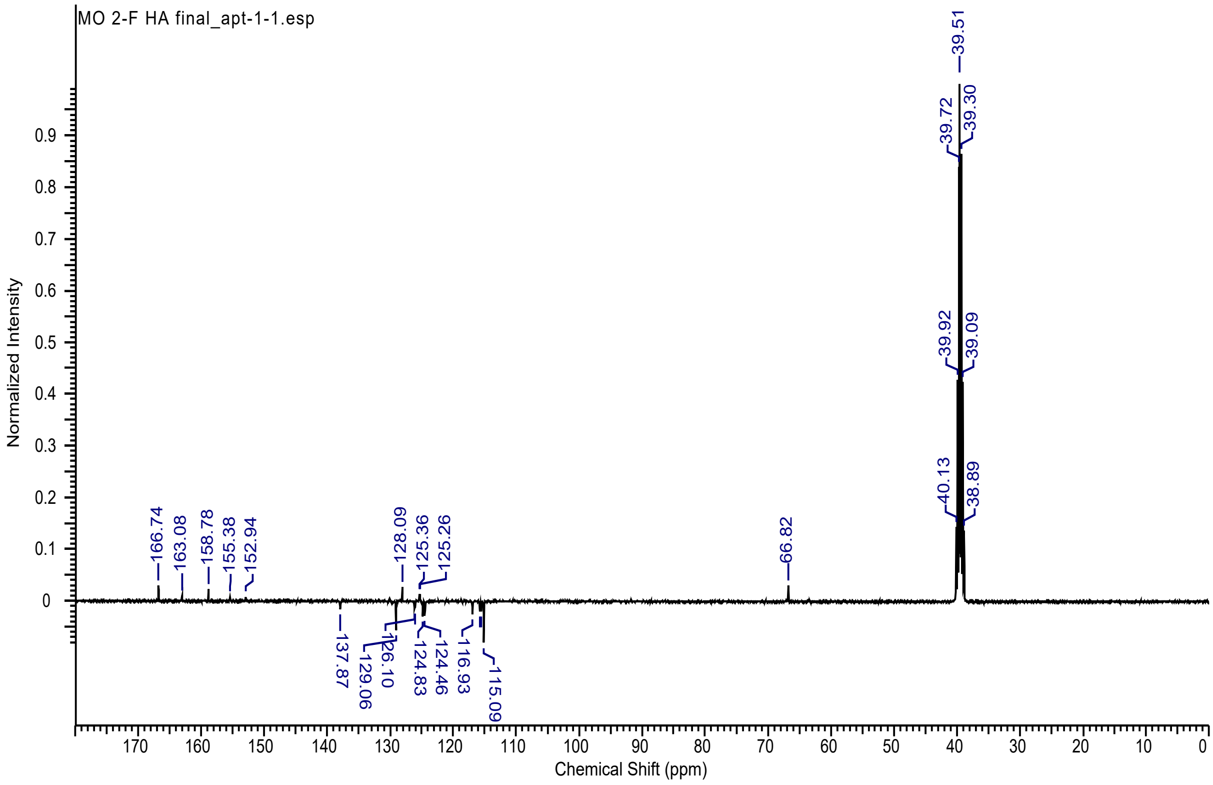

MO 2-F HA final_apt-1-1.esp
Normalized Intensity
Chemical Shift (ppm)
166.74
163.08
158.78
155.38
152.94
128.09
125.36
125.26
137.87
129.06
126.10
124.83
124.46
116.93
115.09
66.82
39.51
39.72
39.30
39.92
39.09
40.13
38.89

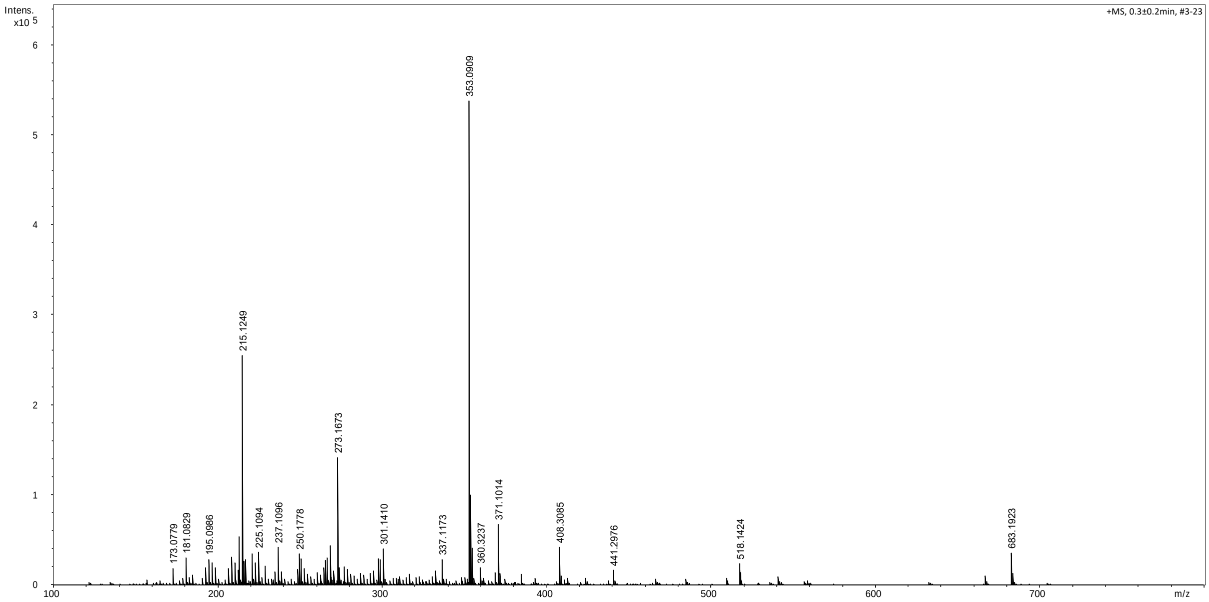

Intens.
x10 5
+MS, 0.3±0.2min, #3-23
353.0909
215.1249
273.1673
173.0779
181.0829
195.0986
225.1094
237.1096
250.1778
301.1410
337.1173
360.3237
371.1014
408.3085
441.2976
518.1424
683.1923
m/z

Chromatogram and Results

Injection Details

Injection Name:	**MO 2-F HA**	*Run Time (min):*	**30,00**
Vial Number:	**BB7**	*Injection Volume:*	**10,00**
Injection Type:	**Unknown**	*Channel:*	**UV_VIS_1**
Calibration Level:		*Wavelength:*	**210,0**
Instrument Method:	**Grad40-60to90-10 MeCN-H2O**	*Bandwidth:*	**2**
Processing Method:	**New Processing Method**	*Dilution Factor:*	**1,0000**
Injection Date/Time:	**06.1.22 11:07**	*Sample Weight:*	**1,0000**

Chromatogram

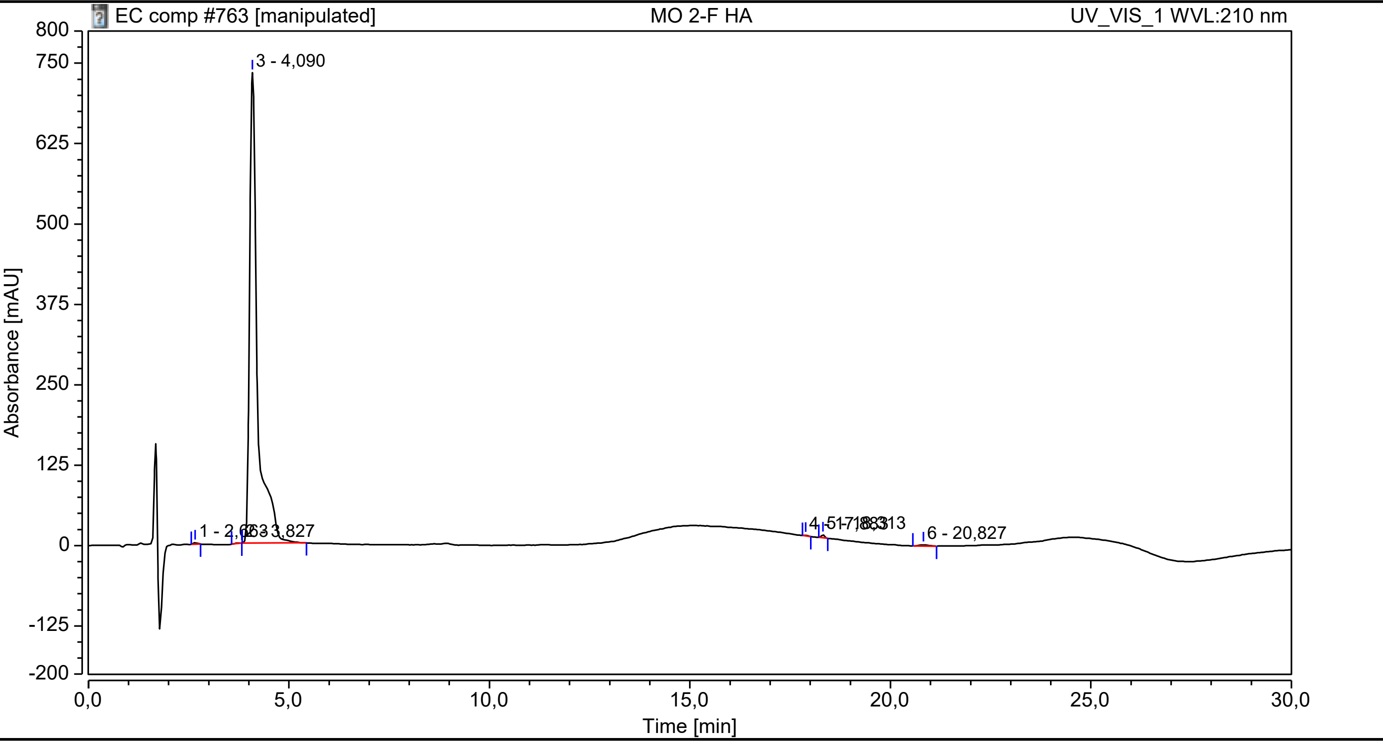

Integration Results

No.	Peak Name	Retention Time min	Area mAU*min	Height mAU	Relative Area %	Relative Height %	Amount n.a.
1	n.a.	2,663	0,294	2,333	0,174	0,31	n.a.
2	n.a.	3,827	0,076	0,000	0,045	0,00	n.a.
3	n.a.	4,090	167,477	731,249	99,143	98,60	n.a.
4	n.a.	17,883	0,112	1,134	0,066	0,15	n.a.
5	n.a.	18,313	0,428	4,963	0,253	0,67	n.a.
6	n.a.	20,827	0,539	1,933	0,319	0,26	n.a.
**Total:**			**168,925**	**741,612**	**100,00**	**100,00**	

(2*E*)-3-(4-{[(3-Fluorophenyl)carbamoyl]methoxy}phenyl)-*N*-hydroxyprop-2-enamide (**7i**)

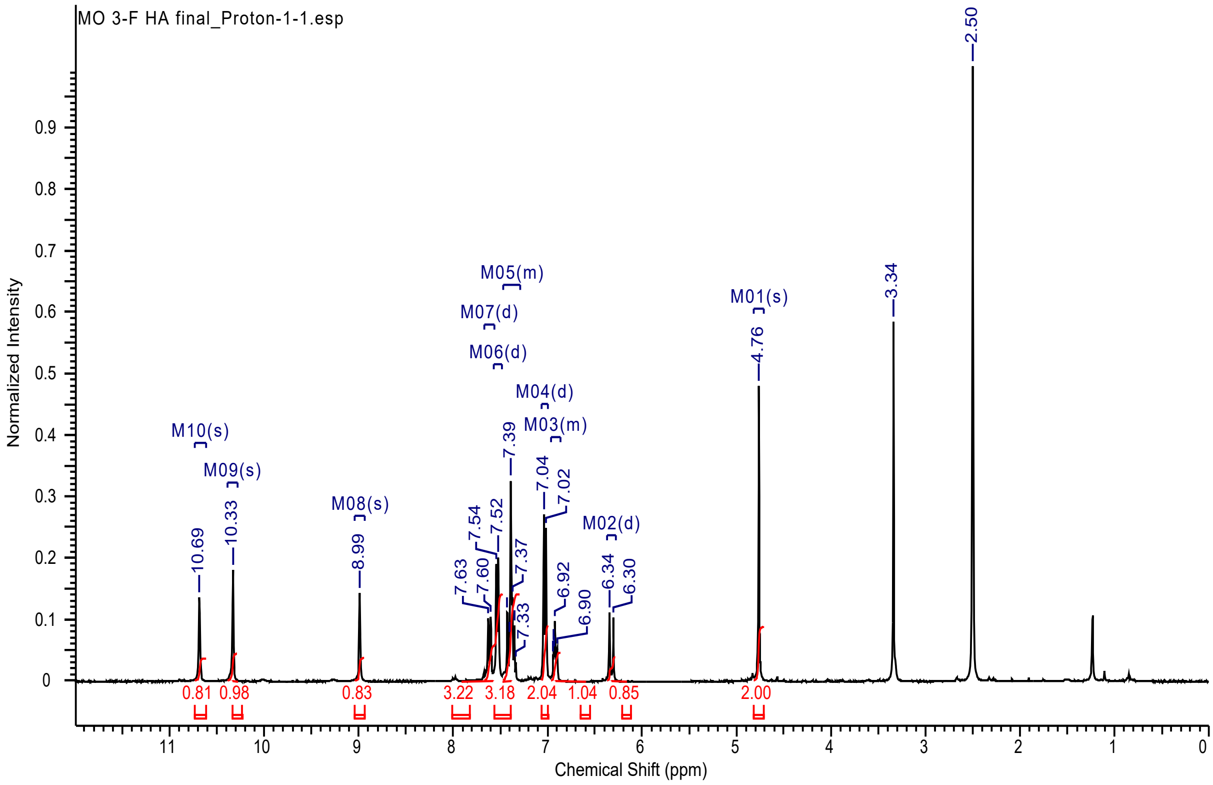

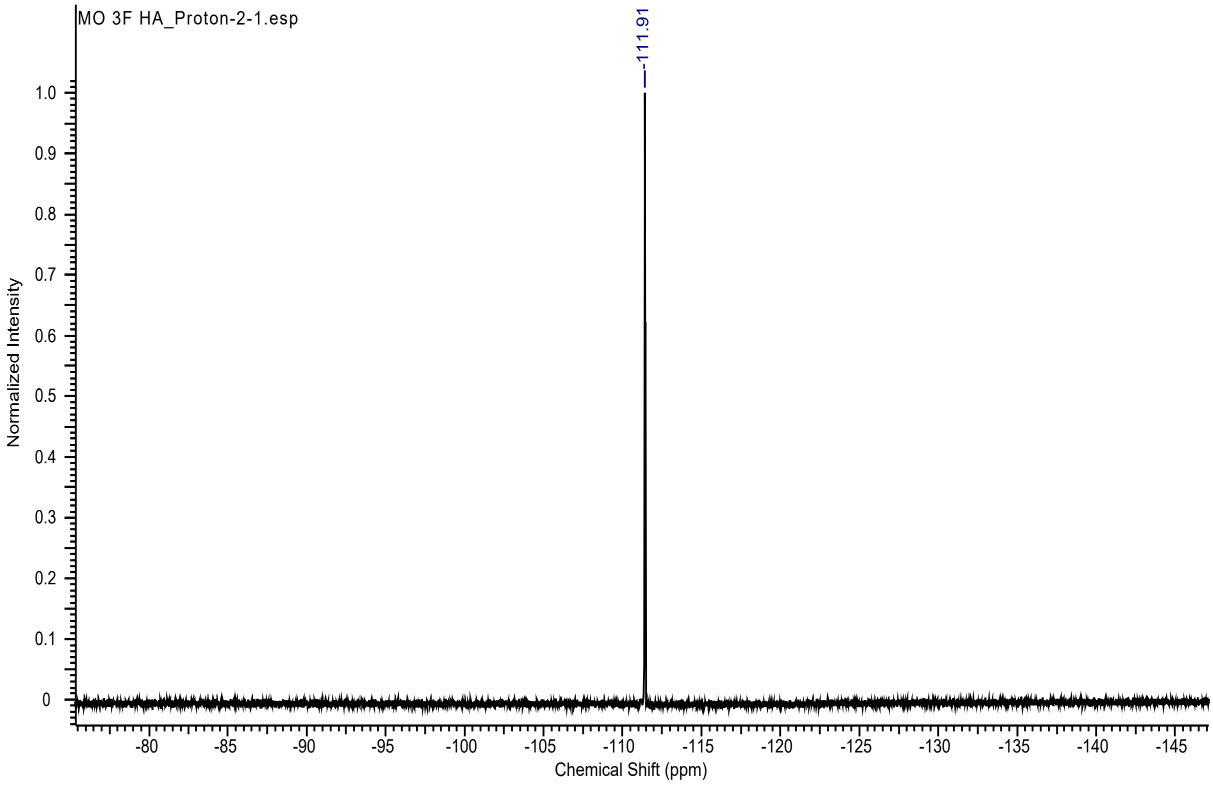

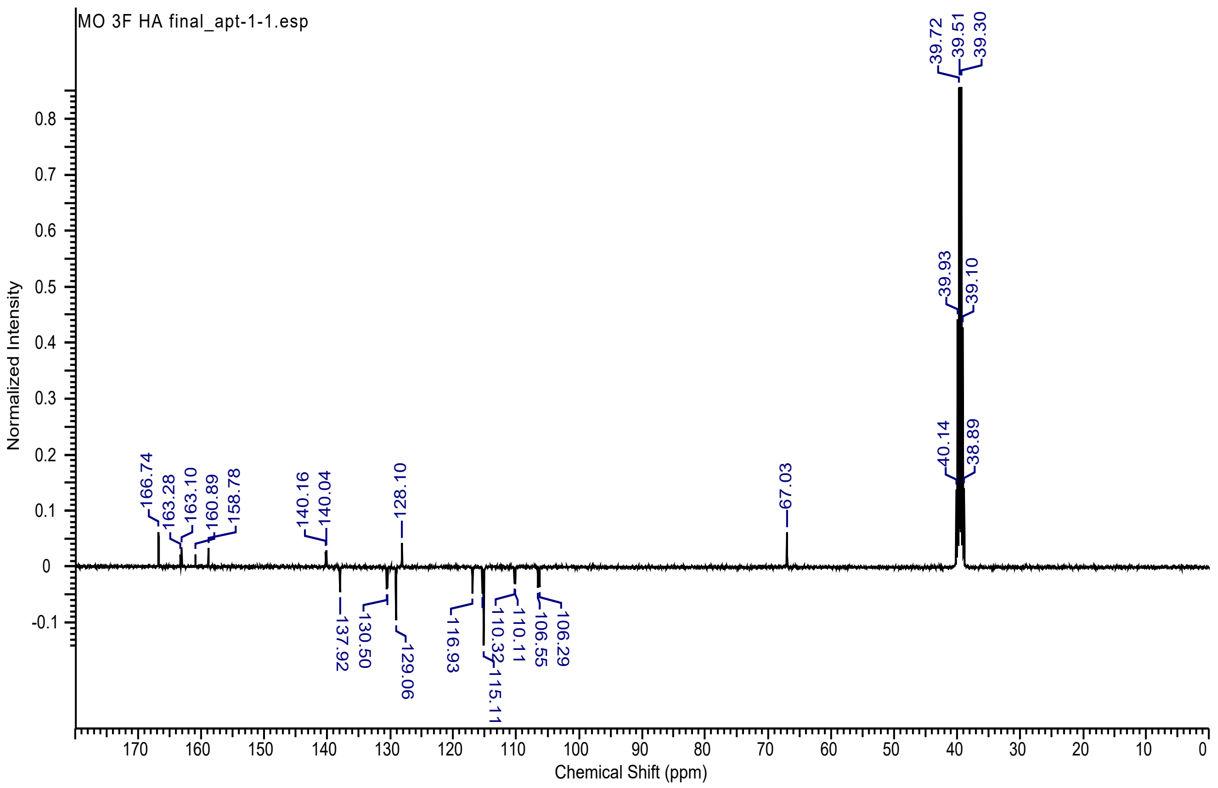
MO 3F HA final_apt-1-1.esp
39.72
39.51
39.30
39.93
39.10
40.14
38.89
166.74
163.28
163.10
160.89
158.78
140.16
140.04
128.10
67.03
137.92
130.50
129.06
116.93
110.32
115.11
110.11
106.55
106.29
Normalized Intensity
Chemical Shift (ppm)

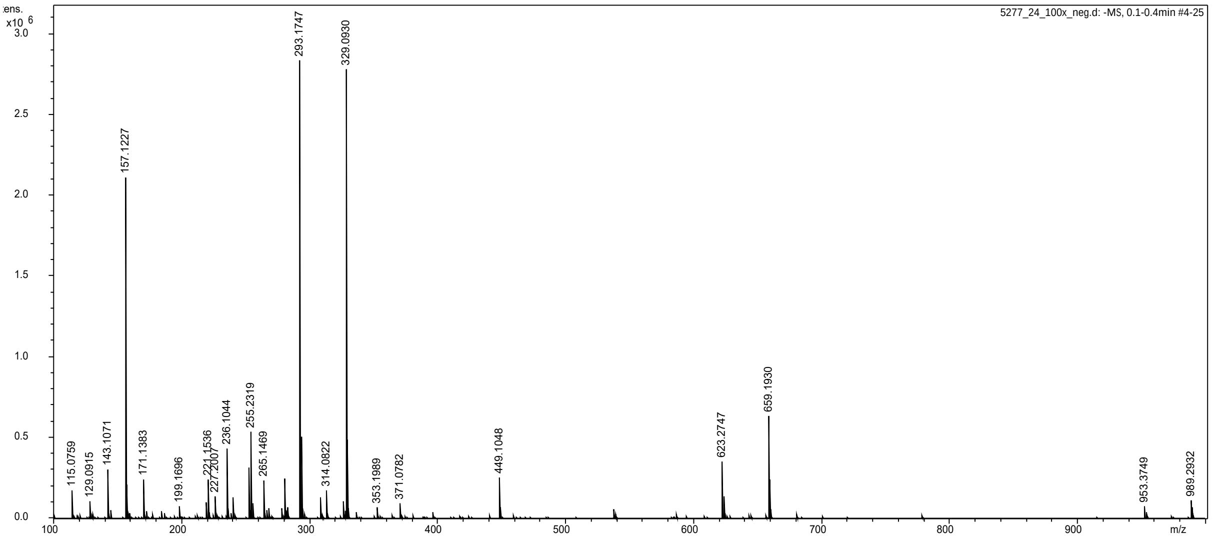
5277_24_100x_neg.d: -MS, 0.1-0.4min #4-25
x10 6
293.1747
329.0930
157.1227
659.1930
623.2747
449.1048
255.2319
236.1044
221.1536
227.2007
265.1469
171.1383
143.1071
129.0915
115.0759
199.1696
314.0822
353.1989
371.0782
953.3749
989.2932
m/z

Chromatogram and Results

Injection Details

Injection Name:	**MO 3-F HA**	*Run Time (min):*	**30,00**
Vial Number:	**BB8**	*Injection Volume:*	**10,00**
Injection Type:	**Unknown**	*Channel:*	**UV_VIS_1**
Calibration Level:		*Wavelength:*	**210,0**
Instrument Method:	**Grad40-60to90-10 MeCN-H2O**	*Bandwidth:*	**2**
Processing Method:	**New Processing Method**	*Dilution Factor:*	**1,0000**
Injection Date/Time:	**06.1.22 13:13**	*Sample Weight:*	**1,0000**

Chromatogram

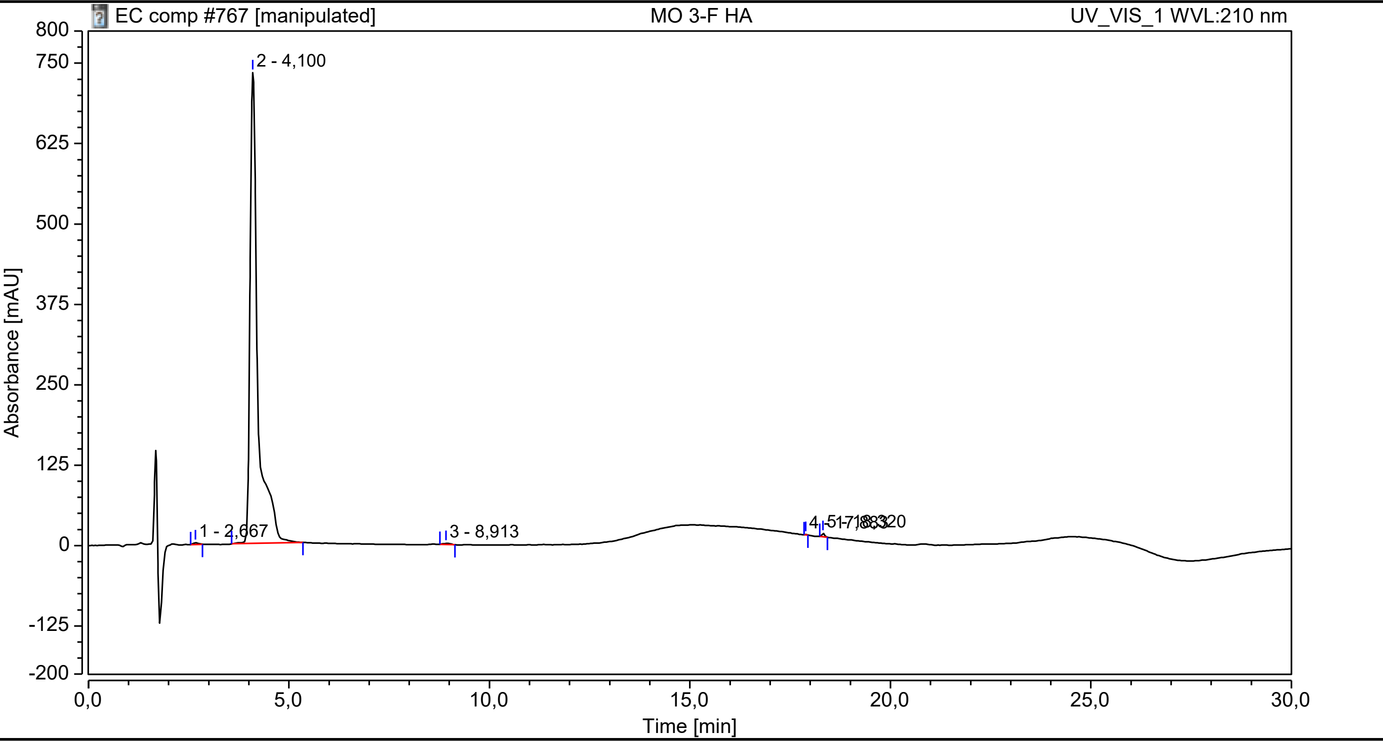

Integration Results

No.	Peak Name	Retention Time<br>min	Area<br>mAU*min	Height<br>mAU	Relative Area<br>%	Relative Height<br>%	Amount<br>n.a.
1	n.a.	2,667	0,361	2,565	0,216	0,35	n.a.
2	n.a.	4,100	165,742	731,901	99,268	98,60	n.a.
3	n.a.	8,913	0,411	1,935	0,246	0,26	n.a.
4	n.a.	17,883	0,037	0,715	0,022	0,10	n.a.
5	n.a.	18,320	0,413	5,192	0,247	0,70	n.a.
**Total:**			**166,963**	**742,307**	**100,00**	**100,00**	

Chromeleon (c) Dionex
Version 7.2.0.3765

(2*E*)-3-(4-{[(4-Fluorophenyl)carbamoyl]methoxy}phenyl)-*N*-hydroxyprop-2-enamide (**7j**)

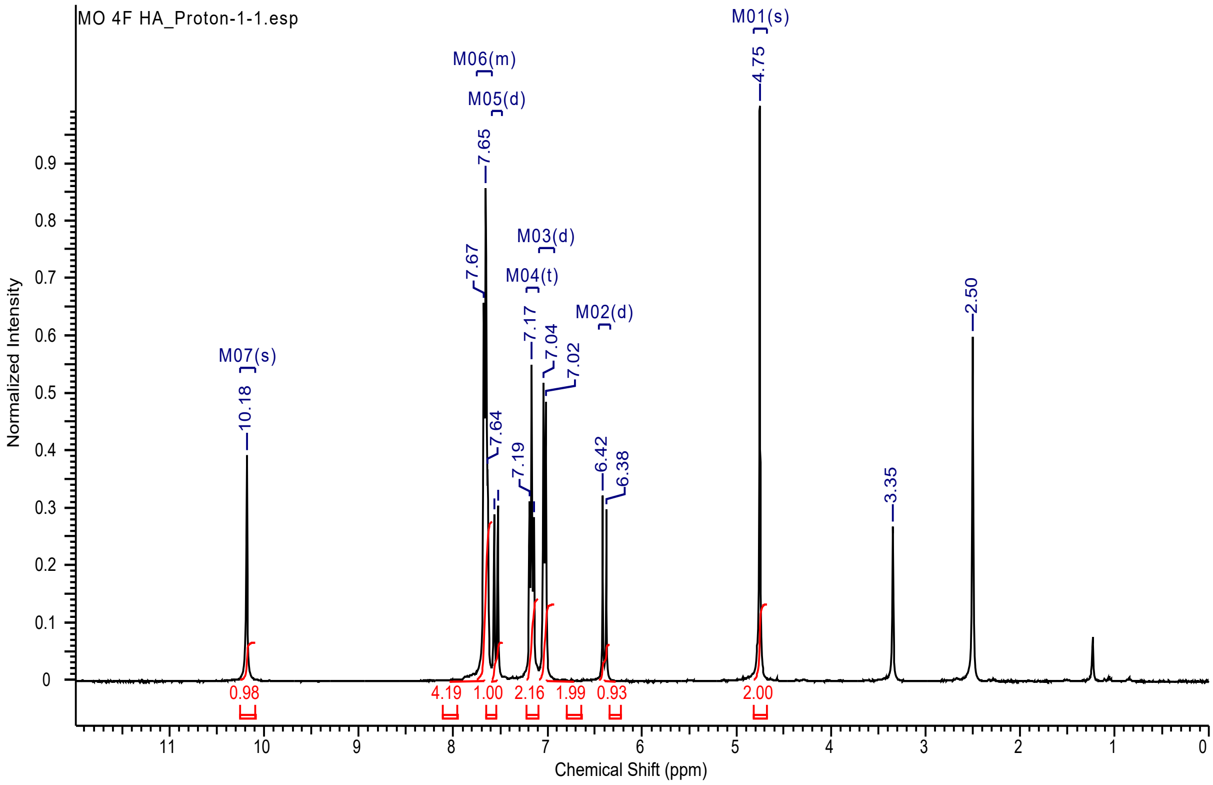

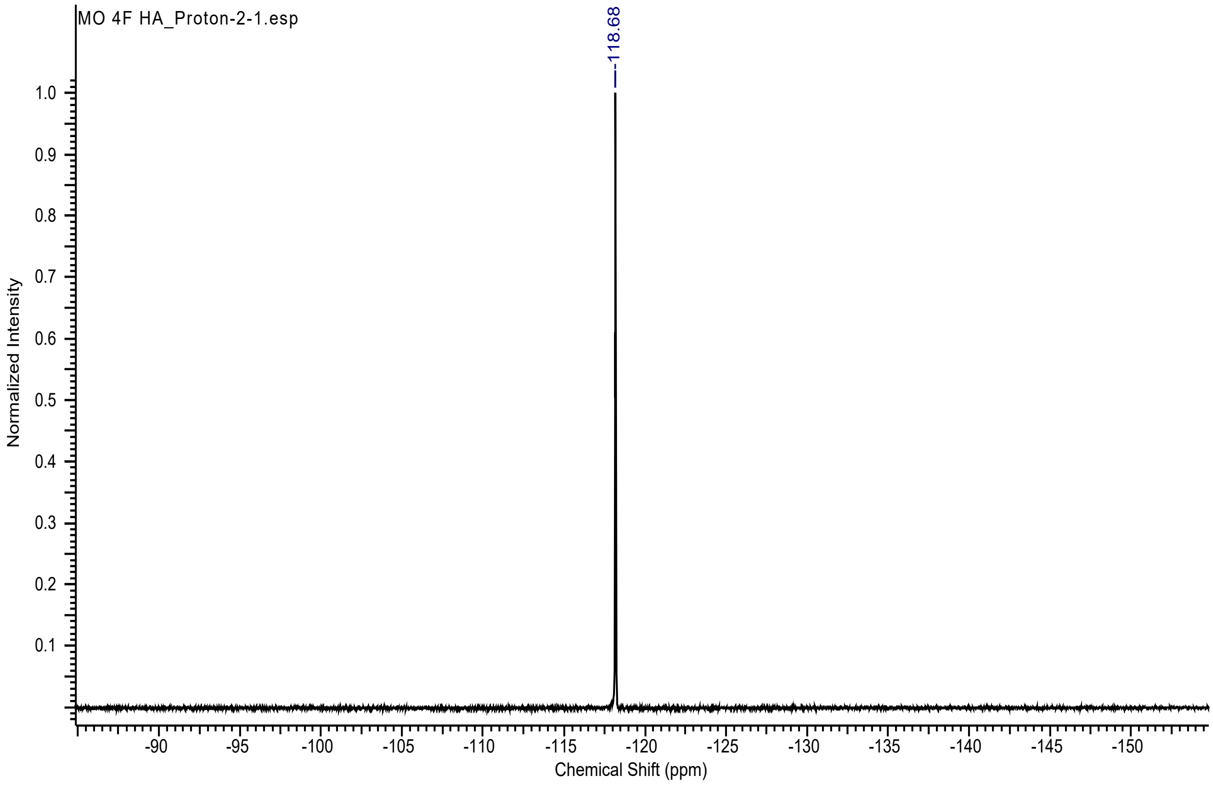

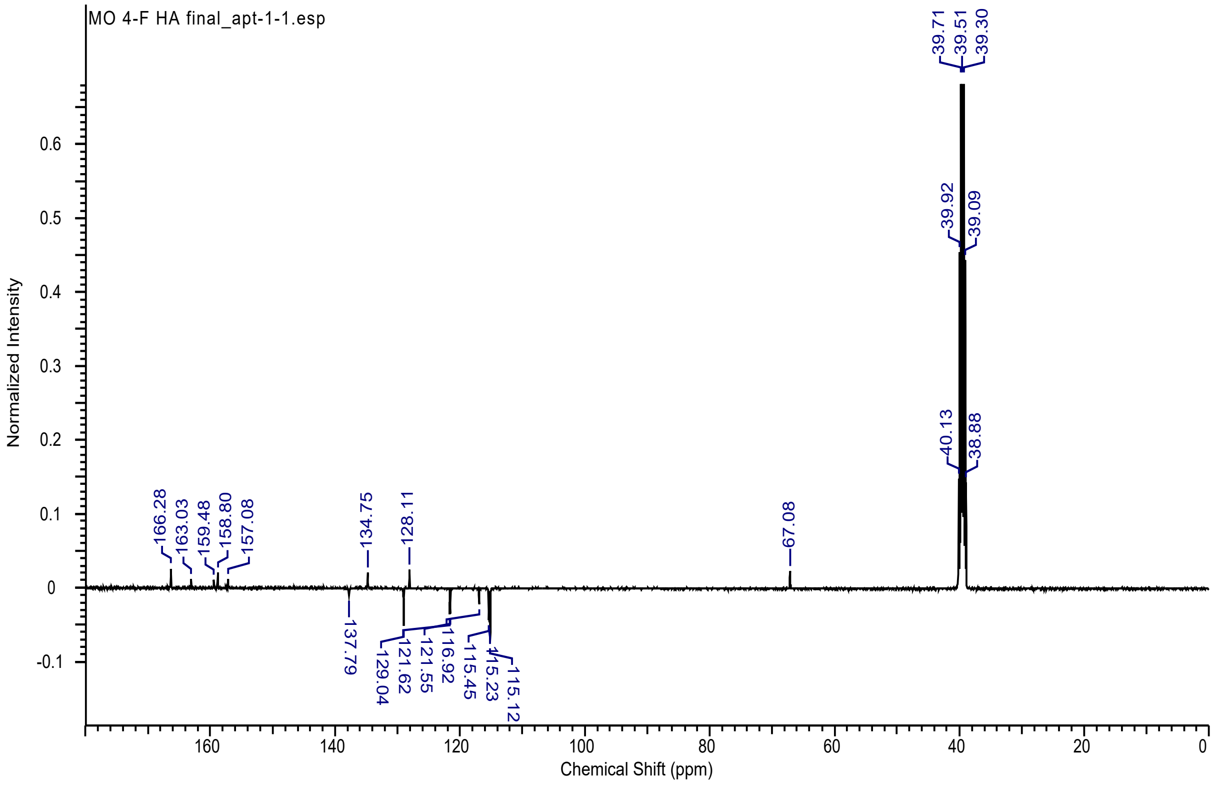

MO 4-F HA final_apt-1-1.esp
Normalized Intensity
Chemical Shift (ppm)
166.28
163.03
159.48
158.80
157.08
134.75
128.11
137.79
129.04
121.62
121.55
116.92
115.45
115.23
115.12
67.08
39.71
39.51
39.30
39.92
39.09
40.13
38.88

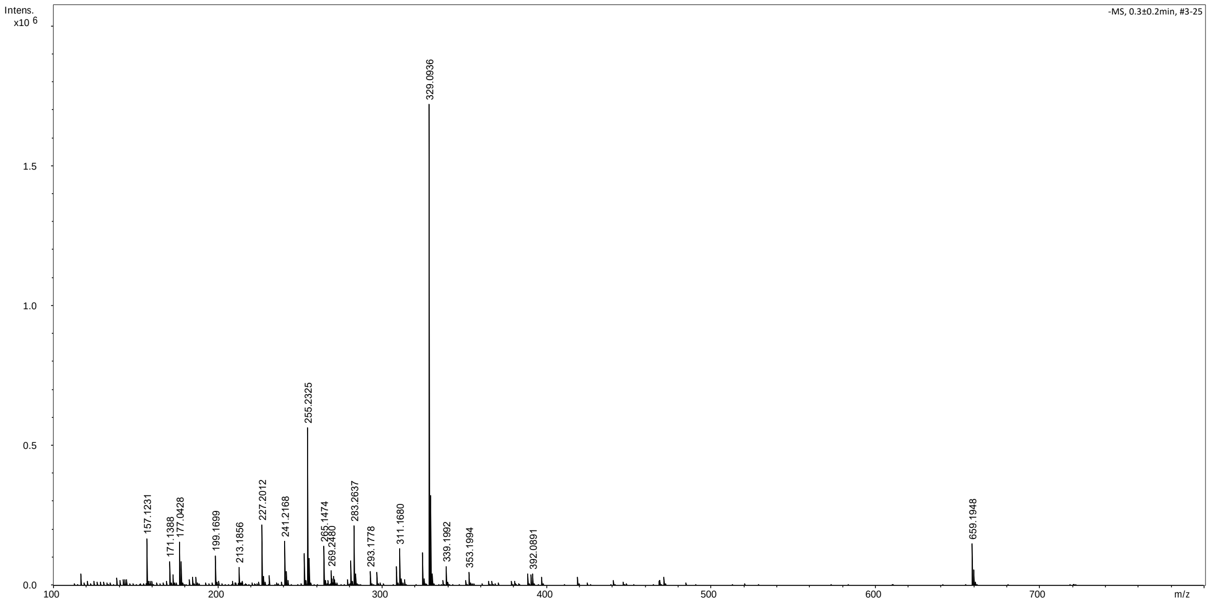

Intens.
x10 6
-MS, 0.3±0.2min, #3-25
329.0936
255.2325
157.1231
171.1388
177.0428
199.1699
213.1856
227.2012
241.2168
265.1474
269.2480
283.2637
293.1778
311.1680
339.1992
353.1994
392.0891
659.1948
m/z

Chromatogram and Results

Injection Details

Injection Name:	**MO 4-F HA**	*Run Time (min):*	**30,00**
Vial Number:	**BC1**	*Injection Volume:*	**10,00**
Injection Type:	**Unknown**	*Channel:*	**UV_VIS_1**
Calibration Level:		*Wavelength:*	**210,0**
Instrument Method:	**Grad40-60to90-10 MeCN-H2O**	*Bandwidth:*	**2**
Processing Method:	**New Processing Method**	*Dilution Factor:*	**1,0000**
Injection Date/Time:	**06.1.22 13:44**	*Sample Weight:*	**1,0000**

Chromatogram

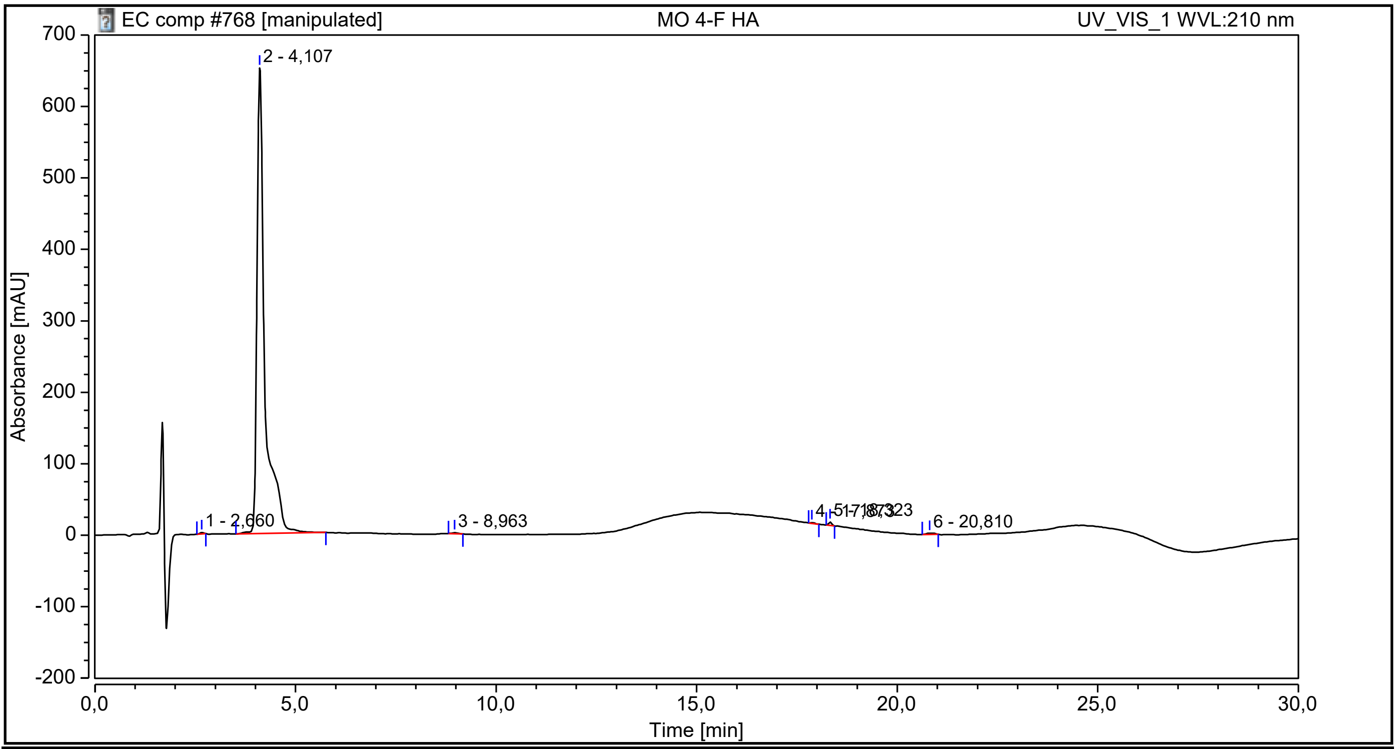

Integration Results

No.	Peak Name	Retention Time min	Area mAU*min	Height mAU	Relative Area %	Relative Height %	Amount n.a.
1	n.a.	2,660	0,211	1,845	0,140	0,28	n.a.
2	n.a.	4,107	149,355	651,187	99,048	98,37	n.a.
3	n.a.	8,963	0,267	1,399	0,177	0,21	n.a.
4	n.a.	17,873	0,118	0,968	0,078	0,15	n.a.
5	n.a.	18,323	0,414	4,879	0,275	0,74	n.a.
6	n.a.	20,810	0,426	1,690	0,282	0,26	n.a.
**Total:**			**150,790**	**661,968**	**100,00**	**100,00**	

(2*E*)-3-(4-{[(2-Chlorophenyl)carbamoyl]methoxy}phenyl)-*N*-hydroxyprop-2-enamide (**7k**)

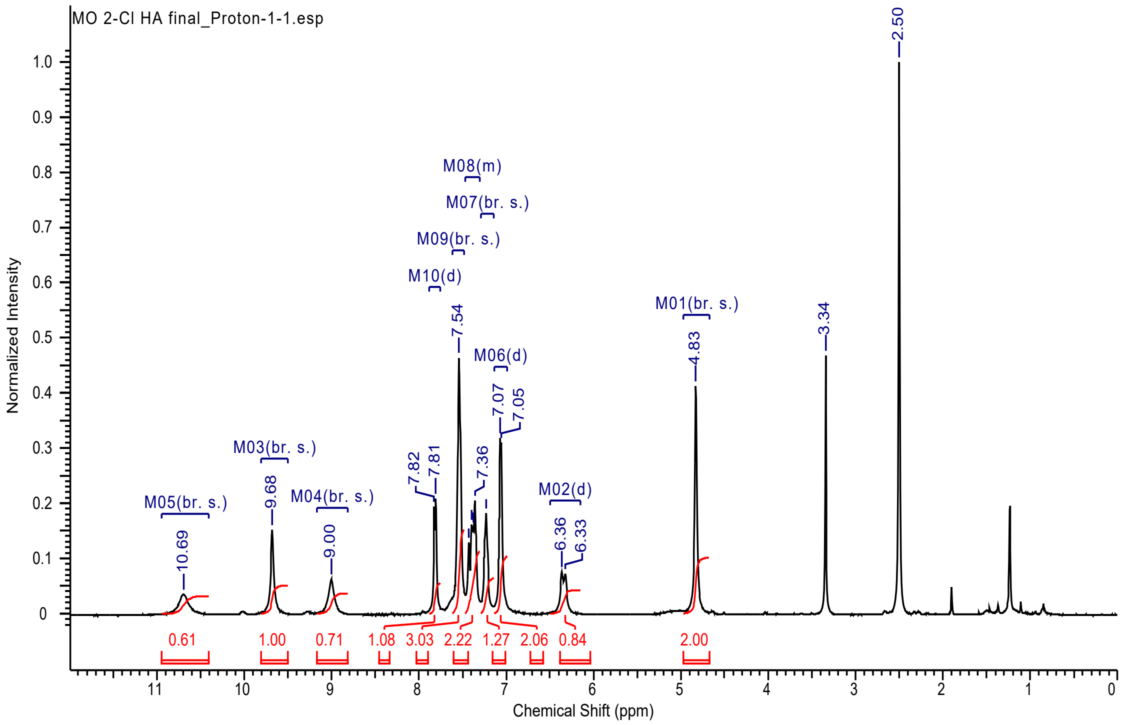

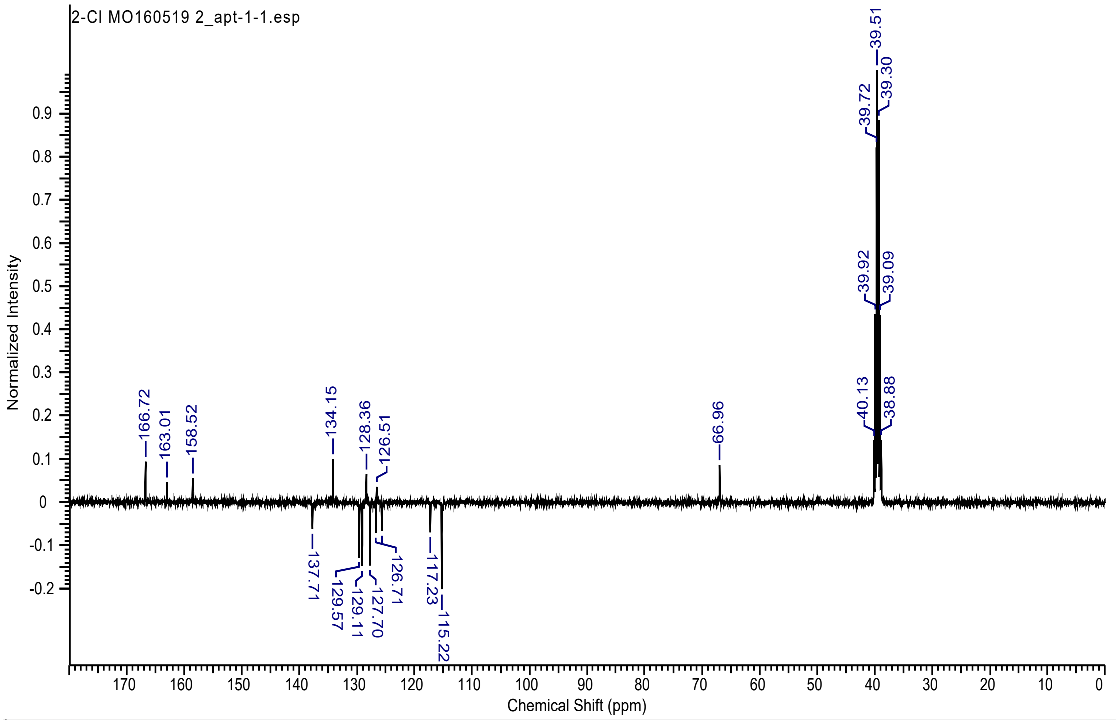

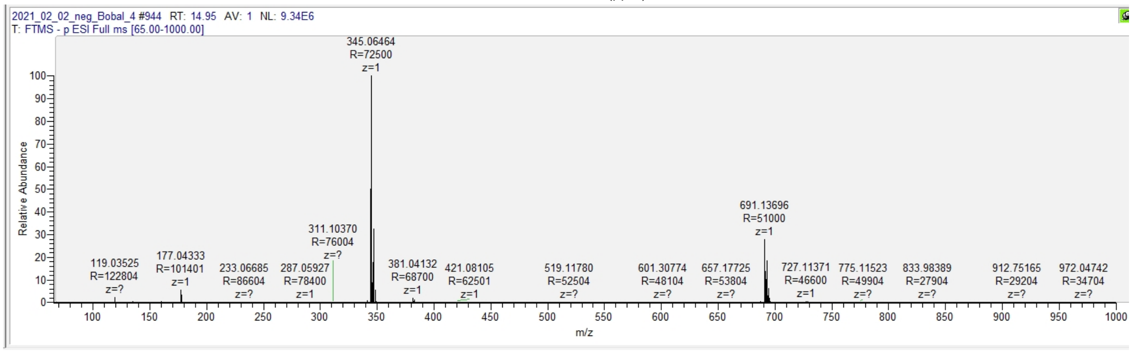

Chromatogram and Results

Injection Details

Injection Name:	**MO 2-Cl HA**	*Run Time (min):*	**30,00**
Vial Number:	**BD8**	*Injection Volume:*	**1,50**
Injection Type:	**Unknown**	*Channel:*	**UV_VIS_1**
Calibration Level:		*Wavelength:*	**210,0**
Instrument Method:	**Grad40-60to90-10 MeCN-H2O**	*Bandwidth:*	**2**
Processing Method:	**New Processing Method**	*Dilution Factor:*	**1,0000**
Injection Date/Time:	**12.1.22 14:16**	*Sample Weight:*	**1,0000**

Chromatogram

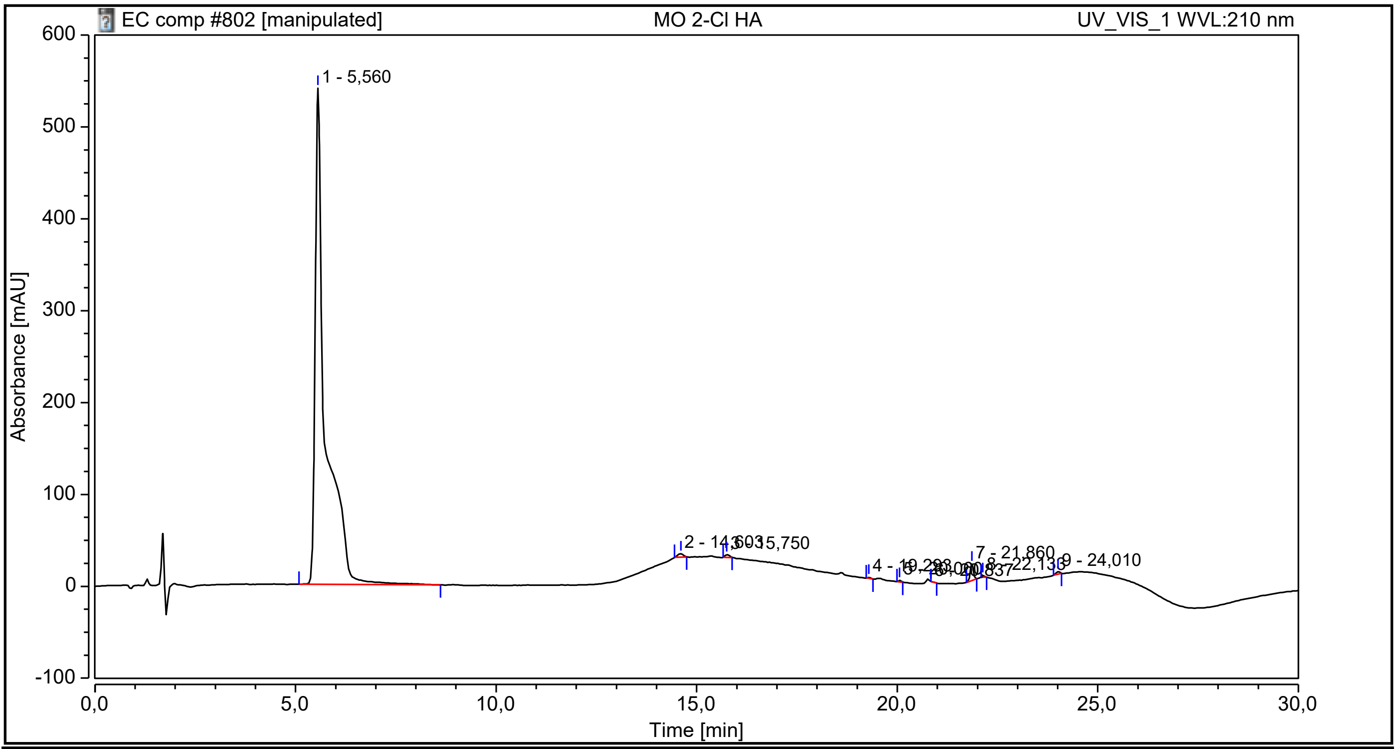

Integration Results

No.	Peak Name	Retention Time min	Area mAU*min	Height mAU	Relative Area %	Relative Height %	Amount n.a.
1	n.a.	5,560	158,165	539,910	98,008	94,48	n.a.
2	n.a.	14,603	0,608	3,661	0,377	0,64	n.a.
3	n.a.	15,750	0,363	2,989	0,225	0,52	n.a.
4	n.a.	19,293	0,093	1,199	0,058	0,21	n.a.
5	n.a.	20,060	0,125	1,624	0,077	0,28	n.a.
6	n.a.	20,837	0,028	0,000	0,017	0,00	n.a.
7	n.a.	21,860	1,589	17,851	0,985	3,12	n.a.
8	n.a.	22,133	0,111	1,504	0,069	0,26	n.a.
9	n.a.	24,010	0,298	2,704	0,184	0,47	n.a.
**Total:**			**161,380**	**571,443**	**100,00**	**100,00**	

(2*E*)-3-(4-{[(3-Chlorophenyl)carbamoyl]methoxy}phenyl)-*N*-hydroxyprop-2-enamide (**7l**)

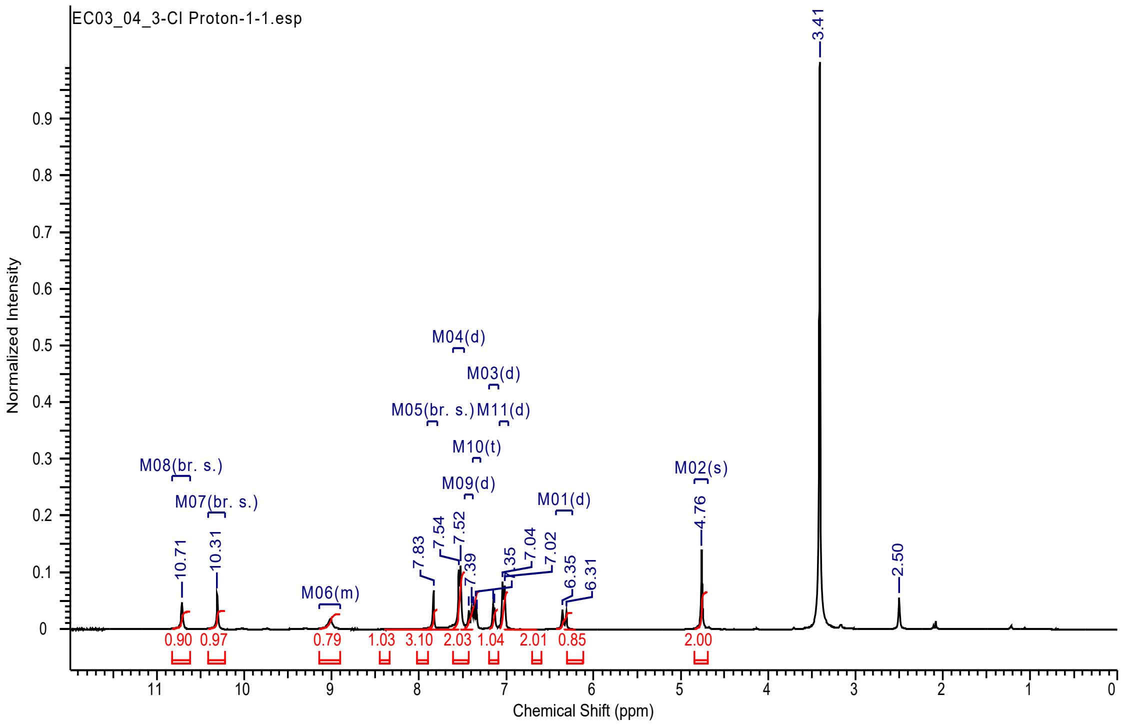

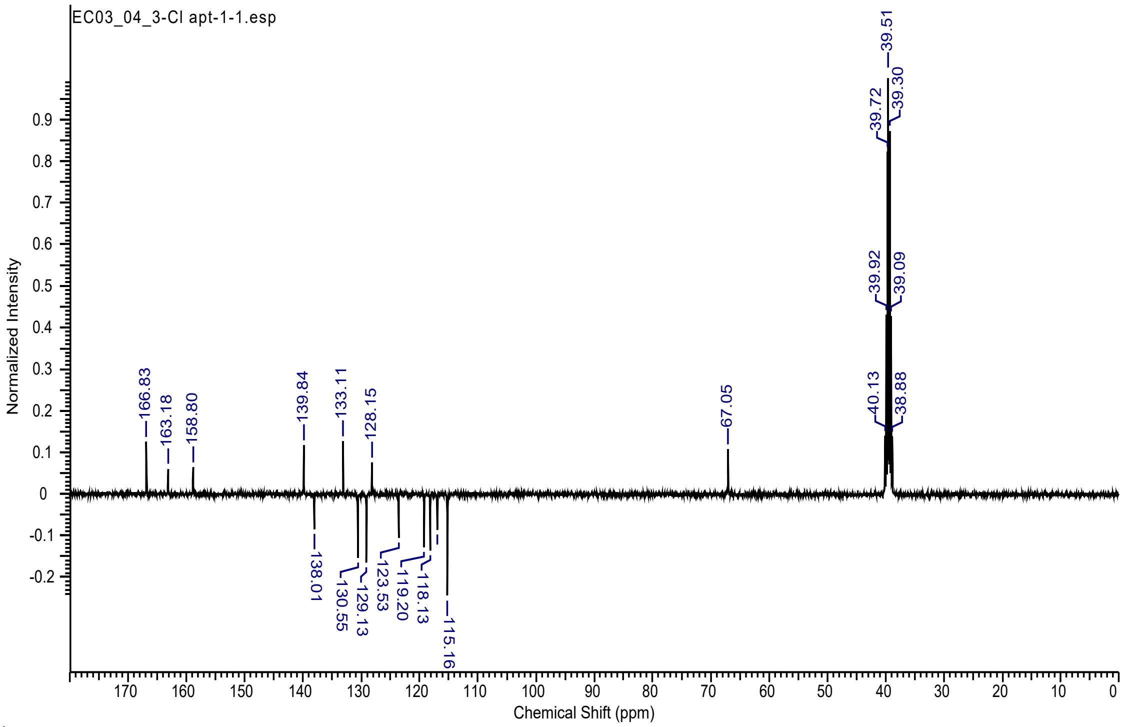

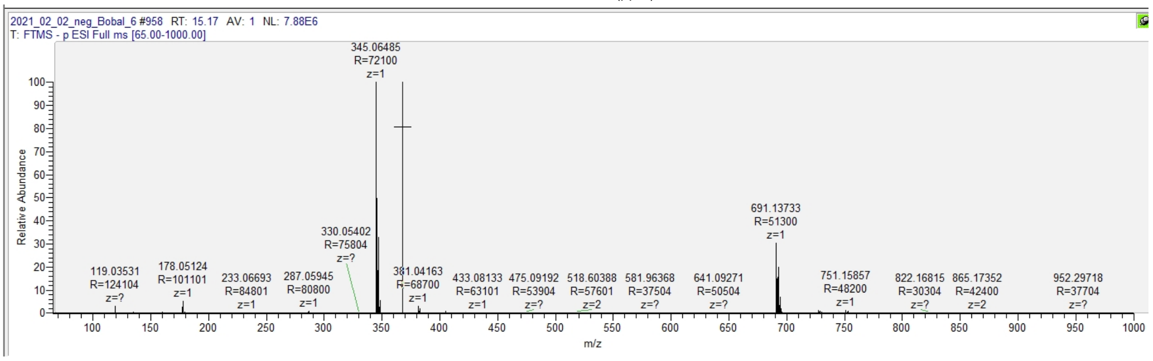

Chromatogram and Results

Injection Details

Injection Name:	**MO 3-Cl HA**	*Run Time (min):*	**30,00**
Vial Number:	**BA4**	*Injection Volume:*	**10,00**
Injection Type:	**Unknown**	*Channel:*	**UV_VIS_1**
Calibration Level:		*Wavelength:*	**210,0**
Instrument Method:	**Grad40-60to90-10 MeCN-H2O**	*Bandwidth:*	**2**
Processing Method:	**New Processing Method**	*Dilution Factor:*	**1,0000**
Injection Date/Time:	**04.1.22 15:26**	*Sample Weight:*	**1,0000**

Chromatogram

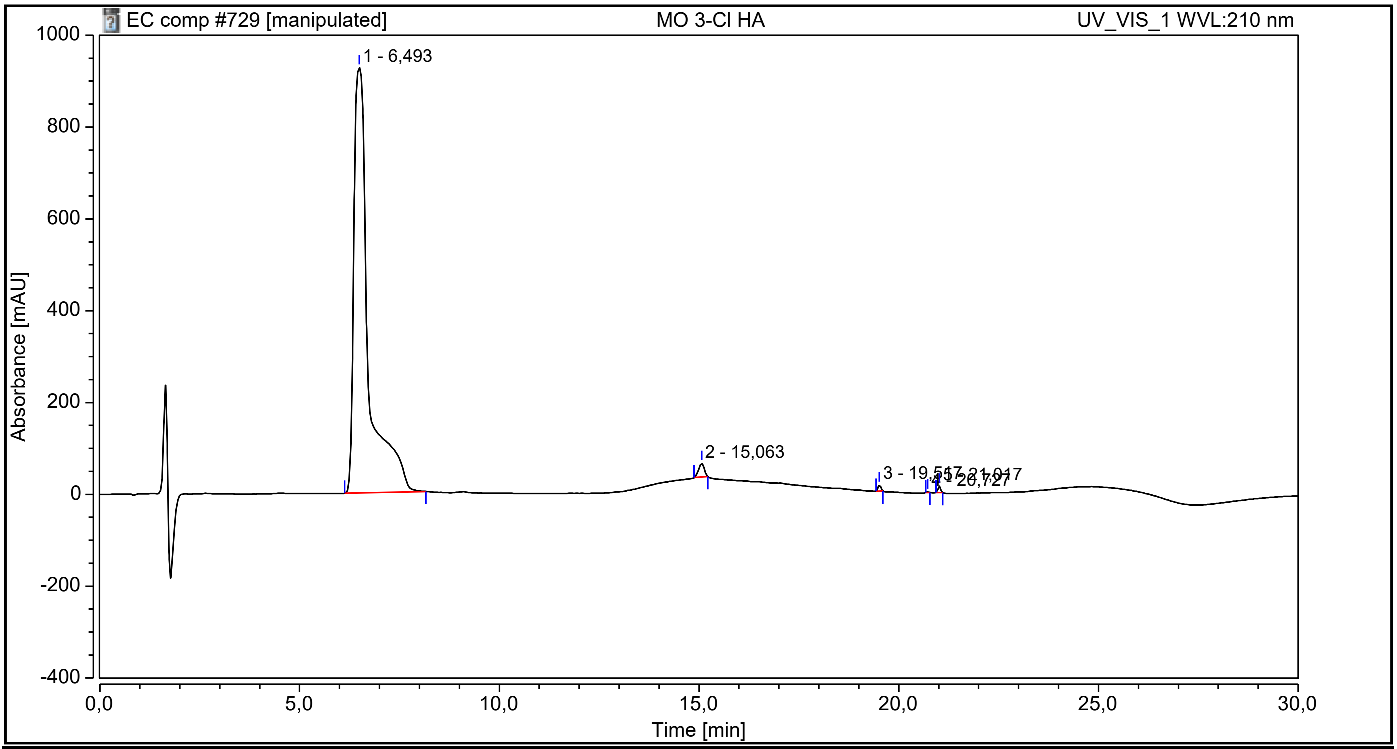

Integration Results

No.	Peak Name	Retention Time min	Area mAU*min	Height mAU	Relative Area %	Relative Height %	Amount n.a.
1	n.a.	6,493	392,395	926,819	98,206	94,07	n.a.
2	n.a.	15,063	4,925	29,449	1,233	2,99	n.a.
3	n.a.	19,517	1,067	13,447	0,267	1,36	n.a.
4	n.a.	20,727	0,086	1,371	0,022	0,14	n.a.
5	n.a.	21,017	1,090	14,123	0,273	1,43	n.a.
**Total:**			**399,563**	**985,209**	**100,00**	**100,00**	

(2*E*)-3-(4-{[(4-Chlorophenyl)carbamoyl]methoxy}phenyl)-*N*-hydroxyprop-2-enamide (**7m**)

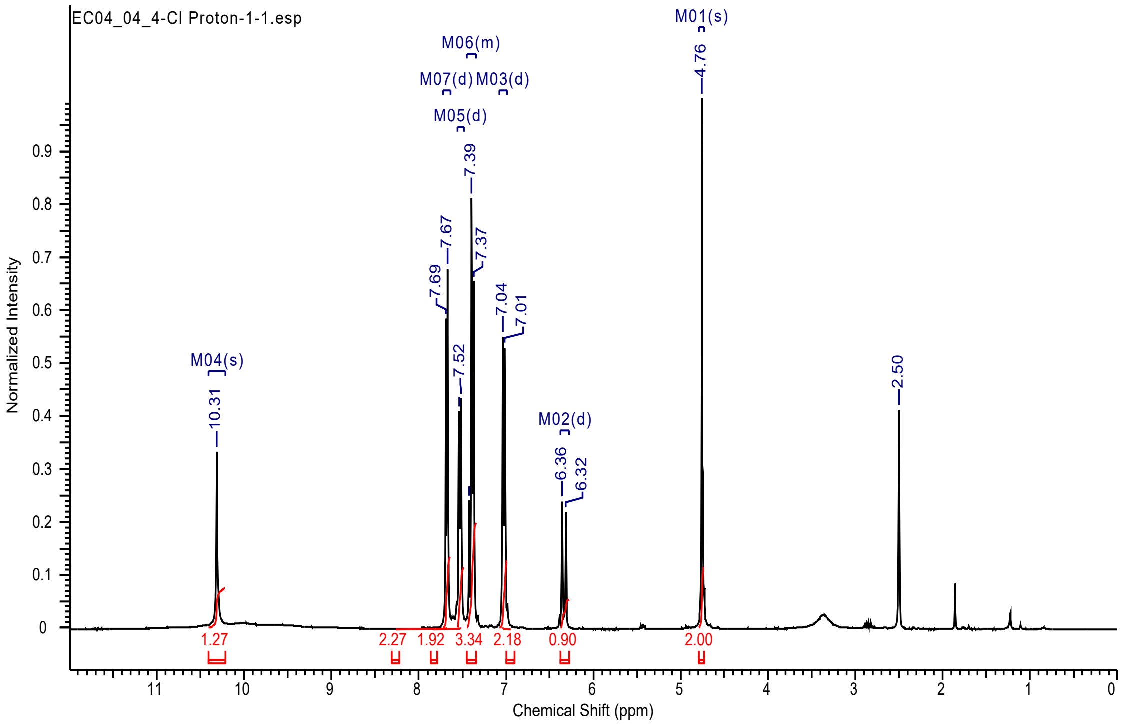

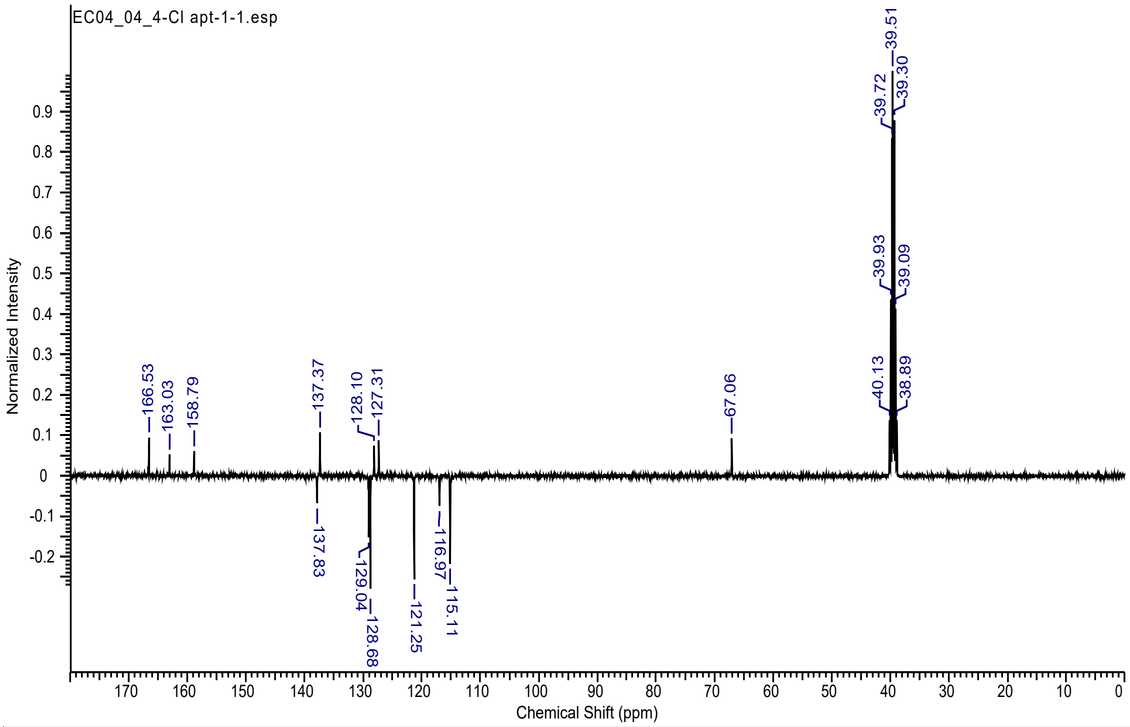

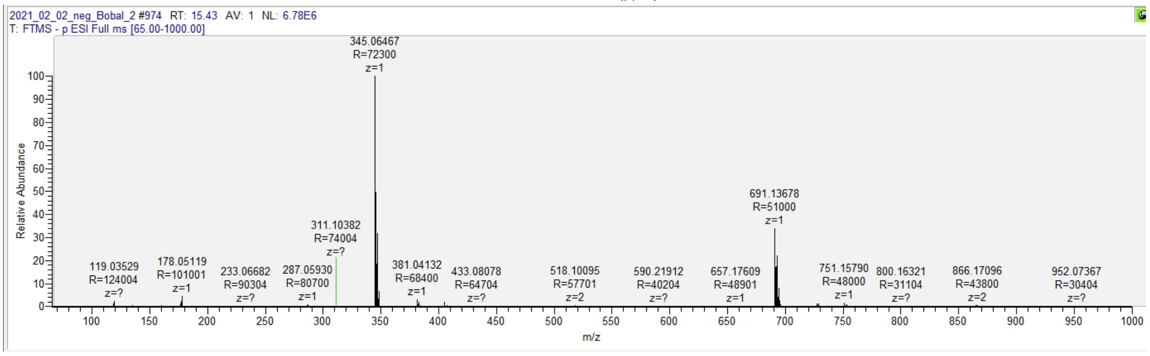

Chromatogram and Results

Injection Details

Injection Name:	**MO 4-Cl HA**	*Run Time (min):*	**30,00**
Vial Number:	**BD6**	*Injection Volume:*	**10,00**
Injection Type:	**Unknown**	*Channel:*	**UV_VIS_1**
Calibration Level:		*Wavelength:*	**210,0**
Instrument Method:	**Grad40-60to90-10 MeCN-H2O**	*Bandwidth:*	**2**
Processing Method:	**New Processing Method**	*Dilution Factor:*	**1,0000**
Injection Date/Time:	**11.1.22 13:22**	*Sample Weight:*	**1,0000**

Chromatogram

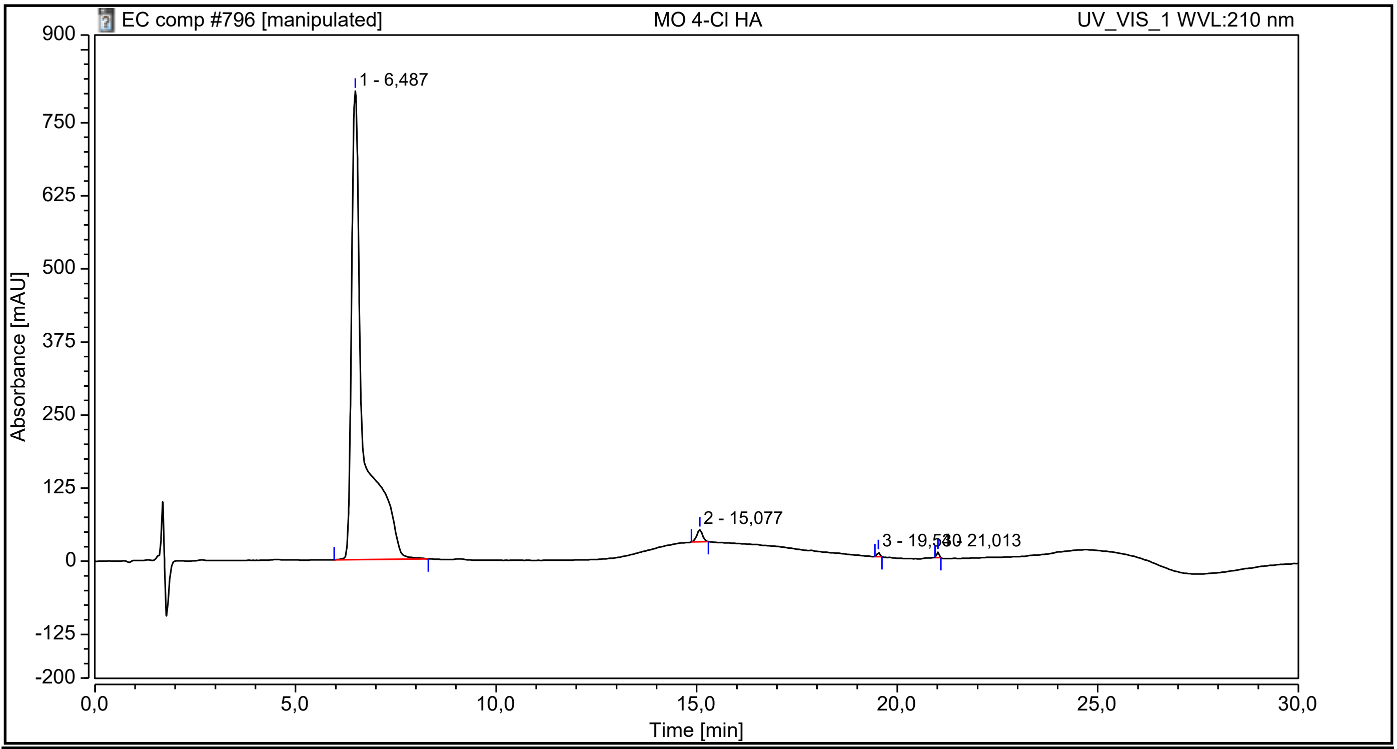

Integration Results

No.	Peak Name	Retention Time min	Area mAU*min	Height mAU	Relative Area %	Relative Height %	Amount n.a.
1	n.a.	6,487	280,541	801,085	98,331	95,60	n.a.
2	n.a.	15,077	3,510	20,644	1,230	2,46	n.a.
3	n.a.	19,530	0,586	7,163	0,205	0,85	n.a.
4	n.a.	21,013	0,667	9,033	0,234	1,08	n.a.
**Total:**			**285,303**	**837,925**	**100,00**	**100,00**	

(2*E*)-3-(4-{[(2-Bromophenyl)carbamoyl]methoxy}phenyl)-*N*-hydroxyprop-2-enamide (**7n**)

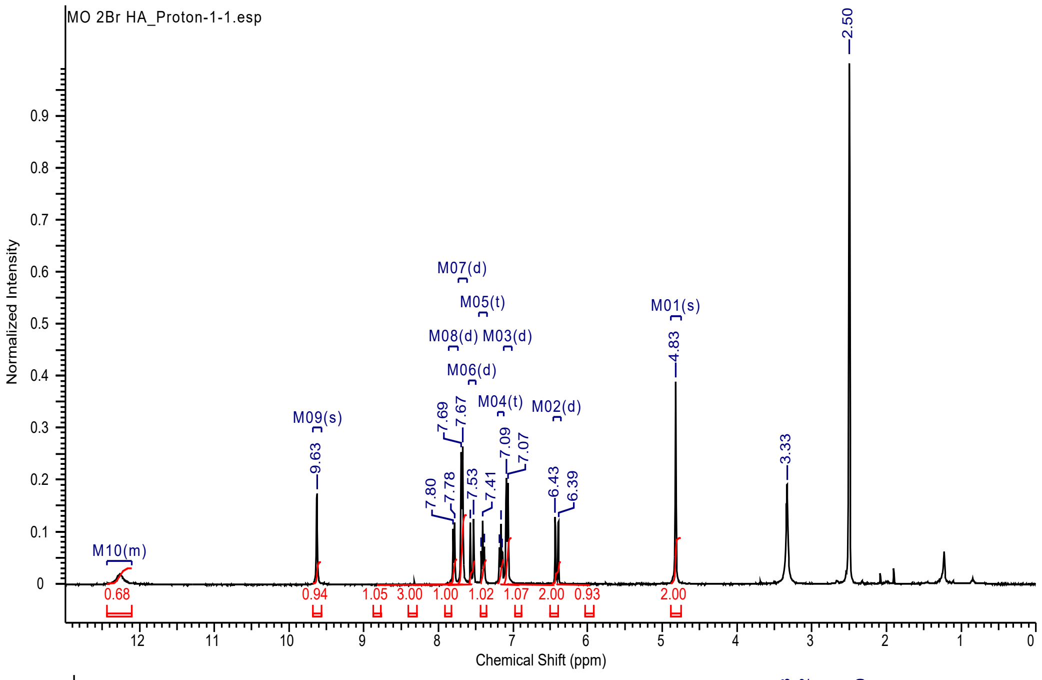

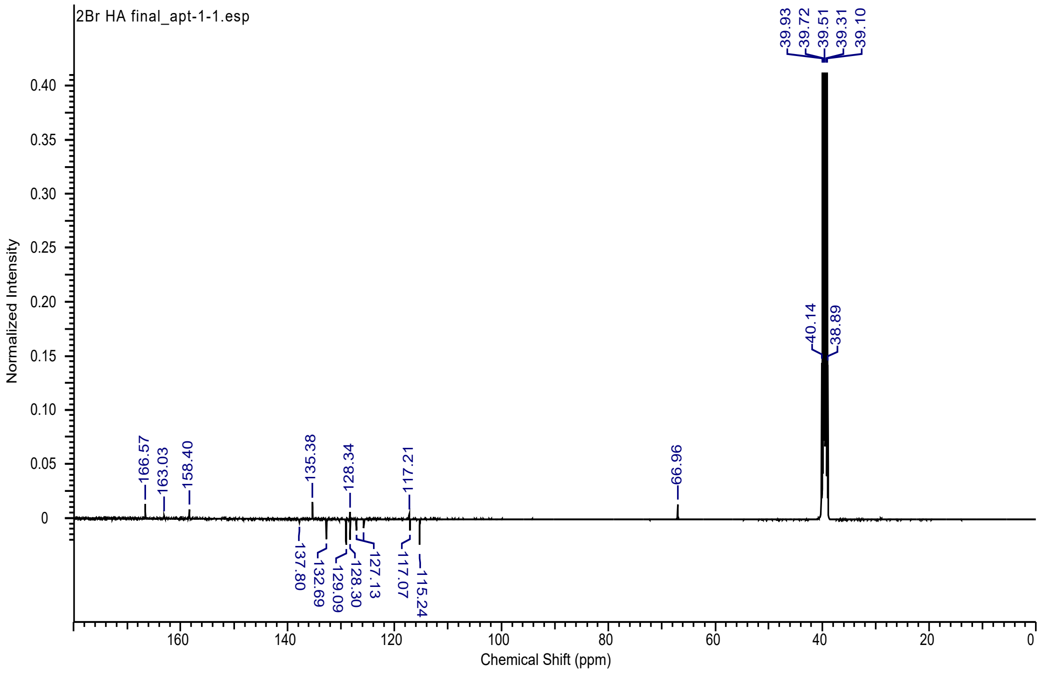

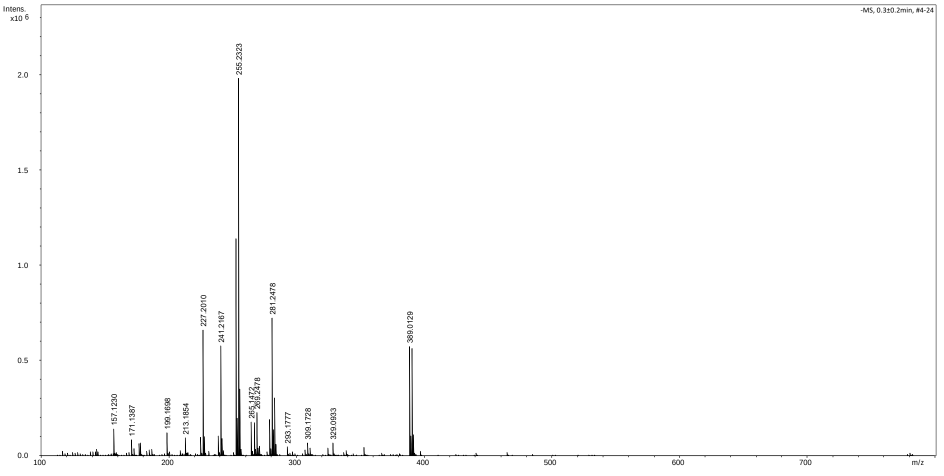

Chromatogram and Results

Injection Details

Injection Name:	**MO 2-Br HA**	*Run Time (min):*	**30,00**
Vial Number:	**BB6**	*Injection Volume:*	**10,00**
Injection Type:	**Unknown**	*Channel:*	**UV_VIS_1**
Calibration Level:		*Wavelength:*	**210,0**
Instrument Method:	**Grad40-60to90-10 MeCN-H2O**	*Bandwidth:*	**2**
Processing Method:	**New Processing Method**	*Dilution Factor:*	**1,0000**
Injection Date/Time:	**06.1.22 10:05**	*Sample Weight:*	**1,0000**

Chromatogram

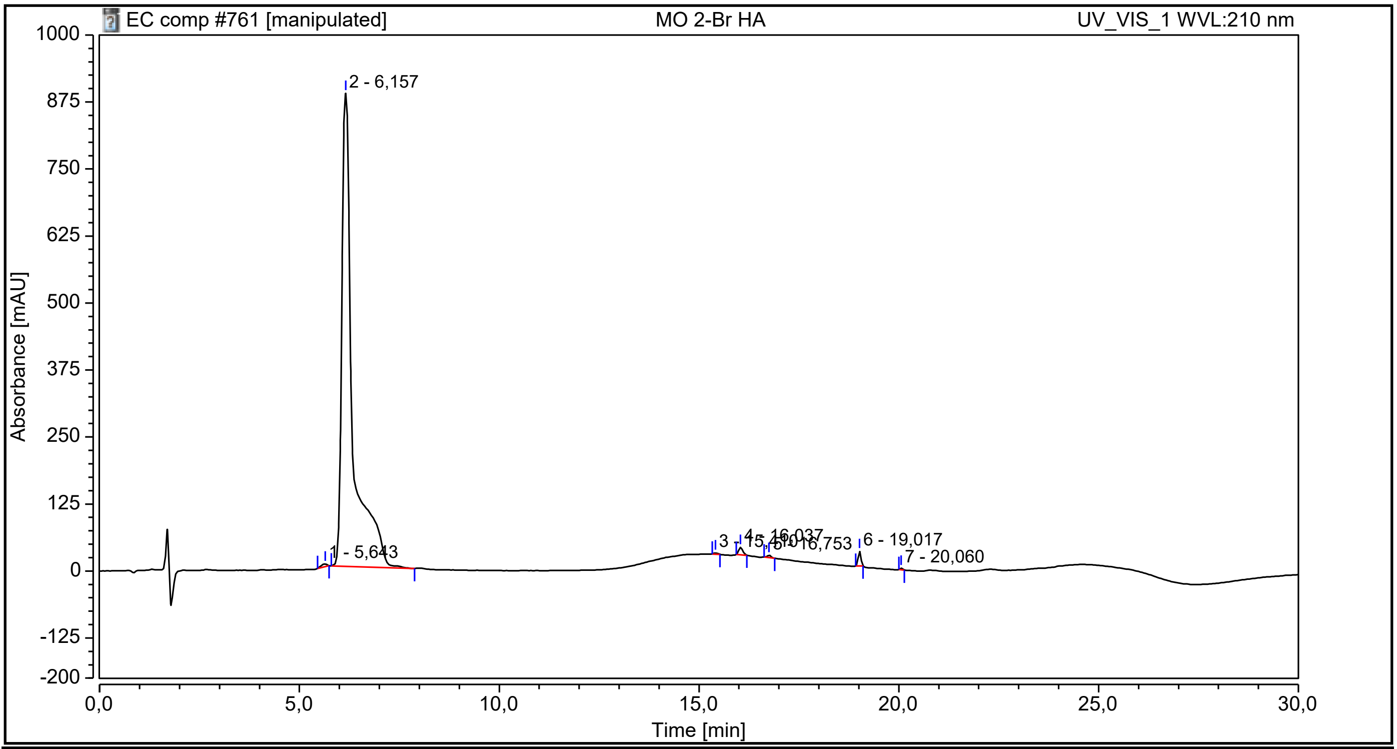

Integration Results

No.	Peak Name	Retention Time min	Area mAU*min	Height mAU	Relative Area %	Relative Height %	Amount n.a.
1	n.a.	5,643	0,874	4,742	0,304	0,51	n.a.
2	n.a.	6,157	281,245	882,292	98,018	94,15	n.a.
3	n.a.	15,410	0,232	2,193	0,081	0,23	n.a.
4	n.a.	16,037	1,695	13,724	0,591	1,46	n.a.
5	n.a.	16,753	0,440	4,011	0,153	0,43	n.a.
6	n.a.	19,017	2,244	27,329	0,782	2,92	n.a.
7	n.a.	20,060	0,202	2,780	0,071	0,30	n.a.
**Total:**			**286,931**	**937,071**	**100,00**	**100,00**	

(2*E*)-3-(4-{[(3-Bromophenyl)carbamoyl]methoxy}phenyl)-*N*-hydroxyprop-2-enamide (**7o**)

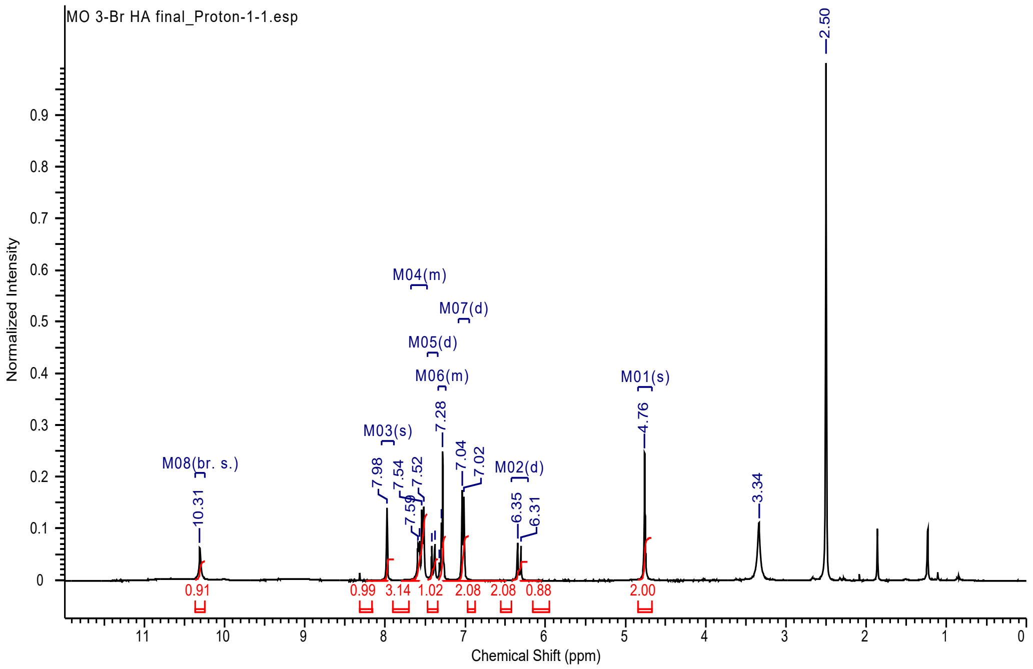

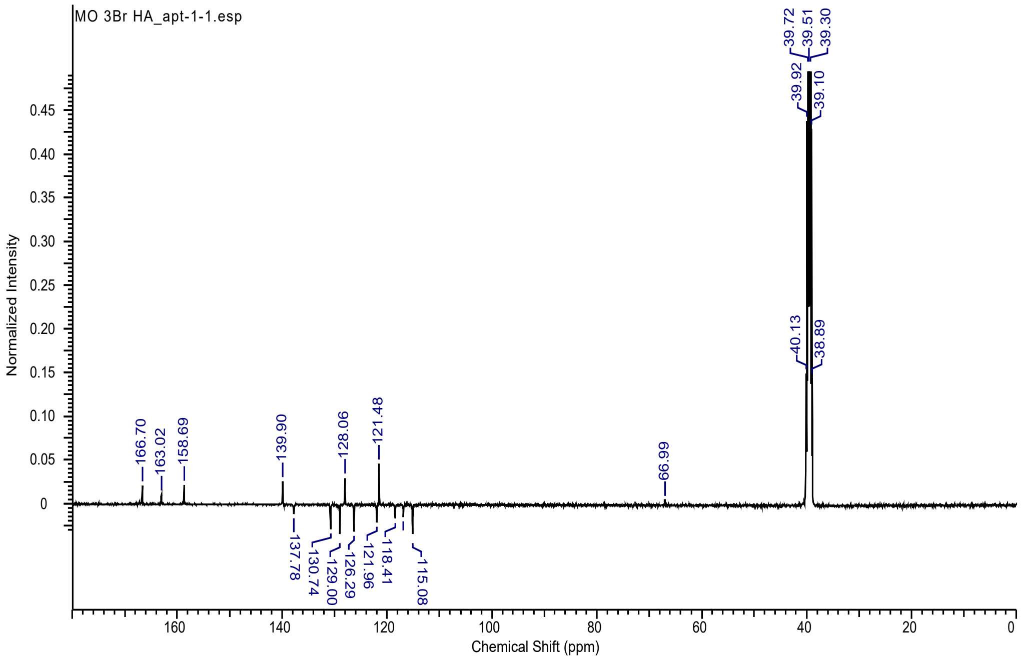

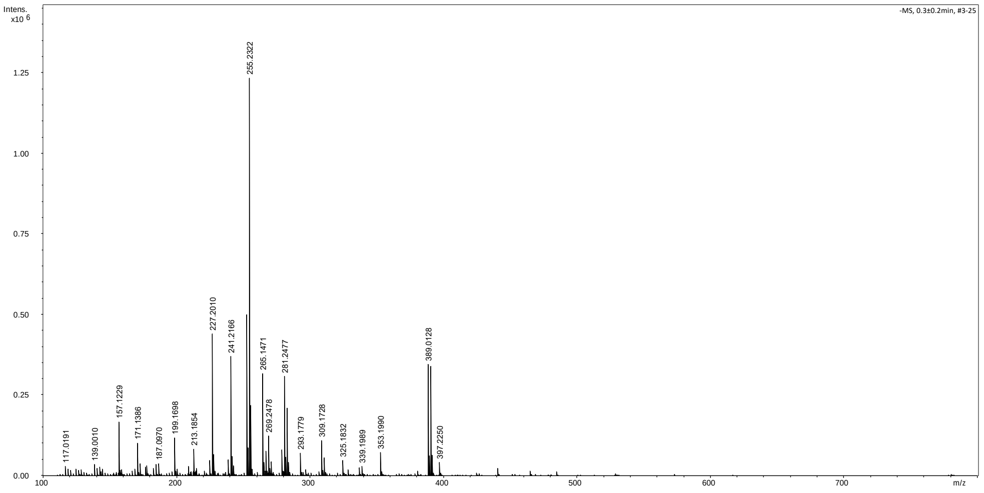

Chromatogram and Results

Injection Details

Injection Name:	**MO 3-Br HA**	*Run Time (min):*	**30,00**
Vial Number:	**BB5**	*Injection Volume:*	**10,00**
Injection Type:	**Unknown**	*Channel:*	**UV_VIS_1**
Calibration Level:		*Wavelength:*	**210,0**
Instrument Method:	**Grad40-60to90-10 MeCN-H2O**	*Bandwidth:*	**2**
Processing Method:	**New Processing Method**	*Dilution Factor:*	**1,0000**
Injection Date/Time:	**05.1.22 20:30**	*Sample Weight:*	**1,0000**

Chromatogram

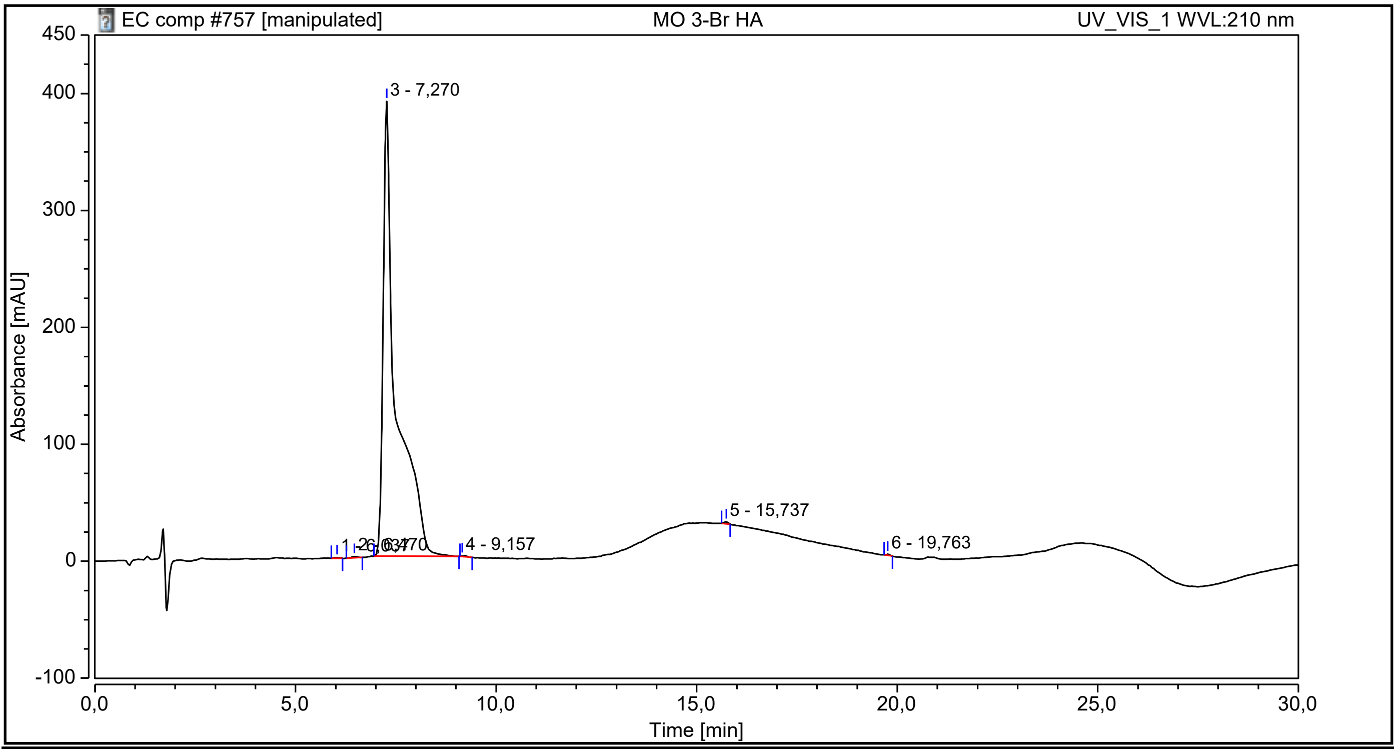

Integration Results

No.	Peak Name	Retention Time min	Area mAU*min	Height mAU	Relative Area %	Relative Height %	Amount n.a.
1	n.a.	6,037	0,084	0,604	0,057	0,15	n.a.
2	n.a.	6,470	0,201	1,195	0,137	0,30	n.a.
3	n.a.	7,270	146,496	388,896	99,538	98,69	n.a.
4	n.a.	9,157	0,105	0,651	0,071	0,17	n.a.
5	n.a.	15,737	0,194	1,677	0,132	0,43	n.a.
6	n.a.	19,763	0,095	1,033	0,065	0,26	n.a.
**Total:**			**147,175**	**394,056**	**100,00**	**100,00**	

(2*E*)-3-(4-{[(4-Bromophenyl)carbamoyl]methoxy}phenyl)-*N*-hydroxyprop-2-enamide (**7p**)

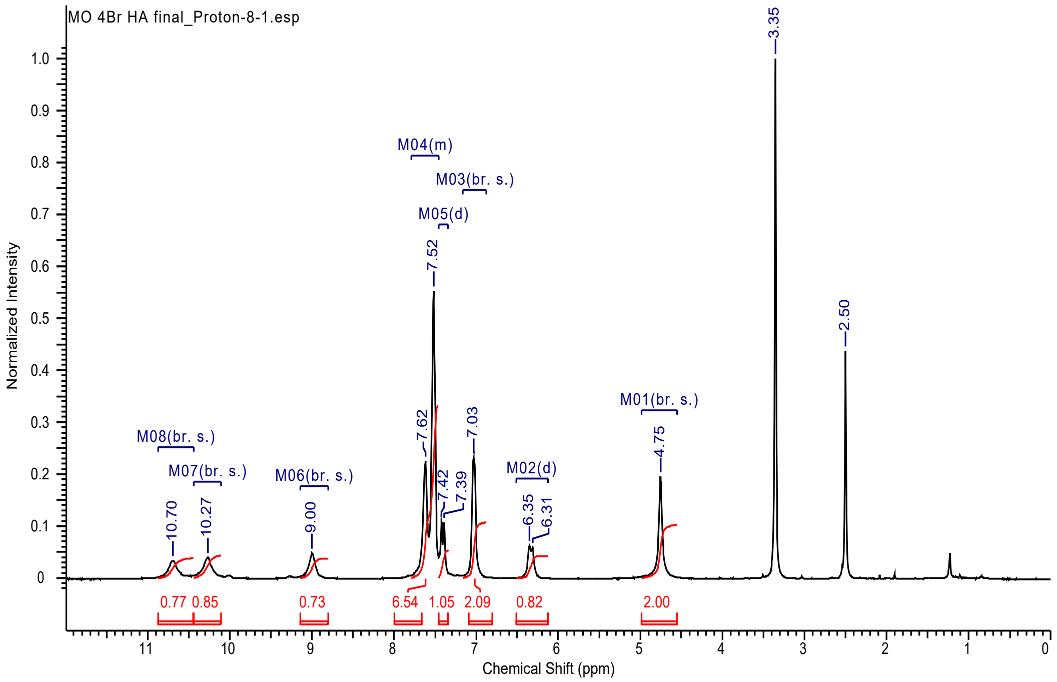

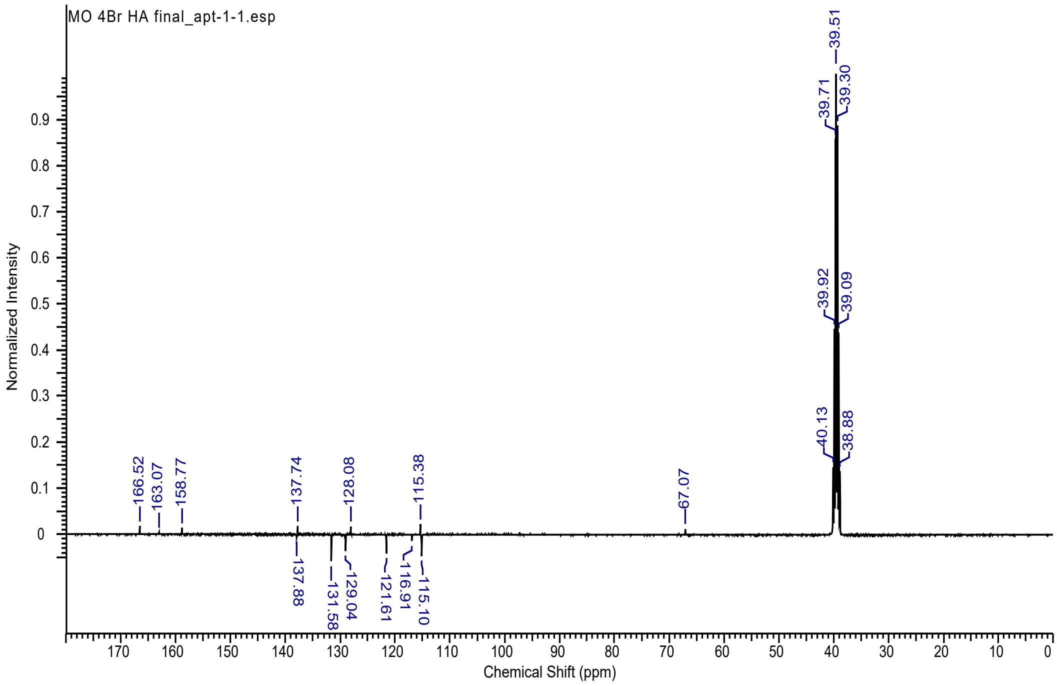

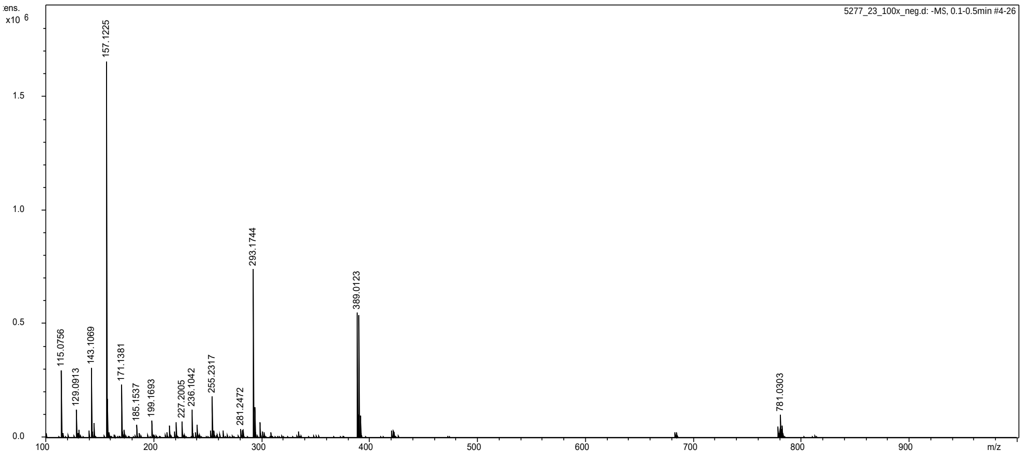

Chromatogram and Results

Injection Details

Injection Name:	**MO 4-Br HA**	*Run Time (min):*	**30,00**
Vial Number:	**BB4**	*Injection Volume:*	**10,00**
Injection Type:	**Unknown**	*Channel:*	**UV_VIS_1**
Calibration Level:		*Wavelength:*	**210,0**
Instrument Method:	**Grad40-60to90-10 MeCN-H2O**	*Bandwidth:*	**2**
Processing Method:	**New Processing Method**	*Dilution Factor:*	**1,0000**
Injection Date/Time:	**05.1.22 18:56**	*Sample Weight:*	**1,0000**

Chromatogram

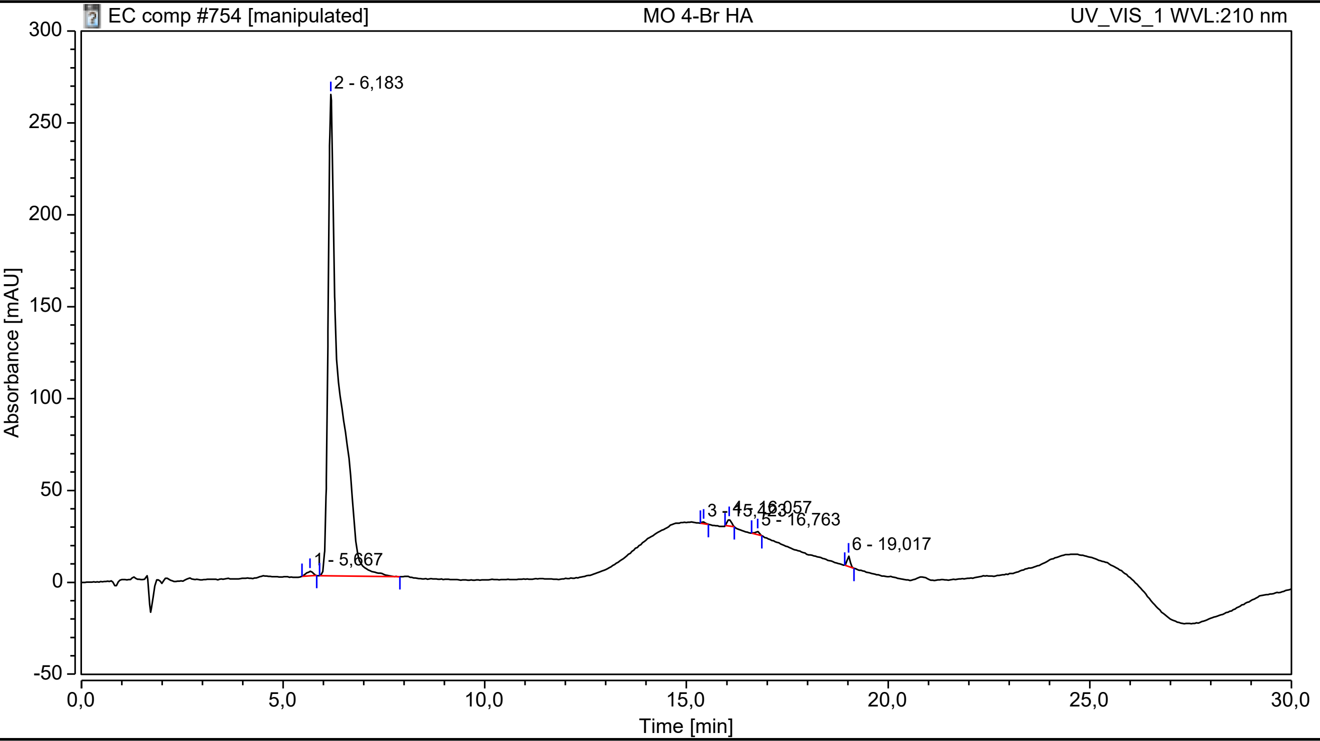

Integration Results

No.	Peak Name	Retention Time<br>min	Area<br>mAU*min	Height<br>mAU	Relative Area<br>%	Relative Height<br>%	Amount<br>n.a.
1	n.a.	5,667	0,458	2,512	0,526	0,91	n.a.
2	n.a.	6,183	85,284	261,939	98,070	94,71	n.a.
3	n.a.	15,423	0,093	0,951	0,107	0,34	n.a.
4	n.a.	16,057	0,438	3,715	0,503	1,34	n.a.
5	n.a.	16,763	0,193	1,740	0,222	0,63	n.a.
6	n.a.	19,017	0,498	5,710	0,572	2,06	n.a.
**Total:**			**86,963**	**276,567**	**100,00**	**100,00**	

(2*E*)-*N*-Hydroxy-3-[4-({[2-(trifluoromethyl)phenyl]carbamoyl}methoxy)phenyl]prop-2-enamide (**7q**)

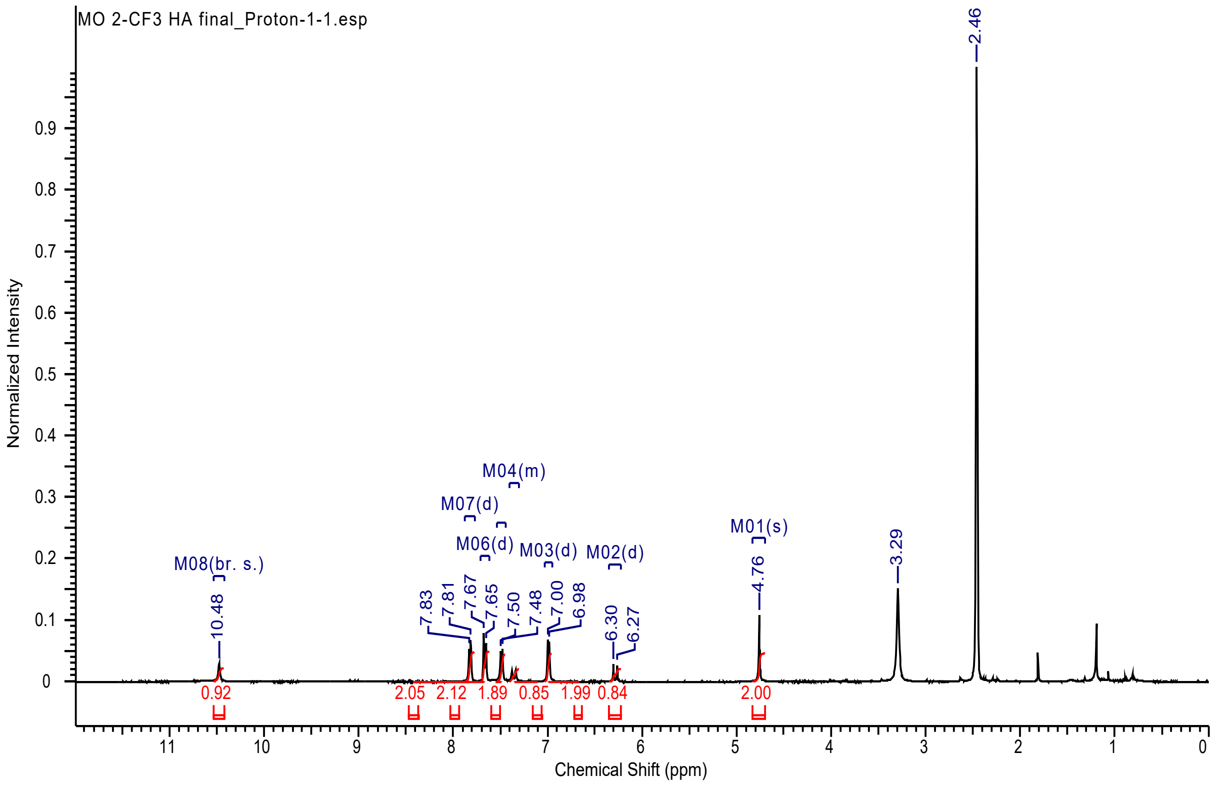

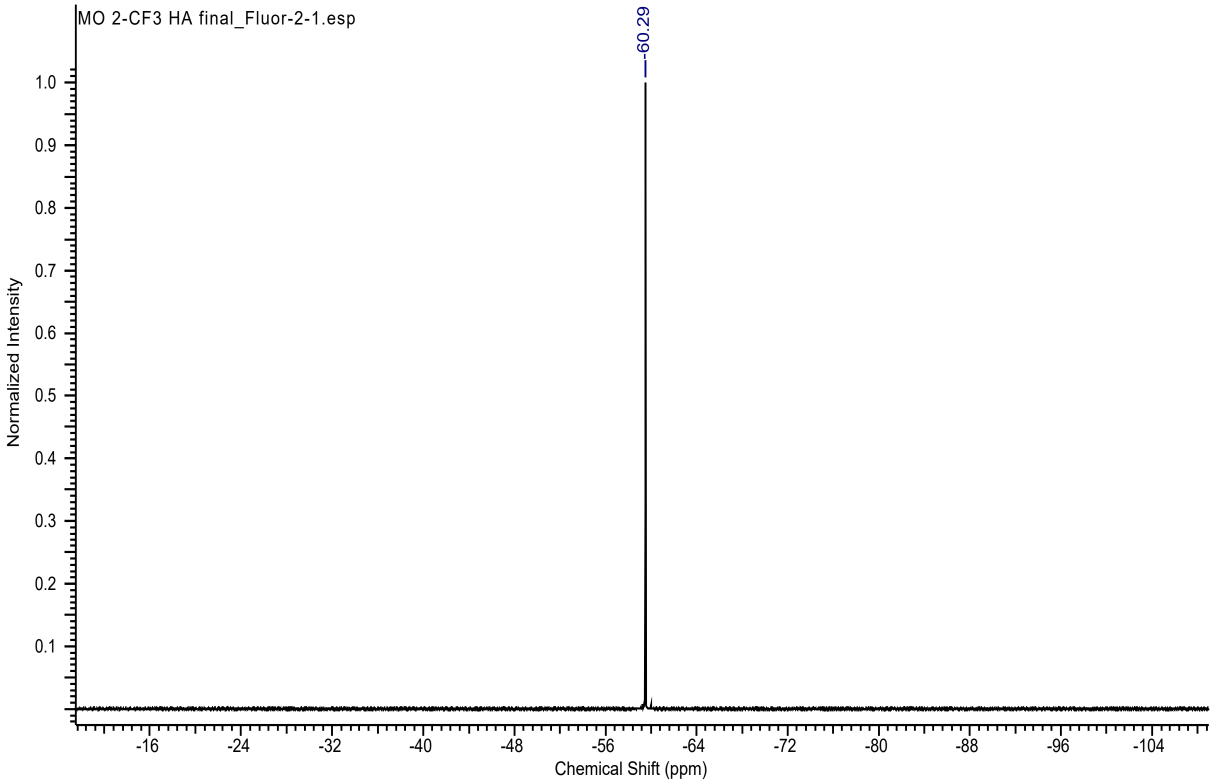

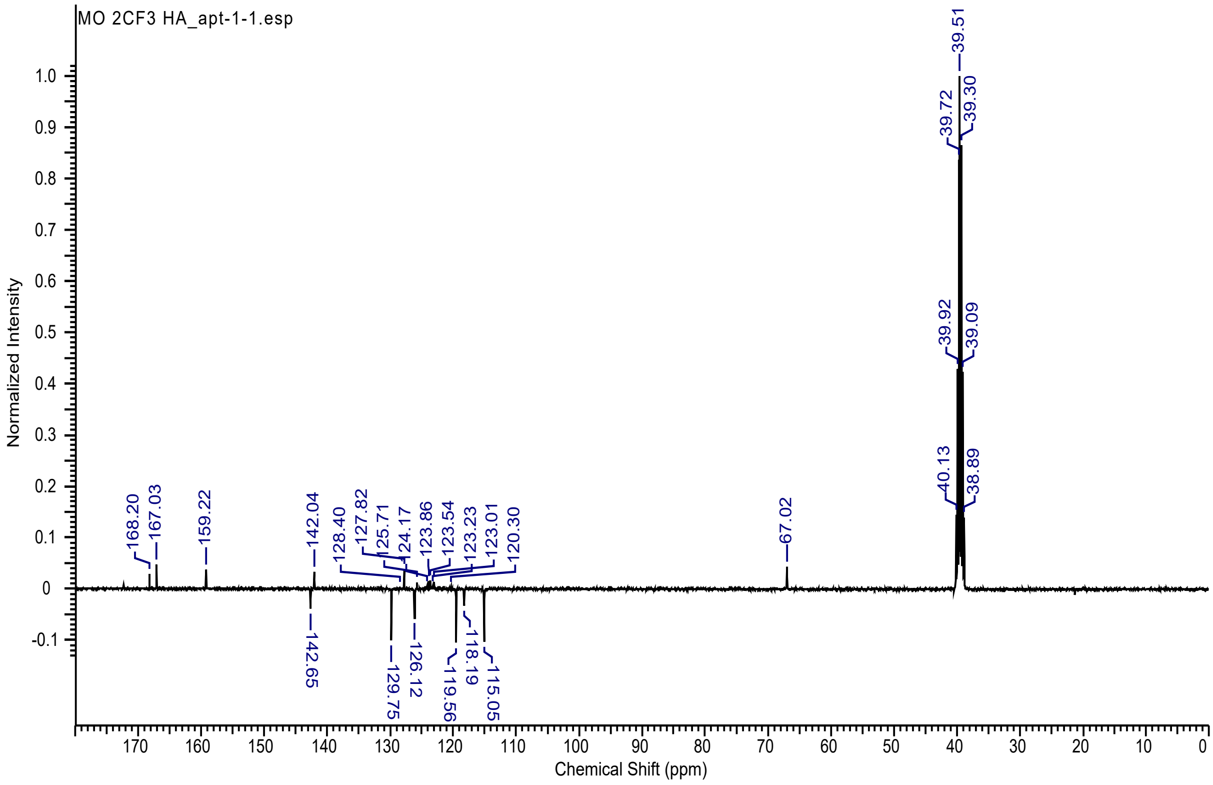

MO 2CF3 HA_apt-1-1.esp
Normalized Intensity
Chemical Shift (ppm)
168.20
167.03
159.22
142.04
128.40
127.82
125.71
124.17
123.86
123.54
123.23
123.01
120.30
67.02
40.13
39.92
39.72
39.51
39.30
39.09
38.89
142.65
129.75
126.12
119.56
118.19
115.05

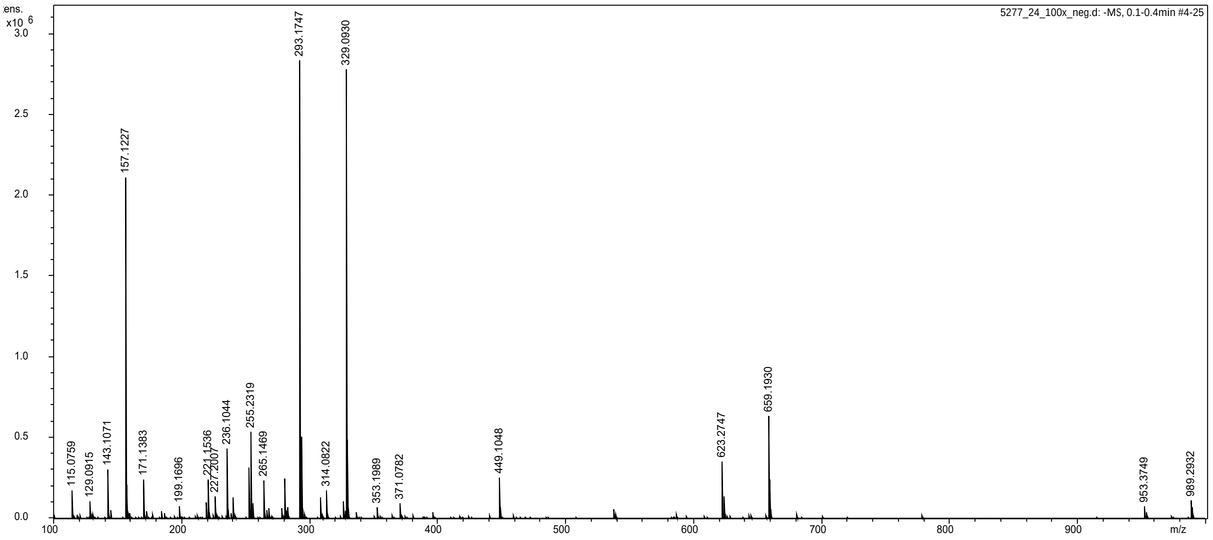

5277_24_100x_neg.d: -MS, 0.1-0.4min #4-25
m/z
115.0759
129.0915
143.1071
157.1227
171.1383
199.1696
221.1536
227.2007
236.1044
255.2319
265.1469
293.1747
314.0822
329.0930
353.1989
371.0782
449.1048
623.2747
659.1930
953.3749
989.2932

Chromatogram and Results

Injection Details

Injection Name:	**MO 2-CF3 HA**	*Run Time (min):*	**30,00**
Vial Number:	**BE1**	*Injection Volume:*	**10,00**
Injection Type:	**Unknown**	*Channel:*	**UV_VIS_1**
Calibration Level:		*Wavelength:*	**210,0**
Instrument Method:	**Grad40-60to90-10 MeCN-H2O**	*Bandwidth:*	**2**
Processing Method:	**New Processing Method**	*Dilution Factor:*	**1,0000**
Injection Date/Time:	**12.1.22 15:29**	*Sample Weight:*	**1,0000**

Chromatogram

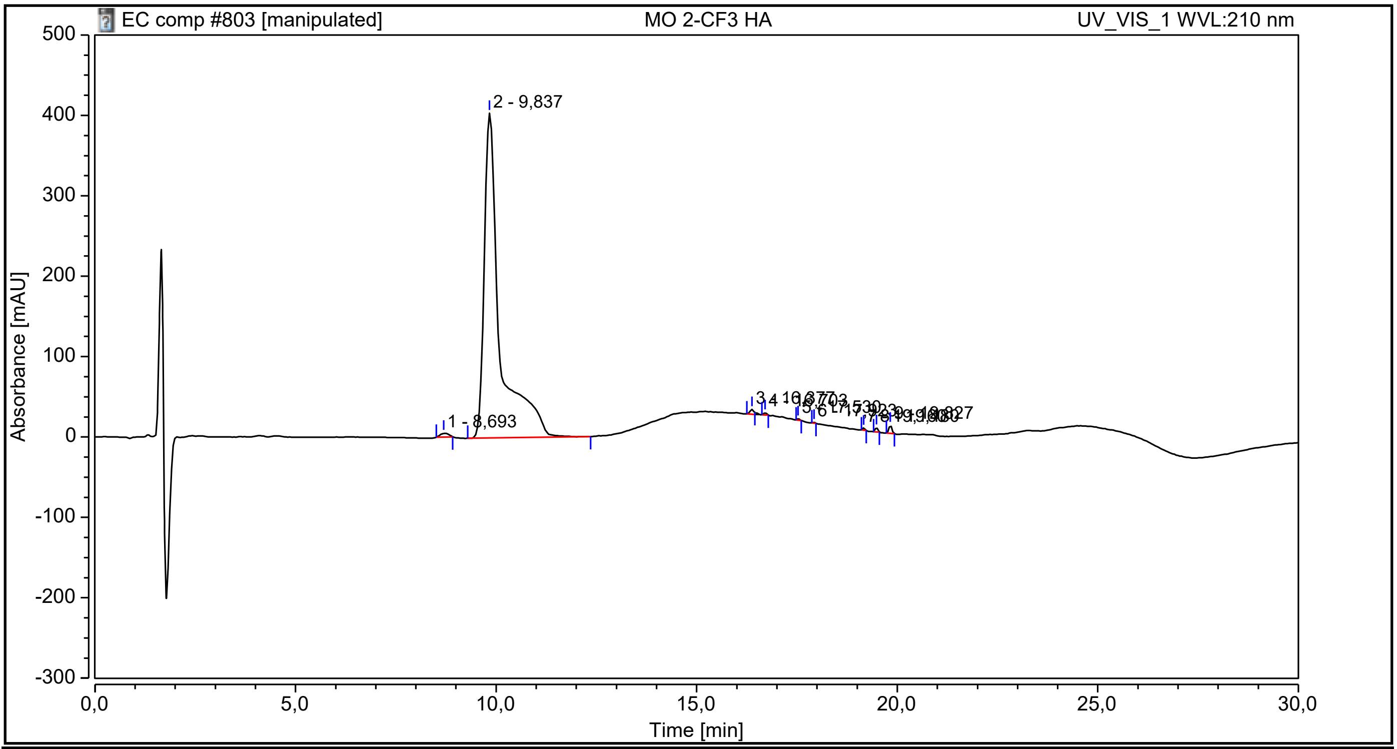

Integration Results

No.	Peak Name	Retention Time min	Area mAU*min	Height mAU	Relative Area %	Relative Height %	Amount n.a.
1	n.a.	8,693	1,145	4,847	0,614	1,10	n.a.
2	n.a.	9,837	182,734	403,873	98,028	92,04	n.a.
3	n.a.	16,377	0,641	5,845	0,344	1,33	n.a.
4	n.a.	16,703	0,252	3,056	0,135	0,70	n.a.
5	n.a.	17,530	0,140	1,709	0,075	0,39	n.a.
6	n.a.	17,923	0,065	1,015	0,035	0,23	n.a.
7	n.a.	19,160	0,164	2,594	0,088	0,59	n.a.
8	n.a.	19,480	0,387	5,318	0,208	1,21	n.a.
9	n.a.	19,827	0,881	10,563	0,473	2,41	n.a.
**Total:**			**186,410**	**438,820**	**100,00**	**100,00**	

(2*E*)-*N*-Hydroxy-3-[4-({[3-(trifluoromethyl)phenyl]carbamoyl}methoxy)phenyl]prop-2-enamide (**7r**)

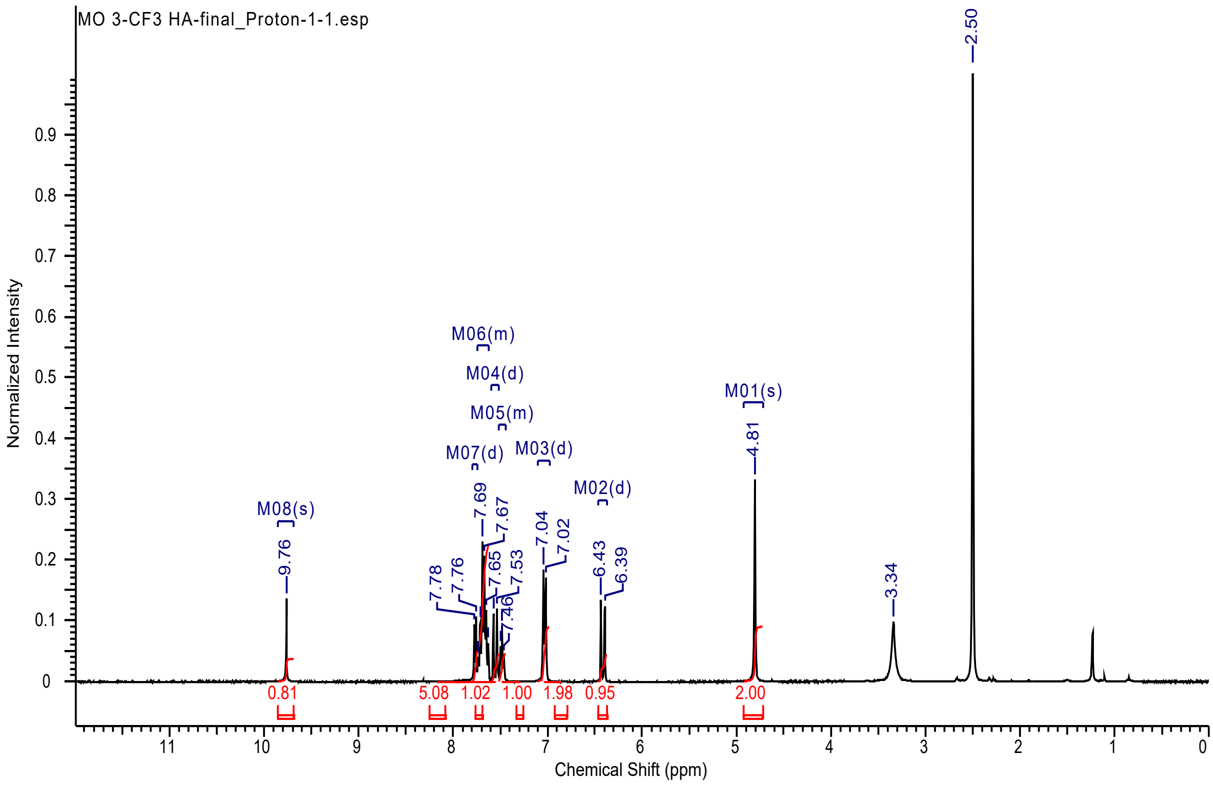

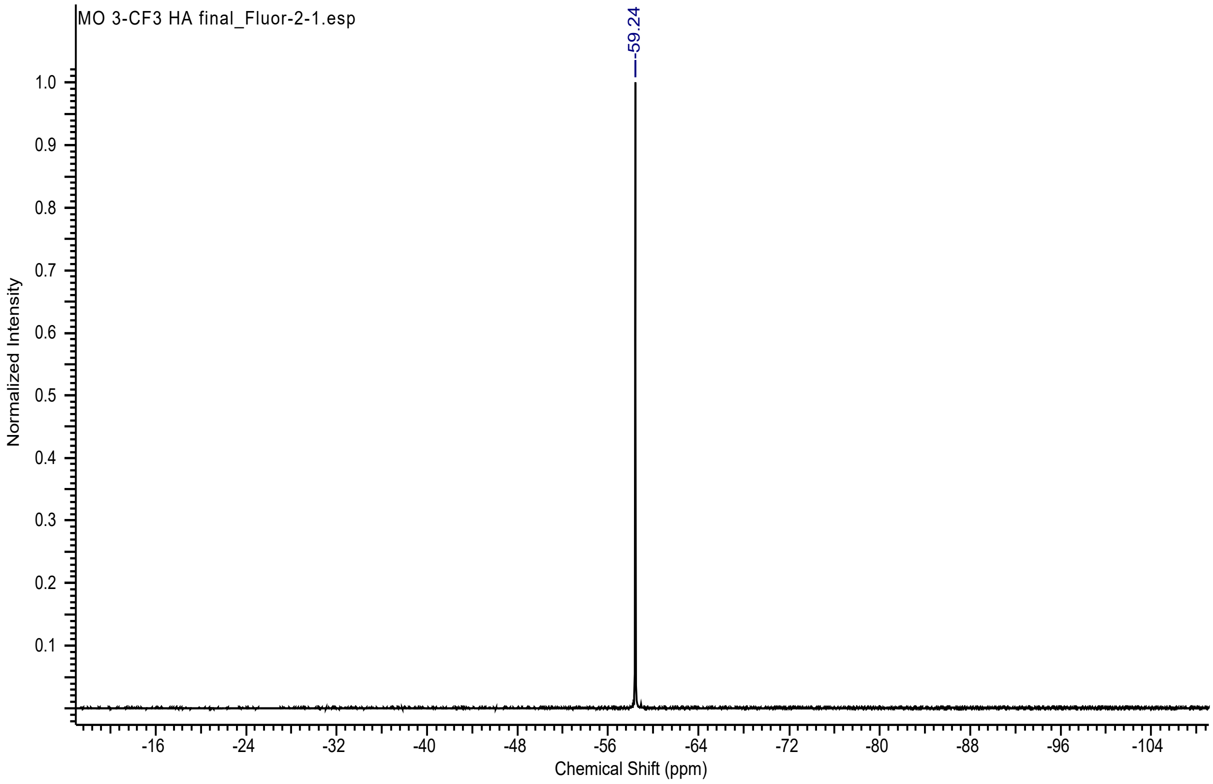

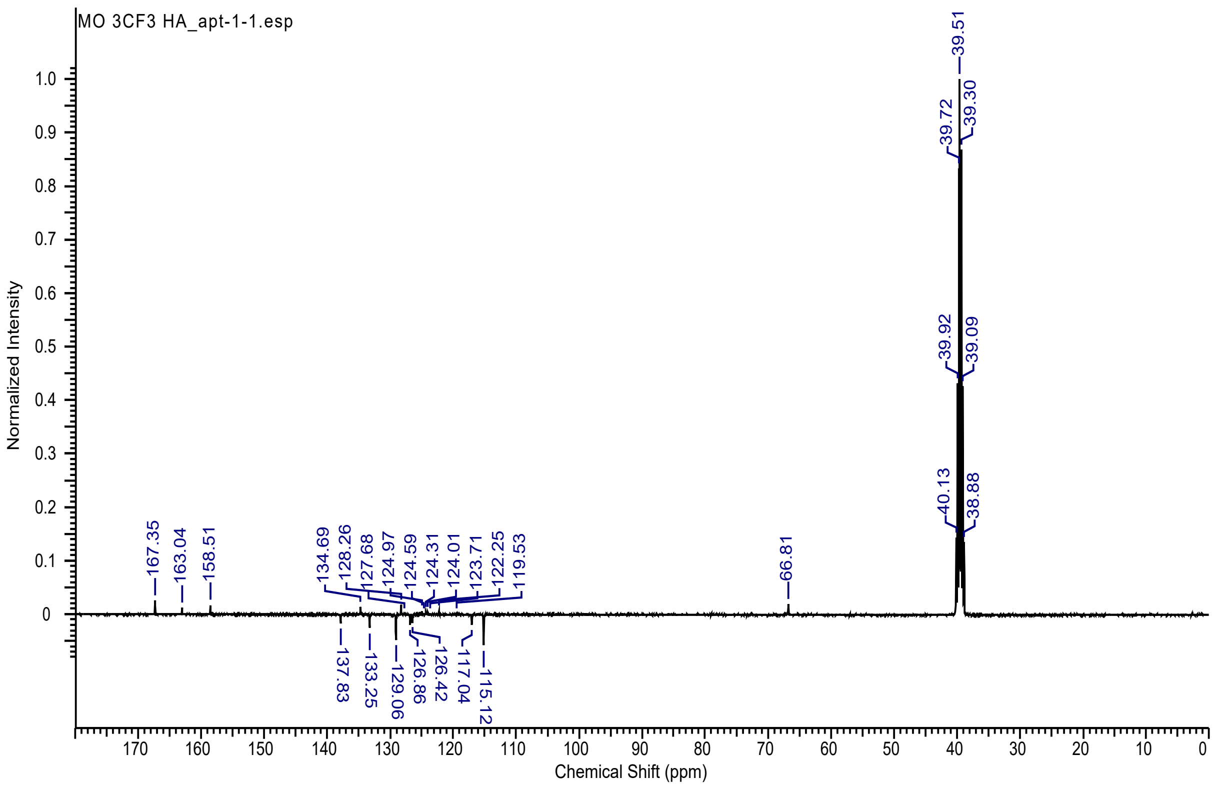
MO 3CF3 HA_apt-1-1.esp
Normalized Intensity
Chemical Shift (ppm)
167.35
163.04
158.51
134.69
128.26
127.68
124.97
124.59
124.31
124.01
123.71
122.25
119.53
66.81
39.51
39.72
39.30
39.92
39.09
40.13
38.88
137.83
133.25
129.06
126.86
126.42
117.04
115.12

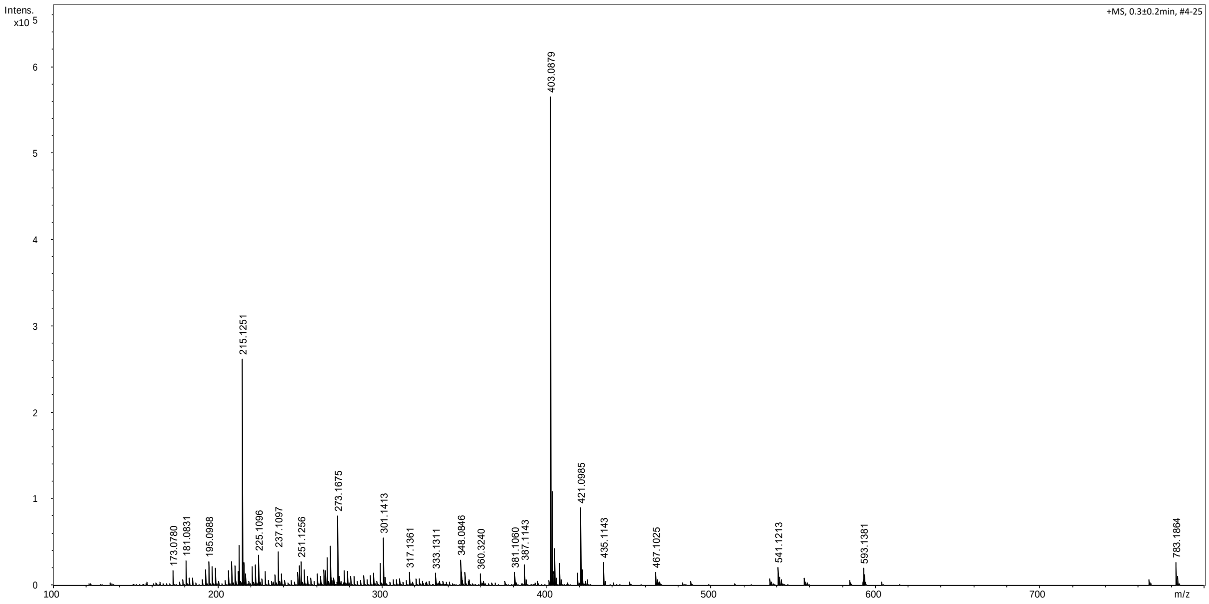
Intens.
x10 5
+MS, 0.3±0.2min, #4-25
403.0879
215.1251
273.1675
301.1413
421.0985
173.0780
181.0831
195.0988
225.1096
237.1097
251.1256
317.1361
333.1311
348.0846
360.3240
381.1060
387.1143
435.1143
467.1025
541.1213
593.1381
783.1864
m/z

Chromatogram and Results

Injection Details

Injection Name:	**MO 3-CF3 HA**	*Run Time (min):*	**30,00**
Vial Number:	**BB2**	*Injection Volume:*	**10,00**
Injection Type:	**Unknown**	*Channel:*	**UV_VIS_1**
Calibration Level:		*Wavelength:*	**210,0**
Instrument Method:	**Grad40-60to90-10 MeCN-H2O**	*Bandwidth:*	**2**
Processing Method:	**New Processing Method**	*Dilution Factor:*	**1,0000**
Injection Date/Time:	**05.1.22 15:16**	*Sample Weight:*	**1,0000**

Chromatogram

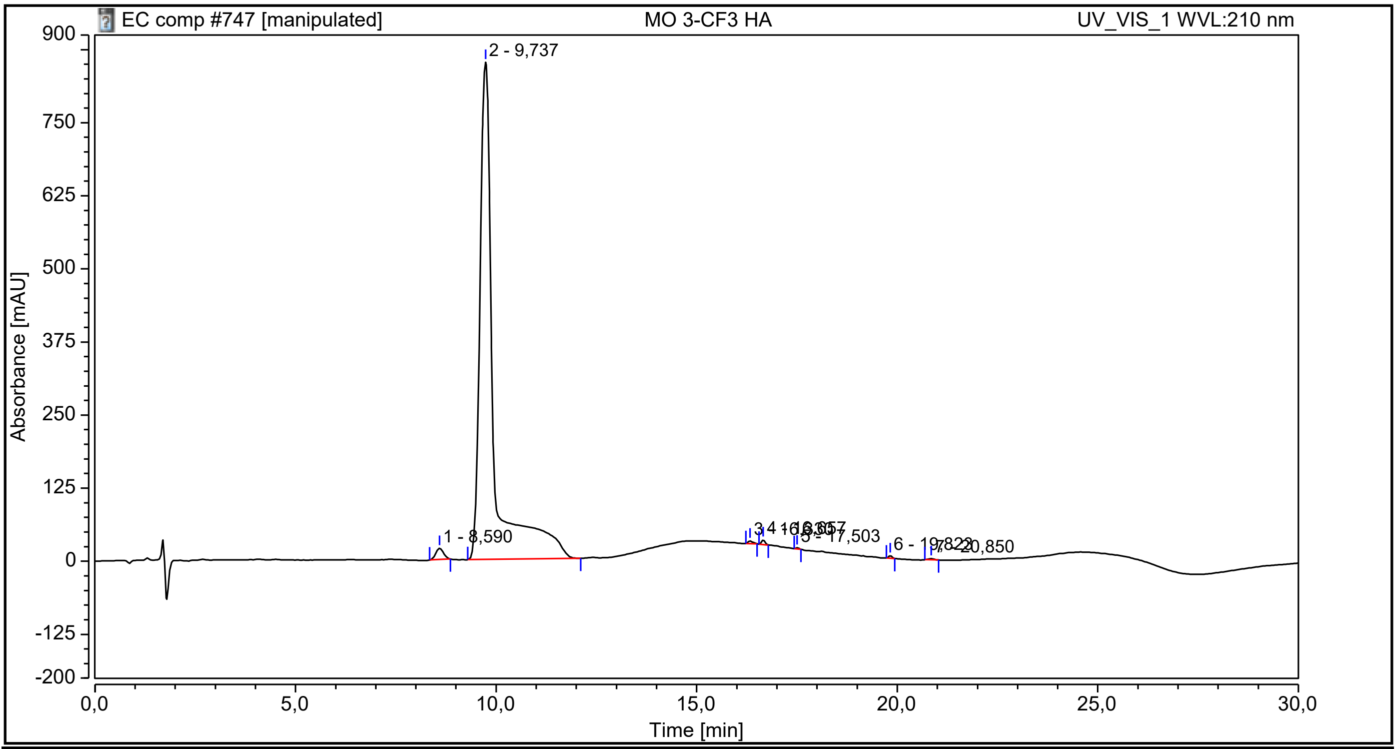

Integration Results

No.	Peak Name	Retention Time min	Area mAU*min	Height mAU	Relative Area %	Relative Height %	Amount n.a.
1	n.a.	8,590	4,226	19,095	1,219	2,14	n.a.
2	n.a.	9,737	340,210	850,479	98,100	95,45	n.a.
3	n.a.	16,330	0,614	4,753	0,177	0,53	n.a.
4	n.a.	16,657	0,804	8,138	0,232	0,91	n.a.
5	n.a.	17,503	0,168	1,907	0,049	0,21	n.a.
6	n.a.	19,823	0,427	4,591	0,123	0,52	n.a.
7	n.a.	20,850	0,349	2,105	0,101	0,24	n.a.
**Total:**			**346,799**	**891,067**	**100,00**	**100,00**	

(2*E*)-*N*-Hydroxy-3-[4-({[4-(trifluoromethyl)phenyl]carbamoyl}methoxy)phenyl]prop-2-enamide (**7s**)

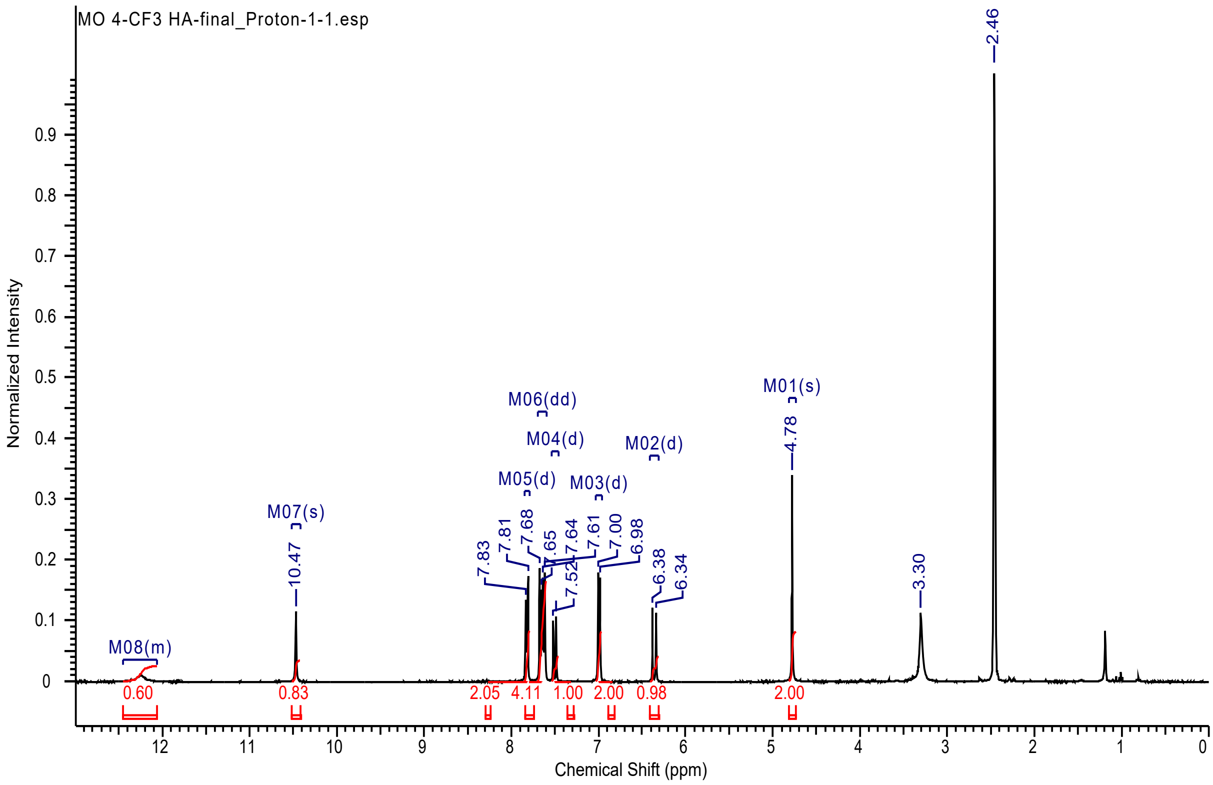

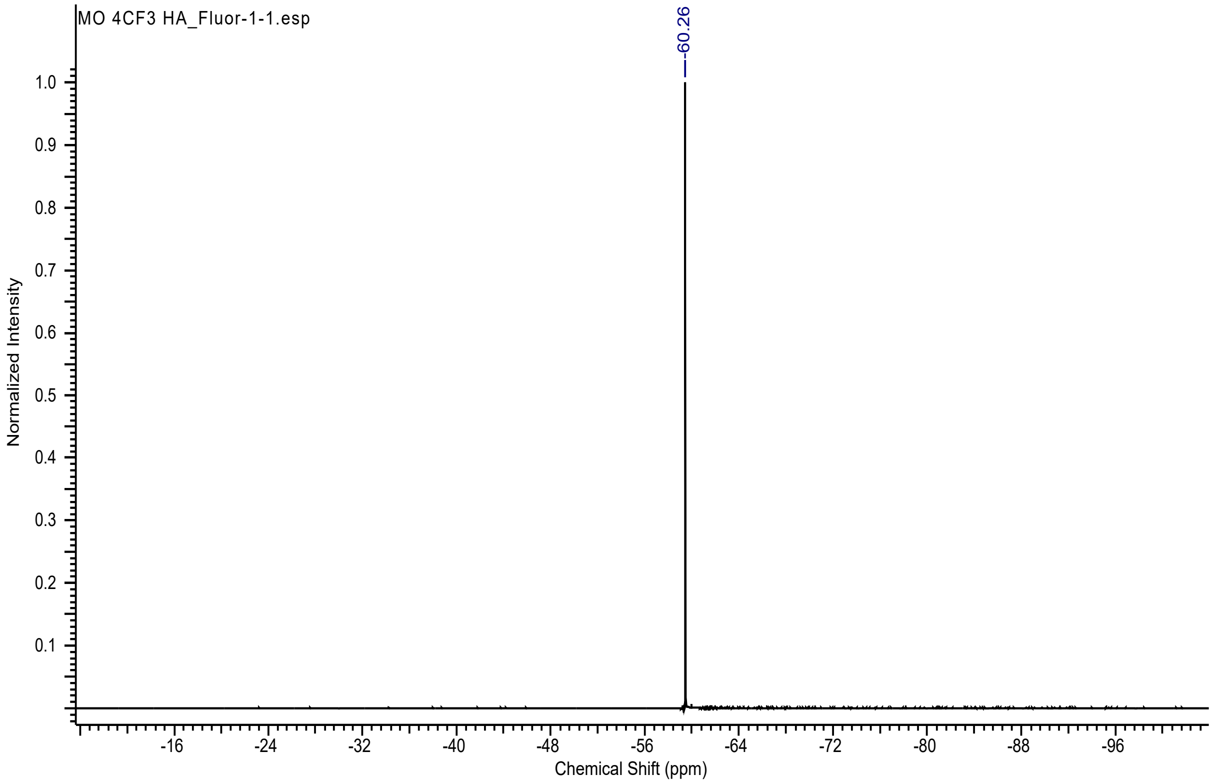

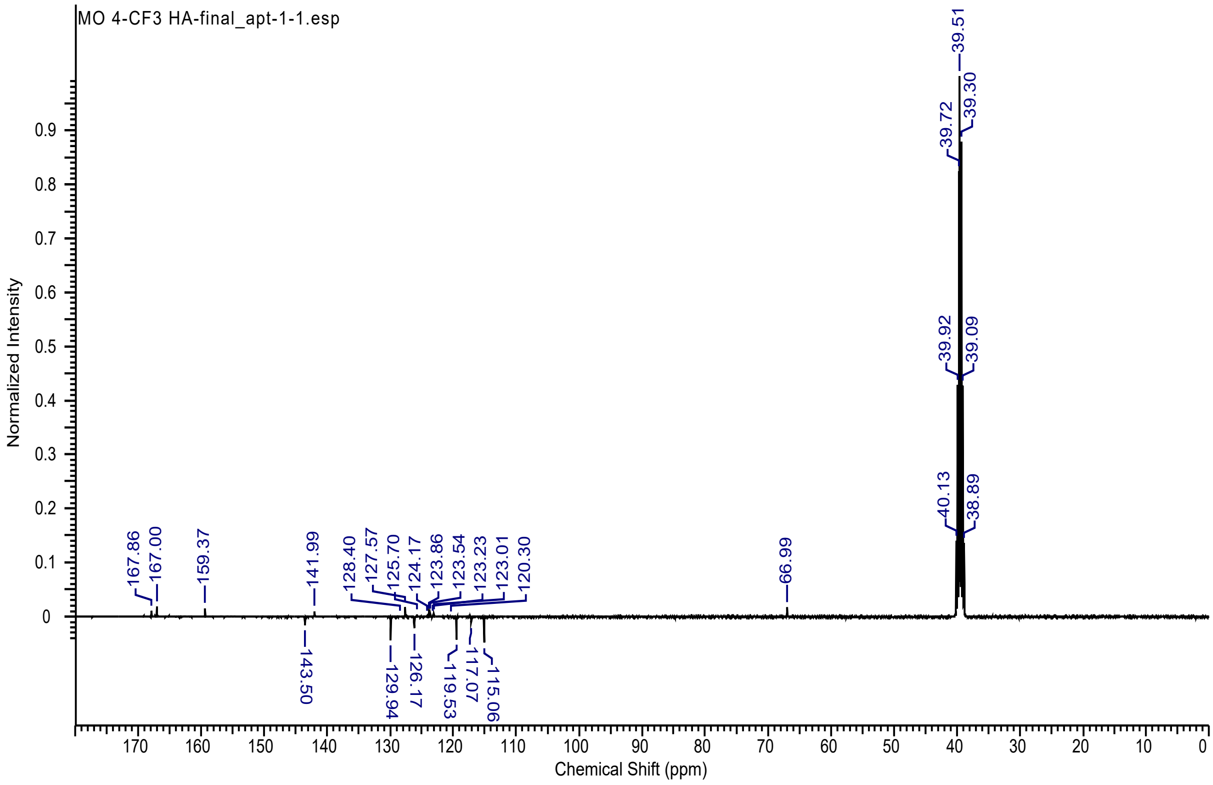
MO 4-CF3 HA-final_apt-1-1.esp
Normalized Intensity
Chemical Shift (ppm)
167.86
167.00
159.37
141.99
128.40
127.57
125.70
124.17
123.86
123.54
123.23
123.01
120.30
66.99
39.51
39.30
39.72
39.92
39.09
40.13
38.89
143.50
129.94
126.17
119.53
117.07
115.06

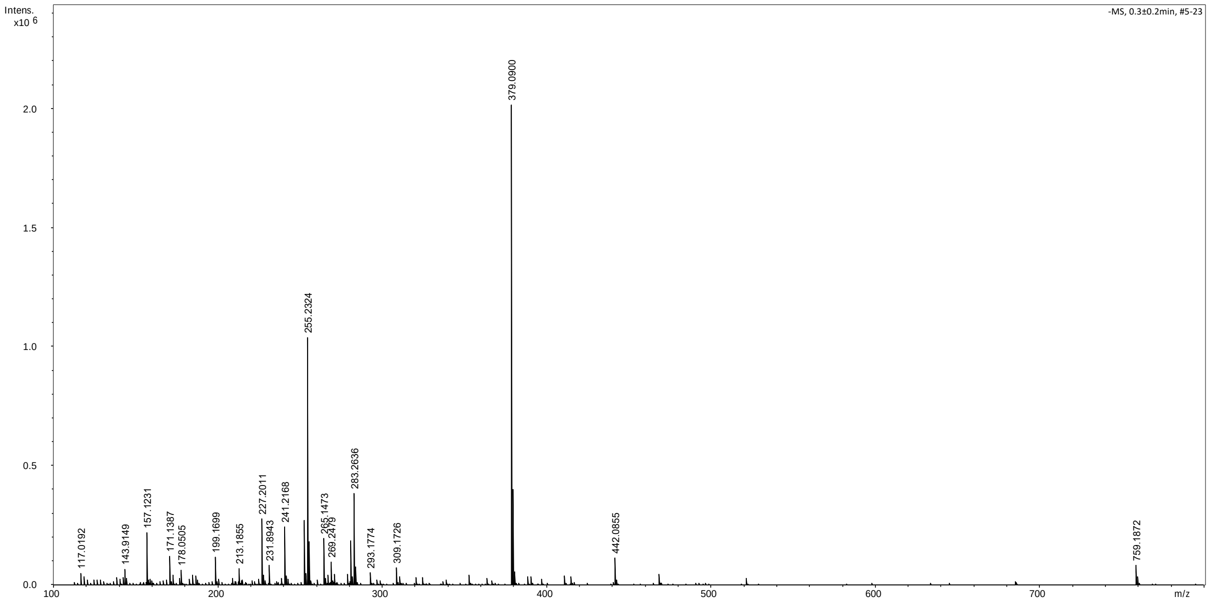
Intens.
x10 6
-MS, 0.3±0.2min, #5-23
379.0900
255.2324
283.2636
117.0192
143.9149
157.1231
171.1387
178.0505
199.1699
213.1855
227.2011
231.8943
241.2168
265.1473
269.2479
293.1774
309.1726
442.0855
759.1872
m/z

Chromatogram and Results

Injection Details

Injection Name:	**MO 4-CF3 HA**	*Run Time (min):*	**30,00**
Vial Number:	**BB3**	*Injection Volume:*	**10,00**
Injection Type:	**Unknown**	*Channel:*	**UV_VIS_1**
Calibration Level:		*Wavelength:*	**210,0**
Instrument Method:	**Grad40-60to90-10 MeCN-H2O**	*Bandwidth:*	**2**
Processing Method:	**New Processing Method**	*Dilution Factor:*	**1,0000**
Injection Date/Time:	**05.1.22 17:53**	*Sample Weight:*	**1,0000**

Chromatogram

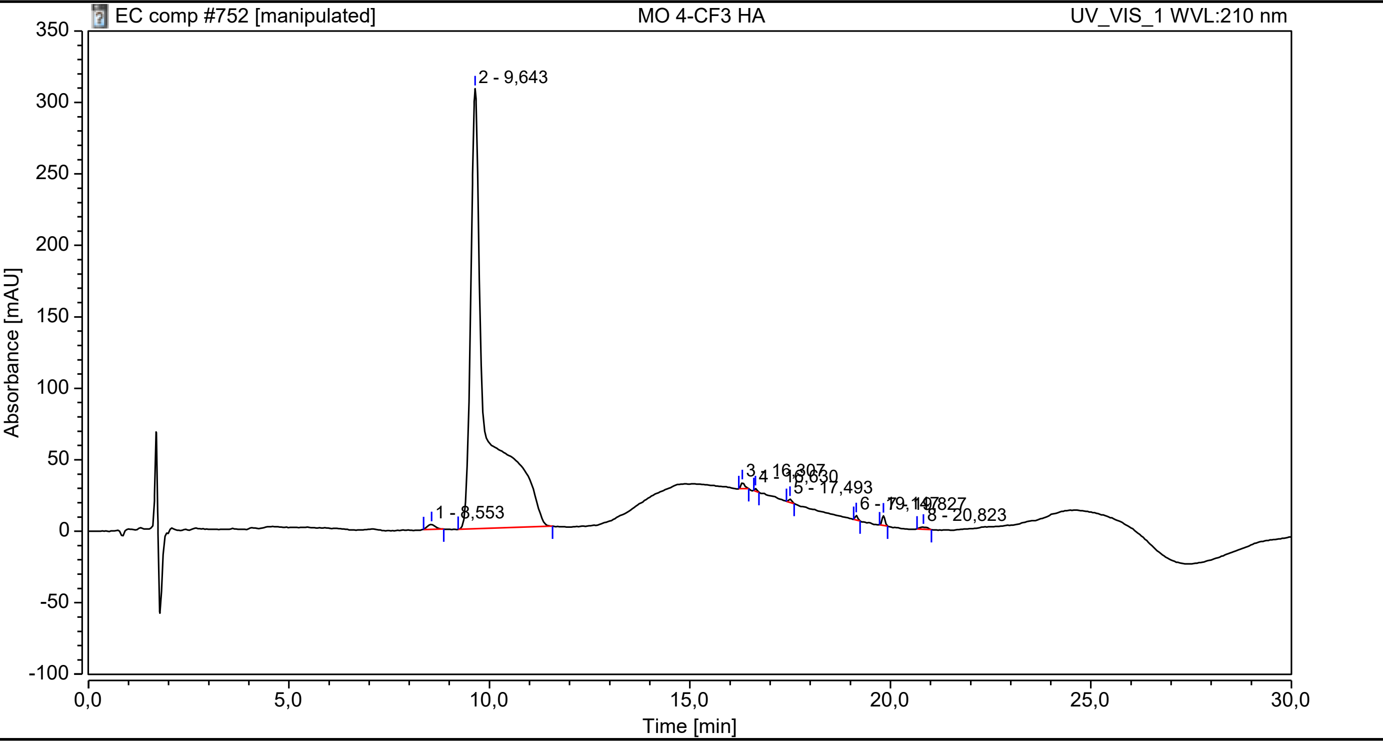

Integration Results

No.	Peak Name	Retention Time min	Area mAU*min	Height mAU	Relative Area %	Relative Height %	Amount n.a.
1	n.a.	8,553	0,778	3,470	0,538	1,05	n.a.
2	n.a.	9,643	141,731	307,843	98,109	93,01	n.a.
3	n.a.	16,307	0,471	4,308	0,326	1,30	n.a.
4	n.a.	16,630	0,135	1,702	0,093	0,51	n.a.
5	n.a.	17,493	0,206	2,300	0,143	0,69	n.a.
6	n.a.	19,147	0,248	3,156	0,172	0,95	n.a.
7	n.a.	19,827	0,561	6,716	0,389	2,03	n.a.
8	n.a.	20,823	0,333	1,489	0,230	0,45	n.a.
**Total:**			**144,463**	**330,985**	**100,00**	**100,00**	

(2*E*)-*N*-Hydroxy-3-(4-{[(2-nitrophenyl)carbamoyl]methoxy}phenyl)prop-2-enamide (**7t**)

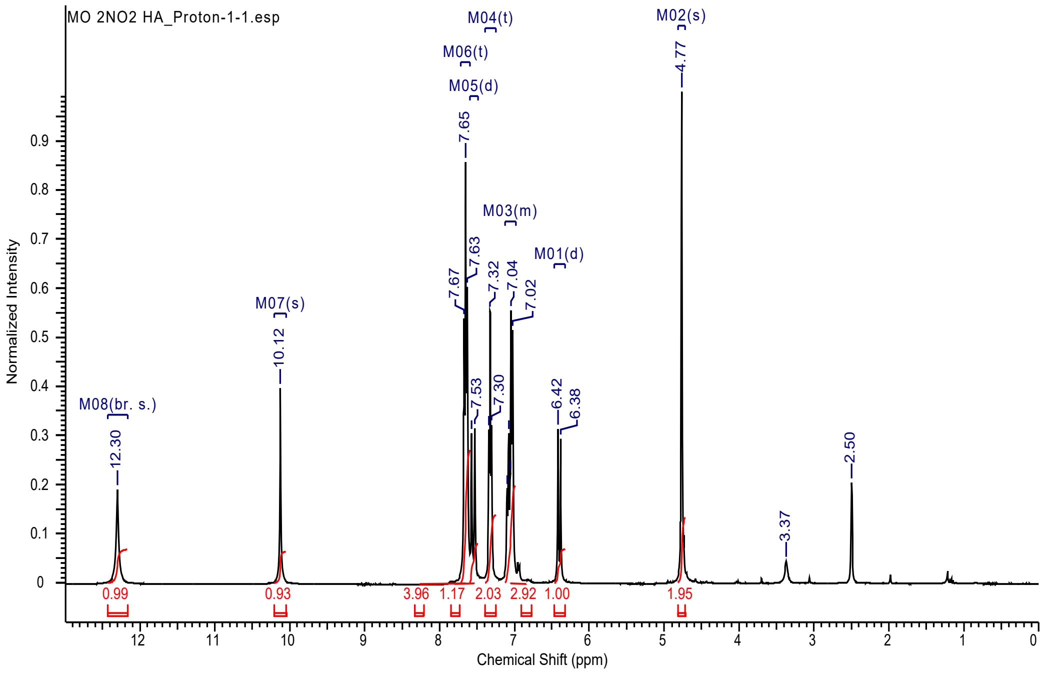

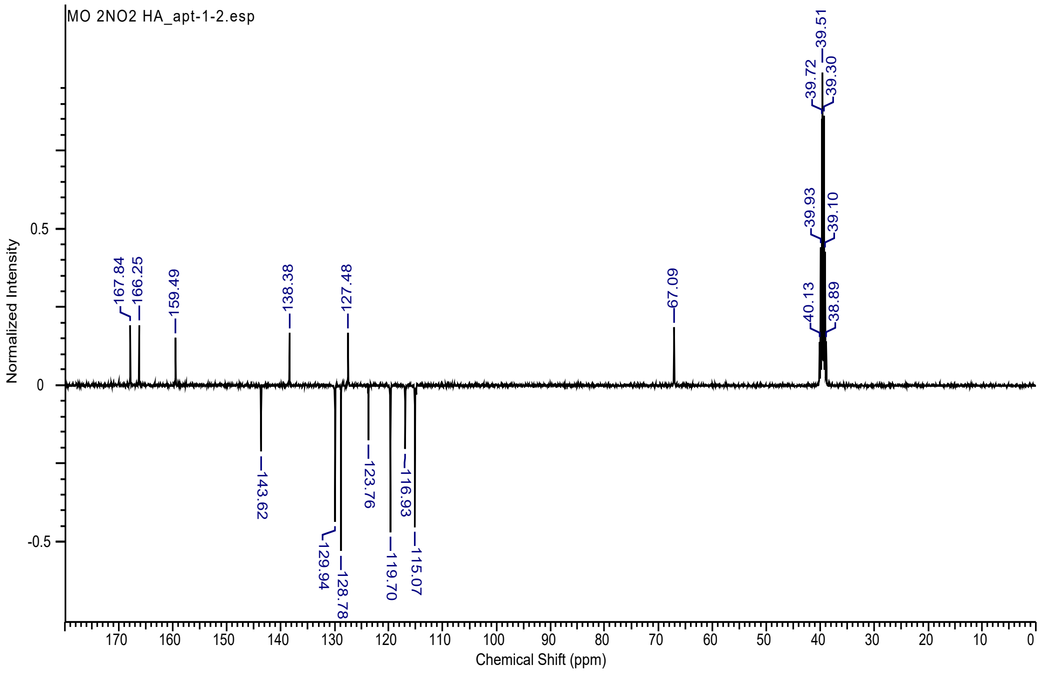

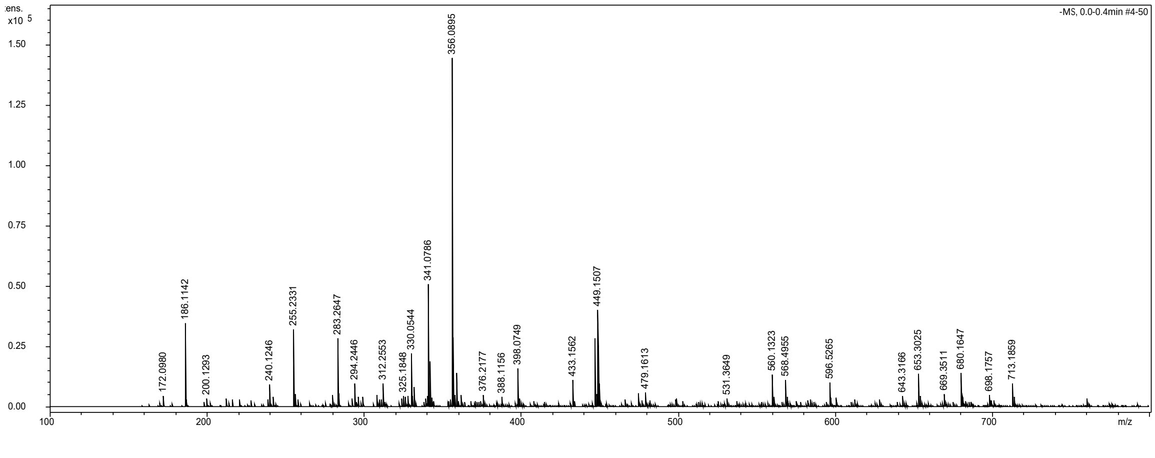

Chromatogram and Results

Injection Details

Injection Name:	**MO 2-NO2 HA**	*Run Time (min):*	**30,00**
Vial Number:	**BC7**	*Injection Volume:*	**5,00**
Injection Type:	**Unknown**	*Channel:*	**UV_VIS_1**
Calibration Level:		*Wavelength:*	**210,0**
Instrument Method:	**Grad40-60to90-10 MeCN-H2O**	*Bandwidth:*	**2**
Processing Method:	**New Processing Method**	*Dilution Factor:*	**1,0000**
Injection Date/Time:	**07.1.22 10:43**	*Sample Weight:*	**1,0000**

Chromatogram

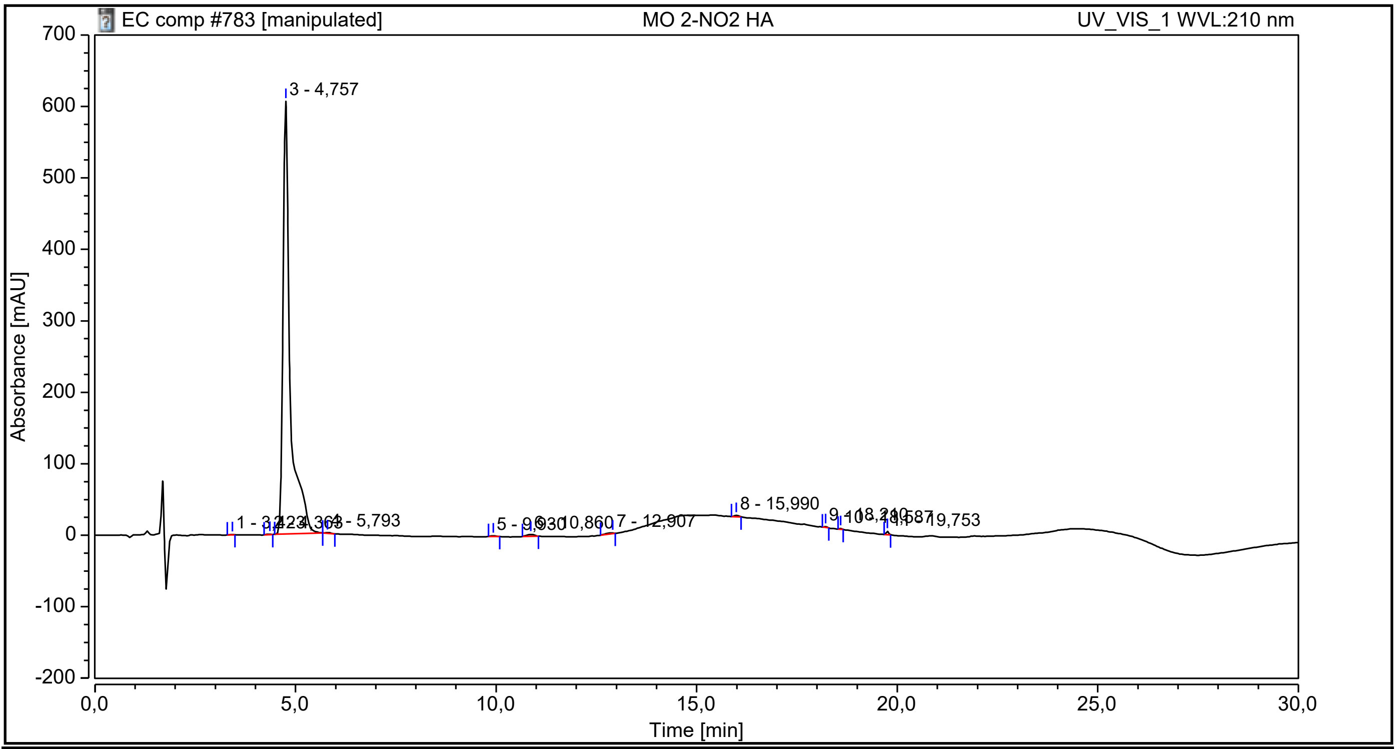

Integration Results

No.	Peak Name	Retention Time<br>min	Area<br>mAU*min	Height<br>mAU	Relative Area<br>%	Relative Height<br>%	Amount<br>n.a.
1	n.a.	3,423	0,043	0,429	0,033	0,07	n.a.
2	n.a.	4,363	0,078	0,653	0,060	0,11	n.a.
3	n.a.	4,757	126,981	605,566	98,428	97,48	n.a.
4	n.a.	5,793	0,165	1,079	0,128	0,17	n.a.
5	n.a.	9,930	0,159	1,068	0,123	0,17	n.a.
6	n.a.	10,860	0,544	2,644	0,421	0,43	n.a.
7	n.a.	12,907	0,298	0,860	0,231	0,14	n.a.
8	n.a.	15,990	0,229	1,954	0,178	0,31	n.a.
9	n.a.	18,210	0,097	1,436	0,075	0,23	n.a.
10	n.a.	18,587	0,063	0,898	0,049	0,14	n.a.
11	n.a.	19,753	0,352	4,645	0,273	0,75	n.a.
**Total:**			**129,009**	**621,232**	**100,00**	**100,00**	

(2*E*)-*N*-Hydroxy-3-(4-{[(3-nitrophenyl)carbamoyl]methoxy}phenyl)prop-2-enamide (**7u**)

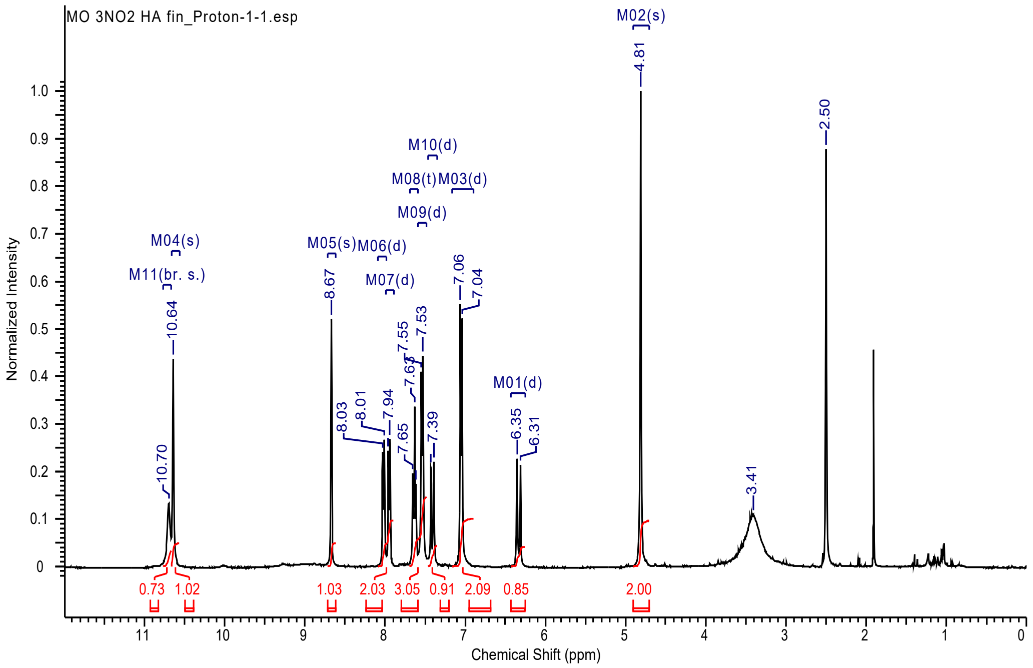

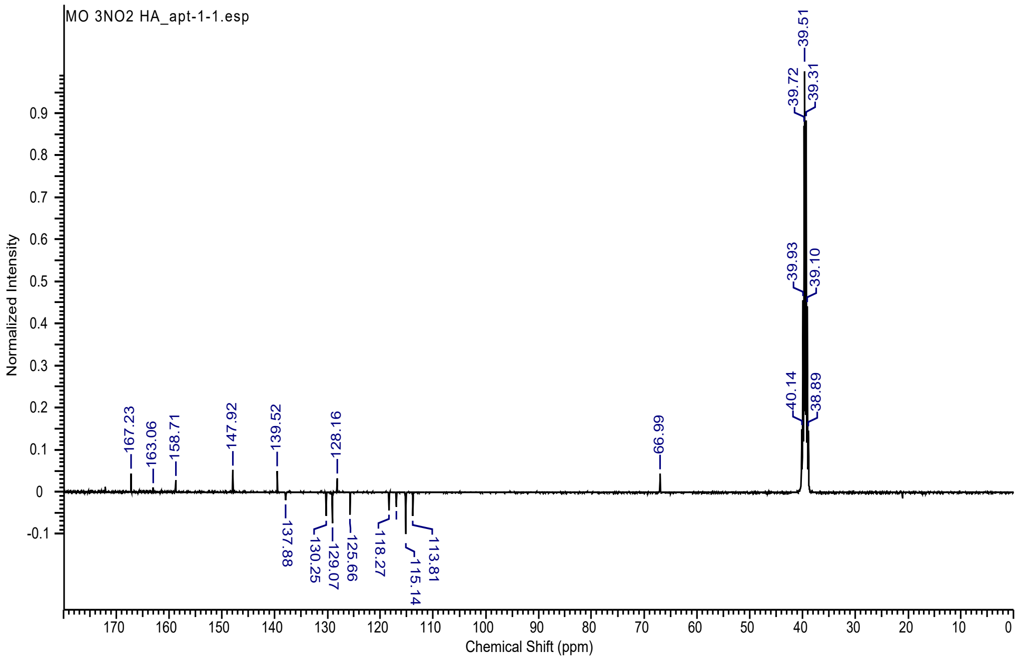

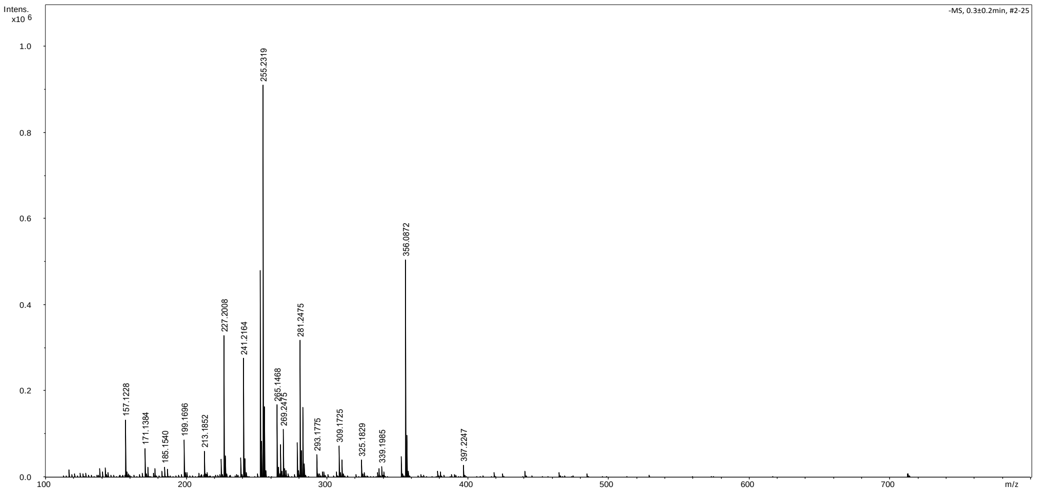

Chromatogram and Results

Injection Details

Injection Name:	**MO 3-NO2 HA**	*Run Time (min):*	**30,00**
Vial Number:	**BC8**	*Injection Volume:*	**5,00**
Injection Type:	**Unknown**	*Channel:*	**UV_VIS_1**
Calibration Level:		*Wavelength:*	**210,0**
Instrument Method:	**Grad40-60to90-10 MeCN-H2O**	*Bandwidth:*	**2**
Processing Method:	**New Processing Method**	*Dilution Factor:*	**1,0000**
Injection Date/Time:	**07.1.22 11:46**	*Sample Weight:*	**1,0000**

Chromatogram

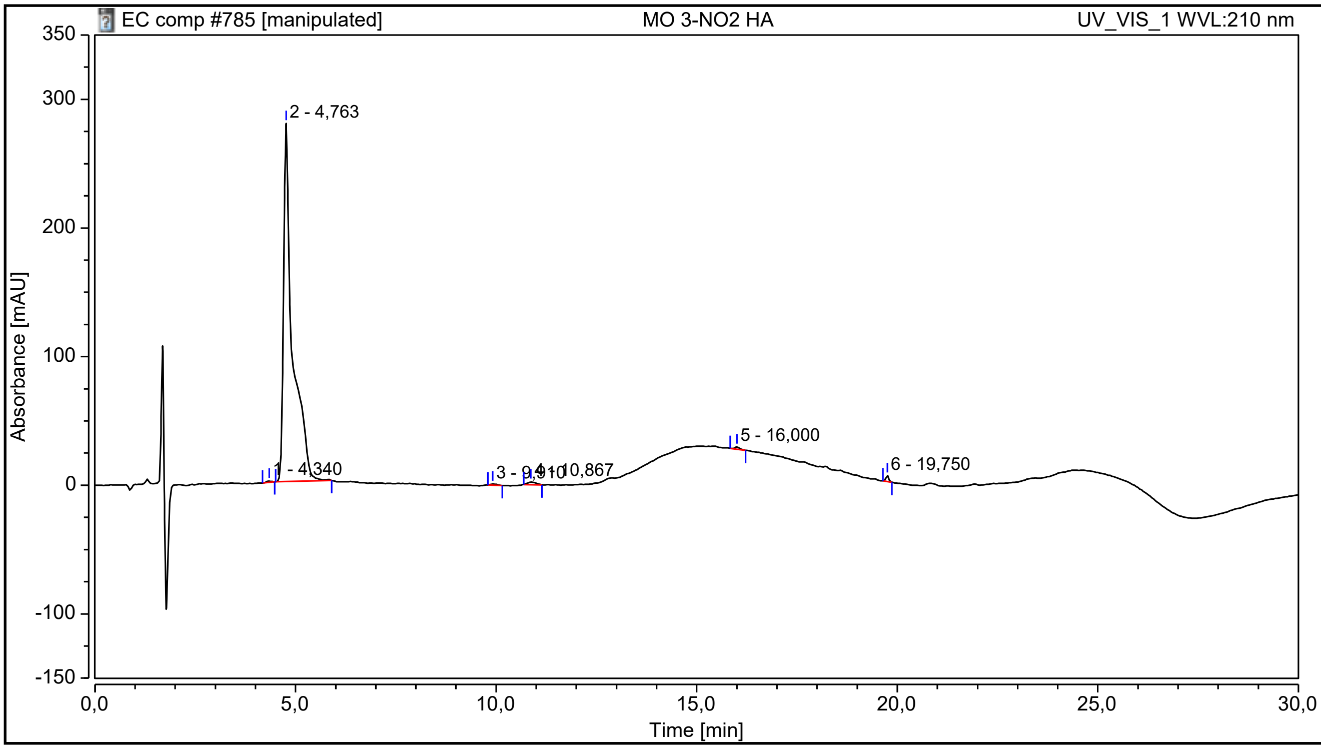

Integration Results

No.	Peak Name	Retention Time<br>min	Area<br>mAU*min	Height<br>mAU	Relative Area<br>%	Relative Height<br>%	Amount<br>n.a.
1	n.a.	4,340	0,131	0,992	0,178	0,34	n.a.
2	n.a.	4,763	71,919	278,363	98,069	96,34	n.a.
3	n.a.	9,910	0,164	0,808	0,224	0,28	n.a.
4	n.a.	10,867	0,485	2,151	0,662	0,74	n.a.
5	n.a.	16,000	0,258	1,872	0,351	0,65	n.a.
6	n.a.	19,750	0,378	4,748	0,516	1,64	n.a.
**Total:**			**73,335**	**288,935**	**100,00**	**100,00**	

(2*E*)-*N*-Hydroxy-3-(4-{[(4-nitrophenyl)carbamoyl]methoxy}phenyl)prop-2-enamide (**7v**)

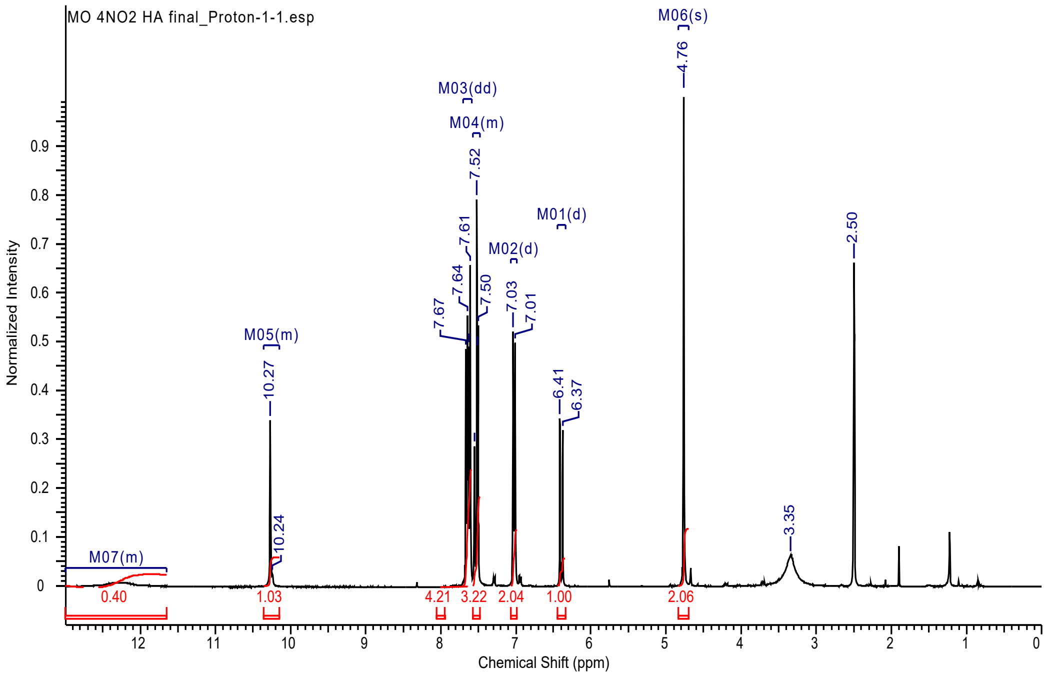

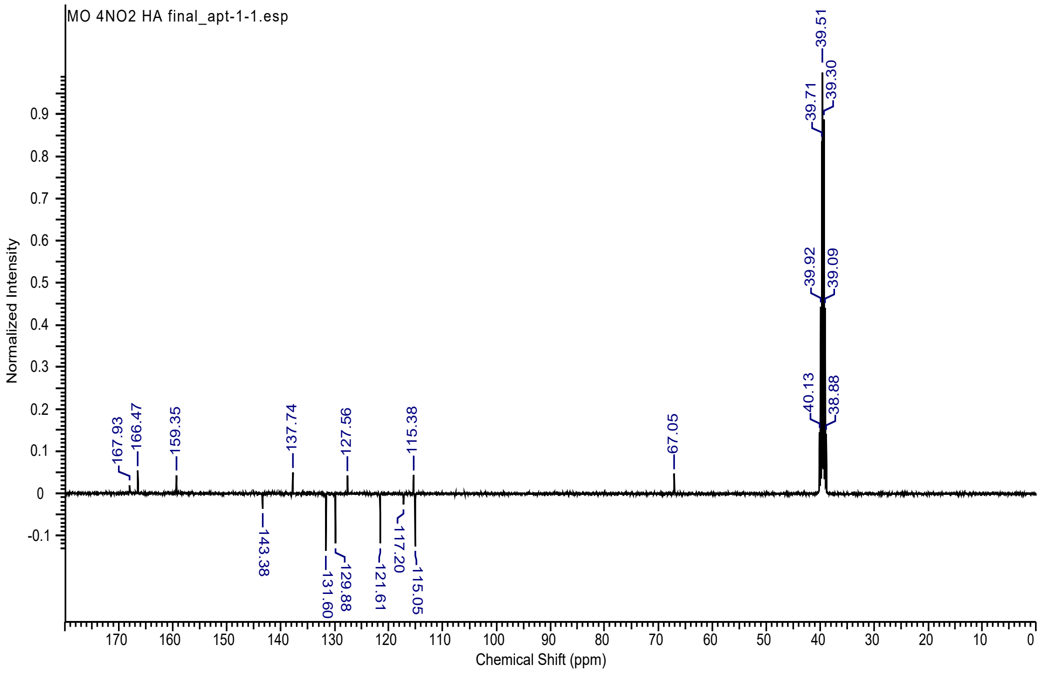

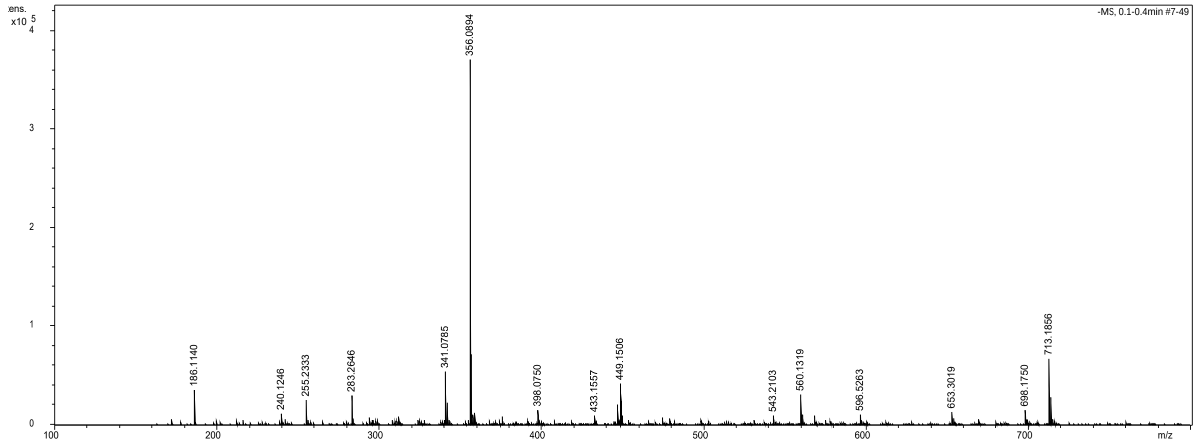

Chromatogram and Results

Injection Details

Injection Name:	**MO 4-NO2 HA**	*Run Time (min):*	**30,00**
Vial Number:	**BD1**	*Injection Volume:*	**5,00**
Injection Type:	**Unknown**	*Channel:*	**UV_VIS_1**
Calibration Level:		*Wavelength:*	**210,0**
Instrument Method:	**Grad40-60to90-10 MeCN-H2O**	*Bandwidth:*	**2**
Processing Method:	**New Processing Method**	*Dilution Factor:*	**1,0000**
Injection Date/Time:	**07.1.22 12:17**	*Sample Weight:*	**1,0000**

Chromatogram

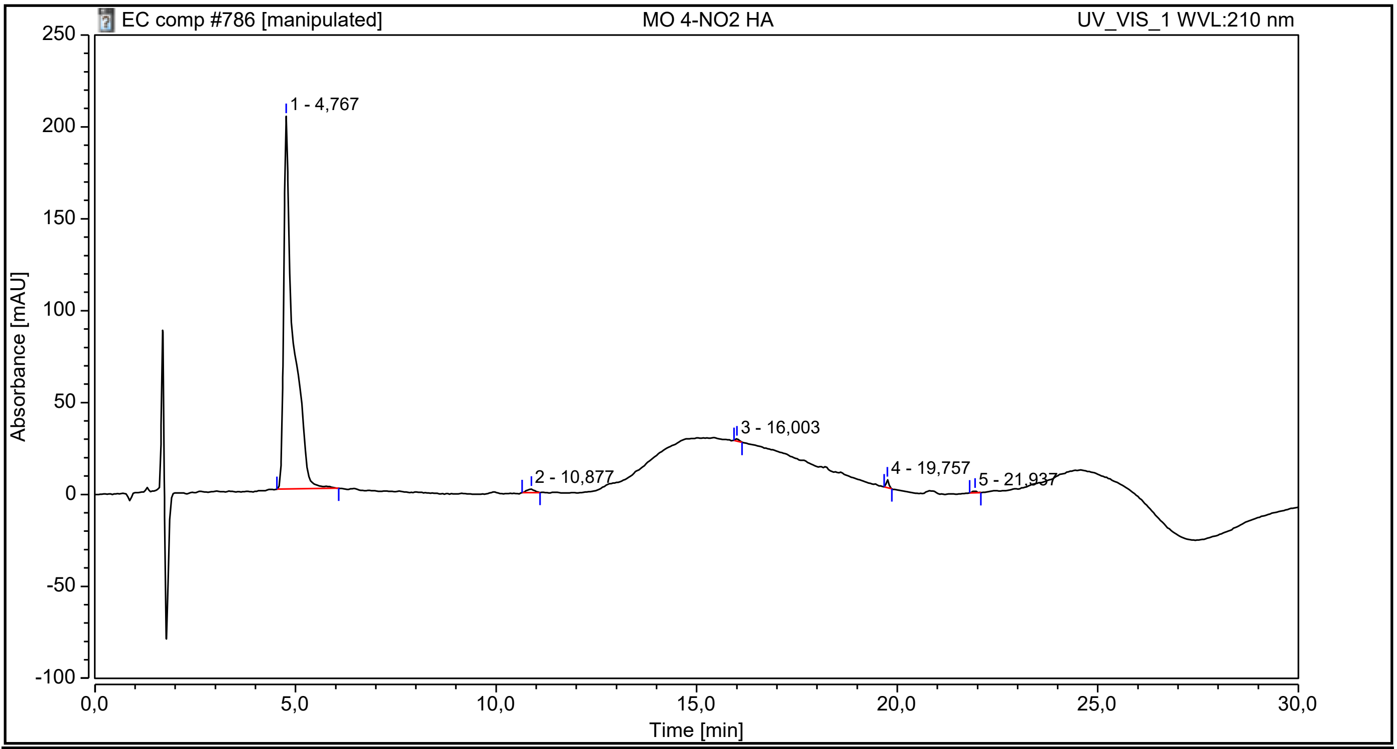

Integration Results

No.	Peak Name	Retention Time min	Area mAU*min	Height mAU	Relative Area %	Relative Height %	Amount n.a.
1	n.a.	4,767	55,878	202,891	98,199	96,02	n.a.
2	n.a.	10,877	0,413	1,887	0,725	0,89	n.a.
3	n.a.	16,003	0,147	1,265	0,259	0,60	n.a.
4	n.a.	19,757	0,337	4,349	0,592	2,06	n.a.
5	n.a.	21,937	0,128	0,917	0,225	0,43	n.a.
**Total:**			**56,902**	**211,309**	**100,00**	**100,00**	

Methyl (2*E*)-3-{4-[(phenylcarbamoyl)methoxy]phenyl}prop-2-enoate (**10a**)

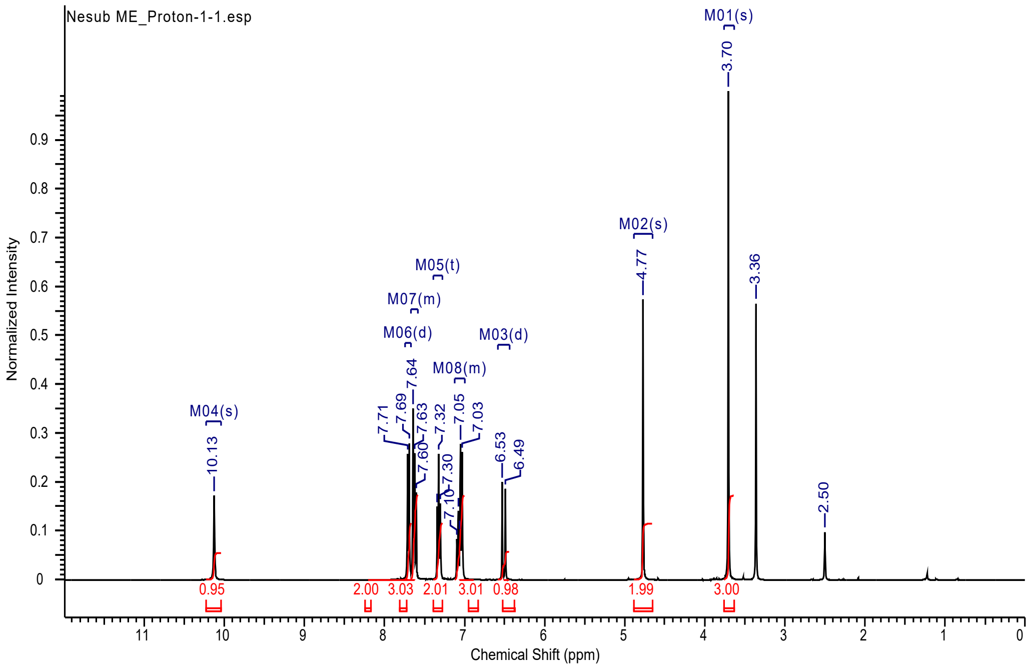

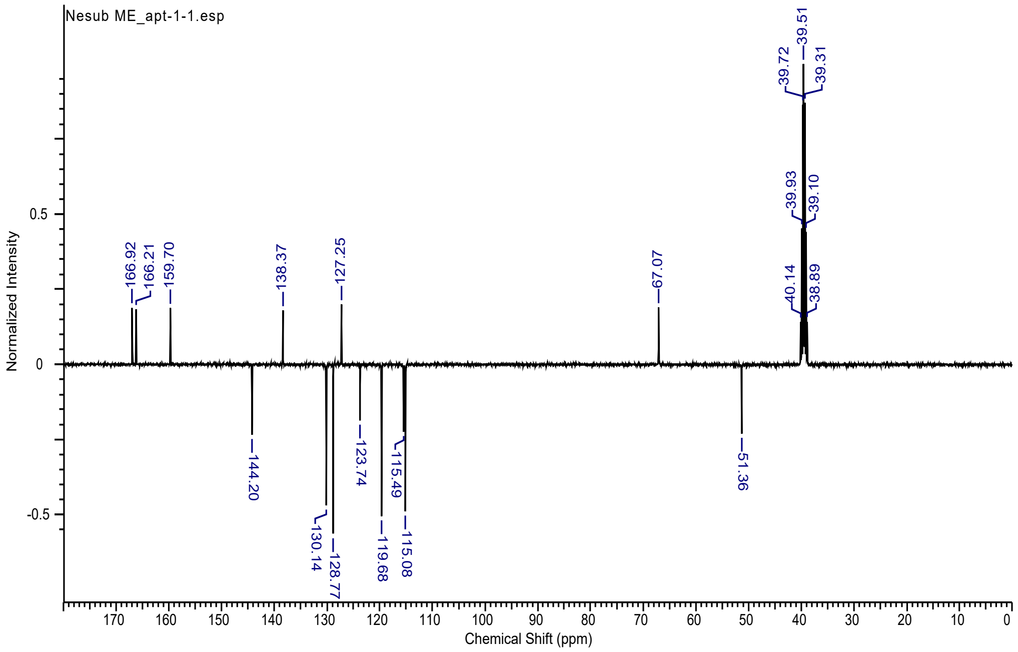

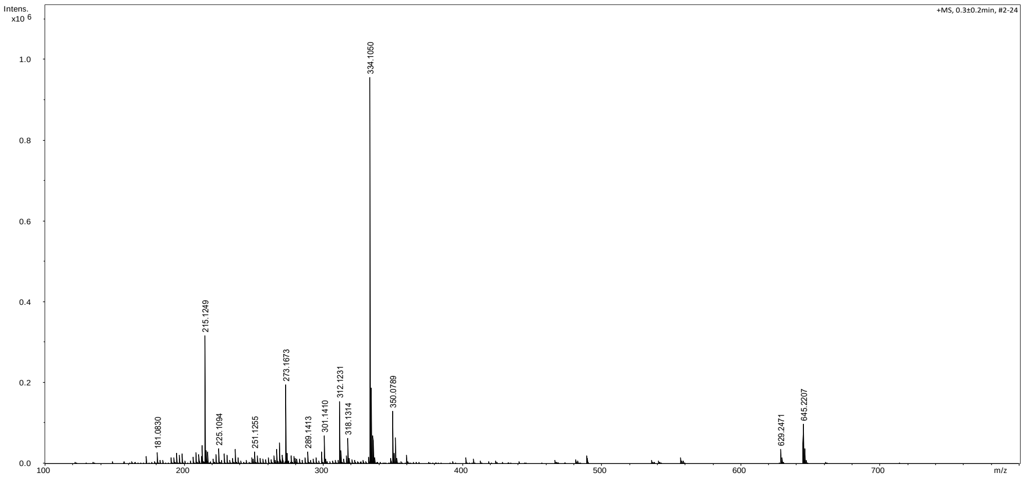

Methyl (2*E*)-3-(4-{[(2-methylphenyl)carbamoyl]methoxy}phenyl)prop-2-enoate (**10b**)

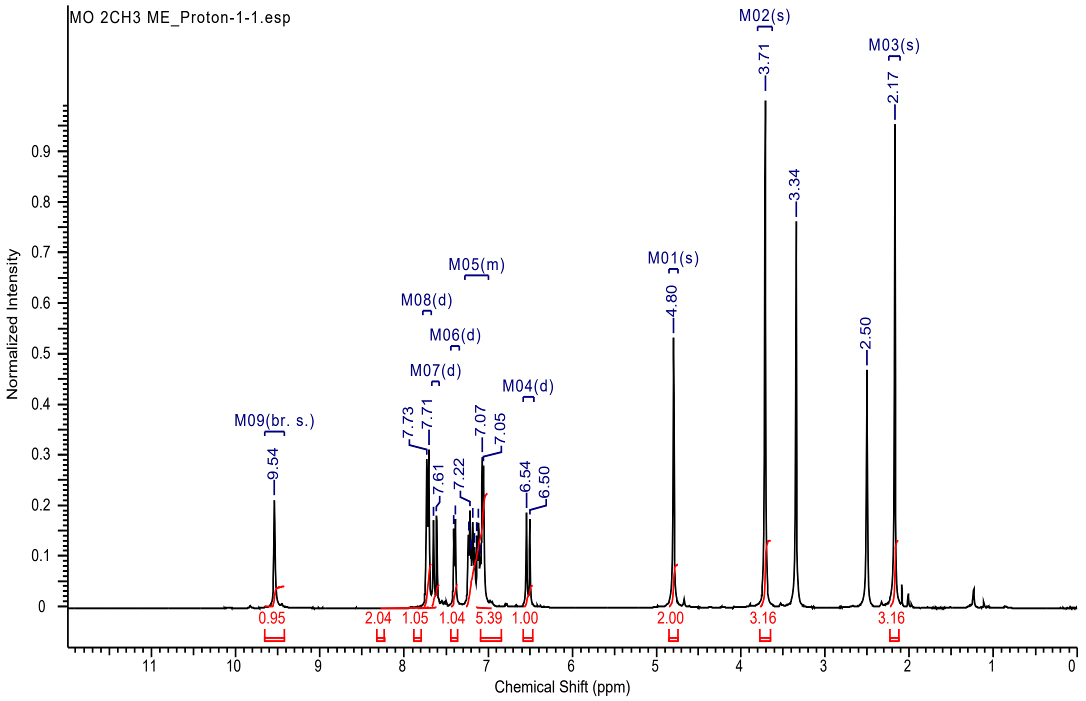

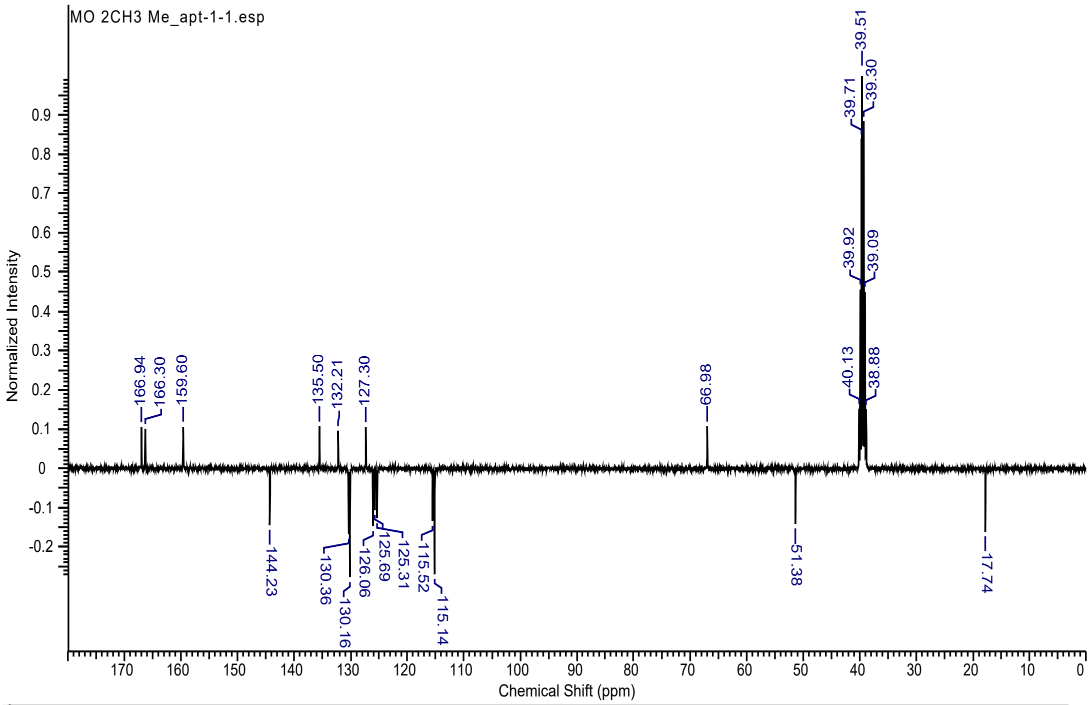

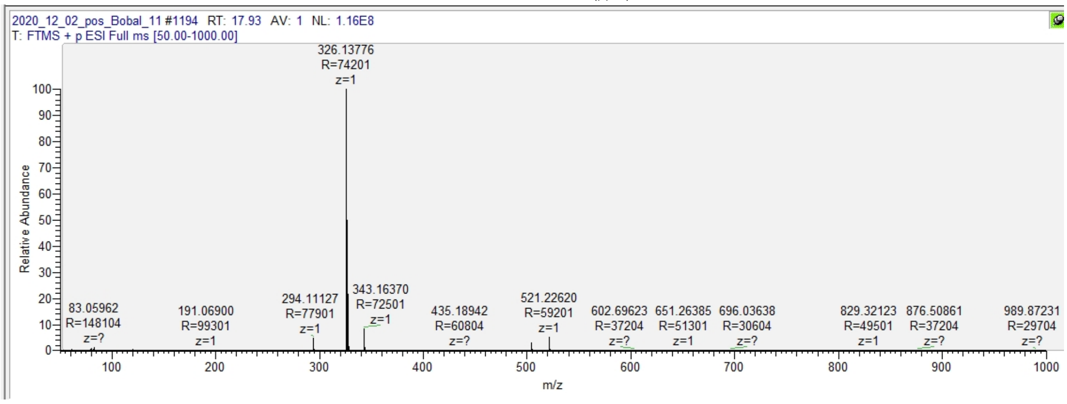

Chromatogram and Results

Injection Details

Injection Name:	**MO 2-CH3 ME**	*Run Time (min):*	**30,00**
Vial Number:	**GE3**	*Injection Volume:*	**10,00**
Injection Type:	**Unknown**	*Channel:*	**UV_VIS_1**
Calibration Level:		*Wavelength:*	**210,0**
Instrument Method:	**Grad40-60to70-30 MeCN-H2O**	*Bandwidth:*	**2**
Processing Method:	**New Processing Method**	*Dilution Factor:*	**1,0000**
Injection Date/Time:	**10.12.20 05:12**	*Sample Weight:*	**1,0000**

Chromatogram

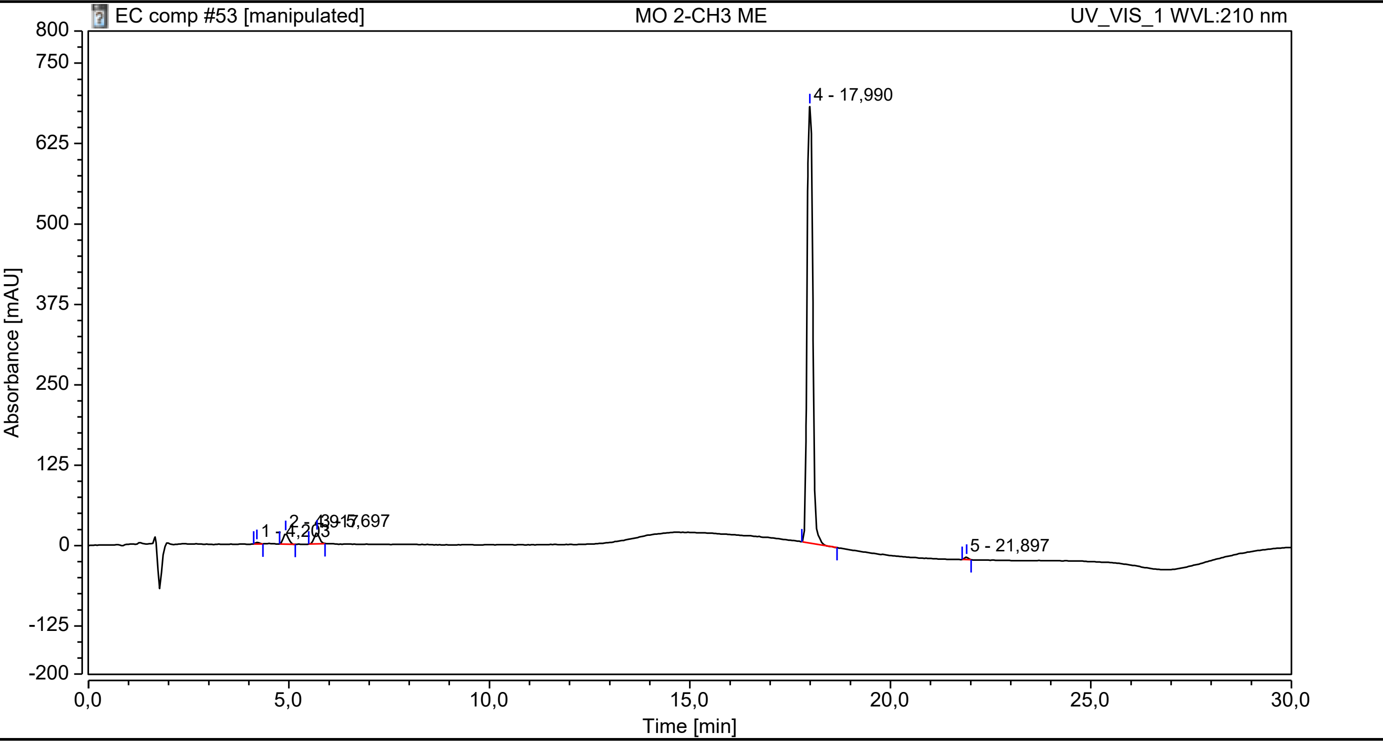

Integration Results

No.	Peak Name	Retention Time min	Area mAU*min	Height mAU	Relative Area %	Relative Height %	Amount n.a.
1	n.a.	4,203	0,302	2,491	0,258	0,35	n.a.
2	n.a.	4,917	2,530	16,824	2,158	2,34	n.a.
3	n.a.	5,697	2,623	17,409	2,237	2,42	n.a.
4	n.a.	17,990	111,395	678,866	95,011	94,36	n.a.
5	n.a.	21,897	0,394	3,826	0,336	0,53	n.a.
**Total:**			**117,245**	**719,416**	**100,00**	**100,00**	

Methyl (2*E*)-3-(4-{[(3-methylphenyl)carbamoyl]methoxy}phenyl)prop-2-enoate (**10c**)

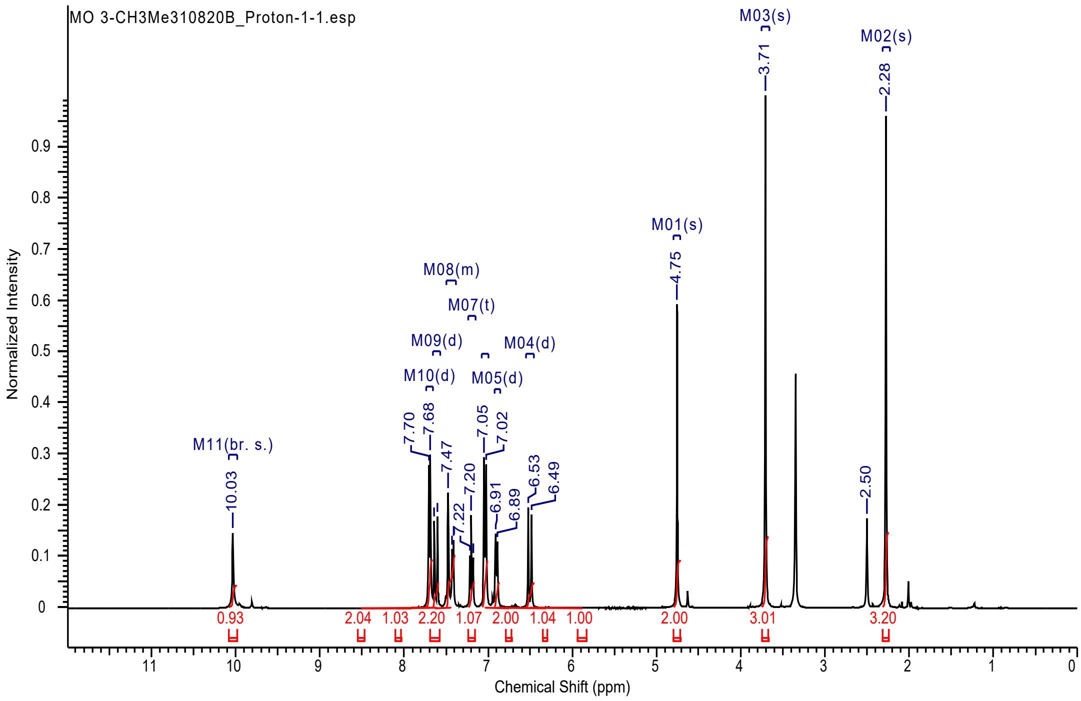

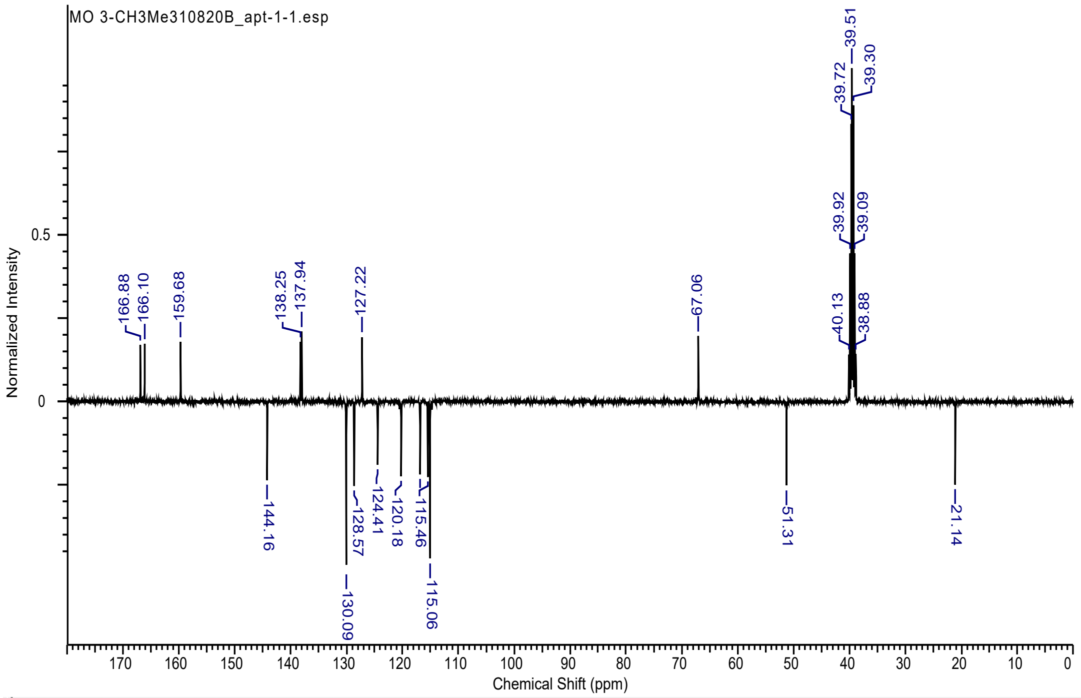

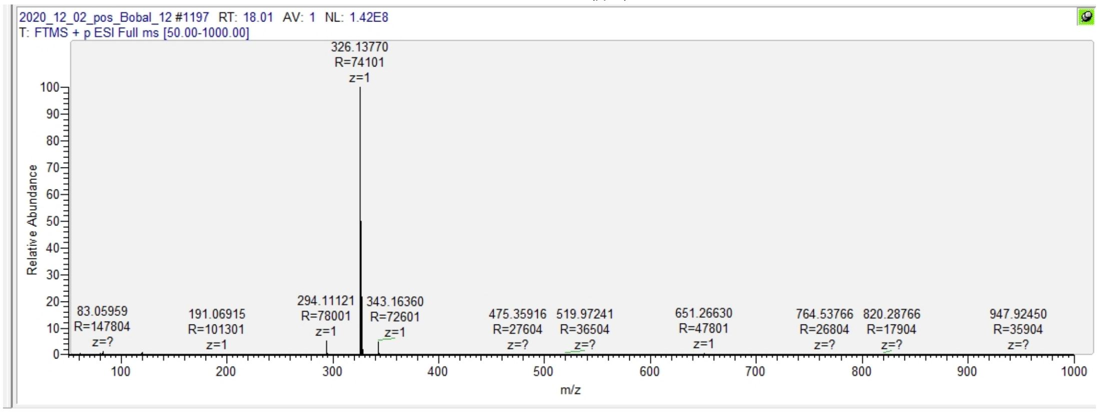

Methyl (2*E*)-3-(4-{[(4-methylphenyl)carbamoyl]methoxy}phenyl)prop-2-enoate (**10d**)

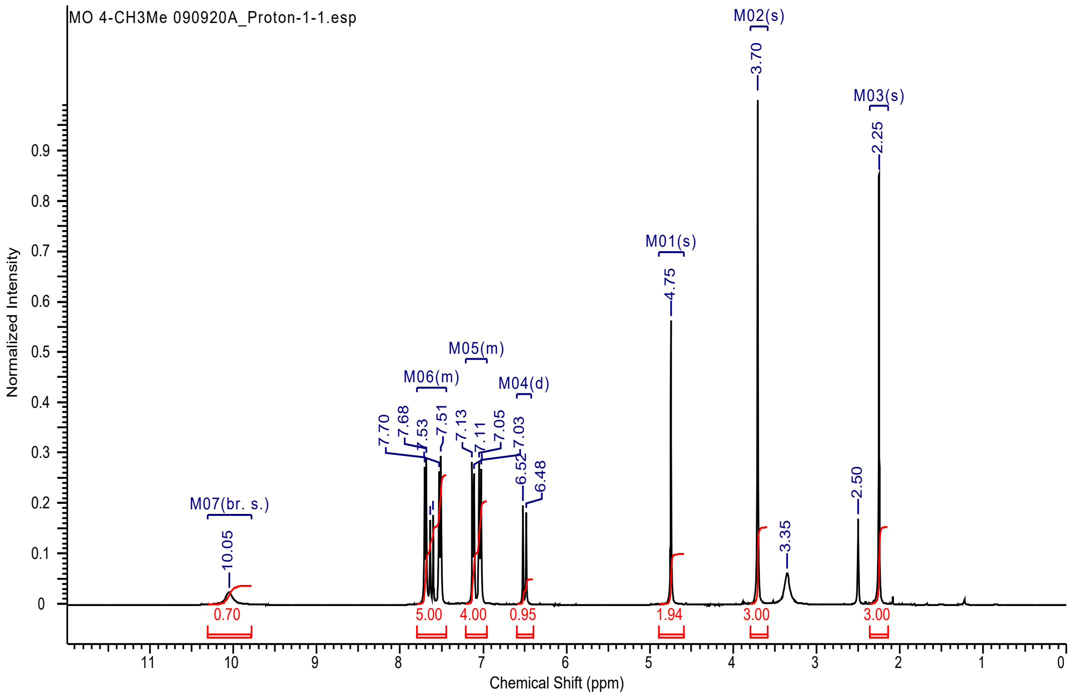

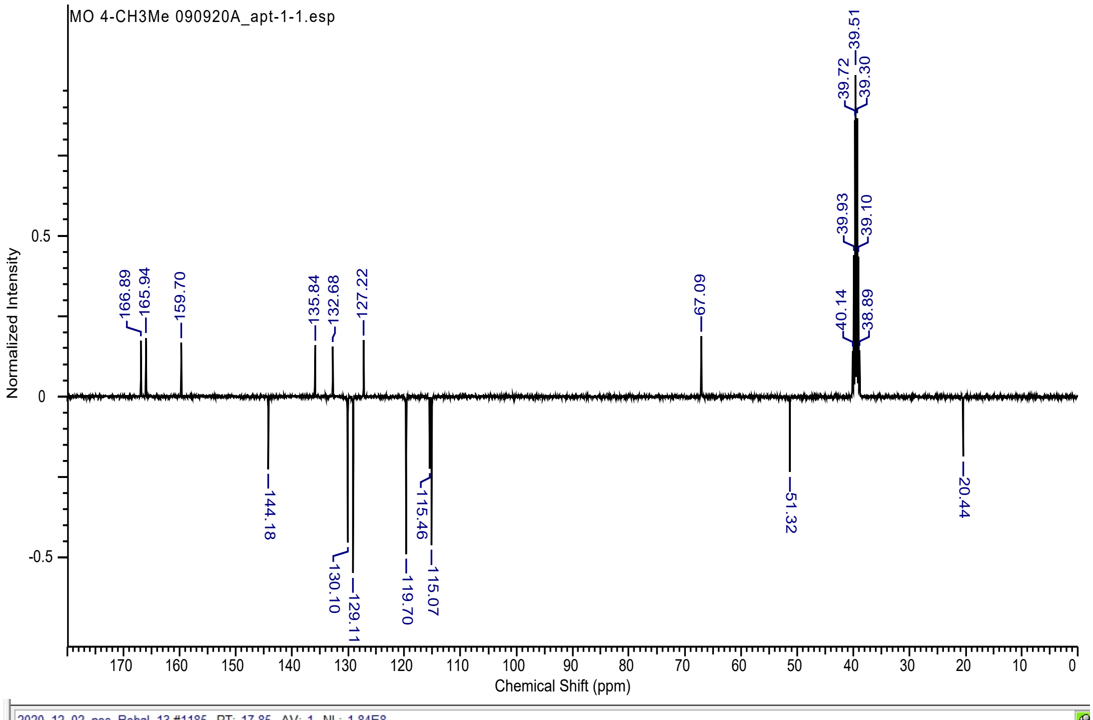

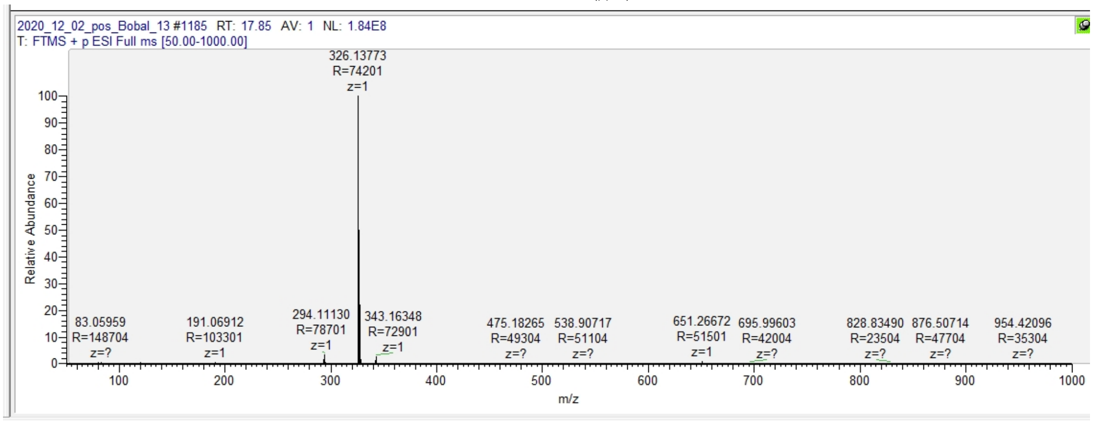

Methyl (2*E*)-3-(4-{[(2-methoxyphenyl)carbamoyl]methoxy}phenyl)prop-2-enoate (**10e**)

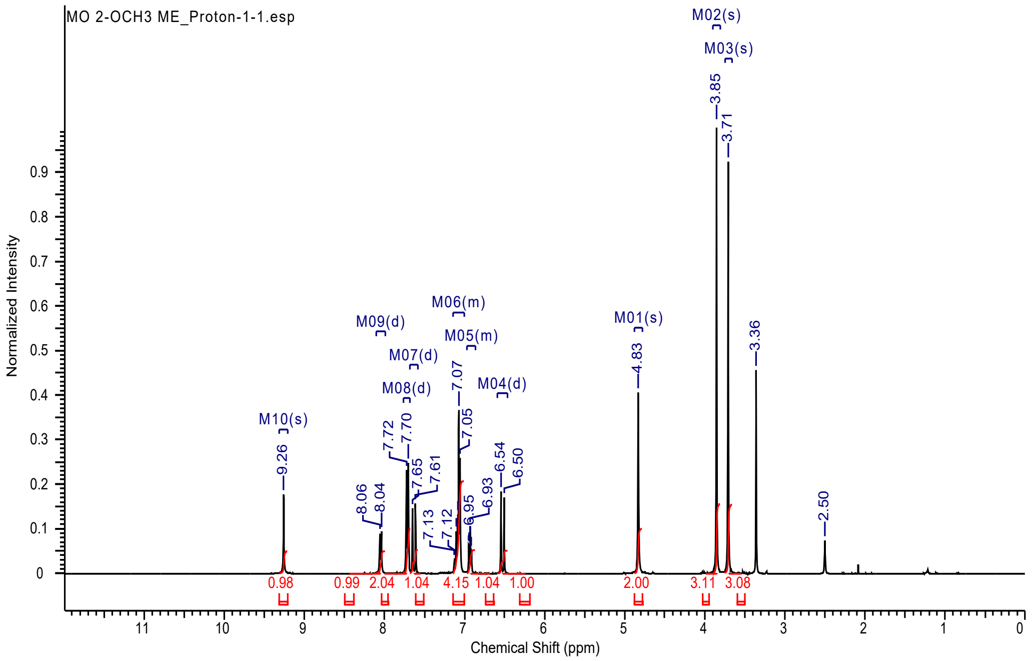

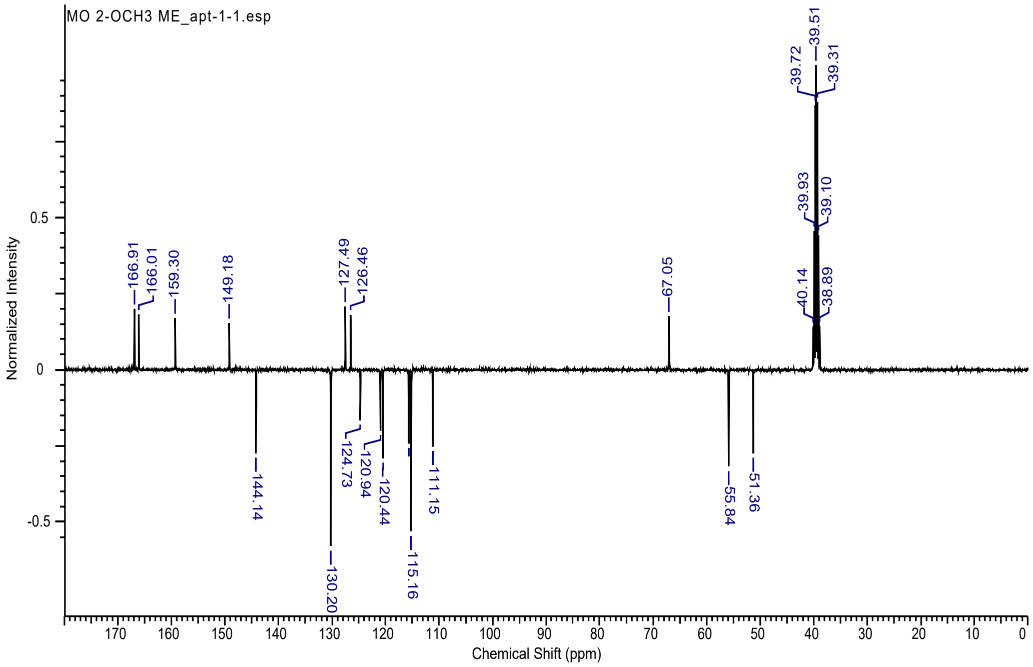

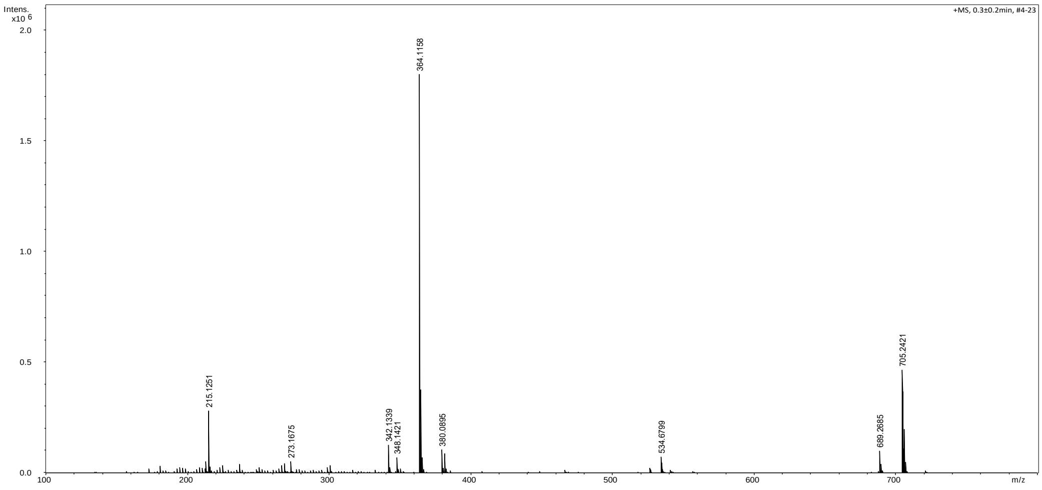

Chromatogram and Results

Injection Details

Injection Name:	**MO 2- OMe ME**	*Run Time (min):*	**30,00**
Vial Number:	**RA5**	*Injection Volume:*	**1,00**
Injection Type:	**Unknown**	*Channel:*	**UV_VIS_1**
Calibration Level:		*Wavelength:*	**210,0**
Instrument Method:	**Grad40-60to90-10 MeCN-H2O**	*Bandwidth:*	**2**
Processing Method:	**New Processing Method**	*Dilution Factor:*	**1,0000**
Injection Date/Time:	**21.10.22 09:55**	*Sample Weight:*	**1,0000**

Chromatogram

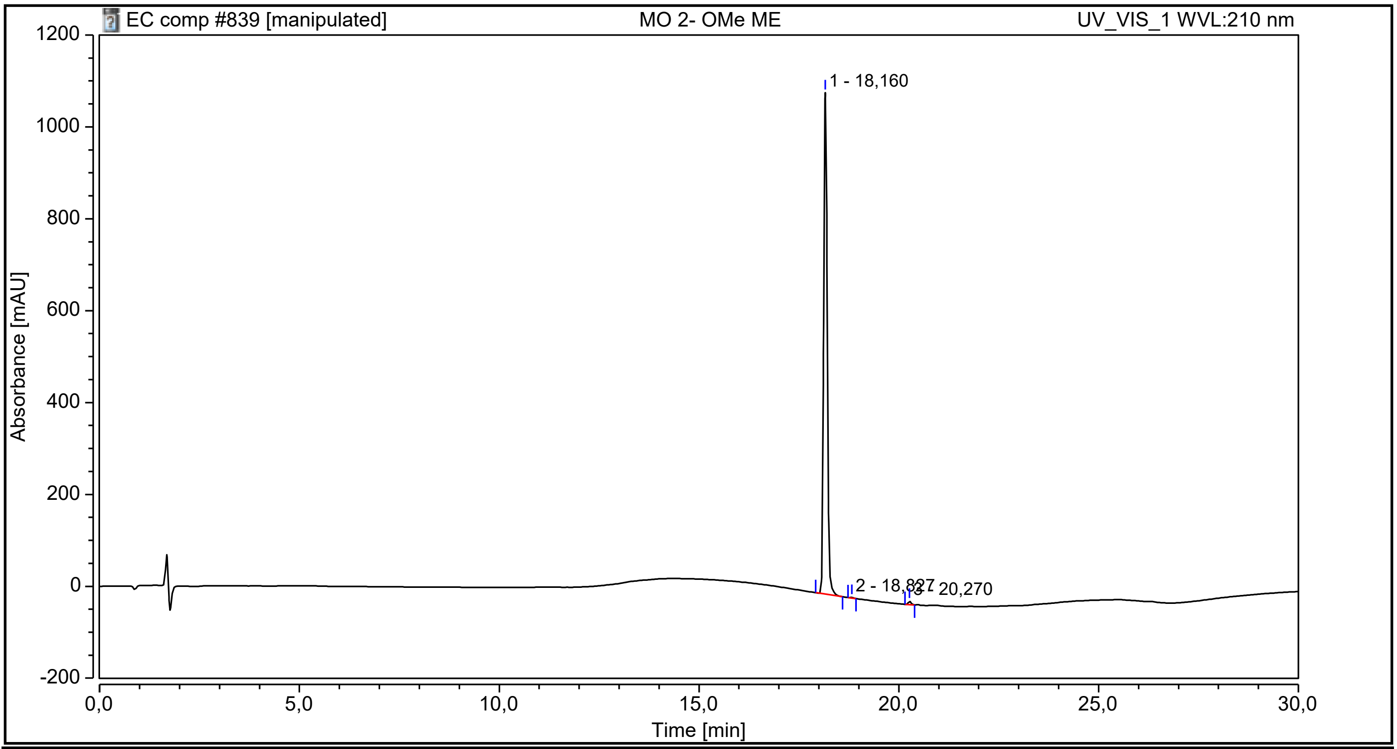

Integration Results

No.	Peak Name	Retention Time min	Area mAU*min	Height mAU	Relative Area %	Relative Height %	Amount n.a.
1	n.a.	18,160	115,517	1091,737	99,357	99,16	n.a.
2	n.a.	18,827	0,137	1,606	0,118	0,15	n.a.
3	n.a.	20,270	0,610	7,622	0,525	0,69	n.a.
**Total:**			**116,264**	**1100,965**	**100,00**	**100,00**	

Methyl (2*E*)-3-(4-{[(3-methoxyphenyl)carbamoyl]methoxy}phenyl)prop-2-enoate (**10f**)

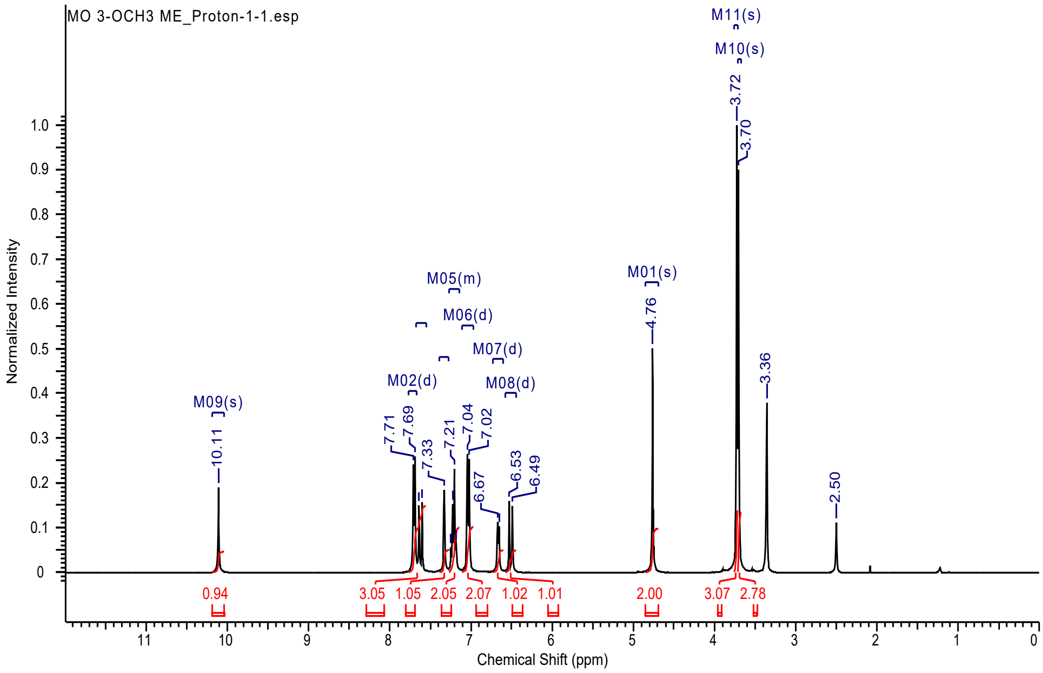

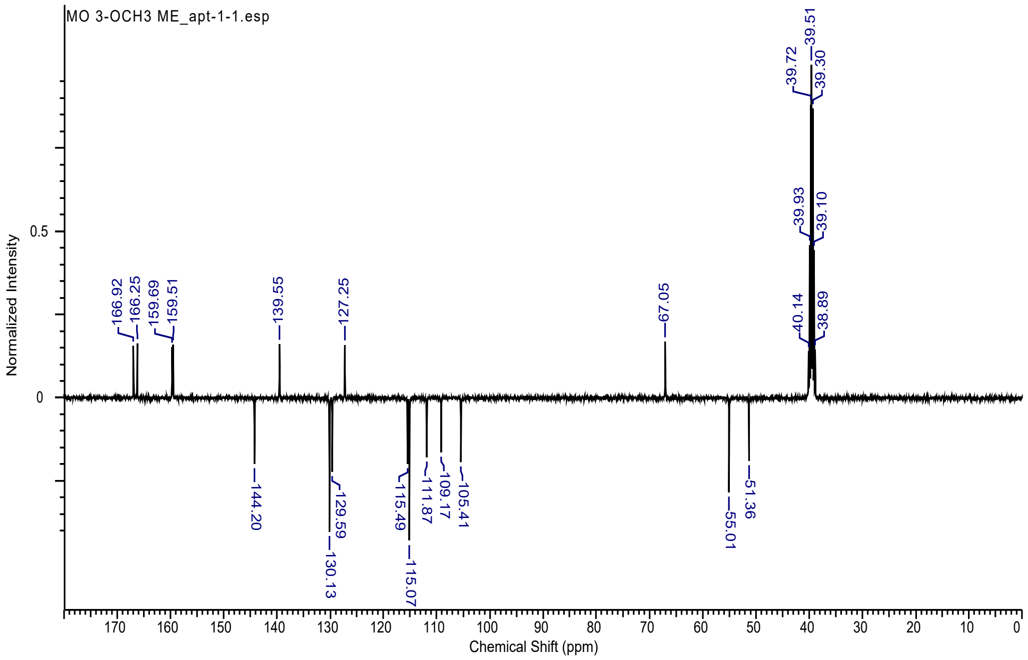

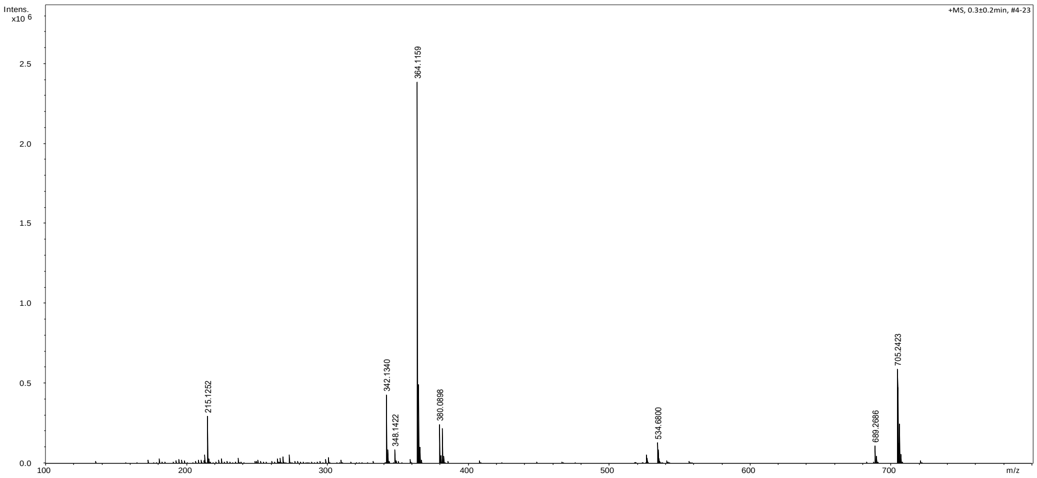

Methyl (2*E*)-3-(4-{[(4-methoxyphenyl)carbamoyl]methoxy}phenyl)prop-2-enoate (**10g**)

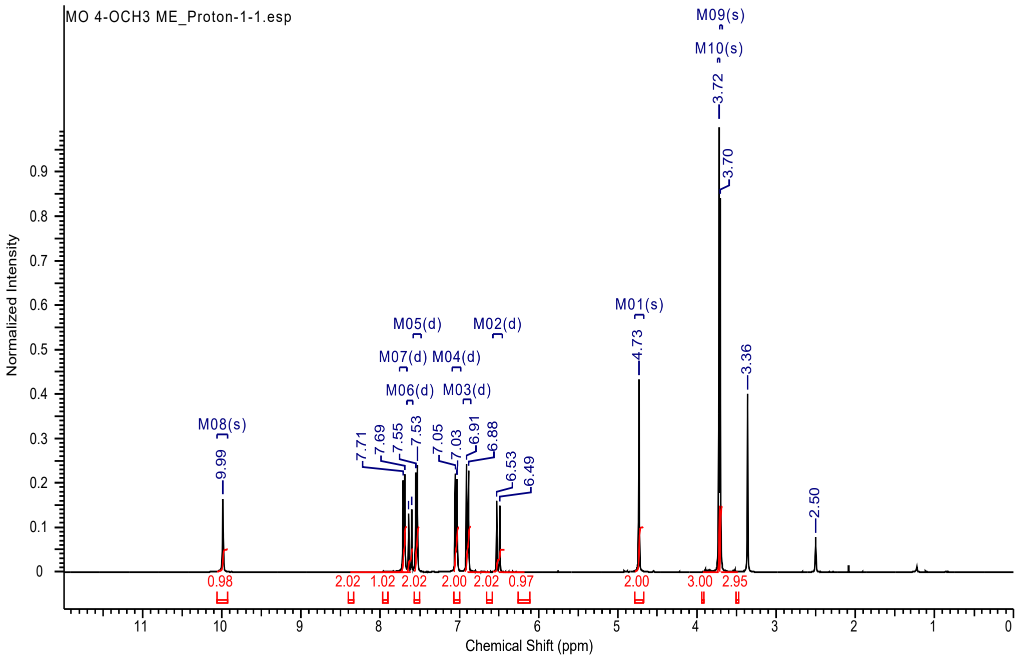

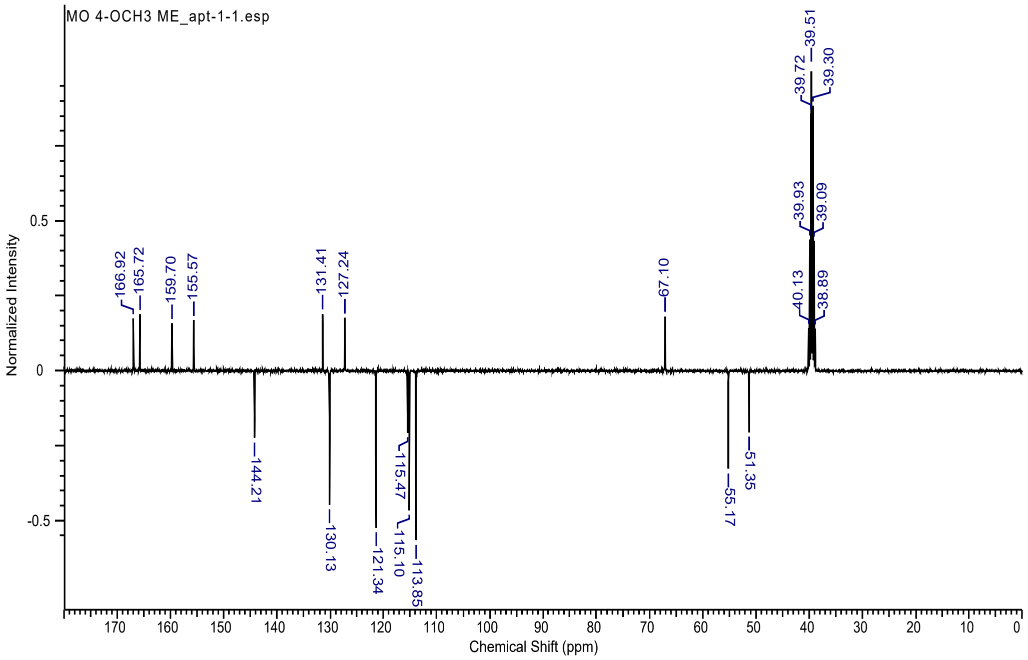

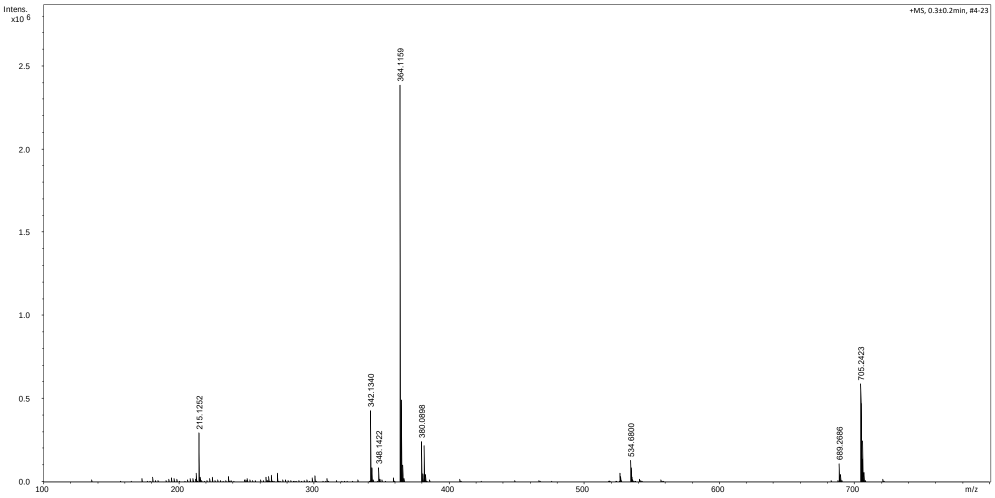

Methyl (2*E*)-3-(4-{[(2-fluorophenyl)carbamoyl]methoxy}phenyl)prop-2-enoate (**10h**)

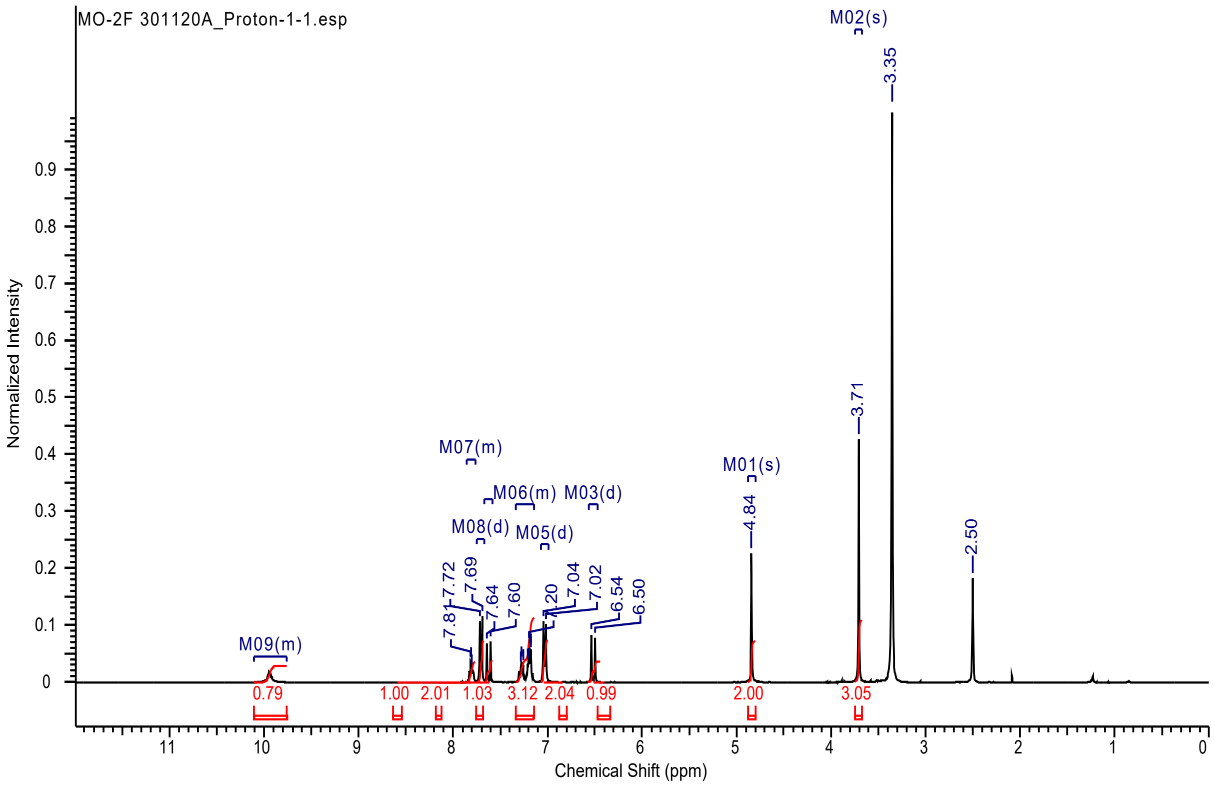

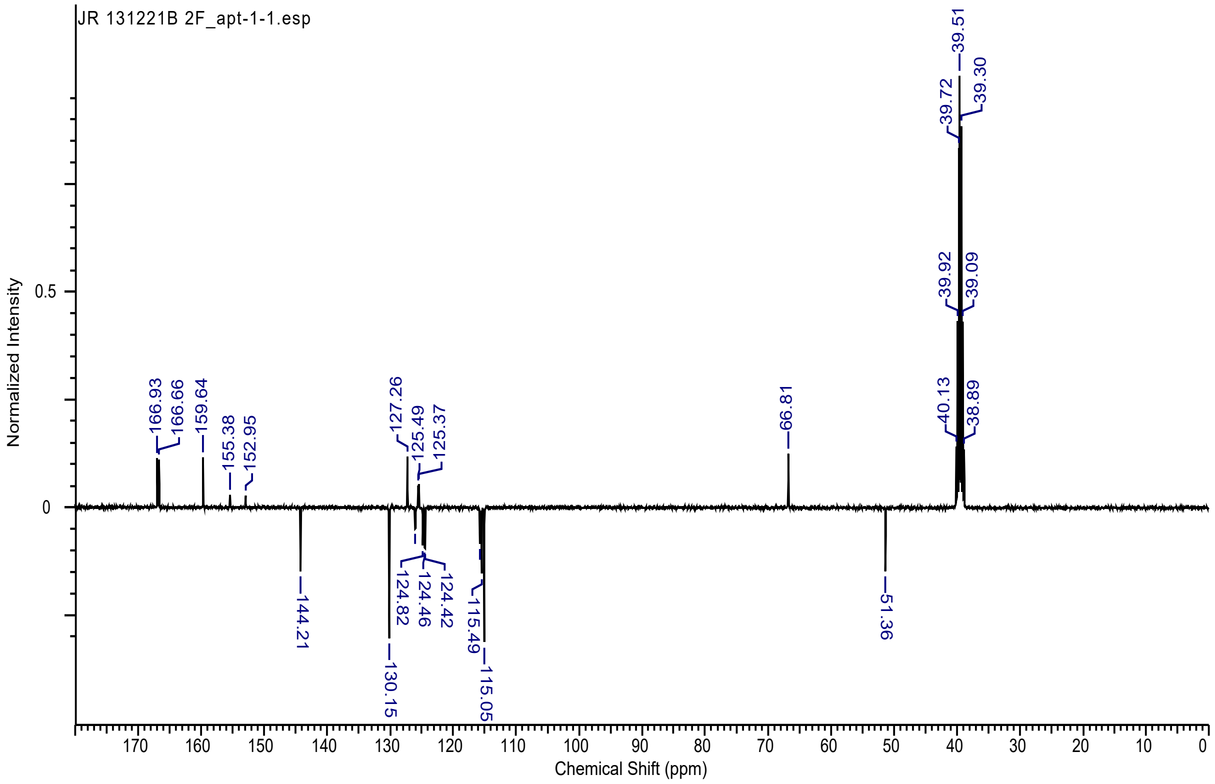

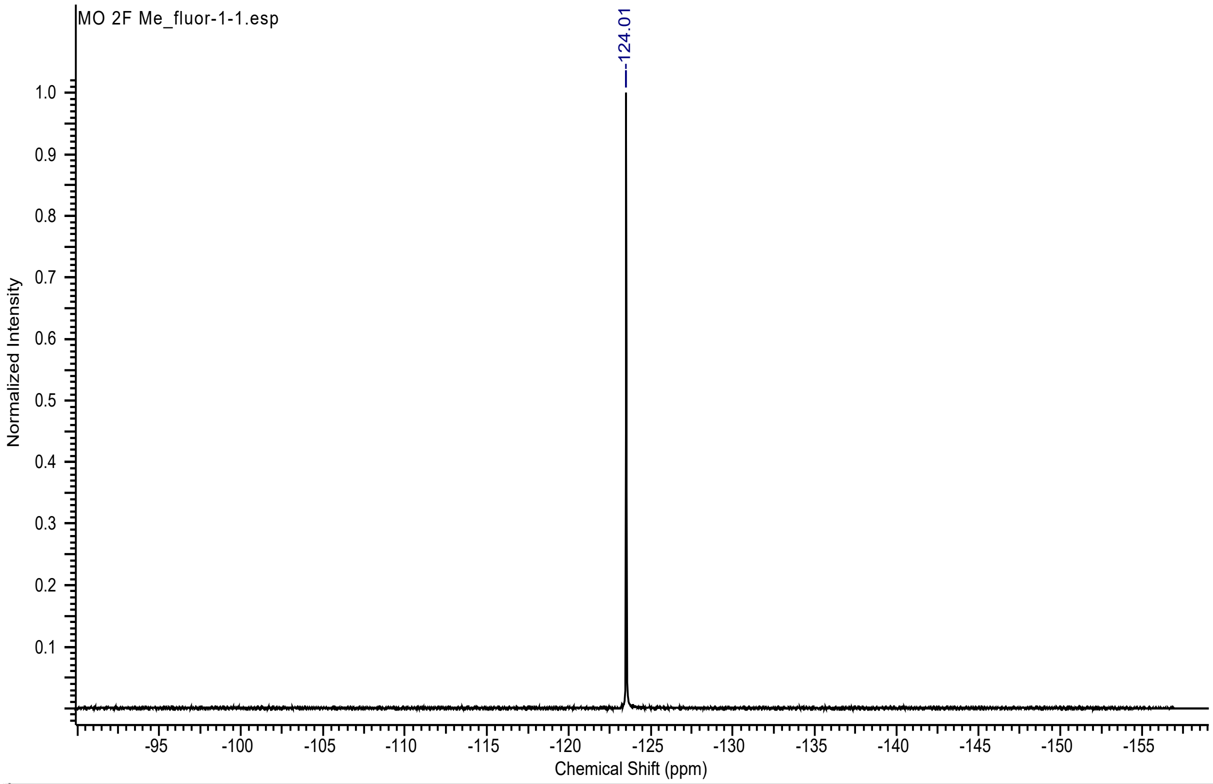

MO 2F Me_fluor-1-1.esp
-124.01
Normalized Intensity
Chemical Shift (ppm)

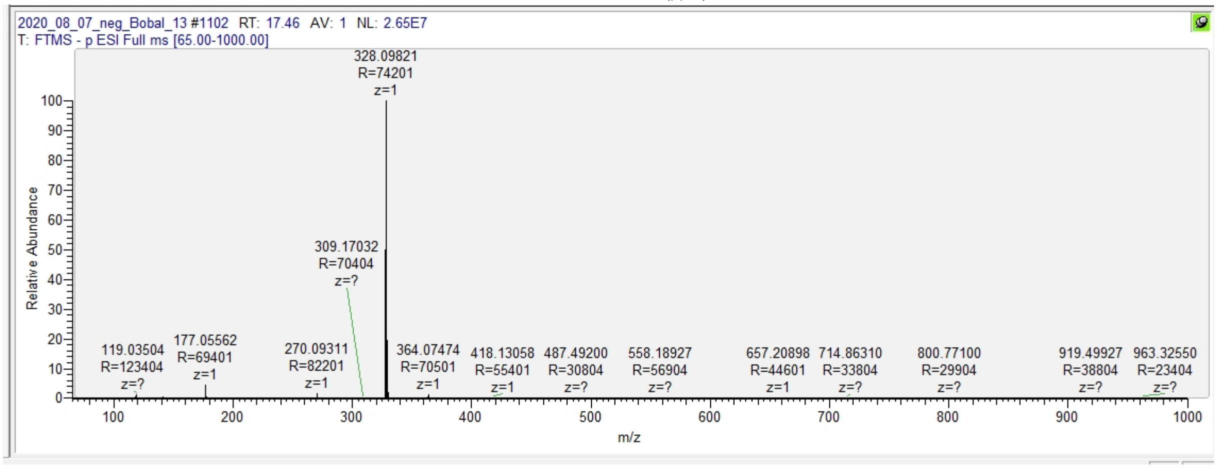

2020_08_07_neg_Bobal_13 #1102 RT: 17.46 AV: 1 NL: 2.65E7
T: FTMS - p ESI Full ms [65.00-1000.00]
328.09821
R=74201
z=1
309.17032
R=70404
z=?
119.03504
R=123404
z=?
177.05562
R=69401
z=1
270.09311
R=82201
z=1
364.07474
R=70501
z=1
418.13058
R=55401
z=1
487.49200
R=30804
z=?
558.18927
R=56904
z=?
657.20898
R=44601
z=1
714.86310
R=33804
z=?
800.77100
R=29904
z=?
919.49927
R=38804
z=?
963.32550
R=23404
z=?
Relative Abundance
m/z

Methyl (2*E*)-3-(4-{[(3-fluorophenyl)carbamoyl]methoxy}phenyl)prop-2-enoate (**10i**)

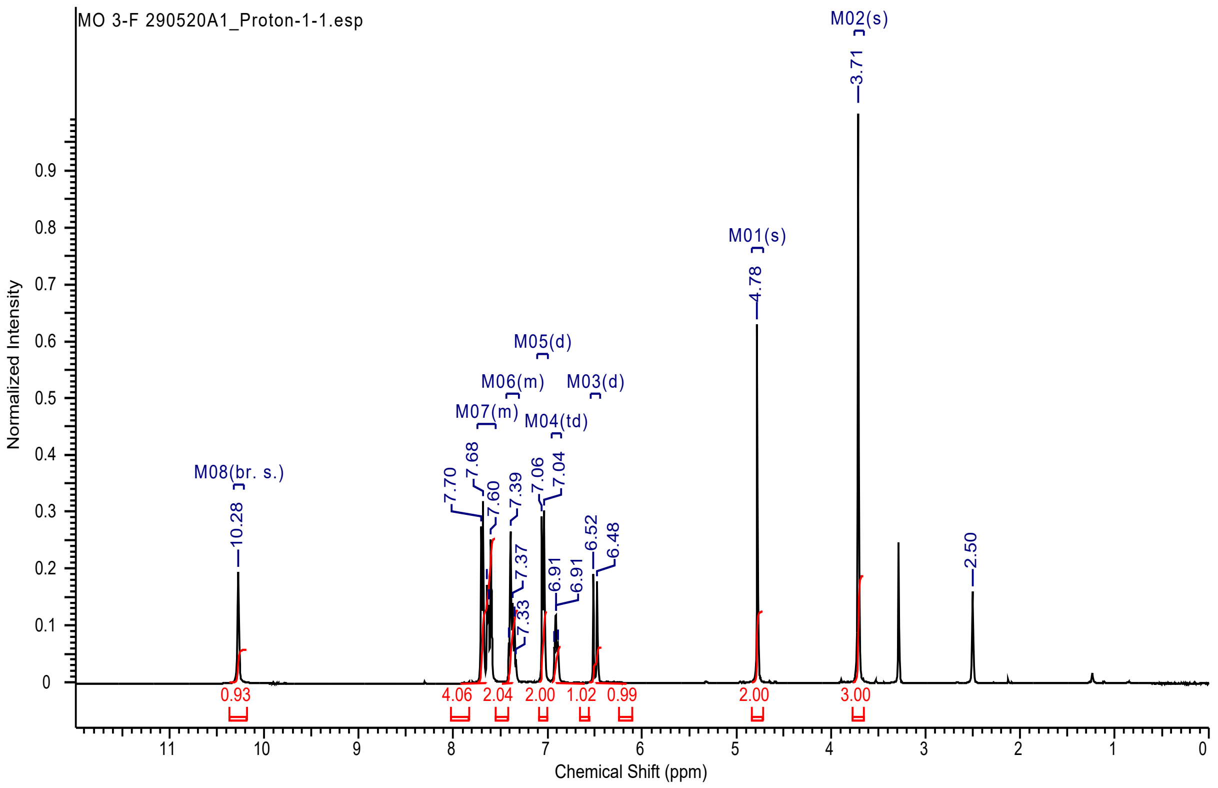

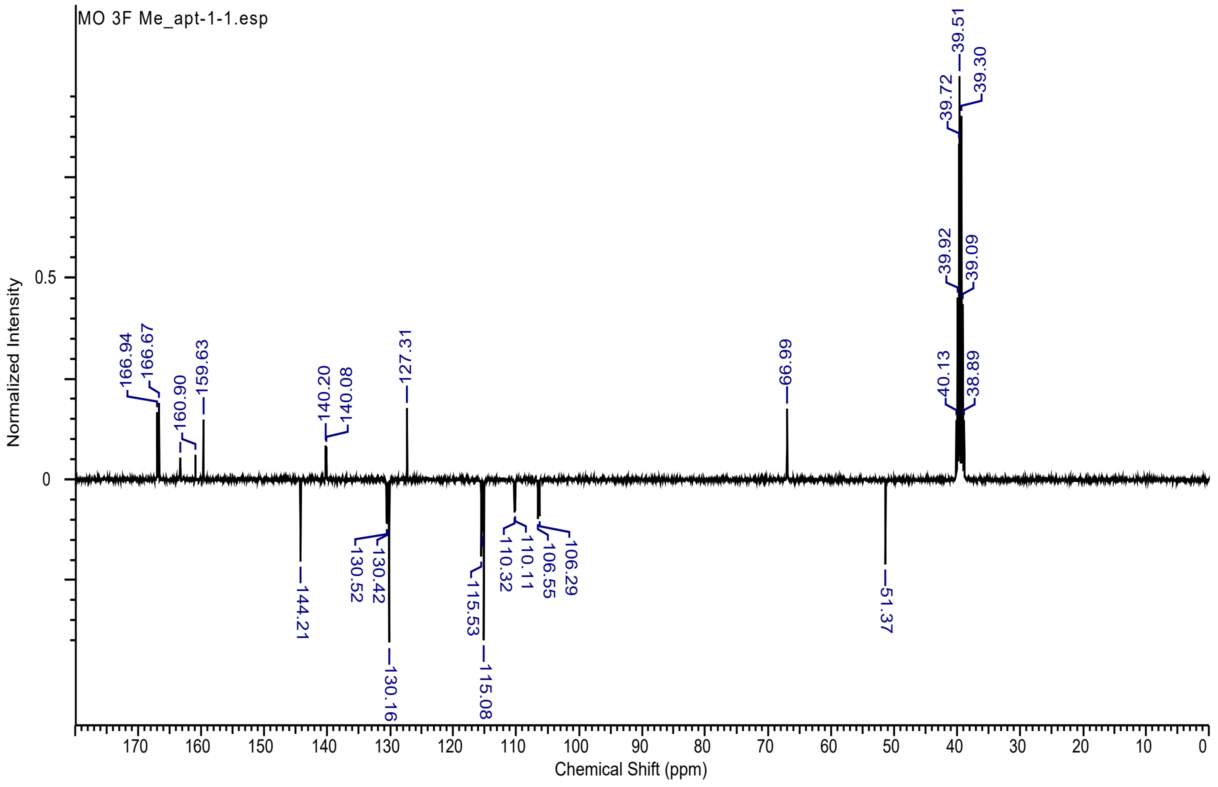

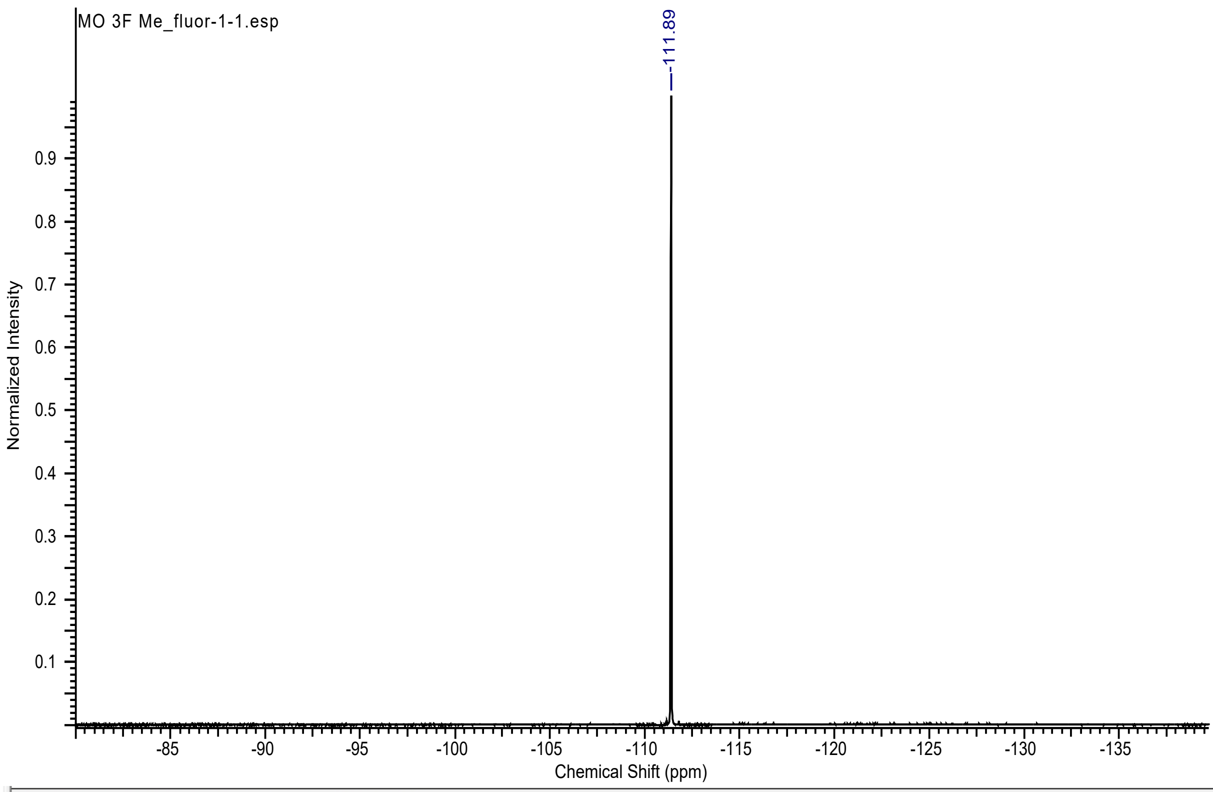
MO 3F Me_fluor-1-1.esp
-111.89
Normalized Intensity
Chemical Shift (ppm)

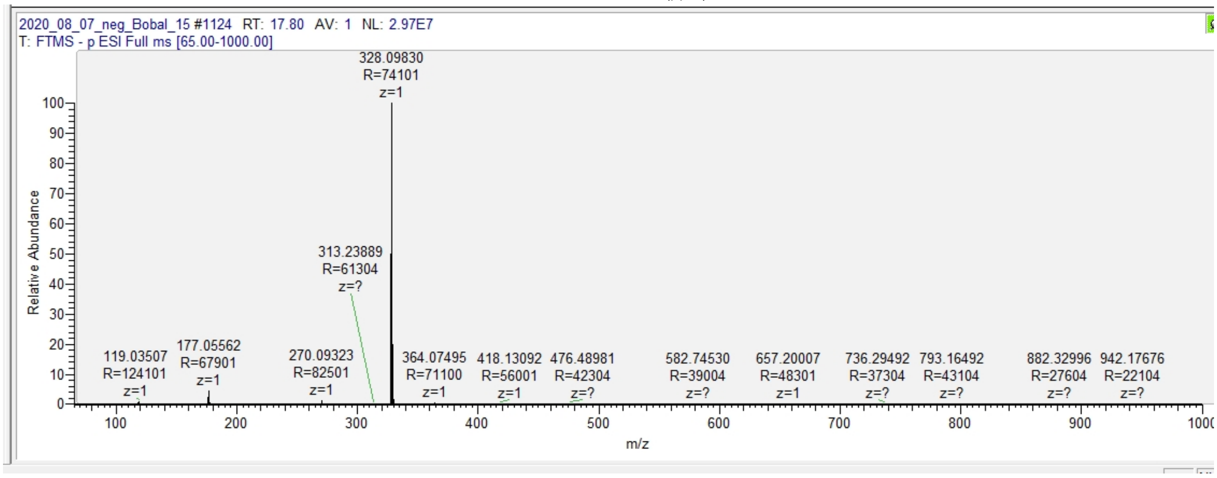
2020_08_07_neg_Bobal_15 #1124 RT: 17.80 AV: 1 NL: 2.97E7
T: FTMS - p ESI Full ms [65.00-1000.00]
328.09830
R=74101
z=1
313.23889
R=61304
z=?
119.03507
R=124101
z=1
177.05562
R=67901
z=1
270.09323
R=82501
z=1
364.07495
R=71100
z=1
418.13092
R=56001
z=1
476.48981
R=42304
z=?
582.74530
R=39004
z=?
657.20007
R=48301
z=1
736.29492
R=37304
z=?
793.16492
R=43104
z=?
882.32996
R=27604
z=?
942.17676
R=22104
z=?
Relative Abundance
m/z

Methyl (2*E*)-3-(4-{[(4-fluorophenyl)carbamoyl]methoxy}phenyl)prop-2-enoate (**10j**)

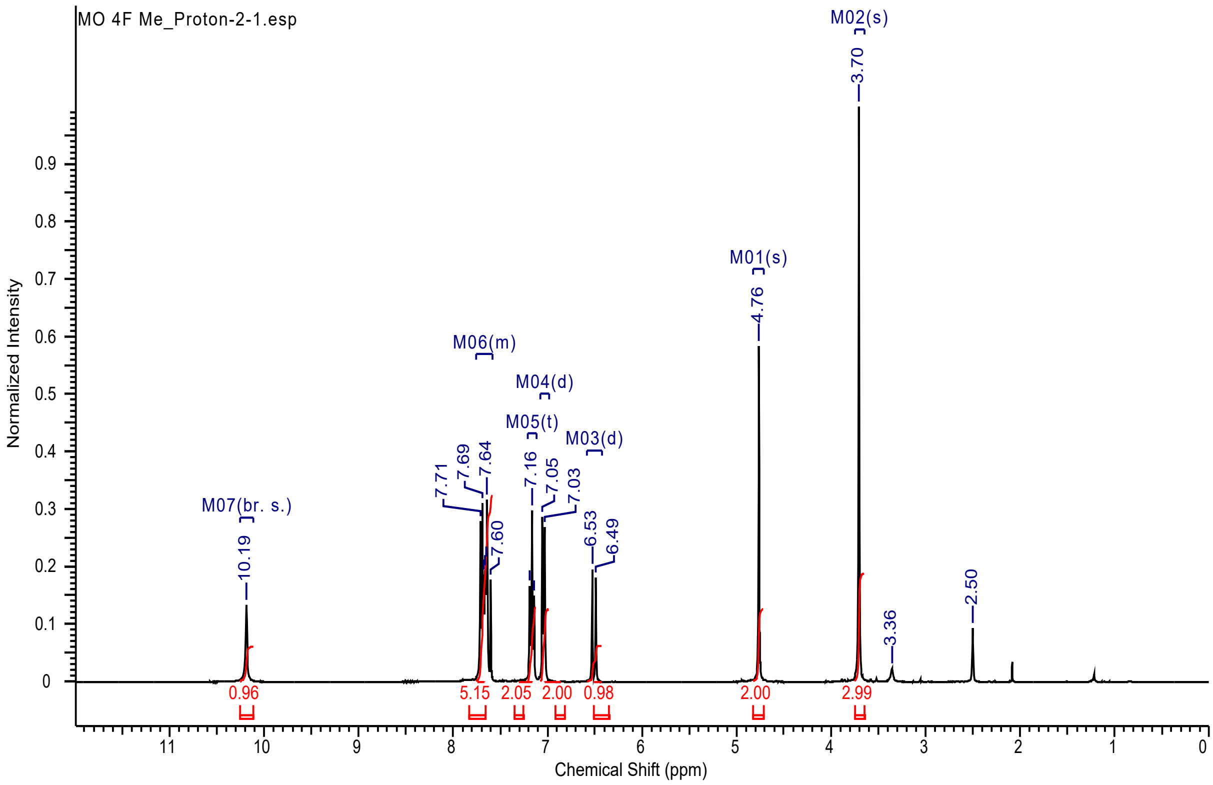

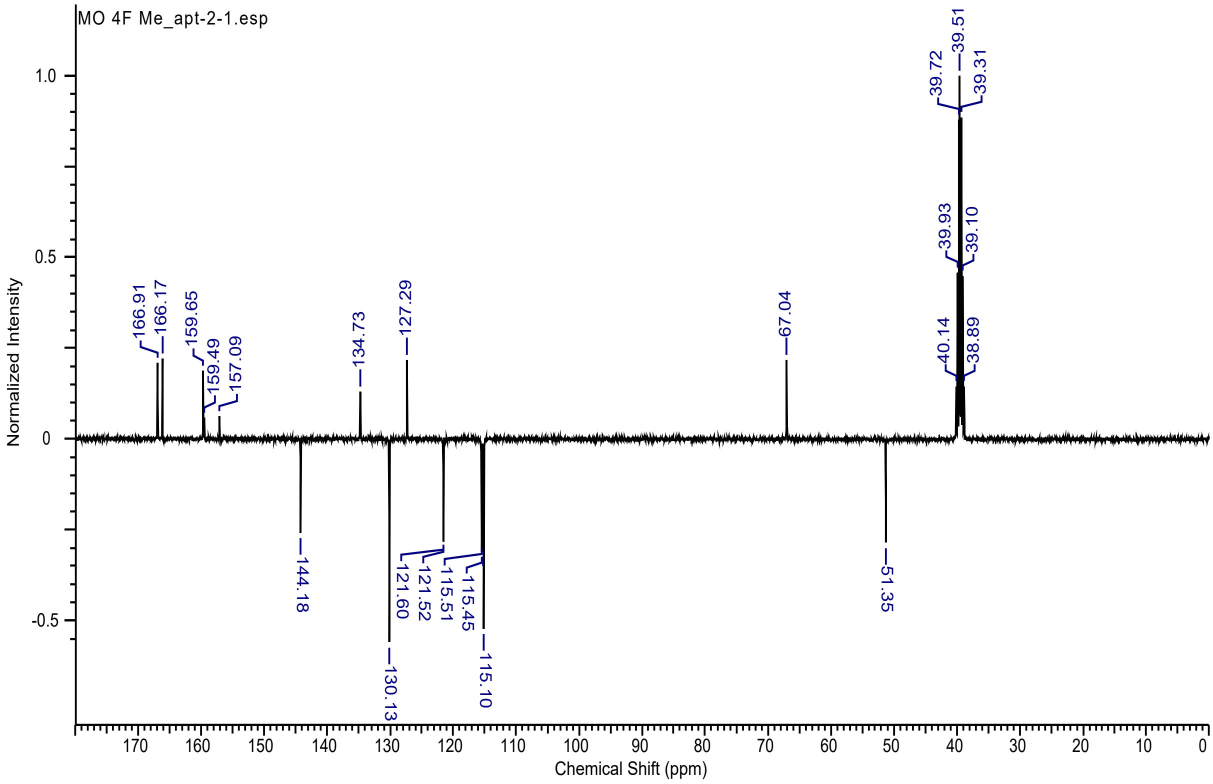

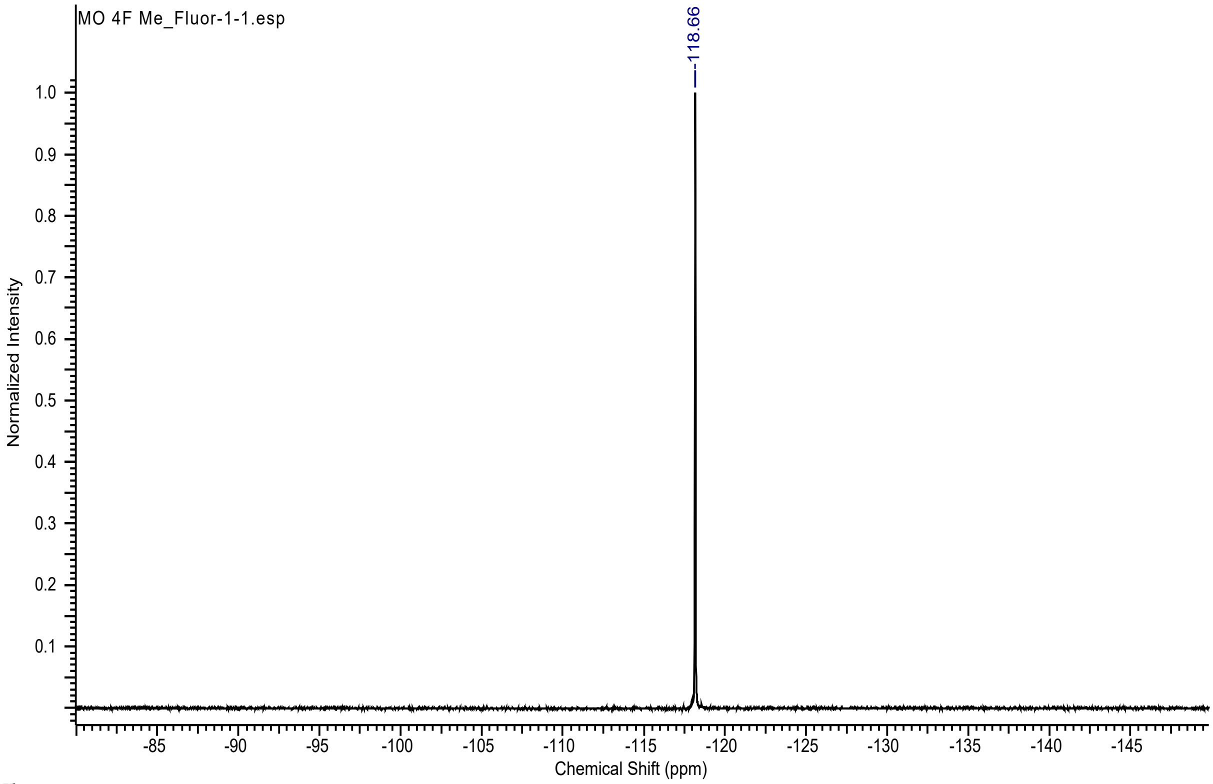

MO 4F Me_Fluor-1-1.esp
-118.66
Normalized Intensity
Chemical Shift (ppm)

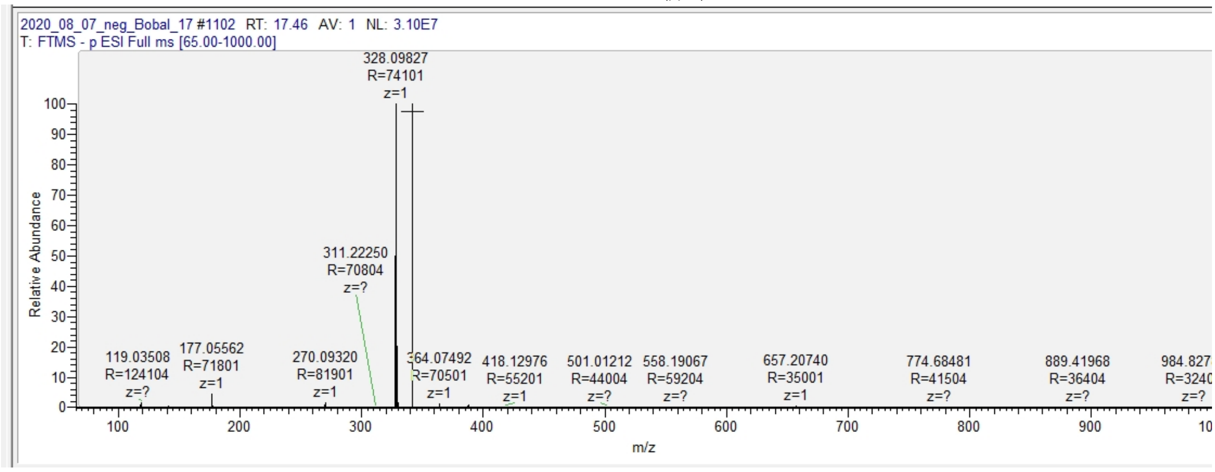

2020_08_07_neg_Bobal_17 #1102 RT: 17.46 AV: 1 NL: 3.10E7
T: FTMS - p ESI Full ms [65.00-1000.00]
328.09827
R=74101
z=1
311.22250
R=70804
z=?
Relative Abundance
m/z

Methyl (2*E*)-3-(4-{[(2-chlorophenyl)carbamoyl]methoxy}phenyl)prop-2-enoate (**10k**)

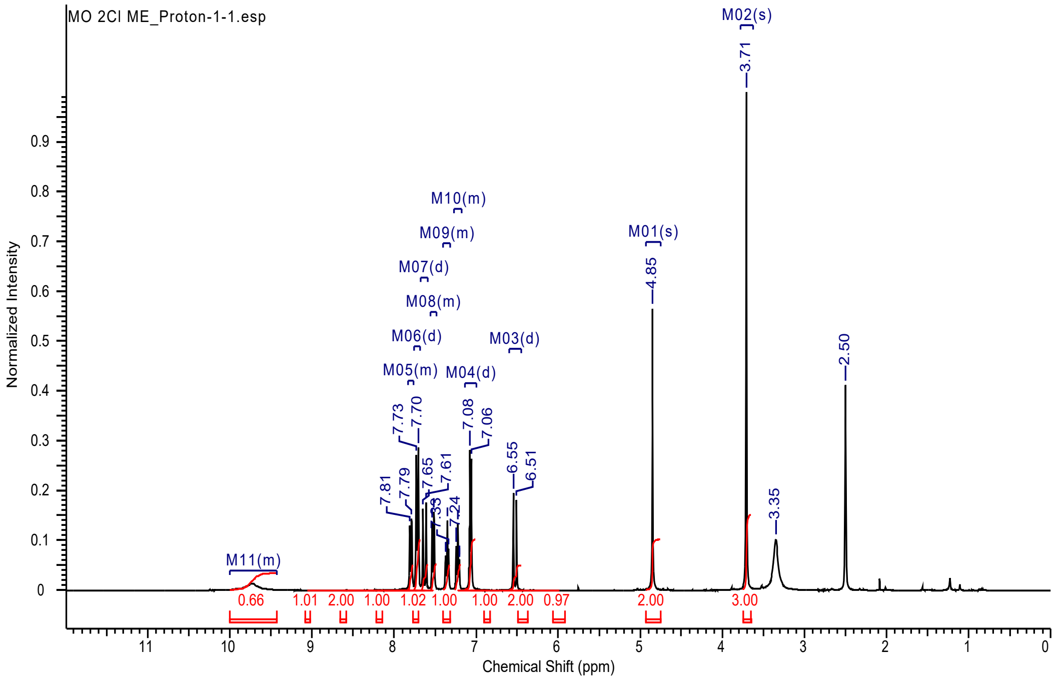

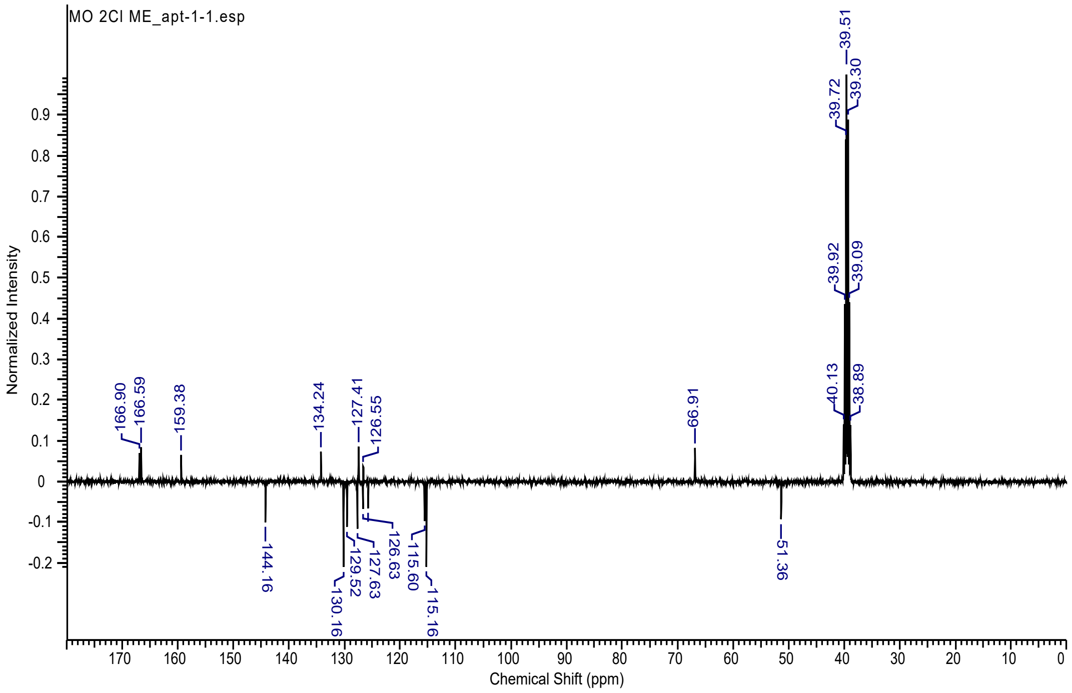

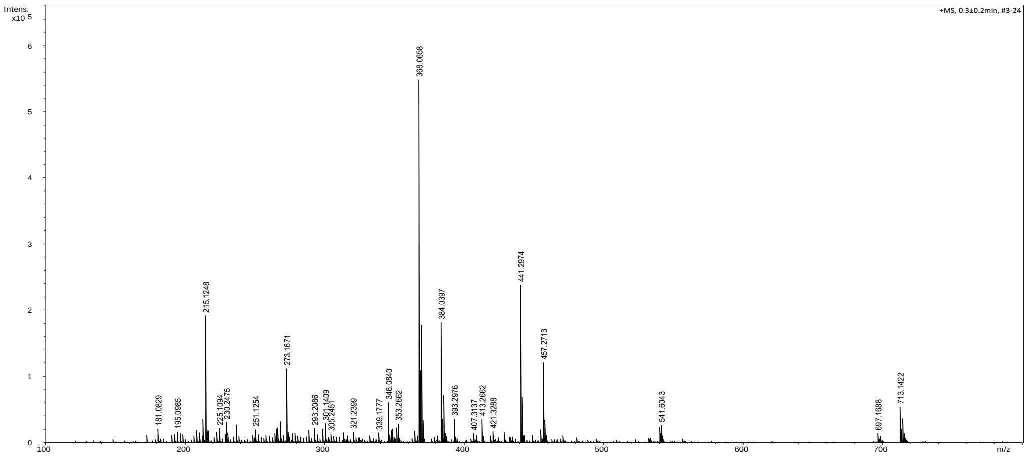

Chromatogram and Results

Injection Details

Injection Name:	**MO 2-Cl ME**	*Run Time (min):*	**30,00**
Vial Number:	**GE8**	*Injection Volume:*	**5,00**
Injection Type:	**Unknown**	*Channel:*	**UV_VIS_1**
Calibration Level:		*Wavelength:*	**210,0**
Instrument Method:	**Grad40-60to90-10 MeCN-H2O**	*Bandwidth:*	**2**
Processing Method:	**New Processing Method**	*Dilution Factor:*	**1,0000**
Injection Date/Time:	**20.10.22 11:49**	*Sample Weight:*	**1,0000**

Chromatogram

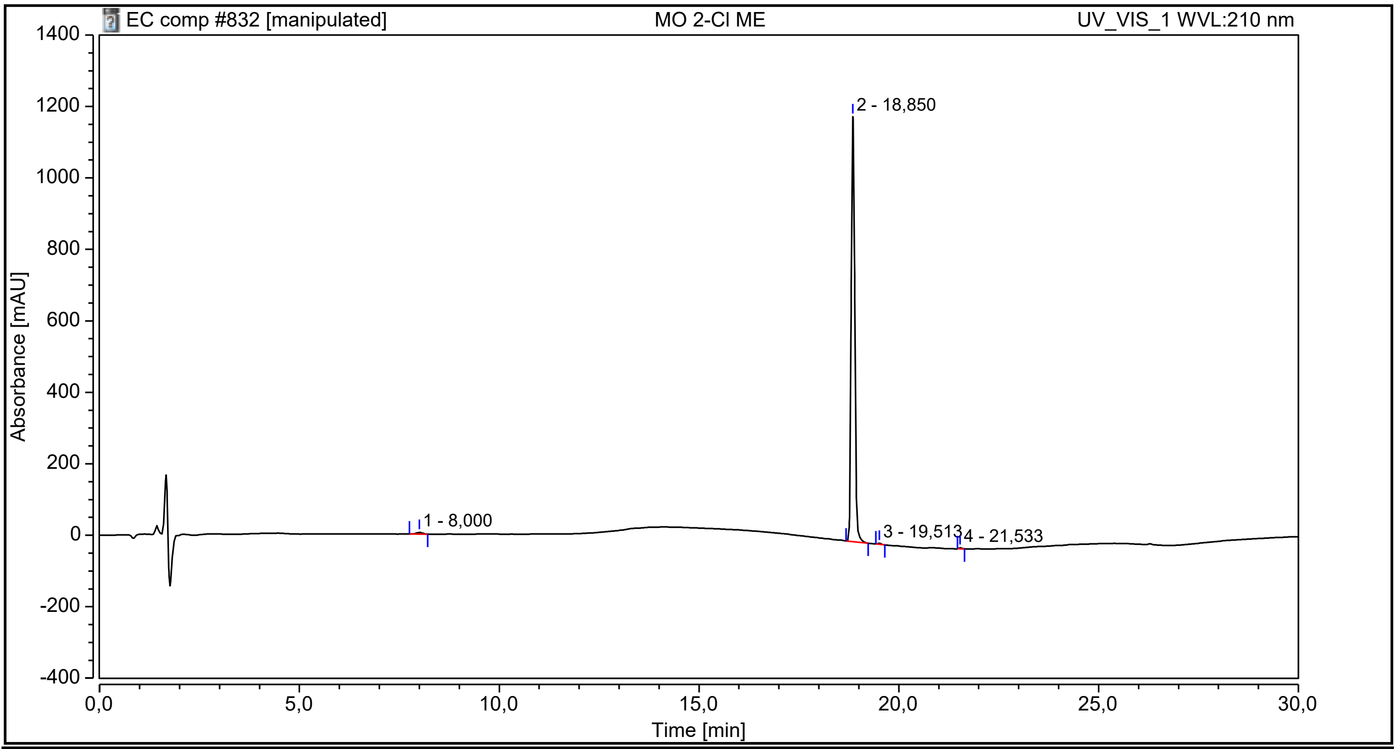

Integration Results

No.	Peak Name	Retention Time<br>min	Area<br>mAU*min	Height<br>mAU	Relative Area<br>%	Relative Height<br>%	Amount<br>n.a.
1	n.a.	8,000	0,796	4,468	0,640	0,37	n.a.
2	n.a.	18,850	123,003	1188,911	98,965	99,13	n.a.
3	n.a.	19,513	0,279	3,365	0,224	0,28	n.a.
4	n.a.	21,533	0,212	2,644	0,170	0,22	n.a.
**Total:**			**124,289**	**1199,388**	**100,00**	**100,00**	

Methyl (2*E*)-3-(4-{[(3-chlorophenyl)carbamoyl]methoxy}phenyl)prop-2-enoate (**10l**)

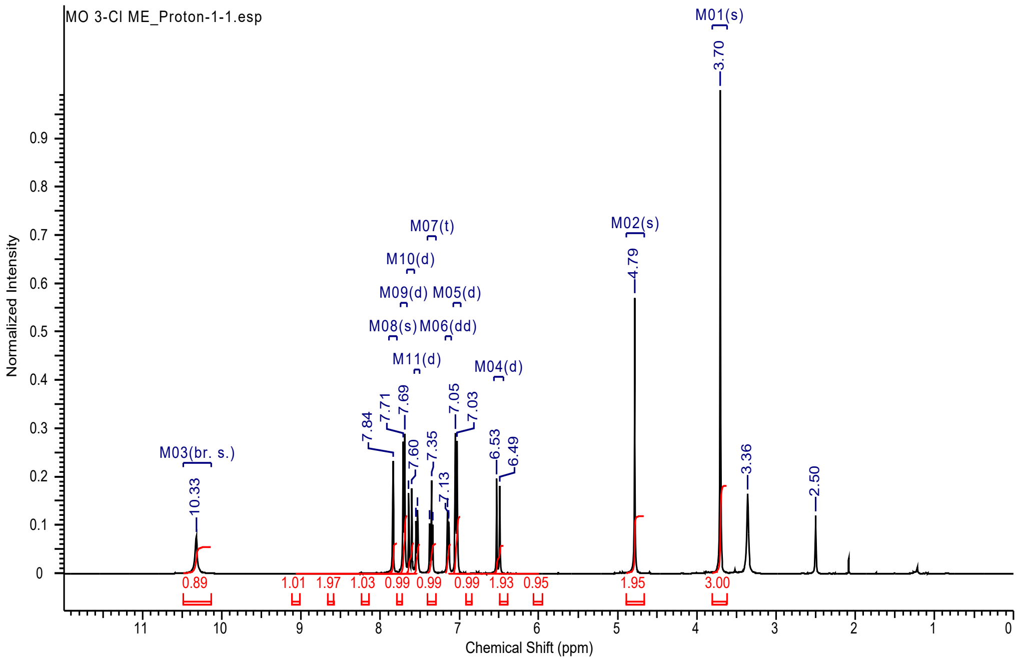

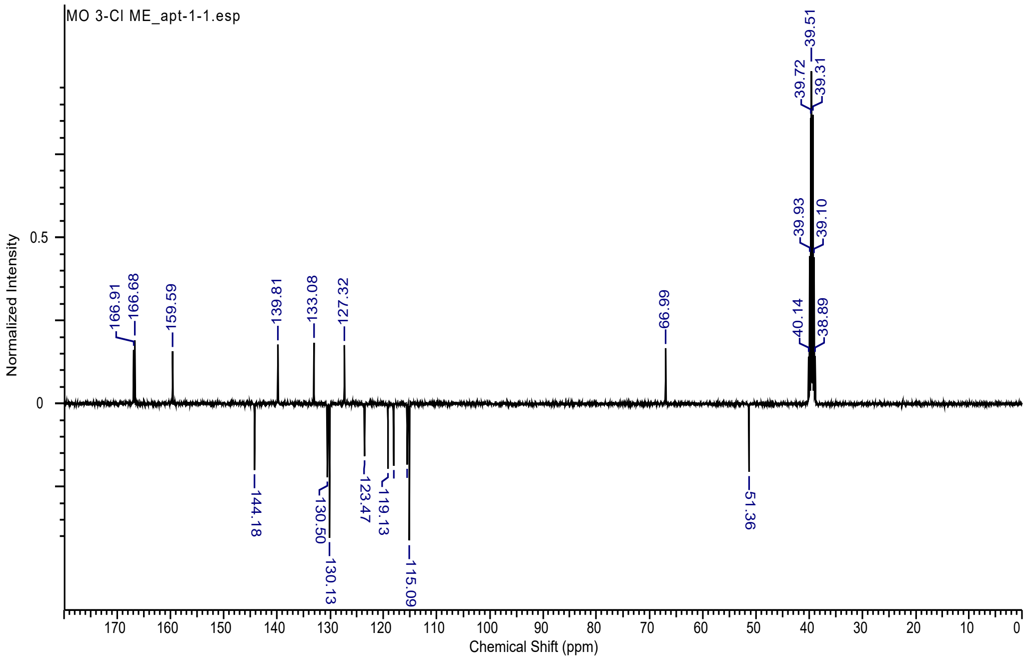

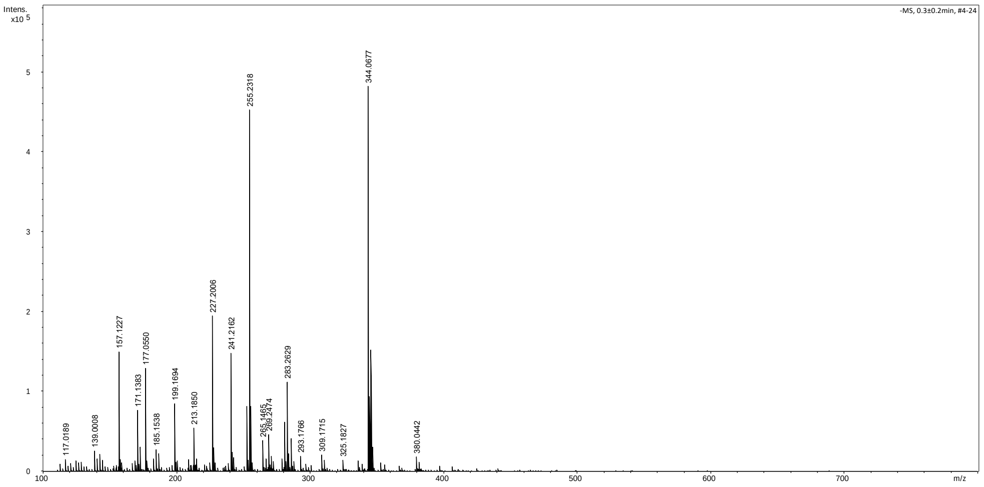

Methyl (2*E*)-3-(4-{[(4-chlorophenyl)carbamoyl]methoxy}phenyl)prop-2-enoate (**10m**)

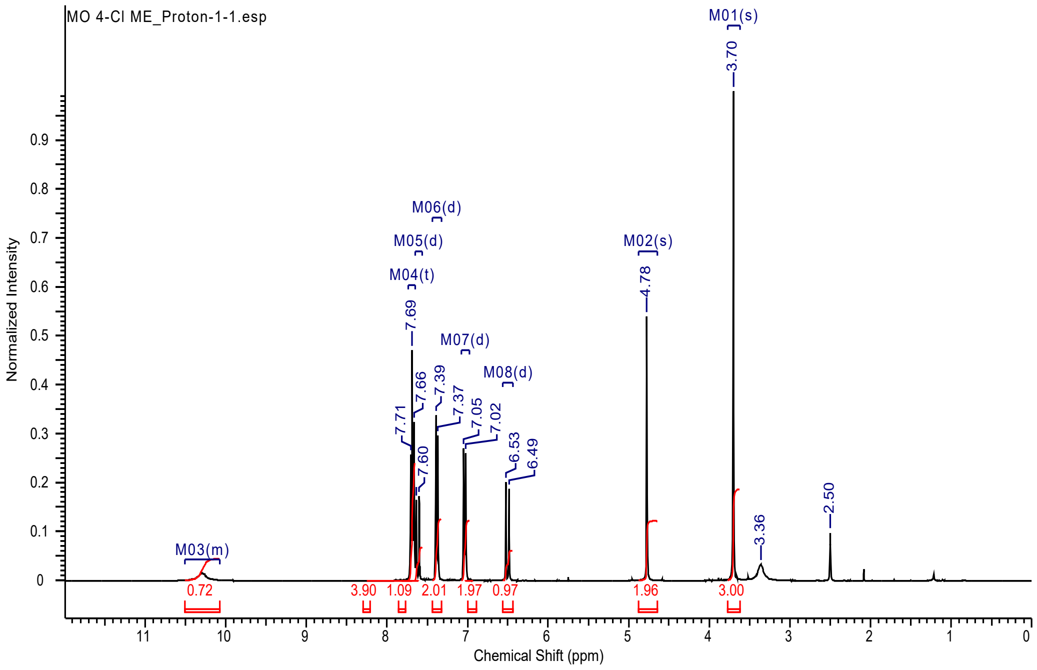

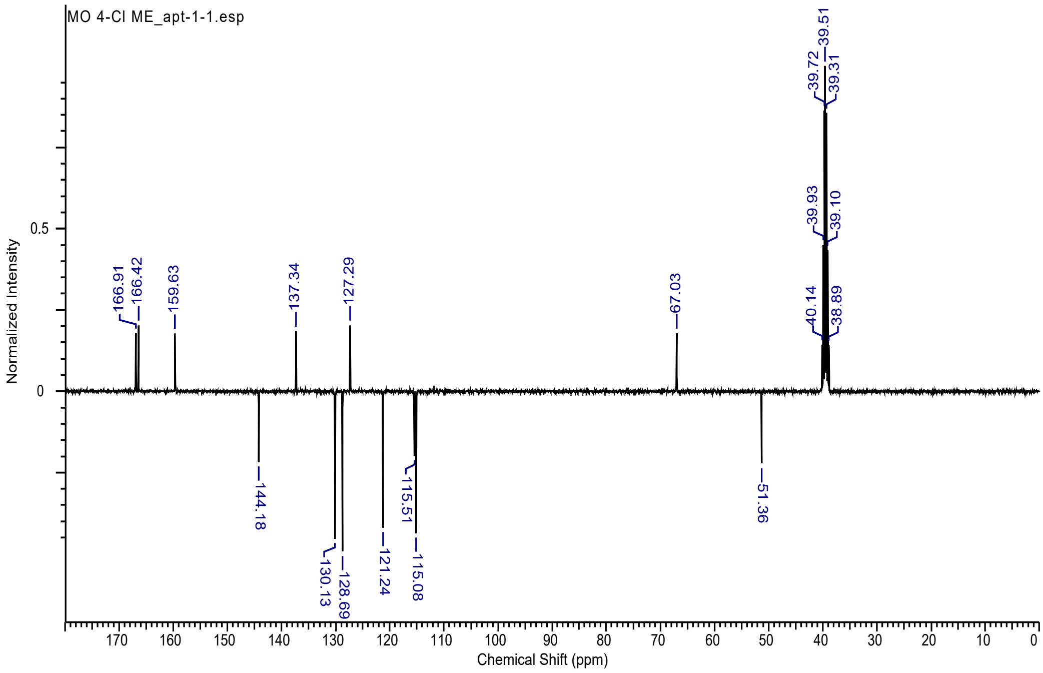

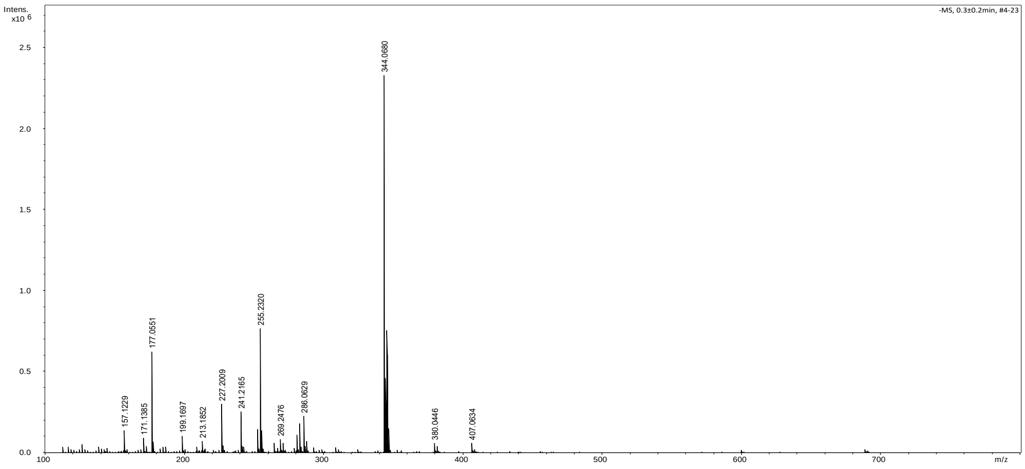

Chromatogram and Results

Injection Details

Injection Name:	**MO 4-Cl ME II**	*Run Time (min):*	**30,00**
Vial Number:	**RA4**	*Injection Volume:*	**5,00**
Injection Type:	**Unknown**	*Channel:*	**UV_VIS_1**
Calibration Level:		*Wavelength:*	**210,0**
Instrument Method:	**Grad40-60to90-10 MeCN-H2O**	*Bandwidth:*	**2**
Processing Method:	**New Processing Method**	*Dilution Factor:*	**1,0000**
Injection Date/Time:	**20.10.22 12:20**	*Sample Weight:*	**1,0000**

Chromatogram

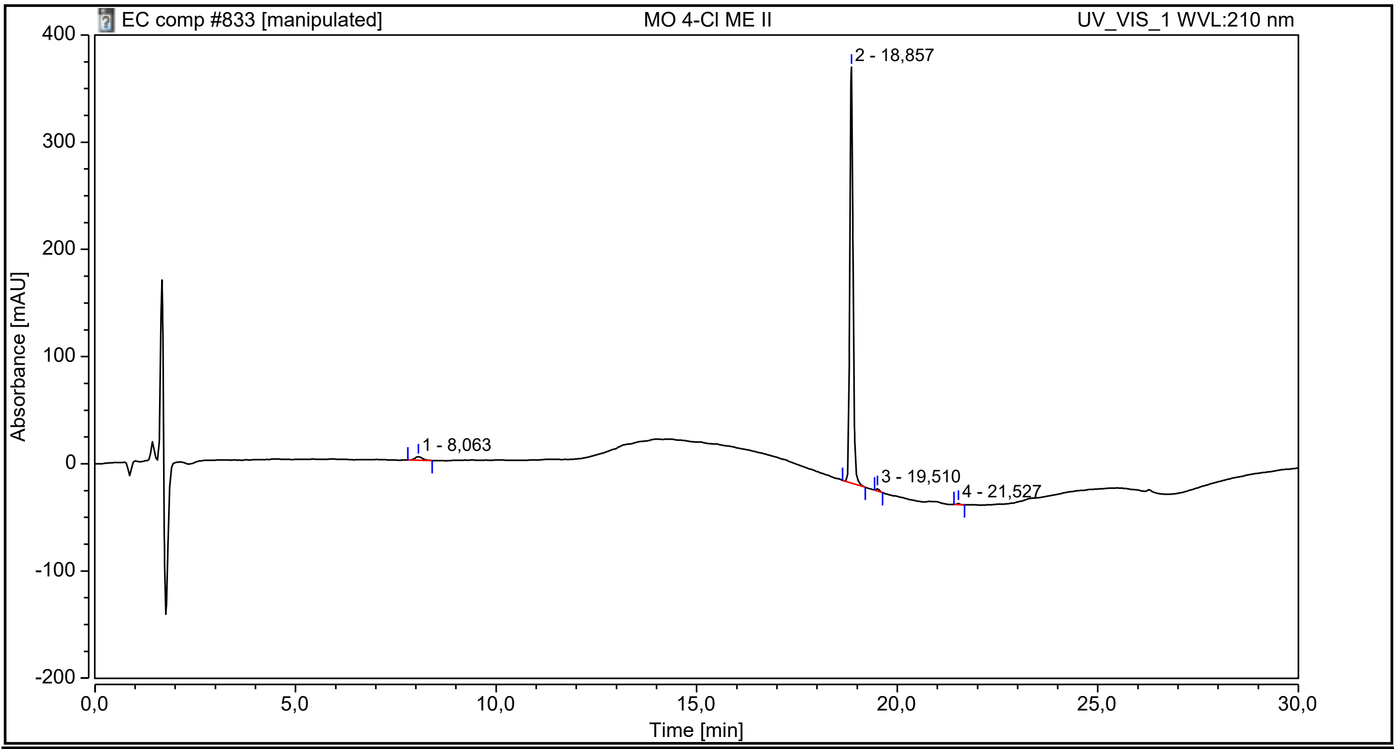

Integration Results

No.	Peak Name	Retention Time min	Area mAU*min	Height mAU	Relative Area %	Relative Height %	Amount n.a.
1	n.a.	8,063	0,682	3,307	1,894	0,84	n.a.
2	n.a.	18,857	35,036	388,122	97,324	98,24	n.a.
3	n.a.	19,510	0,197	2,414	0,548	0,61	n.a.
4	n.a.	21,527	0,084	1,220	0,235	0,31	n.a.
**Total:**			**35,999**	**395,062**	**100,00**	**100,00**	

Methyl (2*E*)-3-(4-{[(2-bromophenyl)carbamoyl]methoxy}phenyl)prop-2-enoate (**10n**)

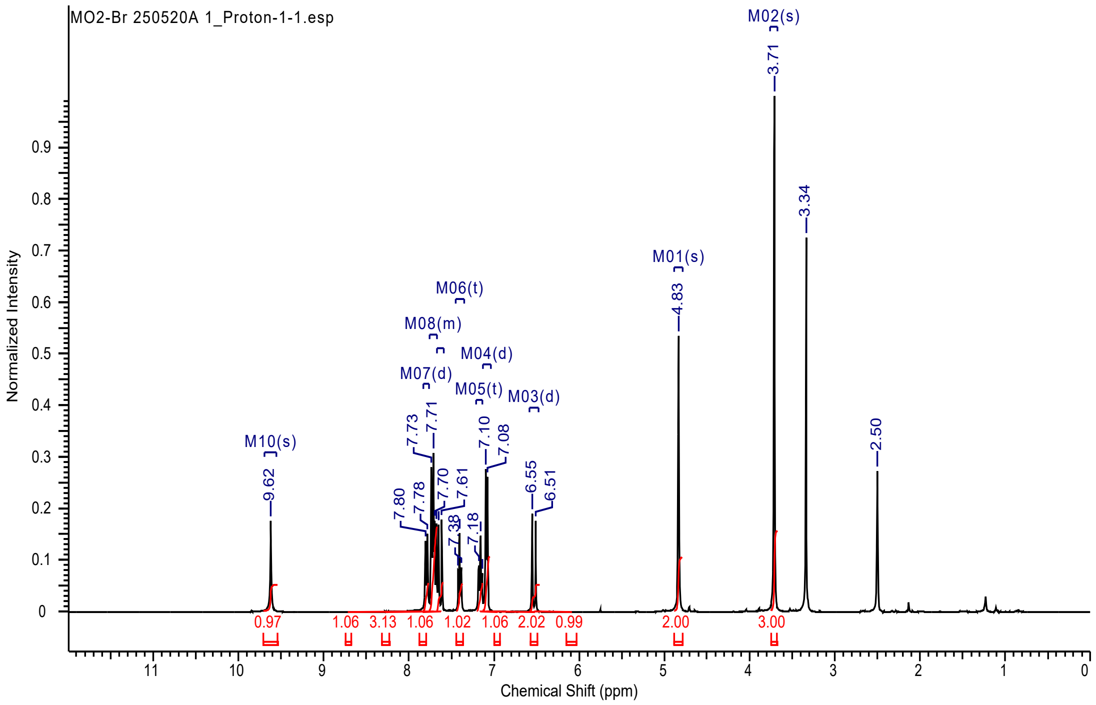

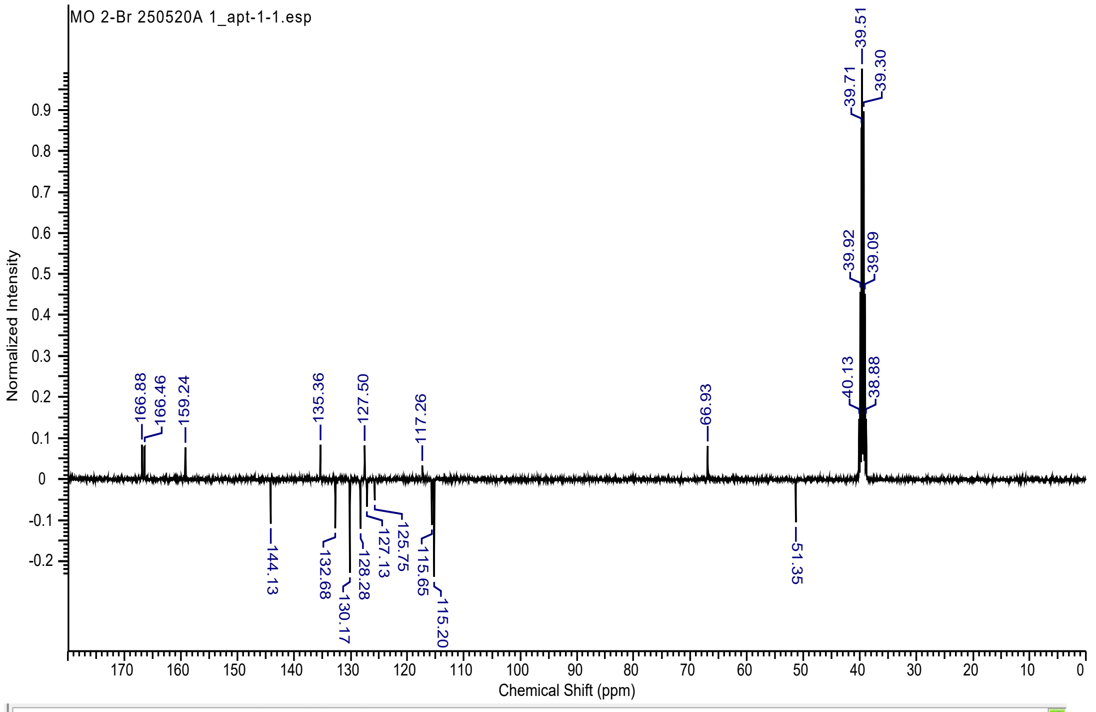

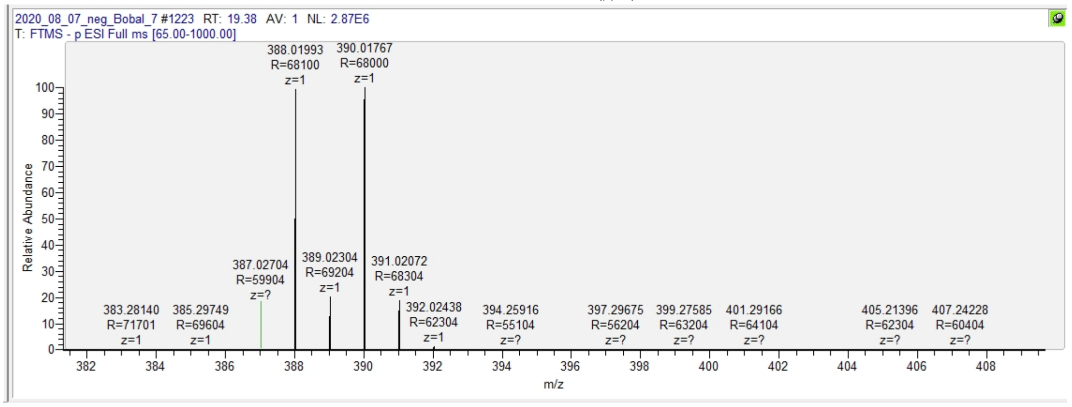

Chromatogram and Results

Injection Details

Injection Name:	**MO 2-Br ME**	*Run Time (min):*	**30,00**
Vial Number:	**GC7**	*Injection Volume:*	**10,00**
Injection Type:	**Unknown**	*Channel:*	**UV_VIS_1**
Calibration Level:		*Wavelength:*	**210,0**
Instrument Method:	**Grad40-60to70-30 MeCN-H2O**	*Bandwidth:*	**2**
Processing Method:	**New Processing Method**	*Dilution Factor:*	**1,0000**
Injection Date/Time:	**09.12.20 22:56**	*Sample Weight:*	**1,0000**

Chromatogram

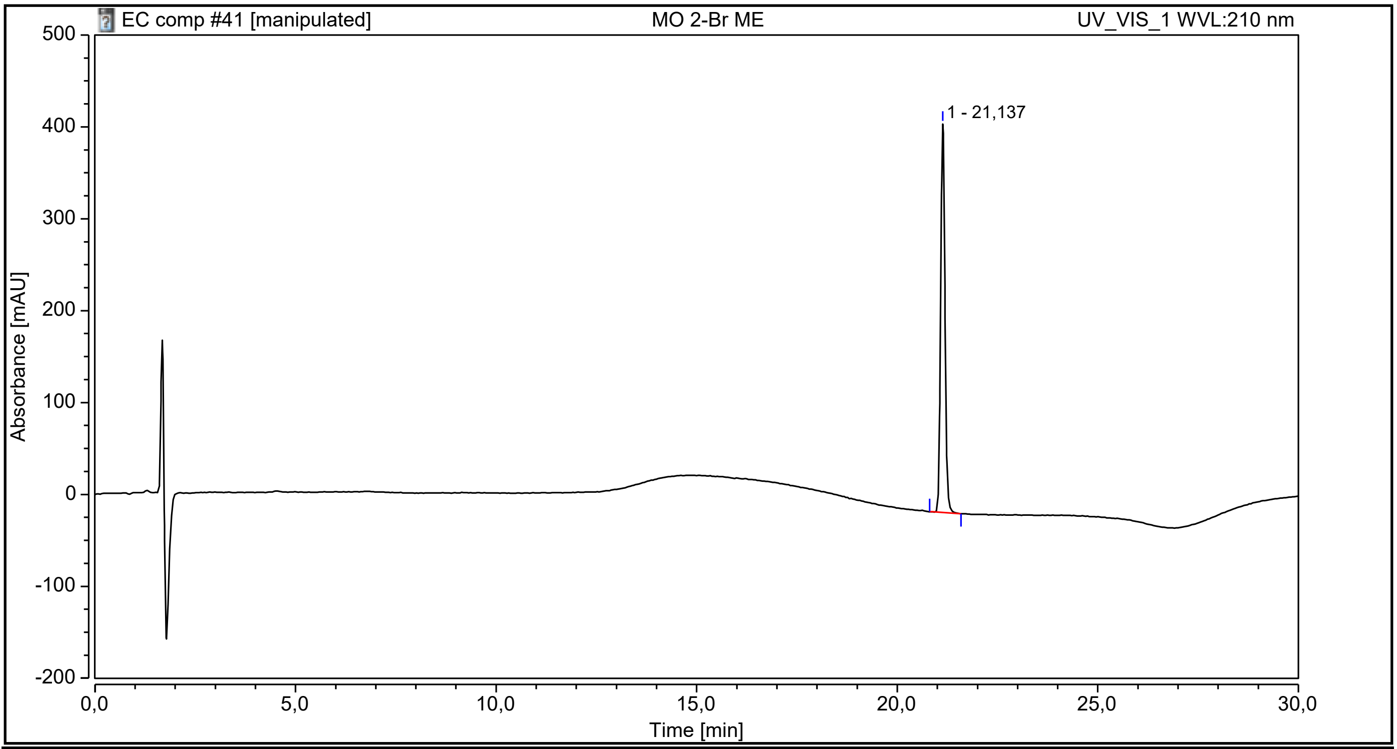

Integration Results

No.	Peak Name	Retention Time min	Area mAU*min	Height mAU	Relative Area %	Relative Height %	Amount n.a.
1	n.a.	21,137	52,428	422,751	100,000	100,00	n.a.
**Total:**			**52,428**	**422,751**	**100,00**	**100,00**	

Methyl (2*E*)-3-(4-{[(3-bromophenyl)carbamoyl]methoxy}phenyl)prop-2-enoate (**10o**)

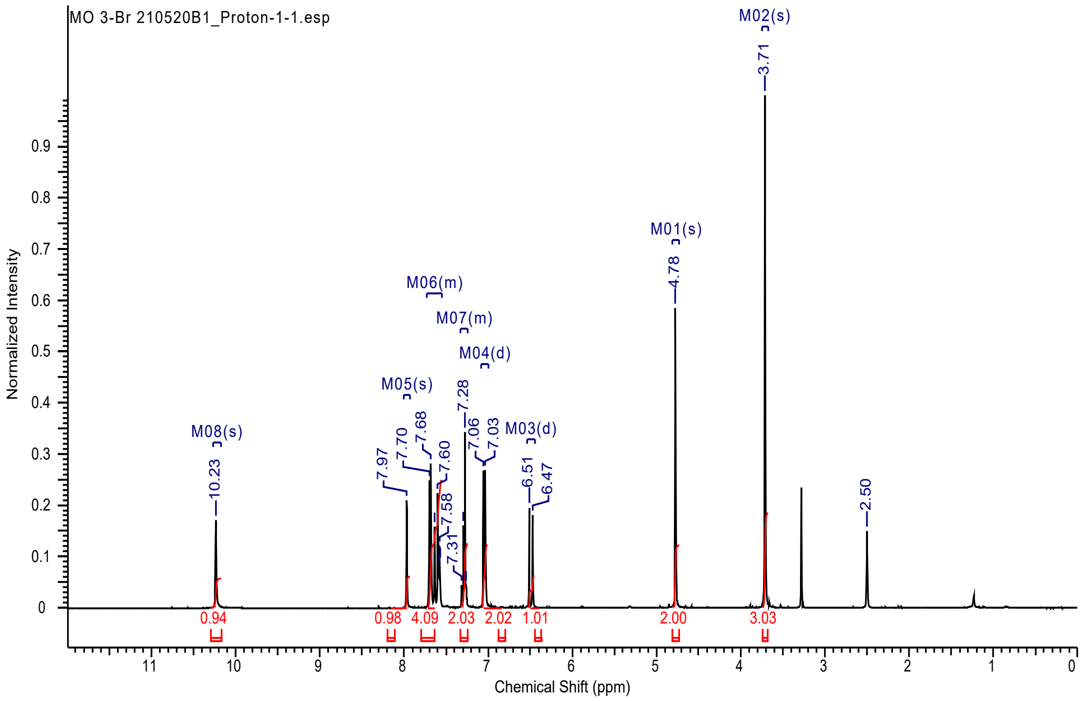

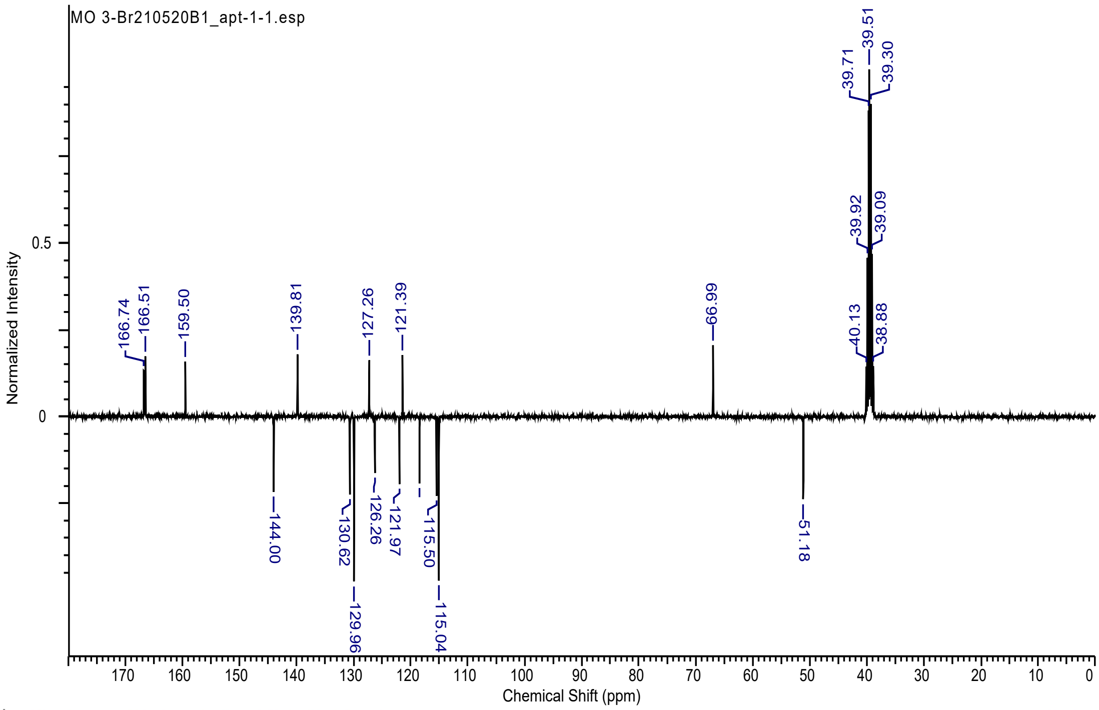

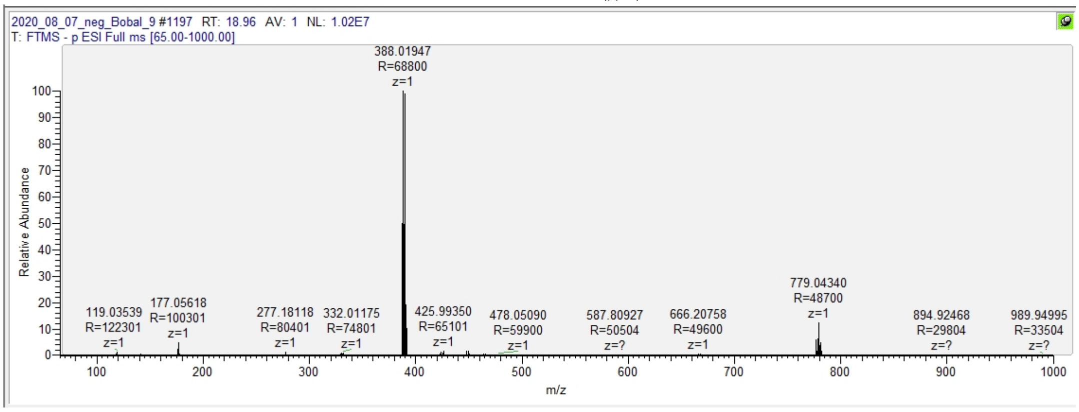

Chromatogram and Results

Injection Details

Injection Name:	**MO 3-Br ME**	*Run Time (min):*	**30,00**
Vial Number:	**GC8**	*Injection Volume:*	**10,00**
Injection Type:	**Unknown**	*Channel:*	**UV_VIS_1**
Calibration Level:		*Wavelength:*	**210,0**
Instrument Method:	**Grad40-60to70-30 MeCN-H2O**	*Bandwidth:*	**2**
Processing Method:	**New Processing Method**	*Dilution Factor:*	**1,0000**
Injection Date/Time:	**09.12.20 23:27**	*Sample Weight:*	**1,0000**

Chromatogram

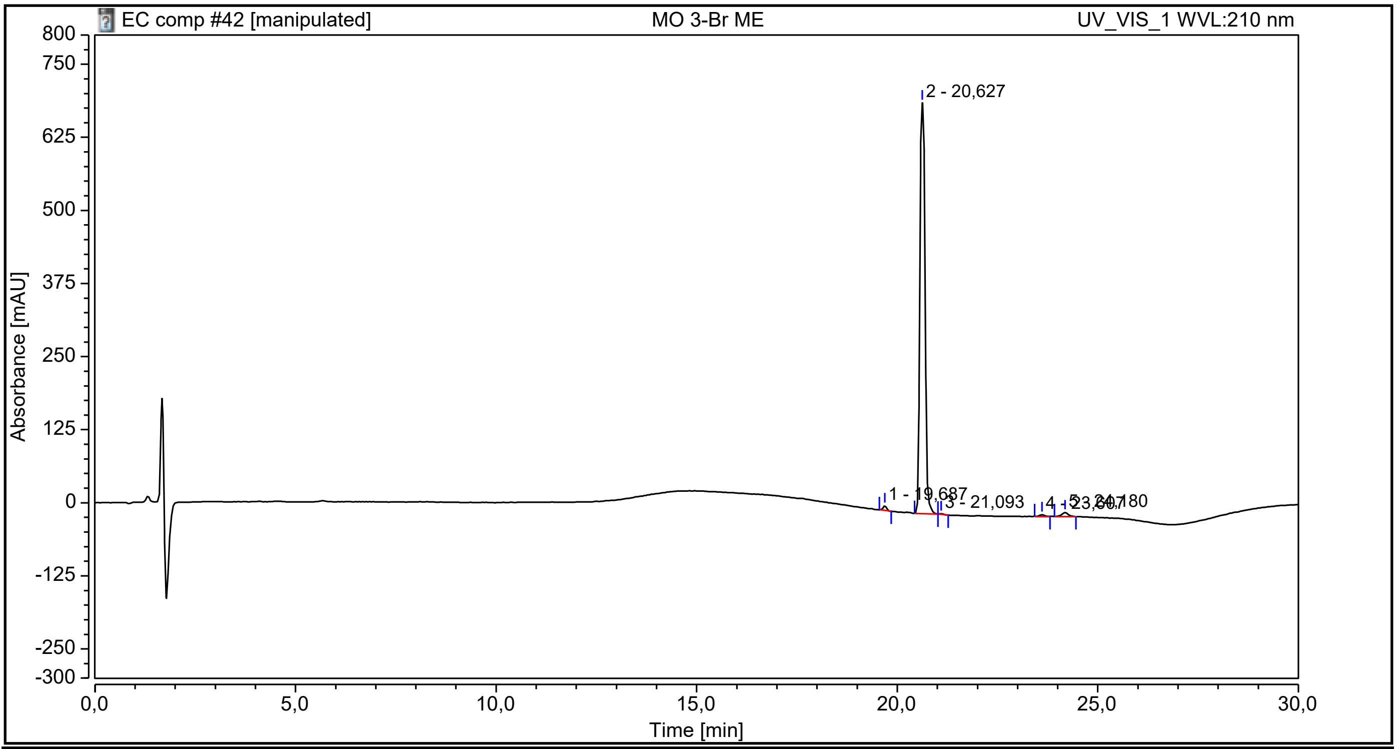

Integration Results

No.	Peak Name	Retention Time<br>min	Area<br>mAU*min	Height<br>mAU	Relative Area<br>%	Relative Height<br>%	Amount<br>n.a.
1	n.a.	19,687	0,788	7,821	0,724	1,08	n.a.
2	n.a.	20,627	106,142	702,498	97,495	97,37	n.a.
3	n.a.	21,093	0,158	1,306	0,145	0,18	n.a.
4	n.a.	23,607	0,466	3,057	0,428	0,42	n.a.
5	n.a.	24,180	1,315	6,806	1,208	0,94	n.a.
**Total:**			**108,869**	**721,489**	**100,00**	**100,00**	

Methyl (2*E*)-3-(4-{[(4-bromophenyl)carbamoyl]methoxy}phenyl)prop-2-enoate (**10p**)

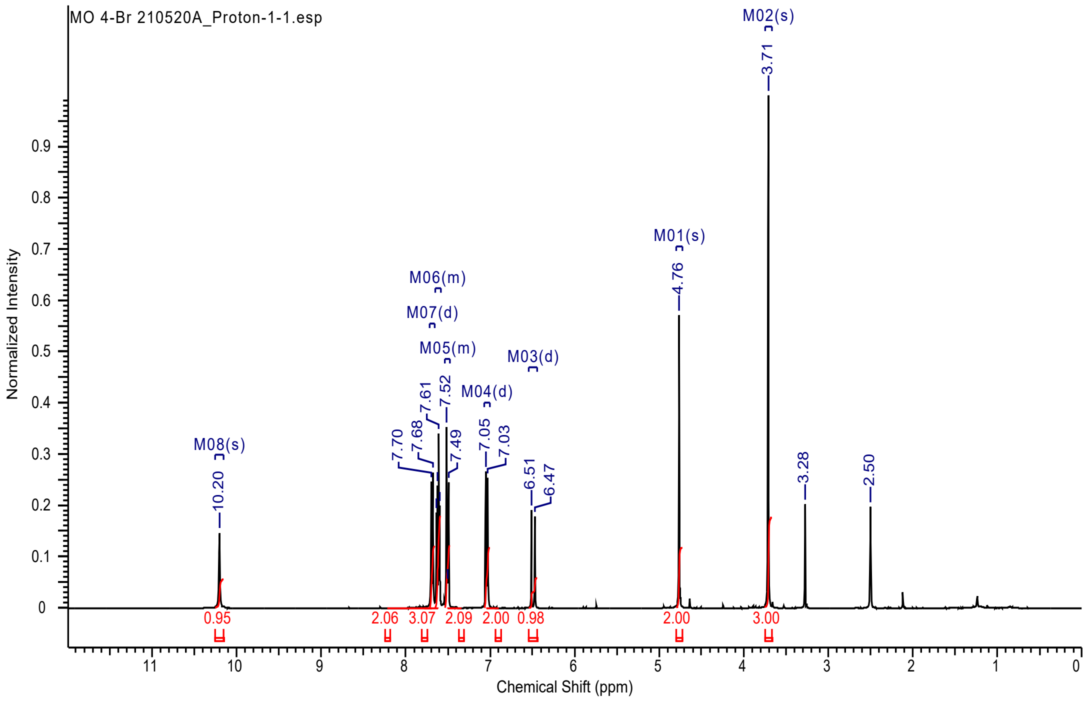

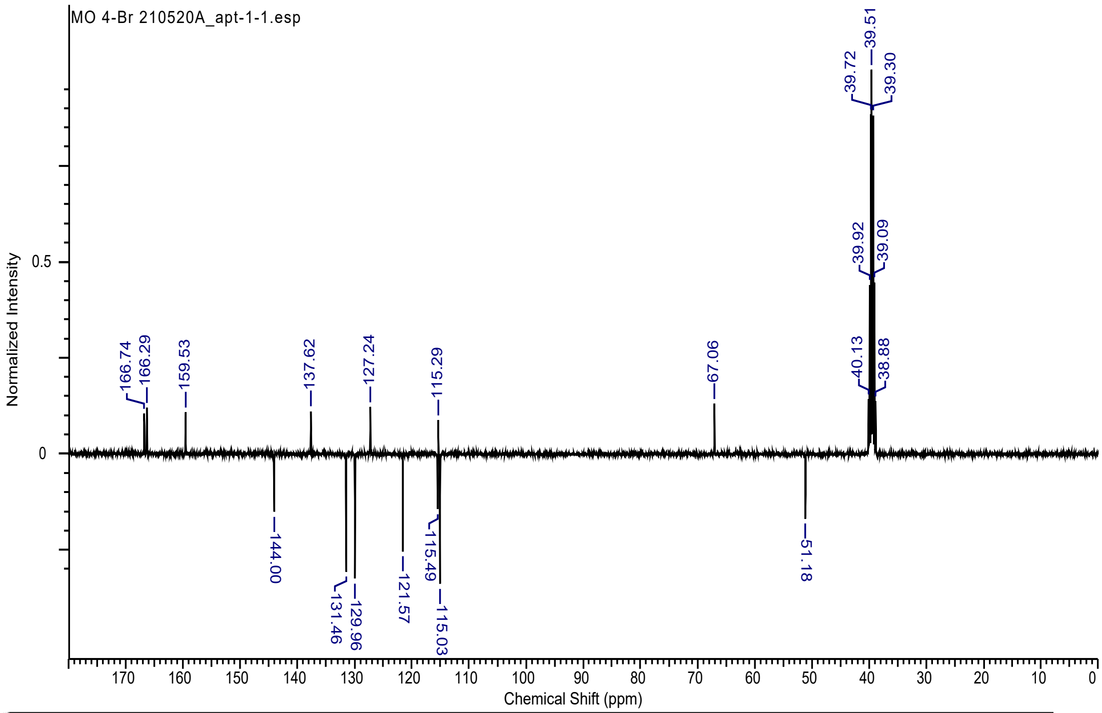

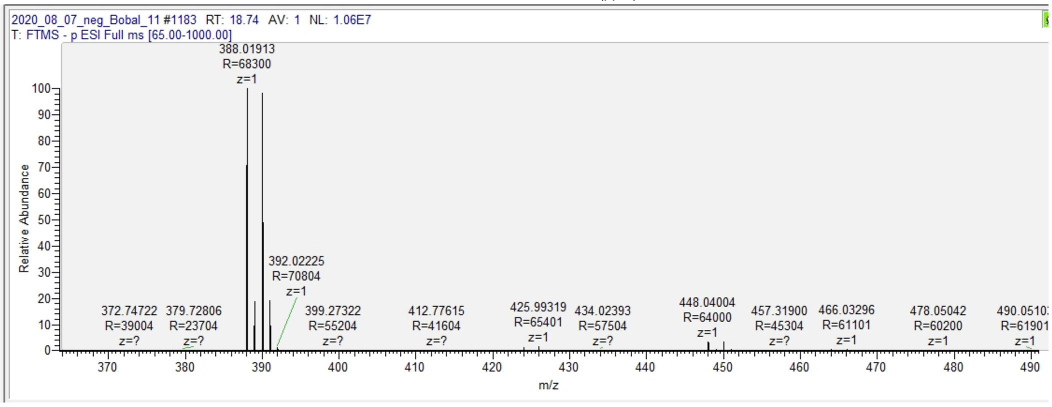

Methyl (2*E*)-3-[4-({[2-(trifluoromethyl)phenyl]carbamoyl}methoxy)phenyl]prop-2-enoate (**10q**)

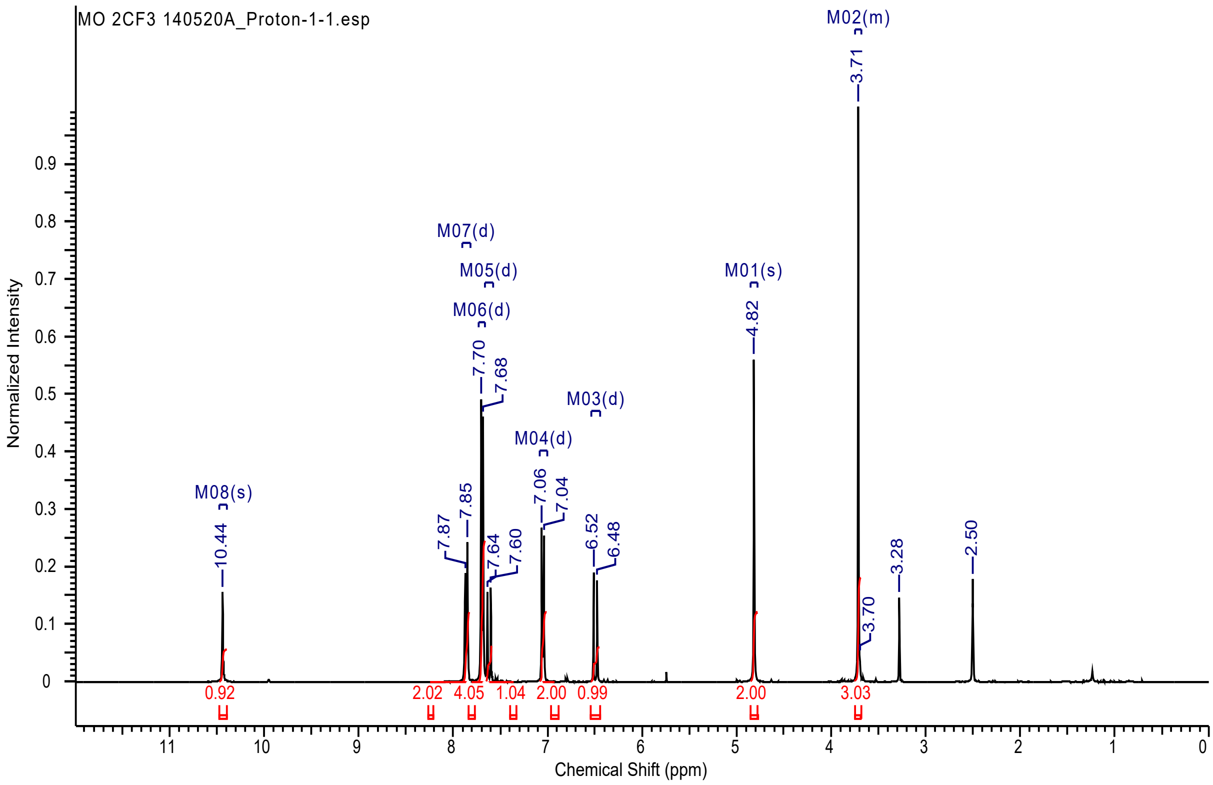

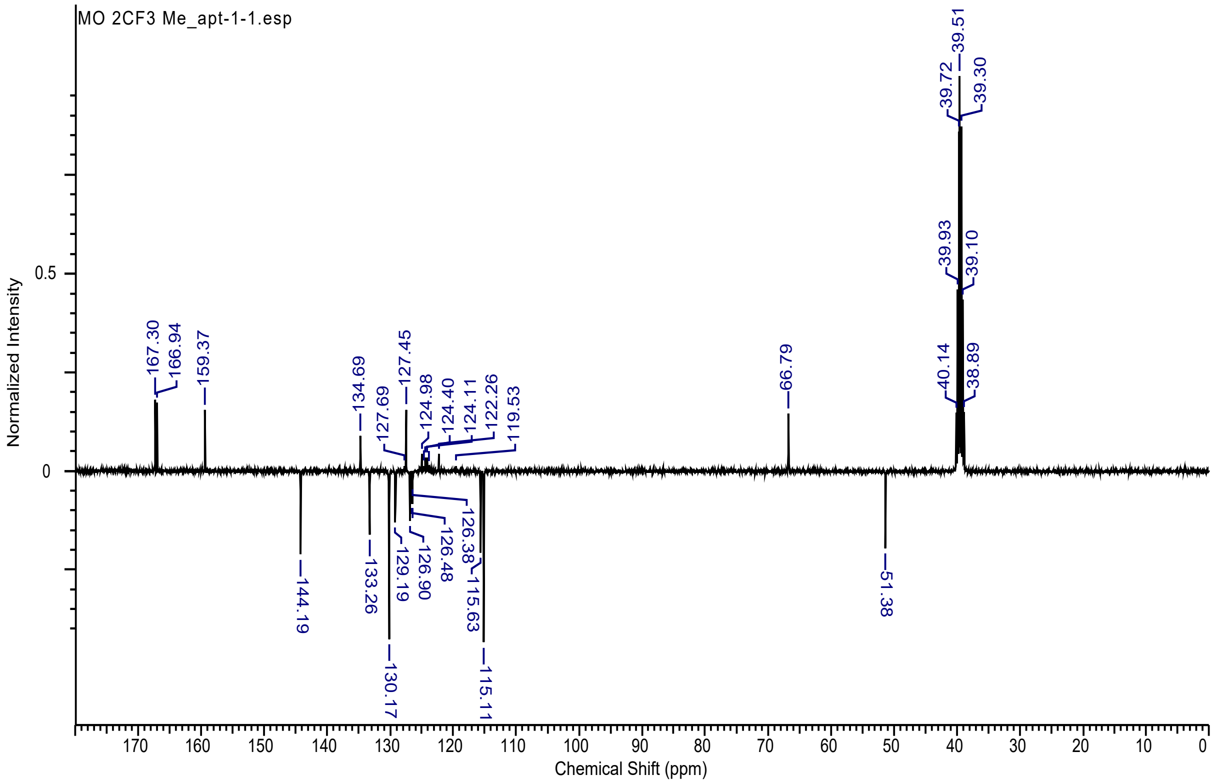

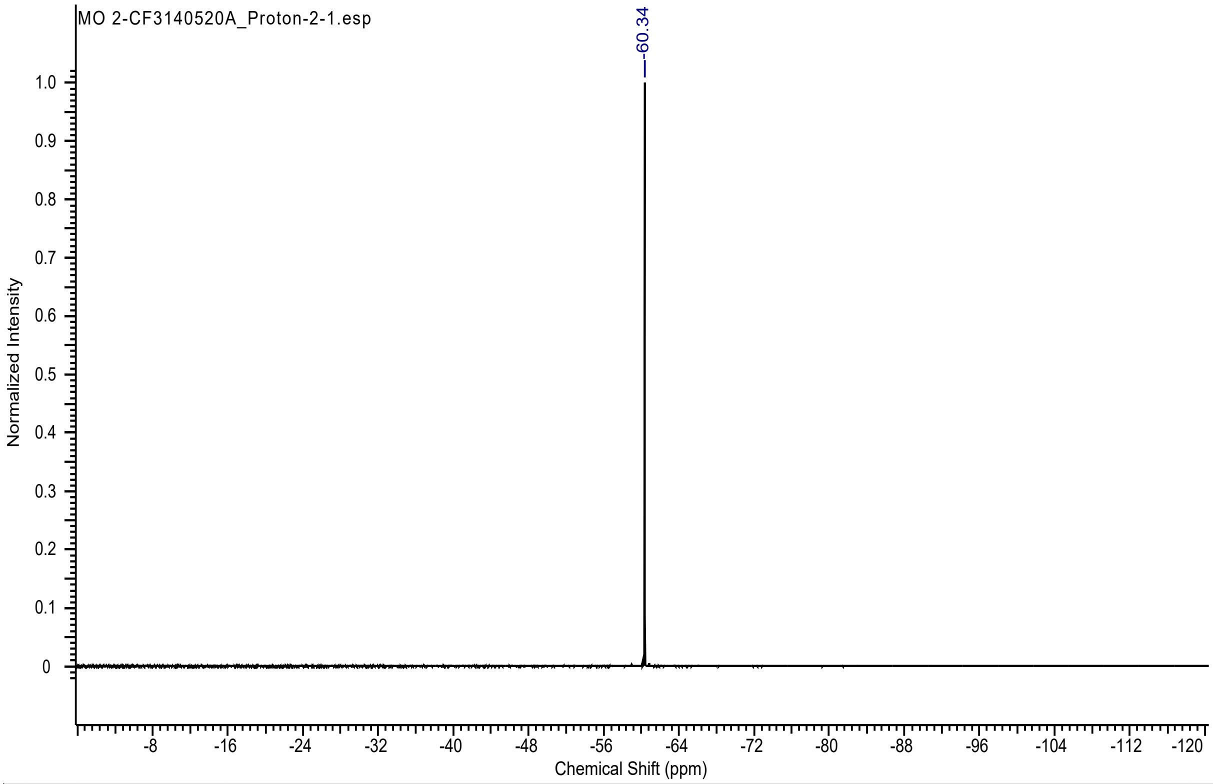
MO 2-CF3140520A_Proton-2-1.esp
-60.34
Normalized Intensity
1.0
0.9
0.8
0.7
0.6
0.5
0.4
0.3
0.2
0.1
0
-8
-16
-24
-32
-40
-48
-56
-64
-72
-80
-88
-96
-104
-112
-120
Chemical Shift (ppm)

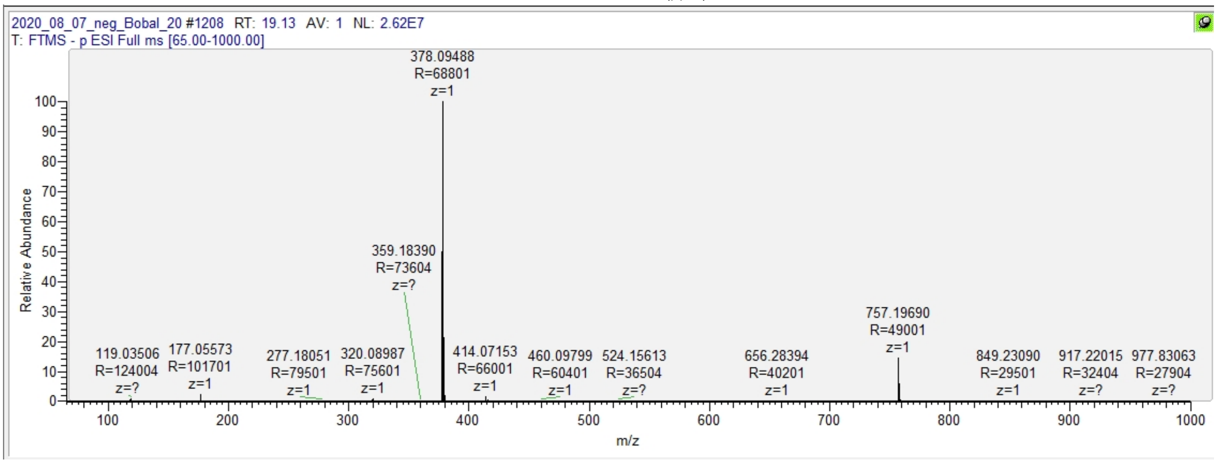
2020_08_07_neg_Bobal_20 #1208 RT: 19.13 AV: 1 NL: 2.62E7
T: FTMS - p ESI Full ms [65.00-1000.00]
378.09488
R=68801
z=1
359.18390
R=73604
z=?
119.03506
R=124004
z=?
177.05573
R=101701
z=1
277.18051
R=79501
z=1
320.08987
R=75601
z=1
414.07153
R=66001
z=1
460.09799
R=60401
z=1
524.15613
R=36504
z=?
656.28394
R=40201
z=1
757.19690
R=49001
z=1
849.23090
R=29501
z=1
917.22015
R=32404
z=?
977.83063
R=27904
z=?
Relative Abundance
m/z

Chromatogram and Results

Injection Details

Injection Name:	**MO 2-CF3 ME**	*Run Time (min):*	**30,00**
Vial Number:	**GD5**	*Injection Volume:*	**10,00**
Injection Type:	**Unknown**	*Channel:*	**UV_VIS_1**
Calibration Level:		*Wavelength:*	**210,0**
Instrument Method:	**Grad40-60to70-30 MeCN-H2O**	*Bandwidth:*	**2**
Processing Method:	**New Processing Method**	*Dilution Factor:*	**1,0000**
Injection Date/Time:	**10.12.20 02:04**	*Sample Weight:*	**1,0000**

Chromatogram

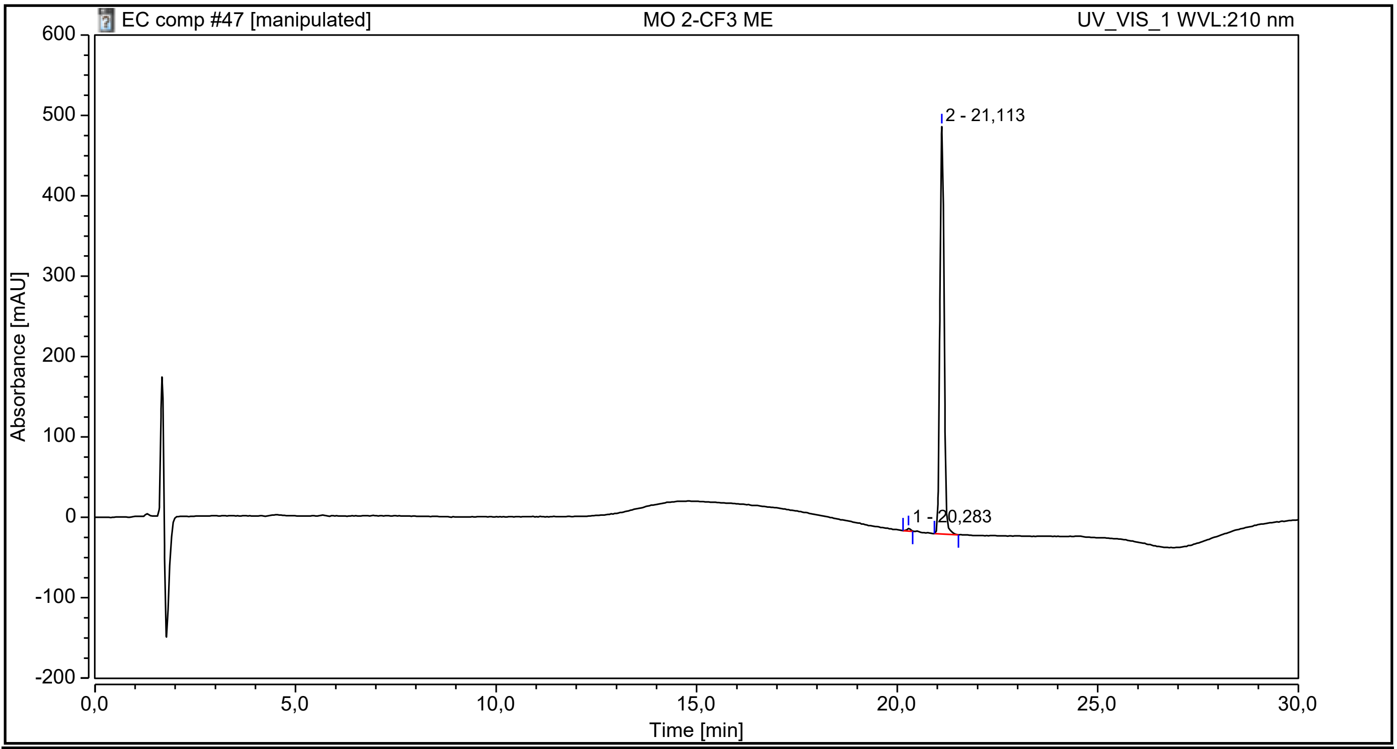

Integration Results

No.	Peak Name	Retention Time min	Area mAU*min	Height mAU	Relative Area %	Relative Height %	Amount n.a.
1	n.a.	20,283	0,350	3,441	0,570	0,67	n.a.
2	n.a.	21,113	60,963	506,534	99,430	99,33	n.a.
**Total:**			**61,312**	**509,975**	**100,00**	**100,00**	

Methyl (2*E*)-3-[4-({[3-(trifluoromethyl)phenyl]carbamoyl}methoxy)phenyl]prop-2-enoate (**10r**)

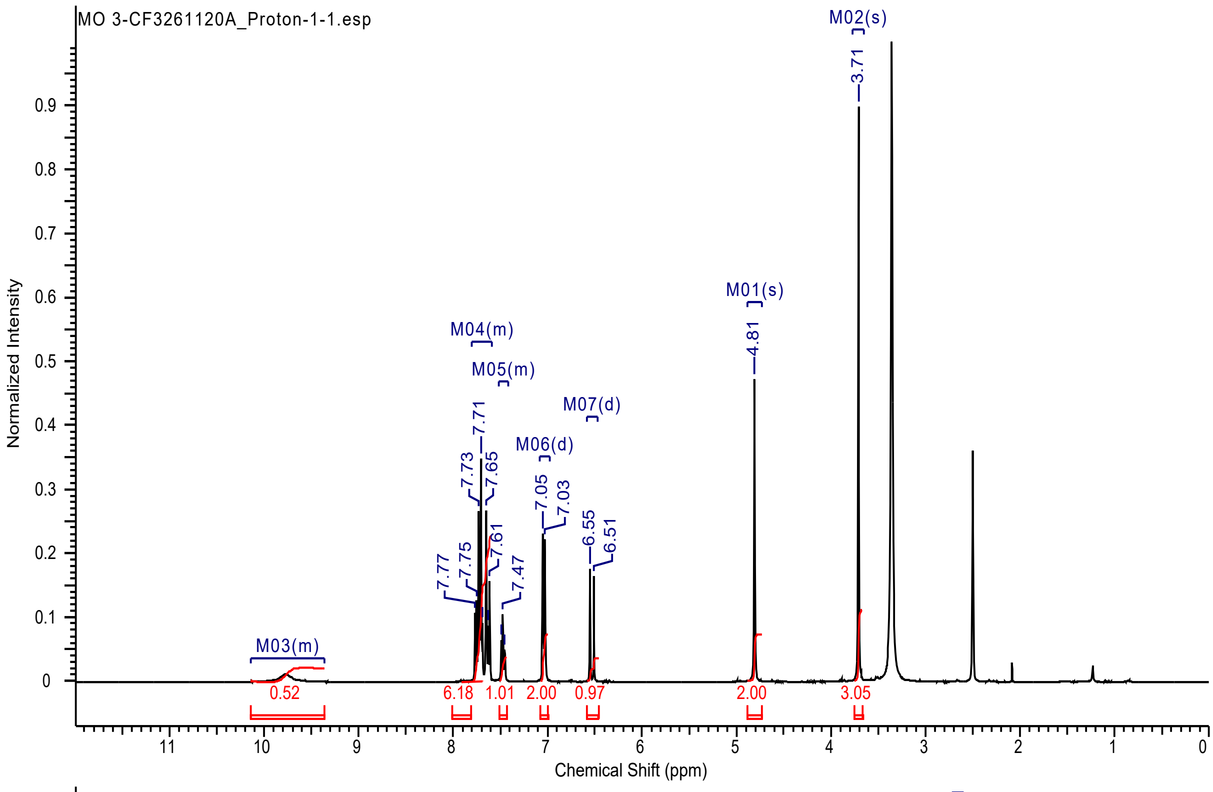

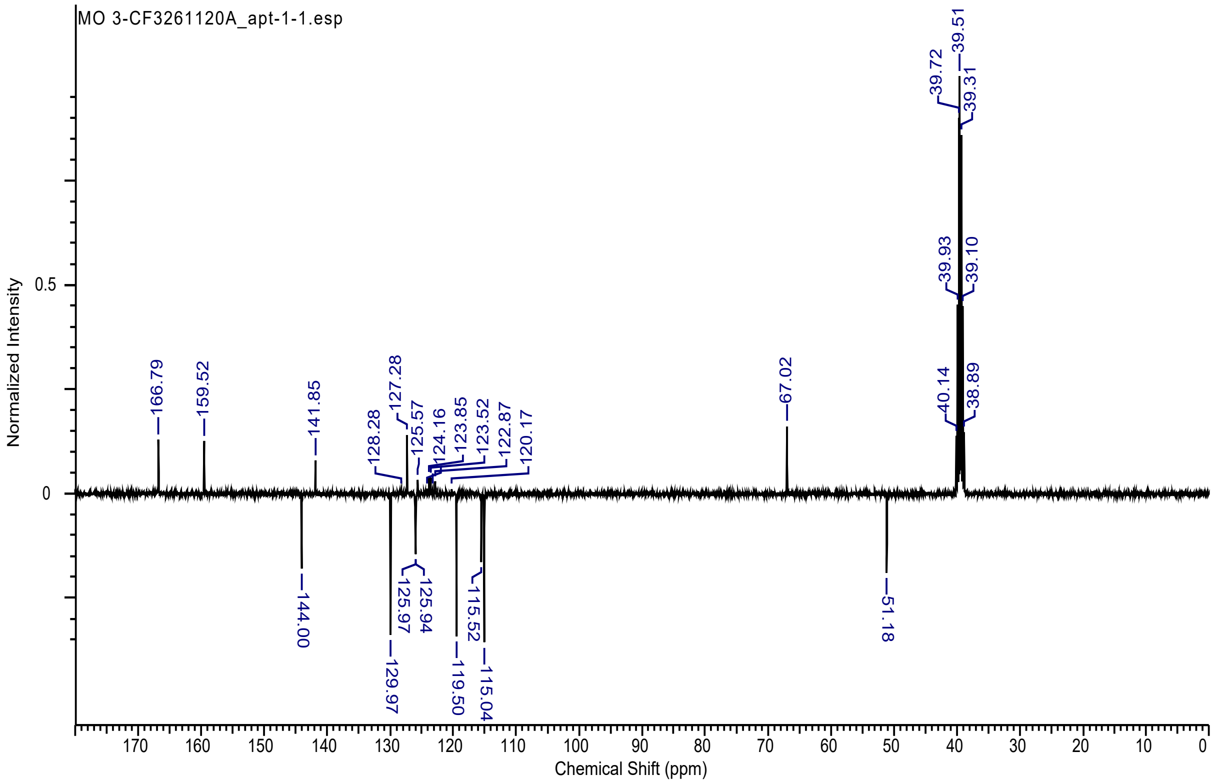

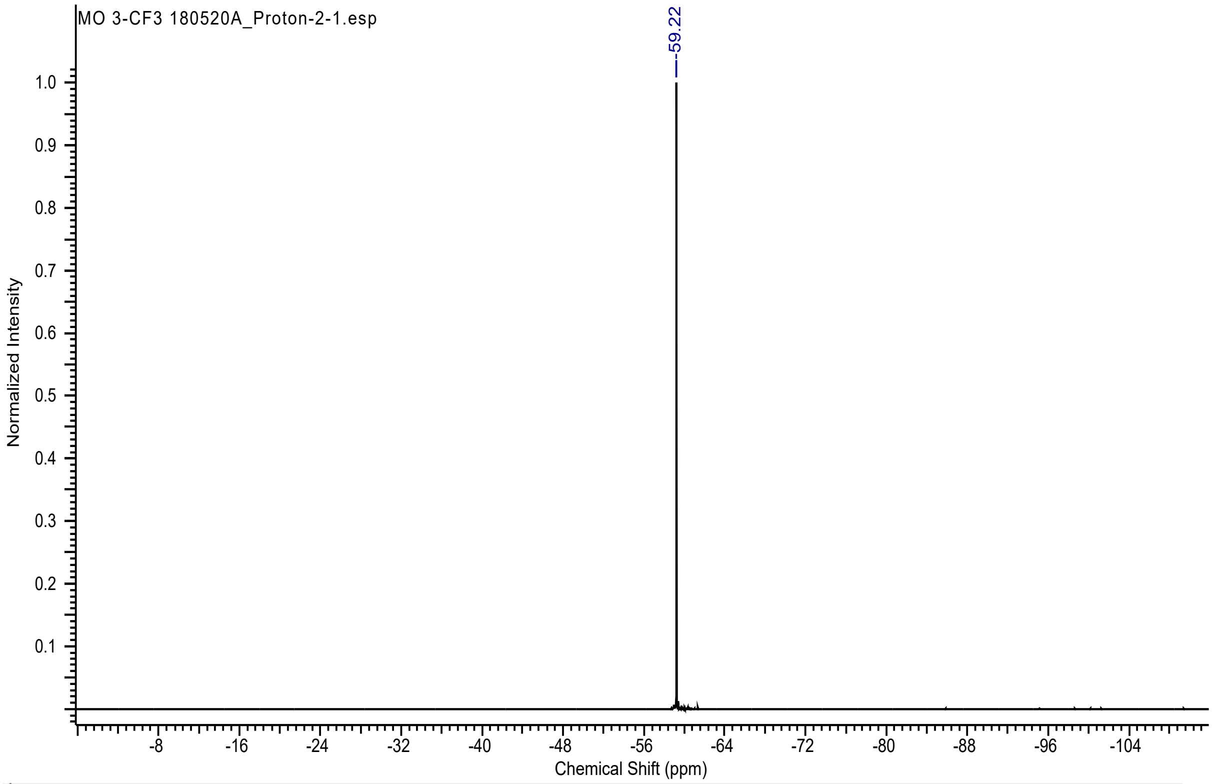
MO 3-CF3 180520A_Proton-2-1.esp
-59.22
Normalized Intensity
1.0
0.9
0.8
0.7
0.6
0.5
0.4
0.3
0.2
0.1
-8
-16
-24
-32
-40
-48
-56
-64
-72
-80
-88
-96
-104
Chemical Shift (ppm)

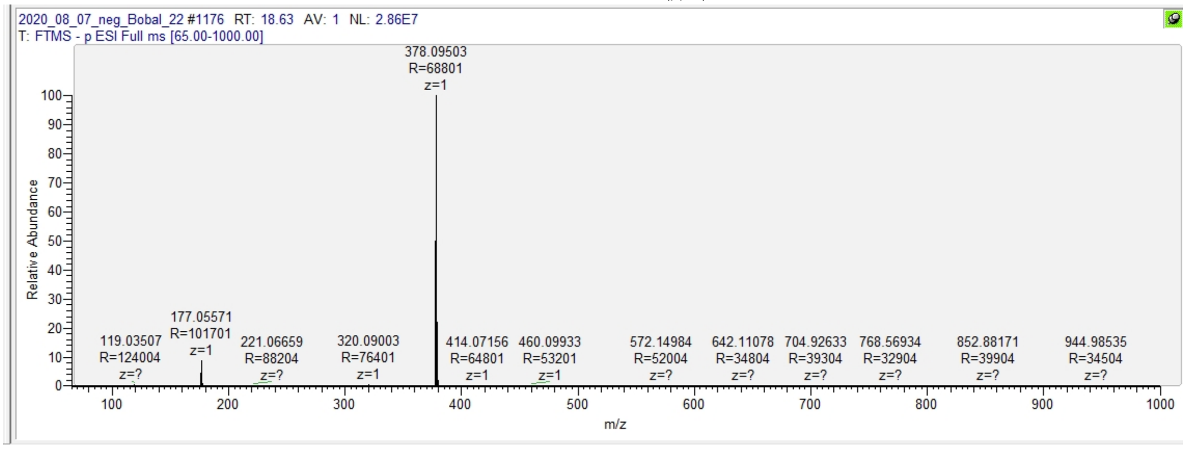
2020_08_07_neg_Bobal_22 #1176 RT: 18.63 AV: 1 NL: 2.86E7
T: FTMS - p ESI Full ms [65.00-1000.00]
378.09503
R=68801
z=1
Relative Abundance
119.03507
R=124004
z=?
177.05571
R=101701
z=1
221.06659
R=88204
z=?
320.09003
R=76401
z=1
414.07156
R=64801
z=1
460.09933
R=53201
z=1
572.14984
R=52004
z=?
642.11078
R=34804
z=?
704.92633
R=39304
z=?
768.56934
R=32904
z=?
852.88171
R=39904
z=?
944.98535
R=34504
z=?
m/z

Methyl (2*E*)-3-[4-({[4-(trifluoromethyl)phenyl]carbamoyl}methoxy)phenyl]prop-2-enoate (**10s**)

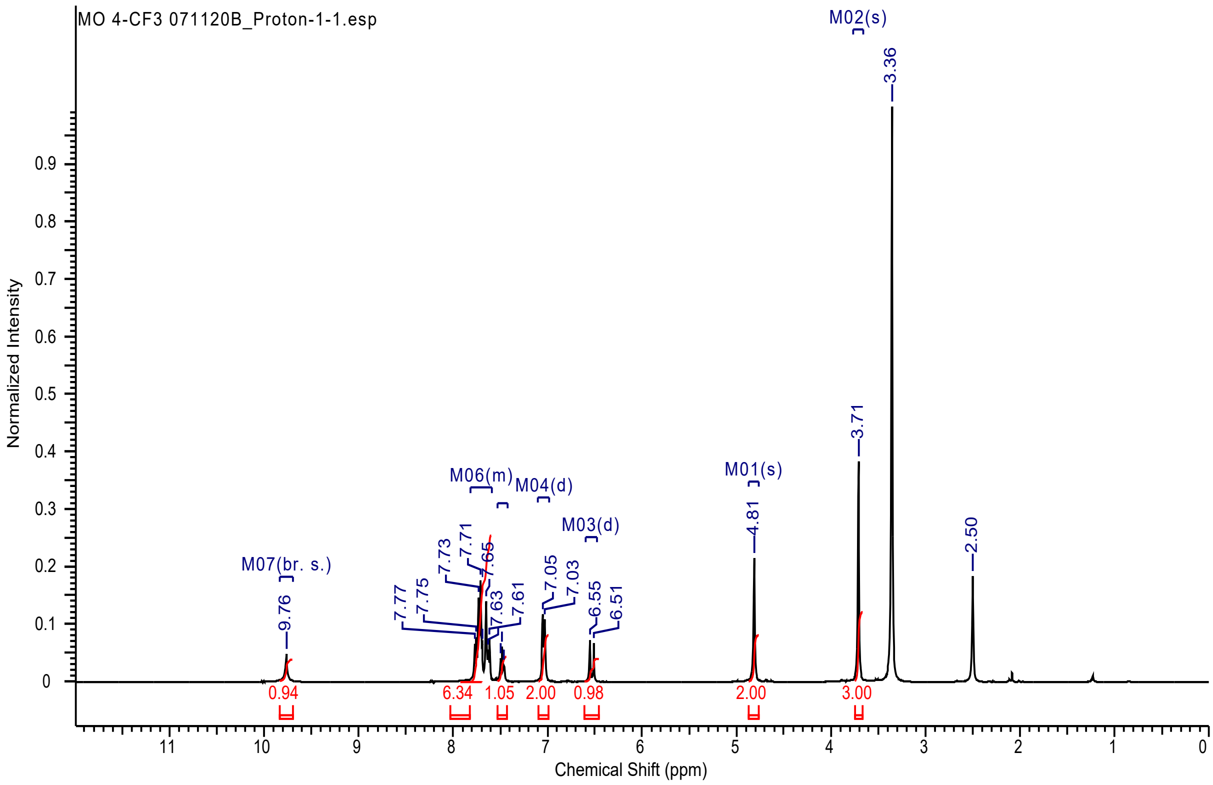

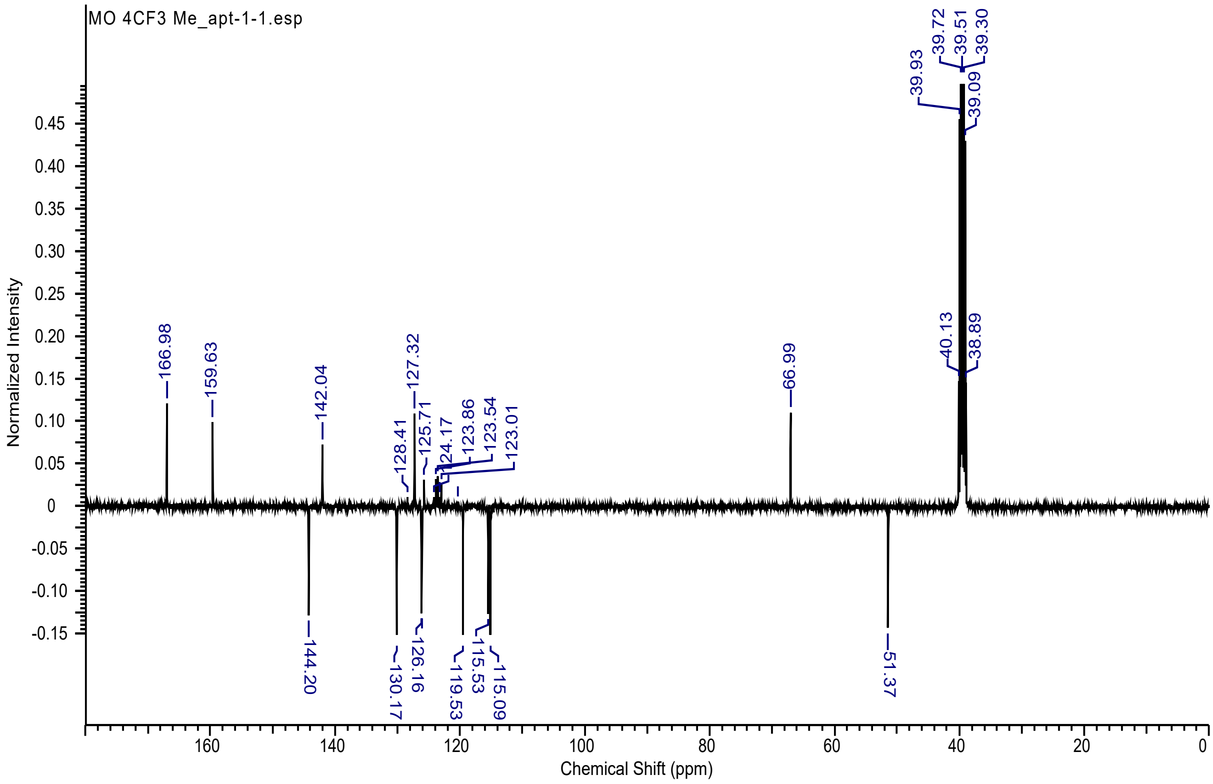

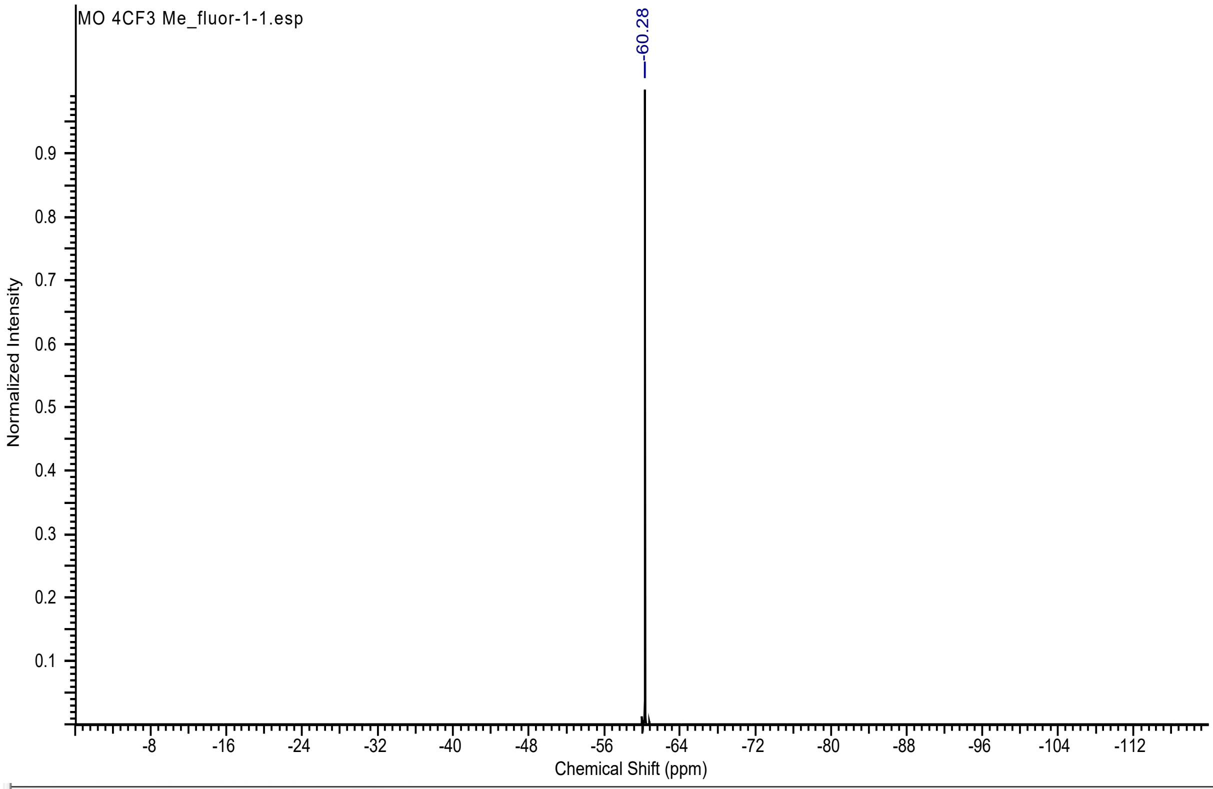
MO 4CF3 Me_fluor-1-1.esp
-60.28
Normalized Intensity
0.9
0.8
0.7
0.6
0.5
0.4
0.3
0.2
0.1
-8
-16
-24
-32
-40
-48
-56
-64
-72
-80
-88
-96
-104
-112
Chemical Shift (ppm)

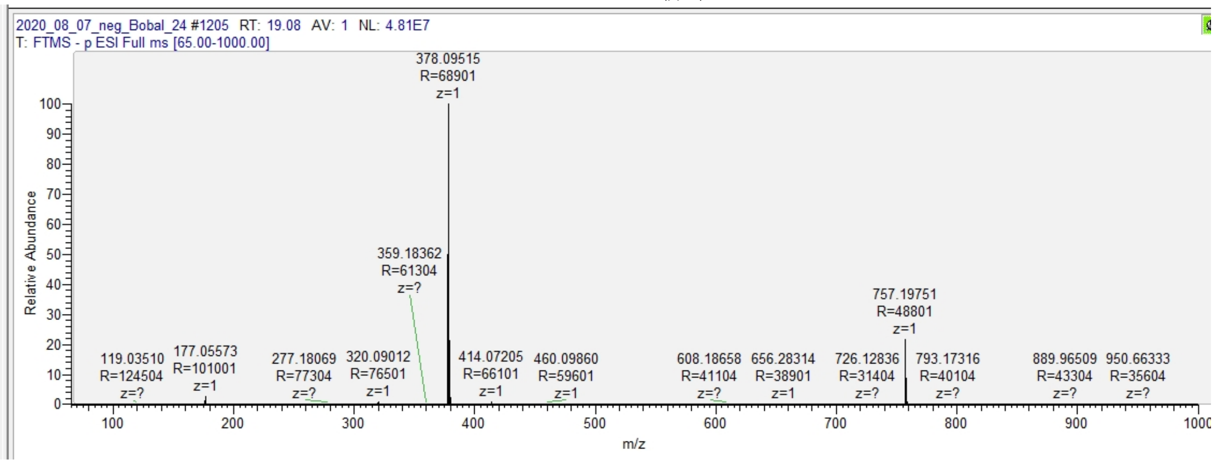
2020_08_07_neg_Bobal_24 #1205 RT: 19.08 AV: 1 NL: 4.81E7
T: FTMS - p ESI Full ms [65.00-1000.00]
378.09515
R=68901
z=1
359.18362
R=61304
z=?
757.19751
R=48801
z=1
119.03510
R=124504
z=?
177.05573
R=101001
z=1
277.18069
R=77304
z=?
320.09012
R=76501
z=1
414.07205
R=66101
z=1
460.09860
R=59601
z=1
608.18658
R=41104
z=?
656.28314
R=38901
z=1
726.12836
R=31404
z=?
793.17316
R=40104
z=?
889.96509
R=43304
z=?
950.66333
R=35604
z=?
Relative Abundance
m/z

Methyl (2*E*)-3-(4-{[(2-nitrophenyl)carbamoyl]methoxy}phenyl)prop-2-enoate (**10t**)

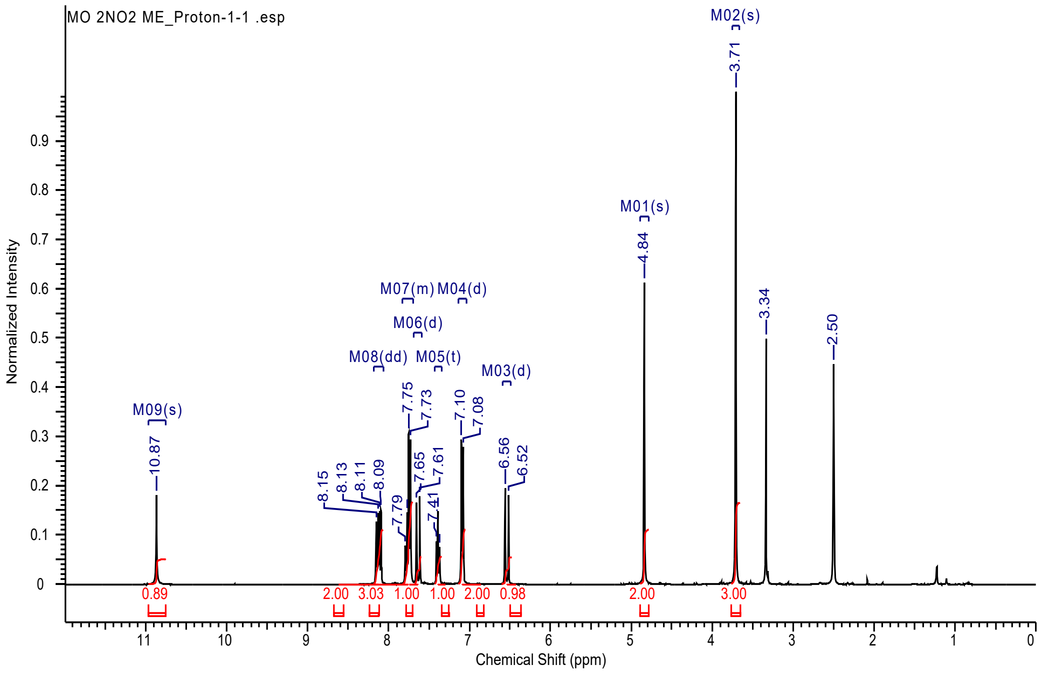

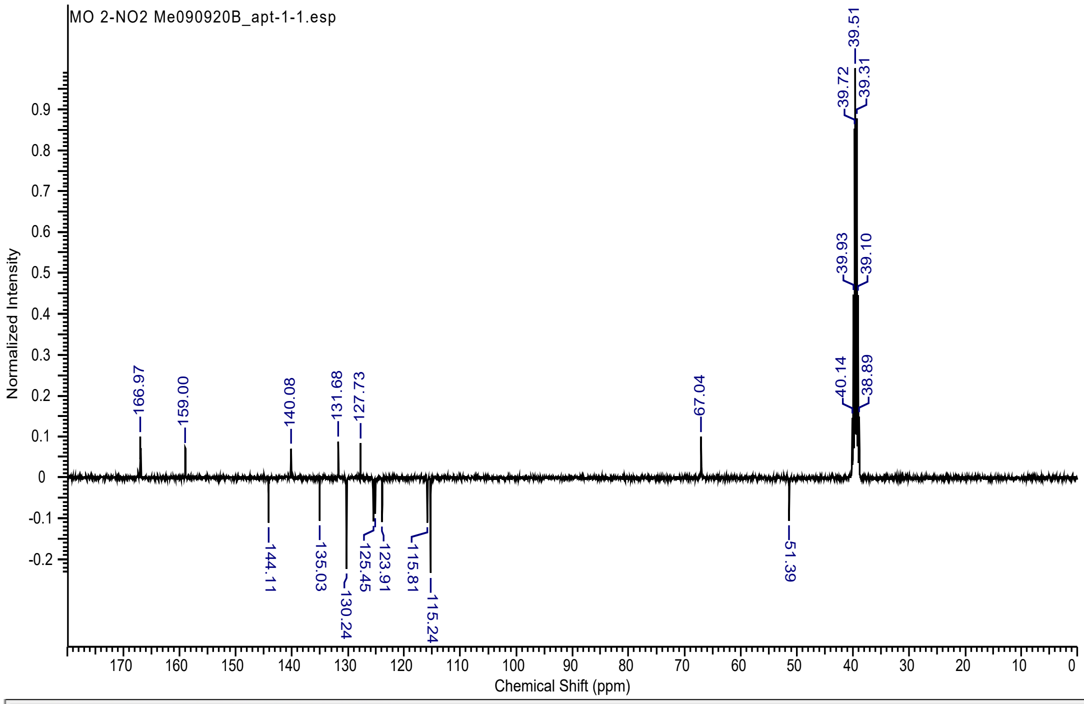

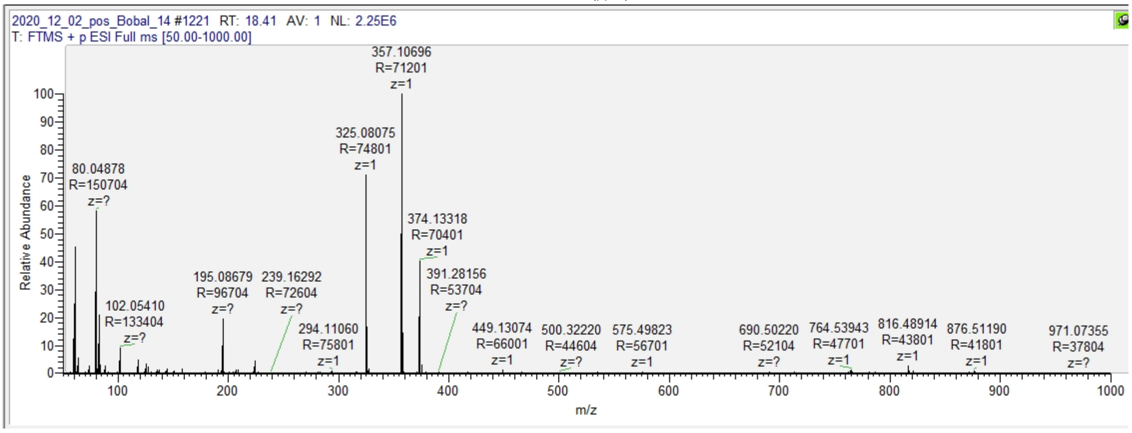

Chromatogram and Results

Injection Details

Injection Name:	**MO 2-NO2 ME**	*Run Time (min):*	**30,00**
Vial Number:	**GE6**	*Injection Volume:*	**10,00**
Injection Type:	**Unknown**	*Channel:*	**UV_VIS_1**
Calibration Level:		*Wavelength:*	**210,0**
Instrument Method:	**Grad40-60to70-30 MeCN-H2O**	*Bandwidth:*	**2**
Processing Method:	**New Processing Method**	*Dilution Factor:*	**1,0000**
Injection Date/Time:	**10.12.20 06:46**	*Sample Weight:*	**1,0000**

Chromatogram

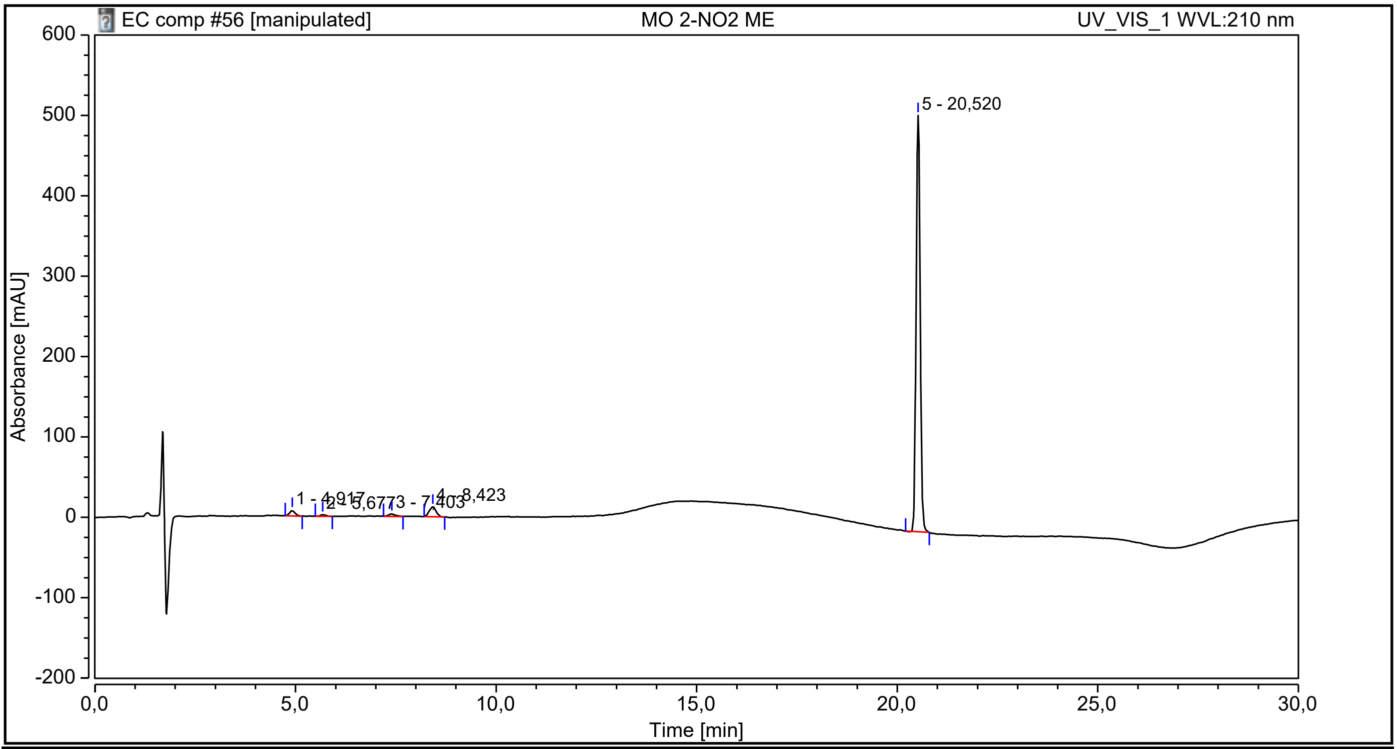

Integration Results

No.	Peak Name	Retention Time min	Area mAU*min	Height mAU	Relative Area %	Relative Height %	Amount n.a.
1	n.a.	4,917	1,076	6,927	1,632	1,28	n.a.
2	n.a.	5,677	0,285	1,809	0,433	0,33	n.a.
3	n.a.	7,403	0,587	3,118	0,890	0,58	n.a.
4	n.a.	8,423	2,428	12,266	3,681	2,26	n.a.
5	n.a.	20,520	61,589	517,967	93,364	95,55	n.a.
**Total:**			**65,967**	**542,087**	**100,00**	**100,00**	

Methyl (2*E*)-3-(4-{[(3-nitrophenyl)carbamoyl]methoxy}phenyl)prop-2-enoate (**10u**)

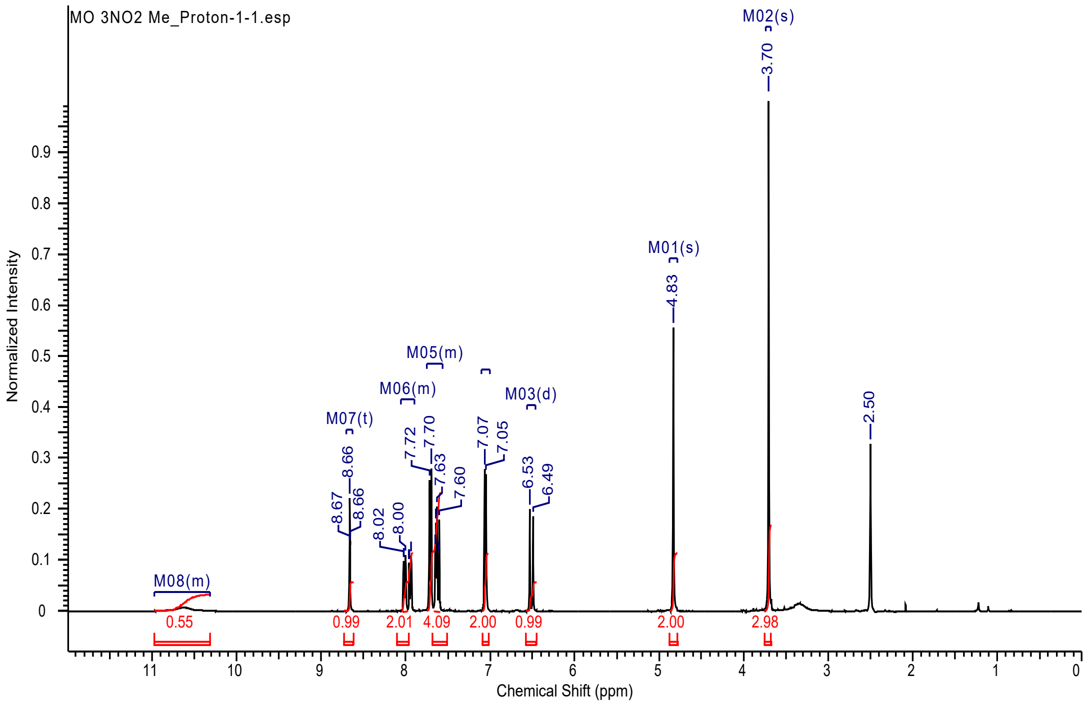

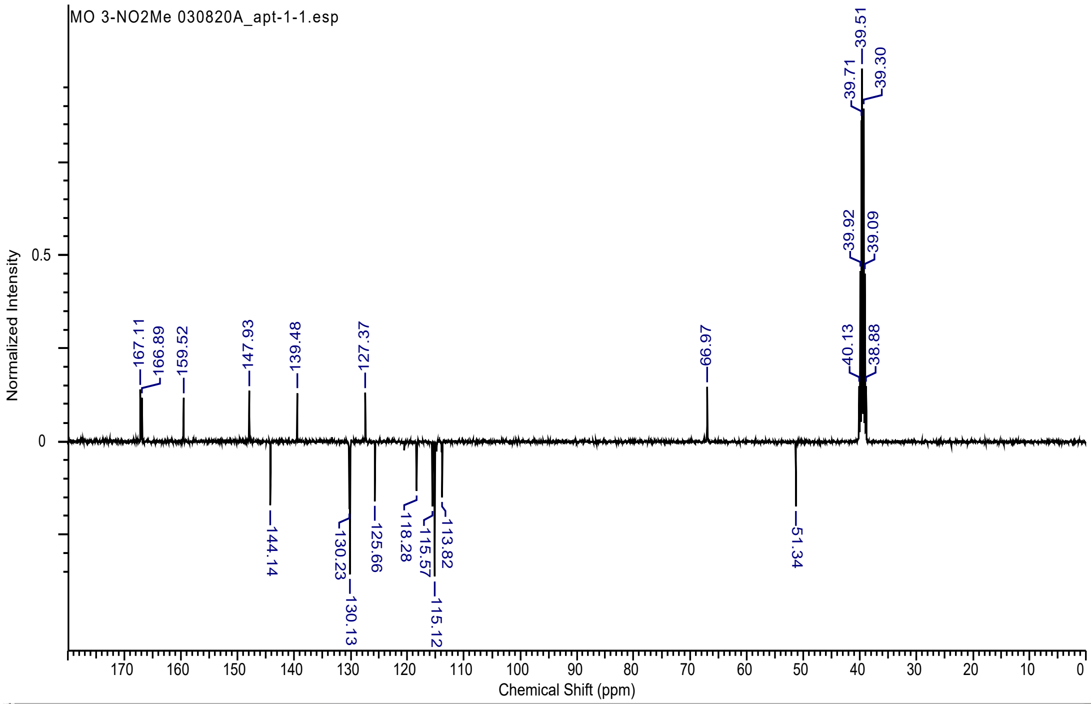

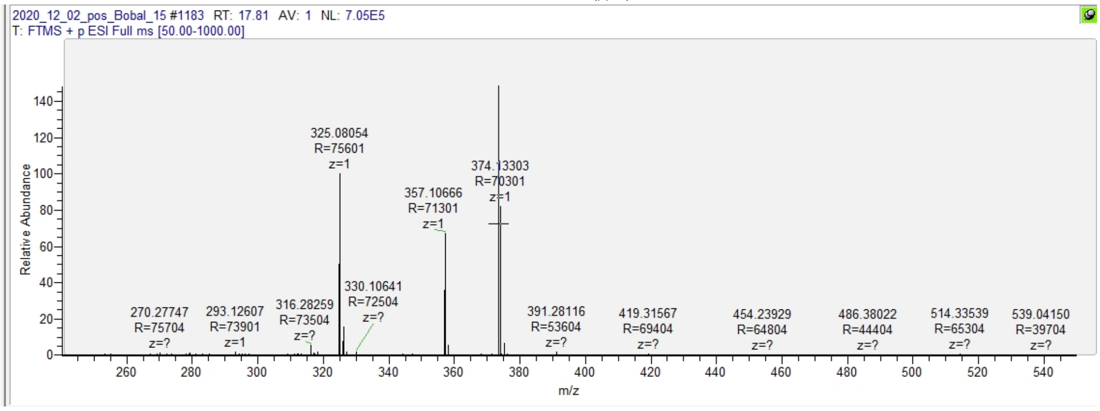

Methyl (2*E*)-3-(4-{[(4-nitrophenyl)carbamoyl]methoxy}phenyl)prop-2-enoate (**10v**)

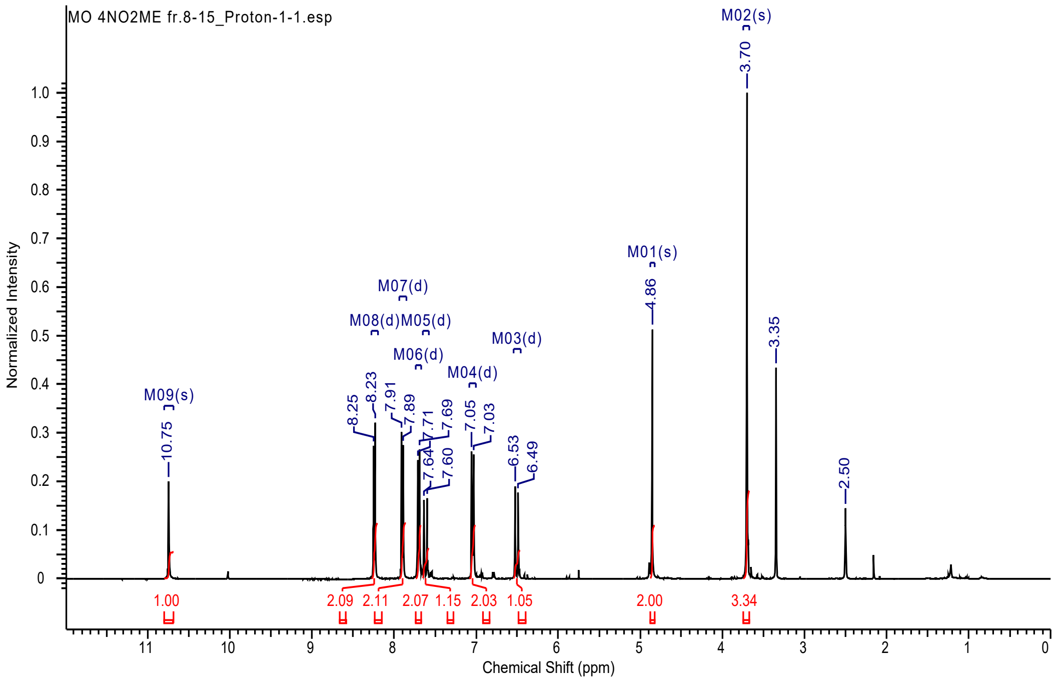

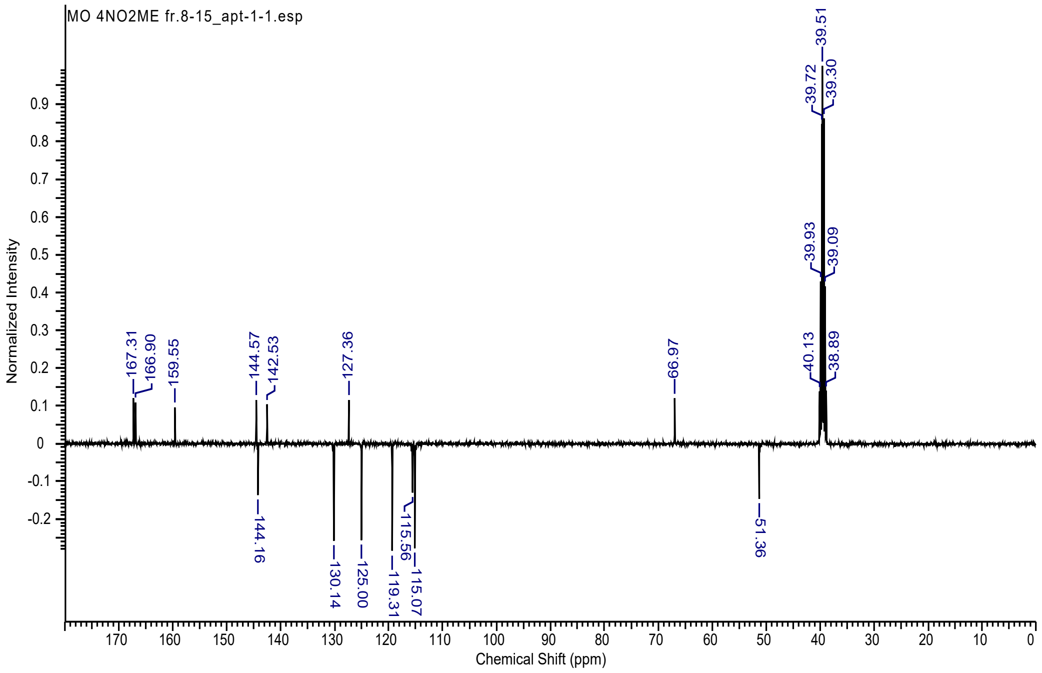

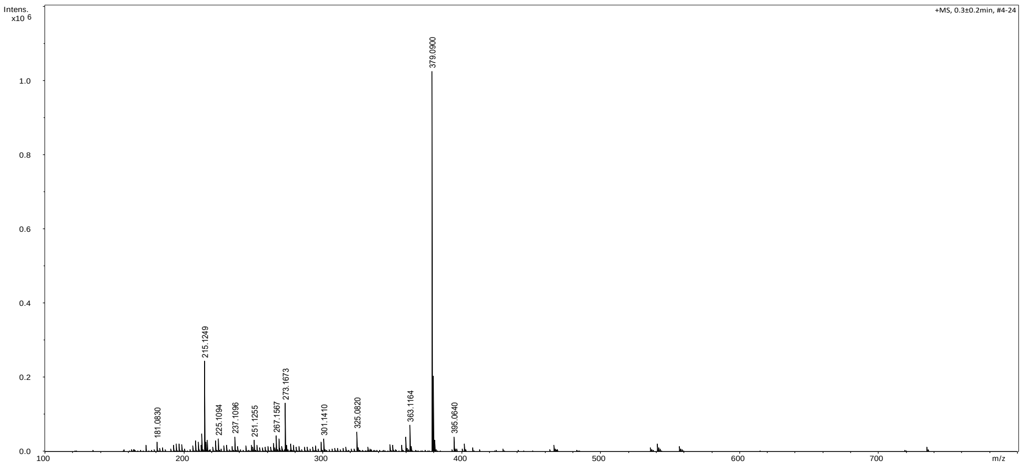

Chromatogram and Results

Injection Details

Injection Name:	**MO 4- NO2 ME**	*Run Time (min):*	**30,00**
Vial Number:	**RA6**	*Injection Volume:*	**10,00**
Injection Type:	**Unknown**	*Channel:*	**UV_VIS_1**
Calibration Level:		*Wavelength:*	**210,0**
Instrument Method:	**Grad40-60to90-10 MeCN-H2O**	*Bandwidth:*	**2**
Processing Method:	**New Processing Method**	*Dilution Factor:*	**1,0000**
Injection Date/Time:	**20.10.22 13:54**	*Sample Weight:*	**1,0000**

Chromatogram

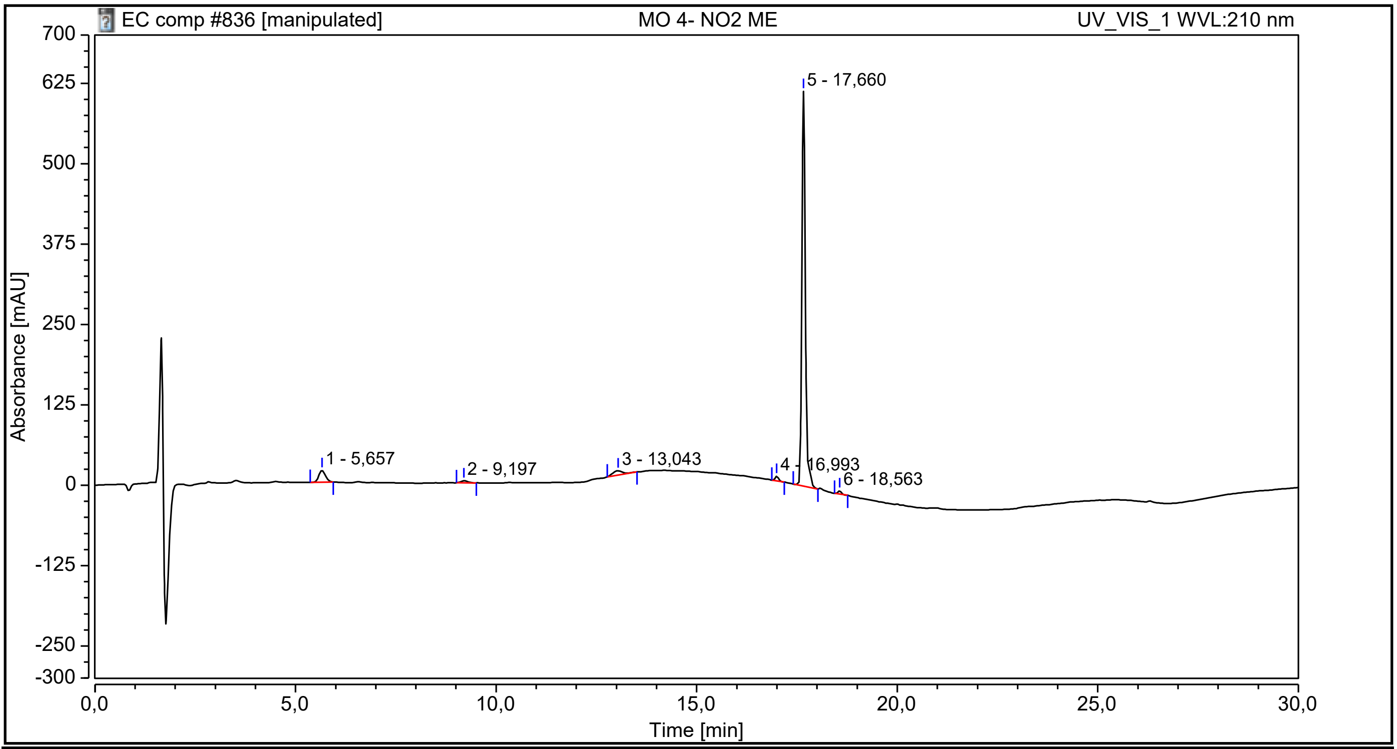

Integration Results

No.	Peak Name	Retention Time min	Area mAU*min	Height mAU	Relative Area %	Relative Height %	Amount n.a.
1	n.a.	5,657	3,467	18,115	5,028	2,77	n.a.
2	n.a.	9,197	0,634	3,104	0,920	0,47	n.a.
3	n.a.	13,043	1,991	6,716	2,887	1,03	n.a.
4	n.a.	16,993	0,658	6,993	0,954	1,07	n.a.
5	n.a.	17,660	61,764	613,855	89,568	93,88	n.a.
6	n.a.	18,563	0,443	5,076	0,642	0,78	n.a.
**Total:**			**68,958**	**653,860**	**100,00**	**100,00**	

Suberanilic acid (**15**)

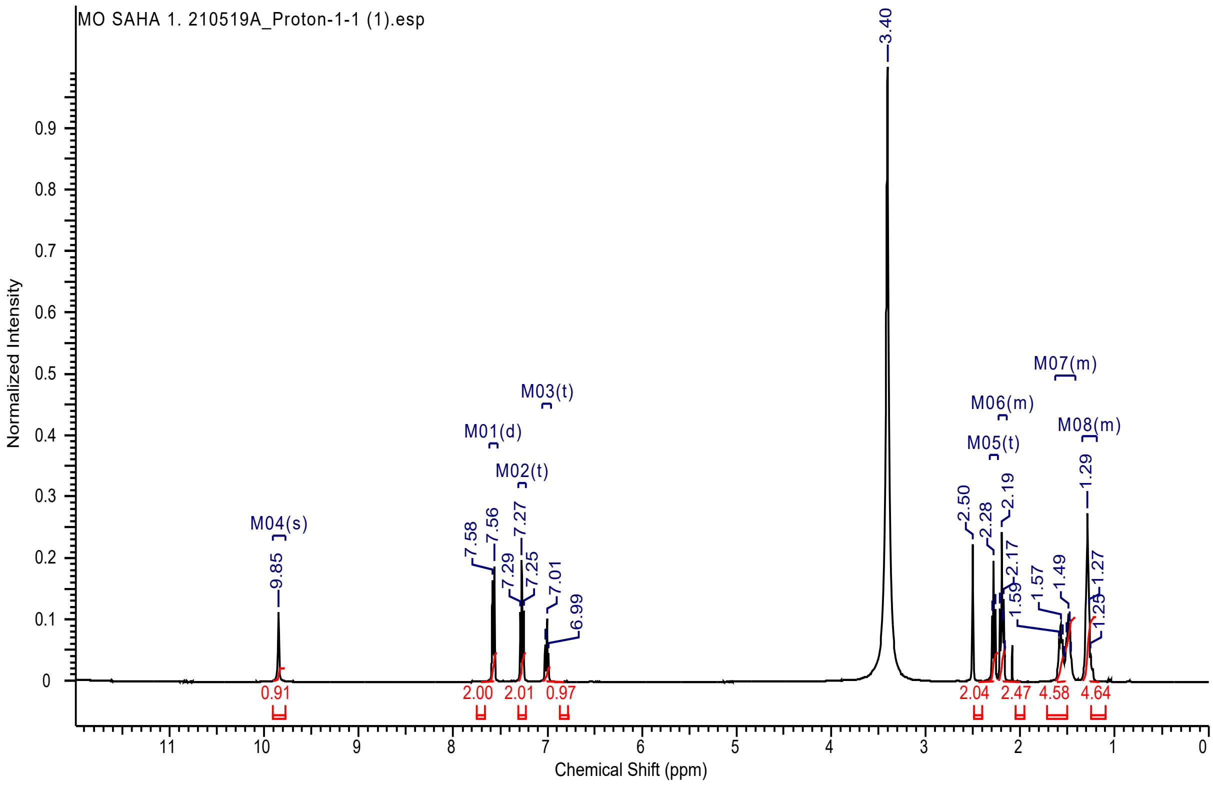

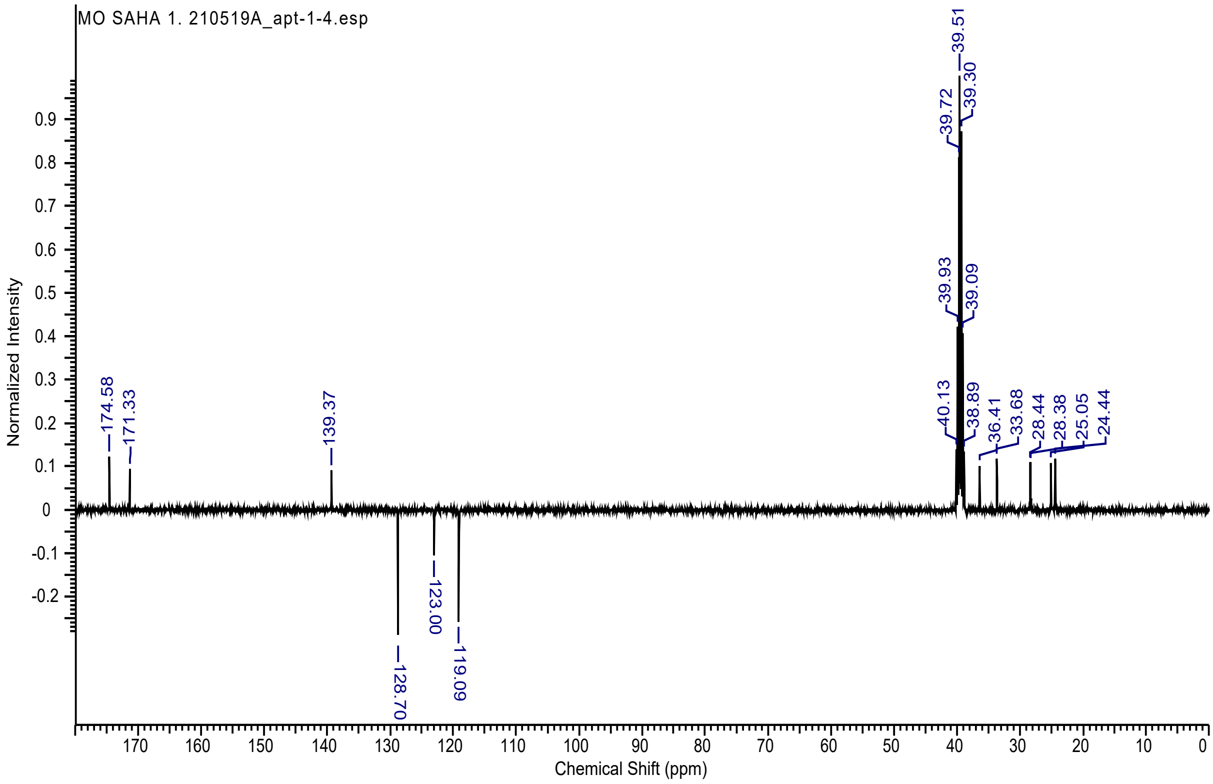

Methyl Suberanilate (**16**)

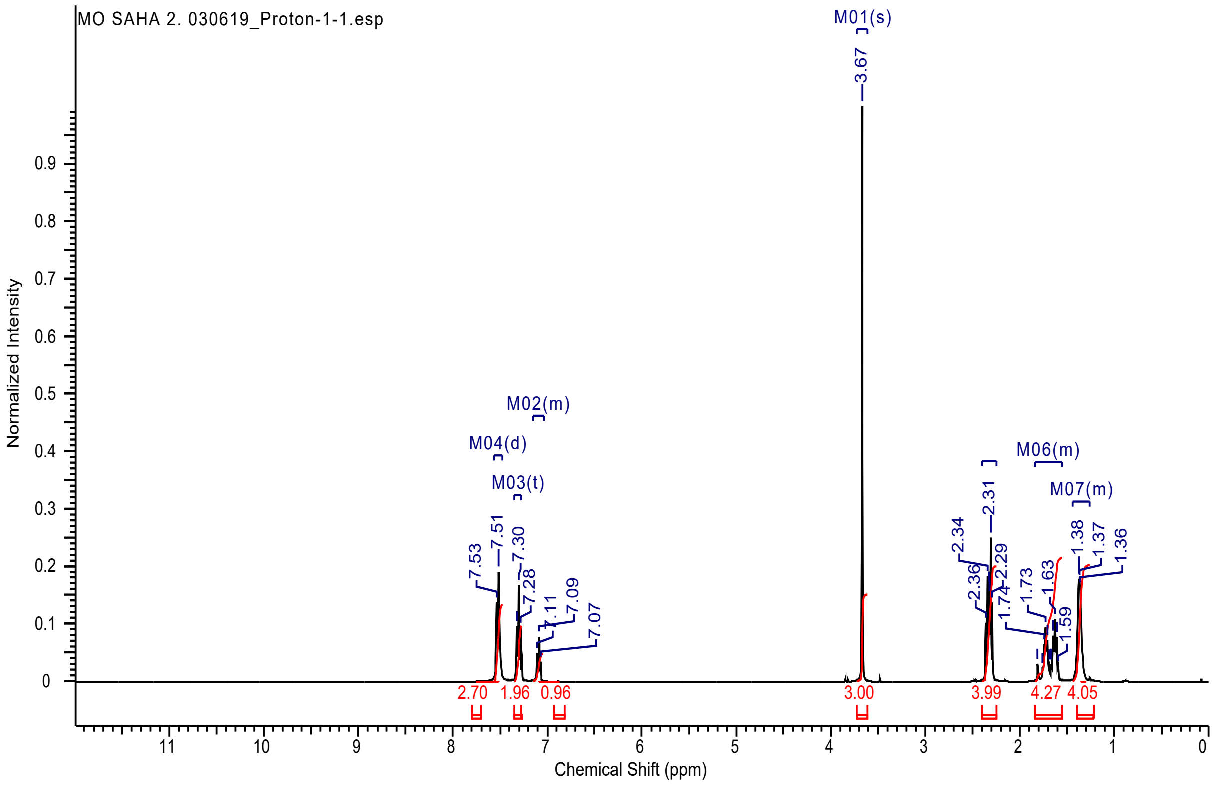

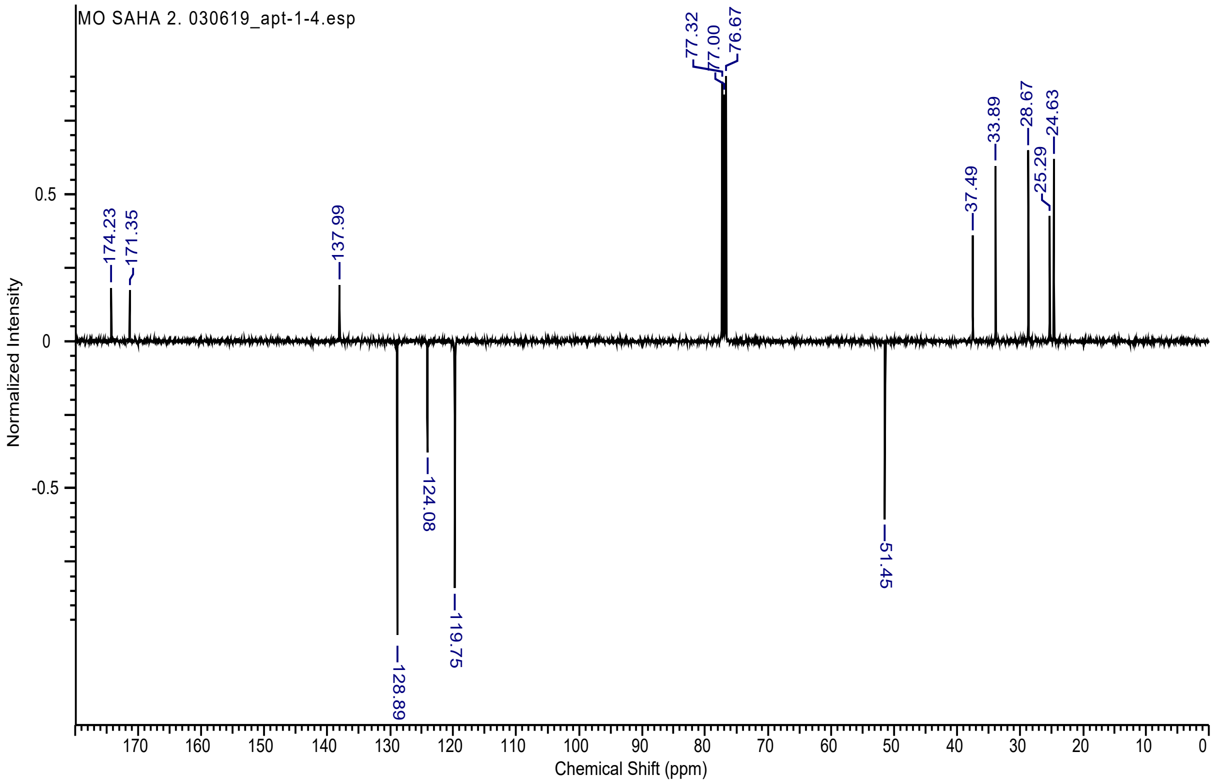

In Silico Modeling and Structural Studies

Table S1. XP GlideScore for new hydroxamic acid derivatives **7a** - **7v** (Fig. 3) and reference inhibitor vorinostat **1** in [kcal·mol^{-1}] computed for bidenate type **A** chelation of the zinc ion (Fig. M1) for selected class I and class II HDACs: HDAC1, 2, 3, 8 and 4, 6, 7.

**A - Bidentate binding mode via two oxygens, neutral hydroxamate -C=O-NH-OH**							
**HDACs**	**HDAC1**	**HDAC2**	**HDAC3**	**HDAC4**	**HDAC6**	**HDAC7**	**HDAC8**
**Ligand**	***5ICN***	***7KBG***	***4A69***	***2VQM***	***5EDU***	***3ZNR***	***5VI6***
**Vorinostat 1**	-8.0	-4.2	-6.2	-9.7	-6.9	-5.6	-8.3
**7a**	-8.9	-5.5	n/a	-7.8	-5.7	-4.1	-8.2
**7b**	-7.3	-5.5	-8.2	-7.9	-2.7	-4.3	-8.9
**7c**	-8.9	-5.6	n/a	-7.9	-2.9	-4.0	-7.9
**7d**	-9.4	-5.9	-8.6	-7.8	-5.6	-3.9	-8.5
**7e**	-9.3	-5.0	-8.2	-7.5	-6.4	-3.5	-8.9
**7f**	-8.6	-5.4	-8.3	-8.3	-6.0	-3.8	-8.4
**7g**	-9.1	-5.8	-9.6	-7.7	-6.4	-3.2	-7.8
**7h**	-9.3	-5.4	-8.8	-8.0	-5.2	-3.7	-8.5
**7i**	-7.7	-4.8	-8.1	-8.1	-5.6	-3.2	-8.7
**7j**	-8.9	-5.4	n/a	-7.6	-3.1	-3.7	-8.5
**7k**	-9.0	-5.2	-8.7	-7.4	-5.5	-3.3	-8.7
**7l**	-7.7	-5.5	-8.1	-8.1	-5.1	-3.8	-8.3
**7m**	-9.5	-5.5	n/a	-7.9	-6.3	-3.5	-8.4
**7n**	-9.2	-5.1	-8.6	-7.8	-5.3	-3.7	-8.8
**7o**	-7.6	-5.2	-7.7	-7.9	-5.4	-3.8	-8.3
**7p**	-9.3	-5.6	-8.4	-7.6	-3.4	0.1	-8.4
**7q**	-8.6	-5.2	-7.6	-7.6	-5.0	-3.7	-8.6
**7r**	-8.7	-5.5	-8.4	-7.6	-9.4	-3.5	-8.1
**7s**	-8.7	-5.3	-8.3	-6.9	-6.6	-3.4	-8.5
**7t**	-9.2	-5.7	-8.8	-7.7	-4.2	-3.6	-8.7
**7u**	-8.6	-5.1	-8.9	-7.9	-6.8	-3.6	-7.9
**7v**	-8.8	-5.4	n/a	-7.5	-8.7	-3.5	-7.3
**Average A**	-8.7 ± 0.6	-5.4 ± 0.3	-8.3 ± 0.6	-7.7 ± 0.3	-5.5 ± 1.6	-3.5 ± 0.8	-8.4 ± 0.4

* Average XP GlideScore in [kcal·mol^{-1}] ± standard deviation over 22 hydroxamate inhibitors **7a** - **7v**

Table S2. XP GlideScore for new hydroxamic acid derivatives **7a** - **7v** (Fig. 3) and reference inhibitor vorinostat **1** in [kcal·mol^{-1}] computed for monodenate type **B** chelation of the zinc ion (Fig. M1) for selected class I and class II HDACs: HDAC1, 2, 3, 8 and 4, 6, 7.

**B - Monodenate binding mode via carbonyl oxygen, neutral hydroxamate -C=O-NH-OH**							
**HDACs**	**HDAC1**	**HDAC2**	**HDAC3**	**HDAC4**	**HDAC6**	**HDAC7**	**HDAC8**
**Ligand**	***5ICN***	***7KBG***	***4A69***	***2VQM***	***5EDU***	***3ZNR***	***5VI6***
**Vorinostat 1**	-6.6	-9.5	-9.1	-7.0	n/a	-1.1	-6.2
**7a**	-6.5	-8.9	-9.3	-5.3	-8.7	-1.6	-8.4
**7b**	-6.5	-9.0	-9.3	-5.3	-9.5	-5.6	-6.8
**7c**	-5.3	-9.0	-8.7	-5.4	-8.0	-5.5	-8.8
**7d**	-6.5	-9.3	-9.6	-5.0	-9.0	-1.3	-8.8
**7e**	-6.8	-8.7	-9.3	-5.3	-8.5	-1.6	-8.6
**7f**	-7.4	-8.9	-9.5	-5.2	-9.5	-1.5	-6.8
**7g**	-6.5	-9.1	-9.6	-5.0	-9.1	-0.8	-9.2
**7h**	-6.5	-9.3	-9.3	-5.3	-9.4	-1.6	-8.6
**7i**	-6.9	-8.8	-9.0	-5.2	-9.2	-1.5	-8.0
**7j**	-7.0	-8.8	-9.4	-5.0	-7.8	-1.5	-8.7
**7k**	-6.4	-9.1	-8.9	-5.3	-9.5	-1.7	-8.5
**7l**	-6.4	-9.1	-8.3	-5.3	-9.3	-1.5	-8.4
**7m**	-6.5	-9.2	-9.4	-4.9	-9.0	-1.0	-7.8
**7n**	-6.4	-8.8	-7.0	-5.3	-9.0	-1.4	-8.5
**7o**	-6.4	-8.7	-8.0	-5.1	-9.4	-1.2	-8.3
**7p**	-6.9	-9.1	-9.1	-4.8	-8.6	-1.2	-7.7
**7q**	-6.5	-8.4	-9.1	-5.2	-8.5	-1.5	-8.4
**7r**	-4.6	-8.8	-8.8	-5.0	-9.0	-1.3	-6.5
**7s**	-6.2	-8.7	-9.2	-4.7	-9.0	-0.7	-8.5
**7t**	-6.9	-8.7	-9.0	-4.8	-8.6	-1.6	-8.8
**7u**	-6.4	-8.9	-9.3	-4.9	-9.2	-1.5	-8.6
**7v**	-6.5	-8.9	-9.3	-4.7	-8.8	-1.3	-8.8
**Average B**	-6.4 ± 0.6	-8.9 ± 0.2	-9.0 ± 0.6	-5.1 ± 0.2	-8.9 ± 0.5	-1.7 ± 1.2	-8.2 ± 0.7

* Average XP GlideScore in [kcal·mol^{-1}] ± standard deviation over 22 hydroxamate inhibitors **7a** - **7v**

Analysis of Subdiploid Cell Population

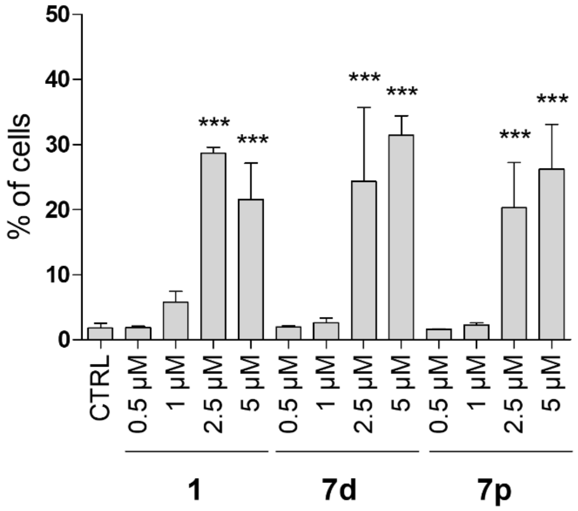

Figure S1. Compounds **7d** and **7p** increase the frequency of the THP-1 cell subdiploid population. THP-1 cells were treated with indicated concentrations of compounds **1**, **7d**, or **7p** for 48 h, and cells were fixed with ethanol, stained by PI, and analyzed by flow cytometry. The results are shown as the mean ± SD of three independent experiments. *** $p < 0.001$, significantly different from drug-free control (CTRL).

ADMET-related properties

Table S3 shows predicted physicochemical and ADMET properties of the studied compounds, which might be of interest.

The molar weight (MW), octanol/water partition coefficient (QlogPo/w) [1], water solubility (QlogS) [2-4], IC_{50} value for blockage of HERG K+ channels (QPlogHERG) [5,6], binding to human serum albumin (QPlogKhsa) [7], blood/braind partition coefficient (QPlogBB) [8], and % of human oral absorption [9] were computed using QikProp from Schrödinger Suit [10]. The human intestinal absorption (HIA) [11], P-glycoprotein substrate [12], and P-glycoprotein inhibitor [13,14] categories have been predicted using admetSAR [15].

The molar weights (MW), lipophilicities, and water solubilities of all the compounds were within normal values, suggesting good oral absorption. This supports the predicted results of high human oral absorption, ranging between 54% and 81%, and positive human intestinal absorption. Binding to blood plasma proteins was rather low. The predicted values of QPlogBB showed that the compounds could not cross the blood brain barrier. They are neither P-glycoprotein substrates nor P-glycoprotein inhibitors. However, QPlogHERG values could raise some caution about the possibility of inducing the long QT syndrome.

Table S3. Key physicochemical parameters and ADMET attributes of the investigated compounds

Comp.	MW	QP logPo/w	QP logS	QPlog HERG	QP logKhsa	QP logBB	% of Human Oral Absorption	HIA	P-glyco protein substrate	P- glyco protein inhibitor
**7a**	312.324	1.605	-3.695	-6.510	-0.332	-1.935	75.041	+	-	-
**7b**	326.351	1.845	-3.944	-6.283	-0.231	-1.870	77.425	+	-	-
**7c**	326.351	1.849	-4.151	-6.429	-0.222	-1.971	76.751	+	-	-
**7d**	326.351	1.842	-4.139	-6.427	-0.225	-1.970	76.715	+	-	-
**7e**	342.351	1.712	-3.814	-6.301	-0.320	-1.983	76.042	+	-	-
**7f**	342.351	1.670	-3.894	-6.486	-0.348	-2.026	75.874	+	-	-
**7g**	342.351	1.670	-3.890	-6.438	-0.342	-2.030	75.719	+	-	-
**7h**	330.315	1.767	-3.885	-6.413	-0.326	-1.796	76.880	+	-	-
**7i**	330.315	1.786	-3.962	-6.373	-0.319	-1.814	76.363	+	-	-
**7j**	330.315	1.783	-3.948	-6.356	-0.320	-1.810	76.342	+	-	-
**7k**	346.769	1.957	-3.936	-6.155	-0.272	-1.666	78.534	+	-	-
**7l**	346.769	2.037	-4.297	-6.395	-0.258	-1.767	77.958	+	-	-
**7m**	346.769	2.030	-4.305	-6.405	-0.259	-1.777	77.802	+	-	-
**7n**	391.220	2.002	-4.074	-6.227	-0.256	-1.710	78.219	+	-	-
**7o**	391.220	2.109	-4.401	-6.422	-0.239	-1.762	78.378	+	-	-
**7p**	391.220	2.102	-4.409	-6.431	-0.240	-1.773	78.222	+	-	-
**7q**	380.323	2.372	-4.417	-6.113	-0.164	-1.565	81.244	+	-	-
**7r**	380.323	2.506	-4.966	-6.452	-0.137	-1.708	80.581	+	-	-
**7s**	380.323	2.507	-4.977	-6.470	-0.137	-1.711	80.619	+	-	-
**7t**	357.322	0.978	-3.678	-6.413	-0.403	-2.833	58.311	+	-	-
**7u**	357.322	0.875	-3.791	-6.413	-0.392	-3.057	54.761	+	-	-
**7v**	357.322	0.877	-3.802	-6.428	-0.392	-3.062	54.782	+	-	-
**Panobinostat**	349.432	2.392	-3.504	-6.993	0.138	-1.444	71.586	+	-	-
**Vorinostat**	264.324	0.739	-2.167	-4.381	-0.755	-1.842	67.740	+	-	-

References

1. Duffy, E. M.; Jorgensen, W. L. Prediction of Properties from Simulations: Free Energies of Solvation in Hexadecane, Octanol, and Water. *J. Am. Chem. Soc.* **2000,** *122*, 2878-2888.

2. Jorgensen, W. L.; Duffy, E. M. Prediction of Drug Solubility from Structure. *Adv. Drug Delivery Rev.* **2002,** *54,* 355-366.

3. Lipinski, C. A.; Lombardo, F.; Dominy, B. W.; Feeney, P. J. Experimental and computational approaches to estimate solubility and permeability in drug discovery and development settings, *Adv. Drug Delivery Rev.* **2001**, *46*, 3-26.

4. Jorgensen, W. L.; Duffy, E. M. Prediction of Drug Solubility from Monte Carlo Simulations. *Bioorg. Med. Chem. Lett.* **2000,** *10,* 1155-1158.

5. Cavalli, A.; Poluzzi, E.; De Ponti, F.; Recanatini, M. Toward a Pharmacophore for Drugs Inducing the Long QT Syndrome: Insights from a CoMFA Study of HERG K^+ Channel Blockers *J. Med. Chem.* **2002**, *45*, 3844–3853.

6. De Ponti, F.; Poluzzi, E.; Montanaro, N. Organising evidence on QT prolongation and occurrence of Torsades de Pointes with non-antiarrhythmic drugs: a call for consensus. *Eur. J. Clin. Pharmacol*. **2001**, *57,* 185–209.

7. Colmenarejo, G.; Alvarez-Pedraglio, A.; Lavandera, J.-L. Cheminformatic Models To Predict Binding Affinities to Human Serum Albumin. *J. Med. Chem.* **2001,** *44,* 4370-4378.

8. Luco, J. M. Prediction of the Brain-Blood Distribution of a Large Set of Drugs from Structurally Derived Descriptors Using Partial Least-Squares (PLS) Modeling. *J. Chem. Inf. Comput. Sci.* **1999**, *39*, 396-404.

9. Stenberg, P.; Norinder, U.; Luthman, K.; Artursson, P. Experimental and Computational Screening Models for the Prediction of Intestinal Drug Absorption. *J. Med. Chem.* **2001**, *44*, 1927–1937.

10. Schrödinger Release 2024-4: QikProp, Schrödinger, LLC, New York, NY, 2024.

11. Jie Shen, Feixiong Cheng, You Xu, Weihua Li, and Yun Tang, Estimation of ADME Properties with Substructure Pattern Recognition, *J. Chem. Inf. Model.* **2010**, *50*(6), 1034-1041.

12. Zhi Wang, Yuanying Chen, Hu Liang, Andreas Bender, Robert C. Glen, and Aixia Yan, P-glycoprotein Substrate Models Using Support Vector Machines Based on a Comprehensive Data set, *J. Chem. Inf. Model.* **2011**, *51*(6), 1447-1456.

13. Lei Chen, Youyong Li, Qing Zhao, Hui Peng, and Tingjun Hou, ADME Evaluation in Drug Discovery. 10. Predictions of P-Glycoprotein Inhibitors Using Recursive Partitioning and Naive Bayesian Classification Techniques, *Mol. Pharmaceutics.* **2011**, *8*(3), 889-900.

14. Fabio Broccatelli, Emanuele Carosati, Annalisa Neri, Maria Frosini, Laura Goracci, Tudor I. Oprea, and Gabriele Cruciani, A Novel Approach for Predicting P-Glycoprotein (ABCB1) Inhibition Using Molecular Interaction Fields, *J. Med. Chem.* **2011**, *54*(6), 1740-1751.

15. Feixiong Cheng, Weihua Li, Yadi Zhou, Jie Shen, Zengrui Wu, Guixia Liu, Philip W. Lee, Yun Tang. admetSAR: a comprehensive source and free tool for evaluating chemical ADMET properties. *J. Chem. Inf. Model.,* **2012**, *52*(11), 3099-3105.
